# Urbanization development characteristics coincide with elevated Anopheles stephensi habitat suitability

**DOI:** 10.1038/s43247-026-03833-0

**Published:** 2026-07-22

**Authors:** Yelin Sun, Weihang Liu, Qinmei Han, Haoqiang Ji, Meng Shang, Xiaoxu Wang, Zhenxu Wang, Wenqin Liang, Qiyong Liu

**Affiliations:** 1 National Key Laboratory of Intelligent Tracking and Forecasting for Infectious Diseases, National Institute for Communicable Disease Control and Prevention, Chinese Center for Disease Control and Prevention & Chinese Academy of Preventive Medicine, Beijing, China; 2 School of Government, Nanjing University, Nanjing, China; 3 National climate center, China Meteorological Administration, Beijing, China; 4 Shandong Center for Disease Control and Prevention; Shandong Provincial Academy of Preventive Medicine, Jinan, China; 5 Vector Surveillance Division, Laboratory Center, Guizhou Center for Disease Control and Prevention, Guiyang, China

**Keywords:** Environmental sciences, Ecology

## Abstract

The invasion of Anopheles stephensi into Africa is increasing risks in urban areas, posing challenges to global malaria elimination. Unlike most malaria vectors, Anopheles stephensi is well adapted to urban environments, thereby imposing a substantial burden on urban malaria transmission. Here, we quantify the influence of urbanization on Anopheles stephensi habitat suitability and examine potential underlying mechanisms. Habitat suitability increases along the urbanization gradient, with the highest suitability observed in high-density urban regions. Urbanization increases the habitat suitability, particularly in Africa, with the transition from urban to high-density urban areas showing the greatest increase in suitability. Moreover, we identify construction-related activities as a key pathway linking urbanization to habitat suitability, consistent with the proliferation of artificial larval habitats in built environments. These findings provide a mechanistic understanding of urban-associated risk, inform large-scale malaria predictions to support the integration of vector surveillance with urban planning.

## Abstract (web summary)

Habitat suitability for Anopheles stephensi rises with urbanization, peaking in high-density cities, and construction activity drives this increase, heightening Africa’s urban malaria risk, according to a statistical analysis of habitat suitability and urbanization dynamics.

## Introduction

P*lasmodium falciparum* and *Plasmodium vivax* are both effectively transmitted by the malaria vector *Anopheles stephensi* (*An. stephensi*)^1^. Prior to 2011, *An. stephensi* was the major malaria vector found mainly in South Asia and the Arabian Peninsula^2^. However, *An. stephensi* has invaded Africa since 2012^2^, and found in Djibouti (2012), Ethiopia (2016), the Republic of the Sudan (2019) and Ghana (2023)^3,4^. Malaria outbreaks were strongly associated with the discovery of *An. stephensi* in Ethiopia and Djibouti^5^, which has created challenges for global malaria elimination^6^.

*An. stephensi* is an anthropophilic mosquito that exhibits both endophilic (resting indoors) and endophagic (feeding indoors) behaviors, with peak activity during crepuscular and nocturnal hours^7^. Like other mosquitoes, *An. stephensi* undergoes four stages of growth and development, including eggs, larval, pupae, adult stages; the larval and pupae are exclusively aquatic utilizing still- or slow-moving-water, while adults are land-based^8^. *An. stephensi* occurs in three known forms – ‘type’, ‘intermediate’, and ‘mysorensis’ – with the type and intermediate forms serving as efficient vectors in both rural and urban environments^7^, which is different from other *Anopheles* mosquitoes transmitted malaria^9^. Moreover, *An. stephensi* can survive extremely high temperature of the dry season^7^, enabling it to adapt to the African environment^10^ and may increase the malaria risk in African cities^11^.

Vector control is critical to eliminate malaria^12^. Recent Asian systematic reviews indicate that long-lasting insecticidal nets (LLINs) and indoor residual spraying (IRS) remain effective for controlling *An. stephensi*-transmitted malaria^13^. The widespread emergency of insecticide resistance poses a serious challenge to sustained chemical control^14^. These challenges underscore the need for alternative, environmentally-based interventions that target larval habitat of *An. stephensi*. Therefore, it is crucial to obtain accurate maps of the potential distribution of *An. stephensi* to facilitate effective monitoring and eradication strategies. However, most of this evidence originates from the species’ native range in Asia, where its ecology and behavior have been studied for decades. In contrast, research on *An. stephensi* in its newly invaded African range remains limited. In addition, local entomological surveys have identified artificial environments as important larval habitats of *An. stephensi*^15,16^, providing valuable mechanistic insights into how urban settings may support mosquito larvae. However, the quantitative impact of urbanization on the habitat suitability of *An. stephensi* still needs to be analyzed, which is crucial for future malaria risk assessments on a large scale.

A key prerequisite for targeted control is a clear understanding of where suitable habitats for this species are most likely to occur. Large-scale potential distribution modeling studies have included multiple factors and mapped potential habitat suitability across regions^17–19^, providing scientific basis for vector surveillance. The quantitative analysis of the mechanisms by which urbanization affects *An. stephensi* habitat suitability enables a more robust identification of where and when risk may increase under ongoing urbanization. It can more accurately predict the future potential distribution of suitable habitats for *An. stephensi*, helping to identify regions that may benefit from enhanced disease surveillance and facilitating more precise resource allocation.

Here, we investigated how urbanization drives the changes in habitat suitability of *An. stephensi*. Using boosted regression trees (BRT) model and partial squares path modeling (PLS-PM), the study found that the historical dynamics of habitat suitability accelerated by urbanization and revealed that construction-related activities served as a key mechanistic pathway linking urbanization to the changes in habitat suitability. This study improves the identification of areas at risk under ongoing urban expansion and offers a robust basis for prioritizing vector control and surveillance efforts.

## Results

### Model validation and feature contribution

We selected 25 climate, land use and population variables, which might be related to suitability habitat of *An. stephensi*, and the details about the variables were provided in Table S1. We contained 14 variables into BRT model through multicollinearity analysis (Fig. S1). Due to sampling preferences and survey accessibility, we generated 10 sets of background points with the same spatial bias as the occurrence points. Each set was randomly sampled 10 times, with 80% of the data for training and 20% for testing. The process was repeated 100 times for BRT model, and the final result was the average of 100 outcomes. The models with 100 iterations performed accurately with a mean area-under-the-curve (AUC) of 0.900 (95% CI: 0.896–0.903), a mean sensitivity of 0.822 (95% CI: 0.809–0.835) and a mean specificity of 0.842 (95% CI: 0.830–0.854), showing model performance was good for simulating habitat suitability of *An. stephensi*.

**Fig. 1:**
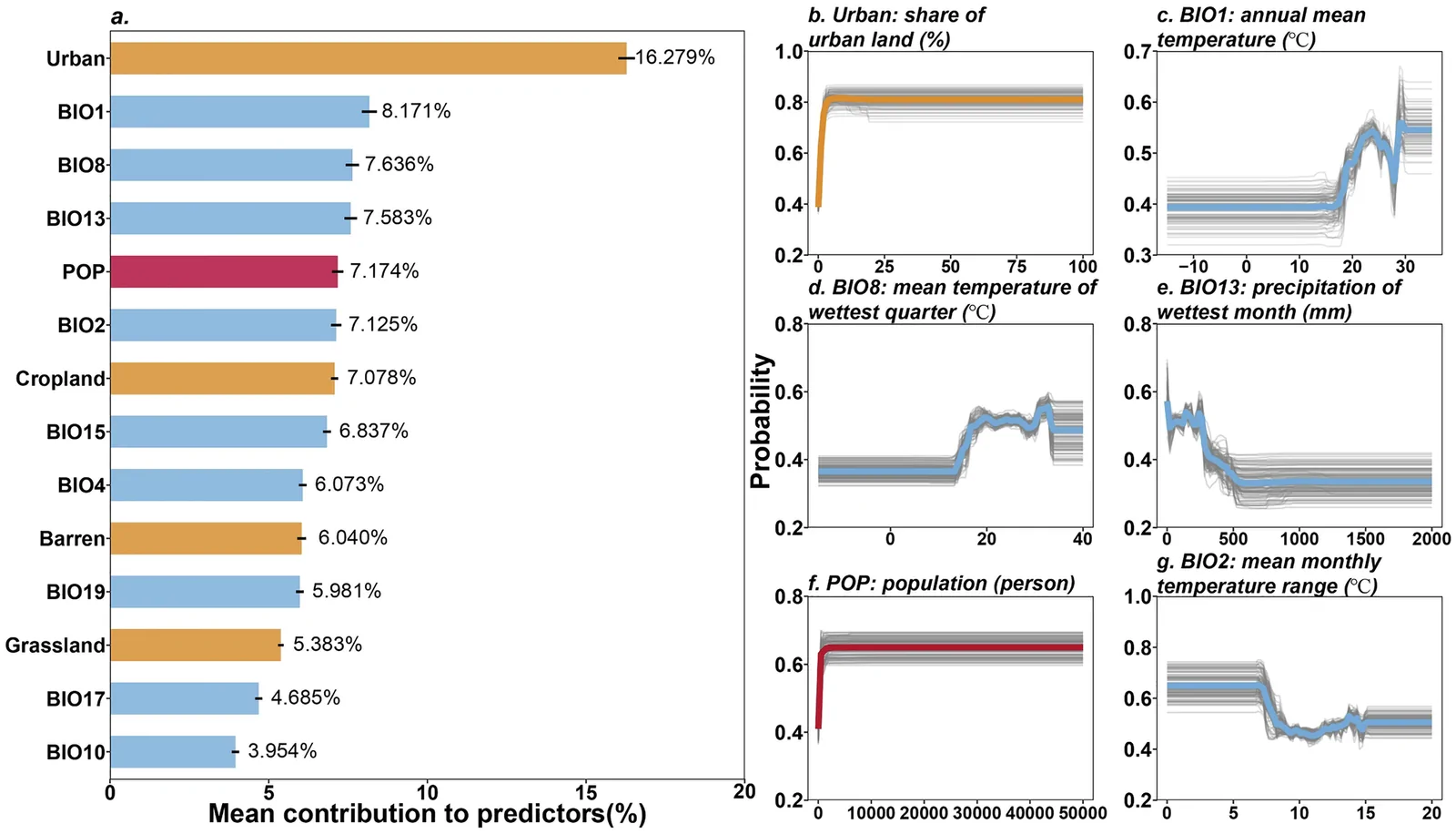
The impact of factors on habitat suitability of *An. stephensi.* **a** the mean contribution of factors to the habitat suitability. The factors included in the BRT models were selected based on the Spearman’s correlation (Fig. S1). **b**–**g** the panel of the response curves of habitat suitability to the top six factors. The response curves of habitat suitability to other factors are shown in Fig. S2. The solid lines are the average of 100 repetitions, while the gray lines are the results of individual runs. The details about the other factors are illustrated in Supplementary Table S1. The AUC, sensitivity and specificity were used to evaluate the performance of the BRT model. Bars represent the 95% confidence interval across 100 iterations. The total number of samples combined with occurrence and background points is 1264, randomly selecting 80% of the data for modeling and the remaining 20% for validation. BIO1: annual mean temperature. BIO2: mean monthly temperature range. BIO4: temperature seasonality. BIO8: mean temperature of wettest quarter. BIO10: mean temperature of coldest quarter. BIO13: precipitation of wettest month. BIO15: precipitation seasonality. BIO17: precipitation of driest quarter. BIO19: precipitation of coldest quarter. Barren: share of barren. Cropland: share of cropland. Grassland: share of grassland. Urban: share of urban land. POP: population.

Urbanization indicators, including share of urban land (Urban) and population (POP), contributed the most to the predicted probability of *An. stephensi* occurrence, with importance of approximately 16.279% (95% CI: 16.015–16.542%) and 7.174% (95% CI: 6.996–7.352%), respectively (Fig. 1a). Habitat suitability increased with increasing Urban, and when Urban reached approximately 26.263%, the habitat suitability plateaued (Fig. 1b). Furthermore, the response curve of the habitat suitability to POP was similar with that to Urban (Fig. 1b), and population growth would not increase habitat suitability when POP reached approximately 16667 (Fig. 1f).

Climatic variables also significantly affected the habitat suitability of *An. stephensi* (Fig. 1). For temperature indexes, the mean contribution of annual mean temperature (BIO1) to habitat suitability was 8.171% (95% CI: 7.935–8.407%). Mean monthly temperature range (BIO2) and mean temperature of wettest quarter (BIO8) were also critical for *An. stephensi*, with the importance of 7.125% (95% CI: 6.962–7.288%) and 7.636% (95% CI: 7.425–7.848%), respectively. For precipitation indexes, the mean contribution of precipitation of wettest month (BIO13) to the habitat suitability was approximately 7.583% (95% CI: 7.387–7.780%). Other climatic variables are less essential to the habitat suitability.

Other land use types had relatively low influence on the habitat suitability of *An. stephensi* compared with Urban. Share of cropland (Cropland) was less important to the habitat suitability of *An. stephensi*, with the mean contribution of approximately 7.078% (95% CI: 6.964–7.192%). The importance of share of barren (Barren) and grassland (Grassland) to the habitat suitability of *An. stephensi* was relatively low (Fig. 1a), with the mean contribution of approximately 6.040% (95% CI: 5.901–6.179%) and 5.383% (95% CI: 5.294–5.473%%), respectively.

### The impact of urbanization on habitat suitability

**Fig. 2:**
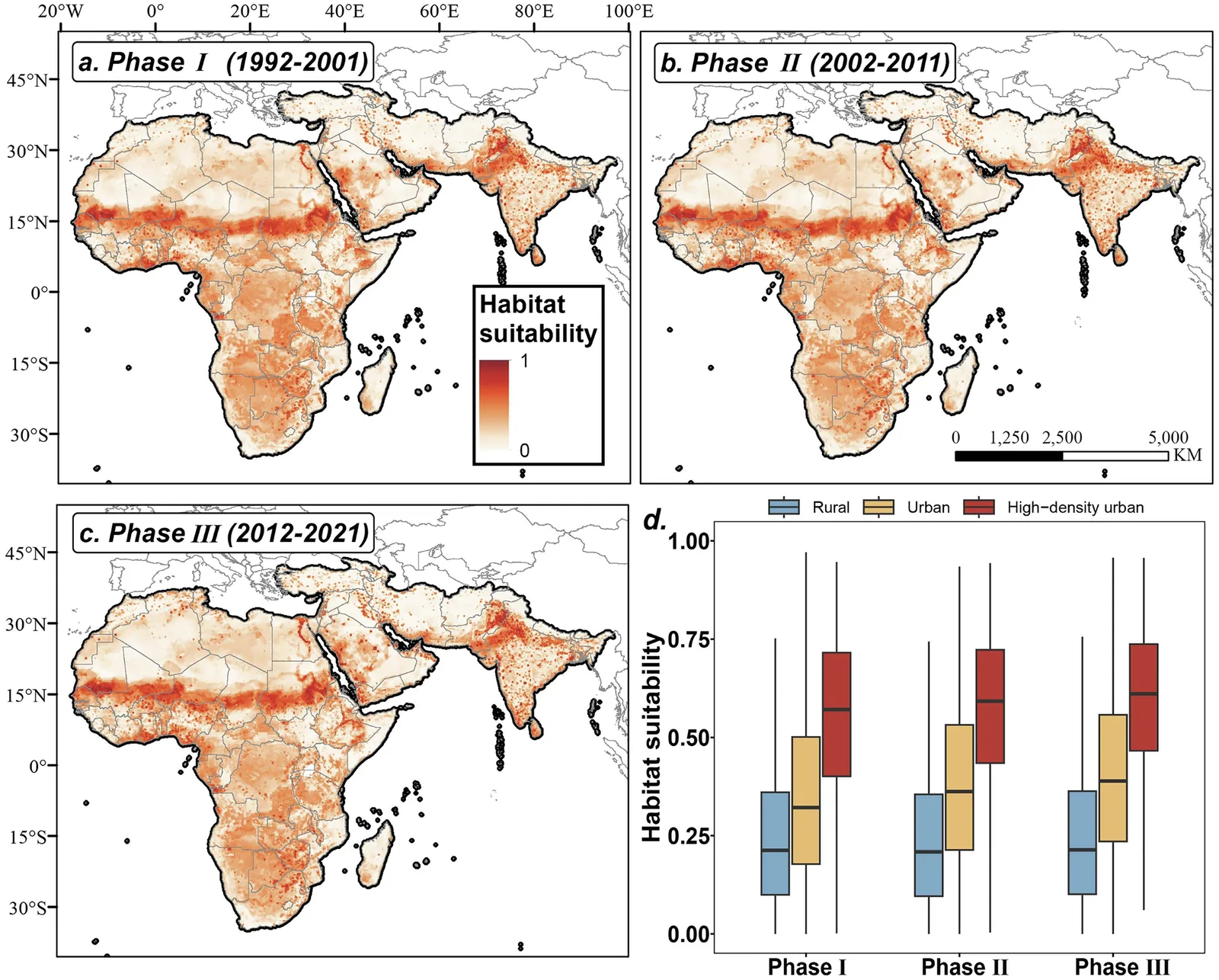
The predicted habitat suitability of *An. stephensi* over three phases. **a**–**c** the mean estimated spatial distribution of the habitat suitability from the BRT models across 100 iterations in the three phases. The dark colors represent high habitat suitability and the light colors represent low habitat suitability. These maps represent environmental suitability. Since the *An. stephensi* has not yet reached equilibrium, our results included some extrapolation. Therefore, we used the coefficient of variation estimated per pixel across 100 simulations of the BRT model in the three phases (Fig. S3) to indicate the stability of the predicted results. **d** the boxplots represent habitat suitability of rural, urban and high-density urban regions according to the classification in the study (*N* = 2,046,839 grid cells in Phase Ⅰ, *N* = 2,046,906 grid cells in Phase Ⅱ and *N* = 2,046,964 grid cells in Phase Ⅲ). The predicted values are scaled from 0 to 1 through a min-max transformation.

To further analyze the impact of urbanization on *An. stephensi*, we reconstructed the habitat suitability of *An. stephensi* in three phases based on the BRT models (Fig. 2). Predicted distributions of *An. stephensi* are shown in Fig. 2a–c. India, the Arabian Peninsula and central and southern parts of Africa were identified as the suitable habitat of *An. stephensi* (Fig. 2a–c). We estimated the habitat suitability in rural, urban and high-density urban regions (Fig. 2d), and found that the average habitat suitability in high-density urban regions was the highest in three phases (Phase Ⅰ: 0.548 [95% CI: 0.543–0.553]; Phase Ⅱ: 0.566 [95% CI: 0.561–0.570]; Phase Ⅲ: 0.585 [95% CI: 0.582–0.589]), with rural areas having the lowest suitability (Fig. 2d). Consistent with the species’ known urban affinity, our quantitative results showed that habitat suitability was approximately twice as high in high-density urban areas as in rural areas.

**Fig. 3:**
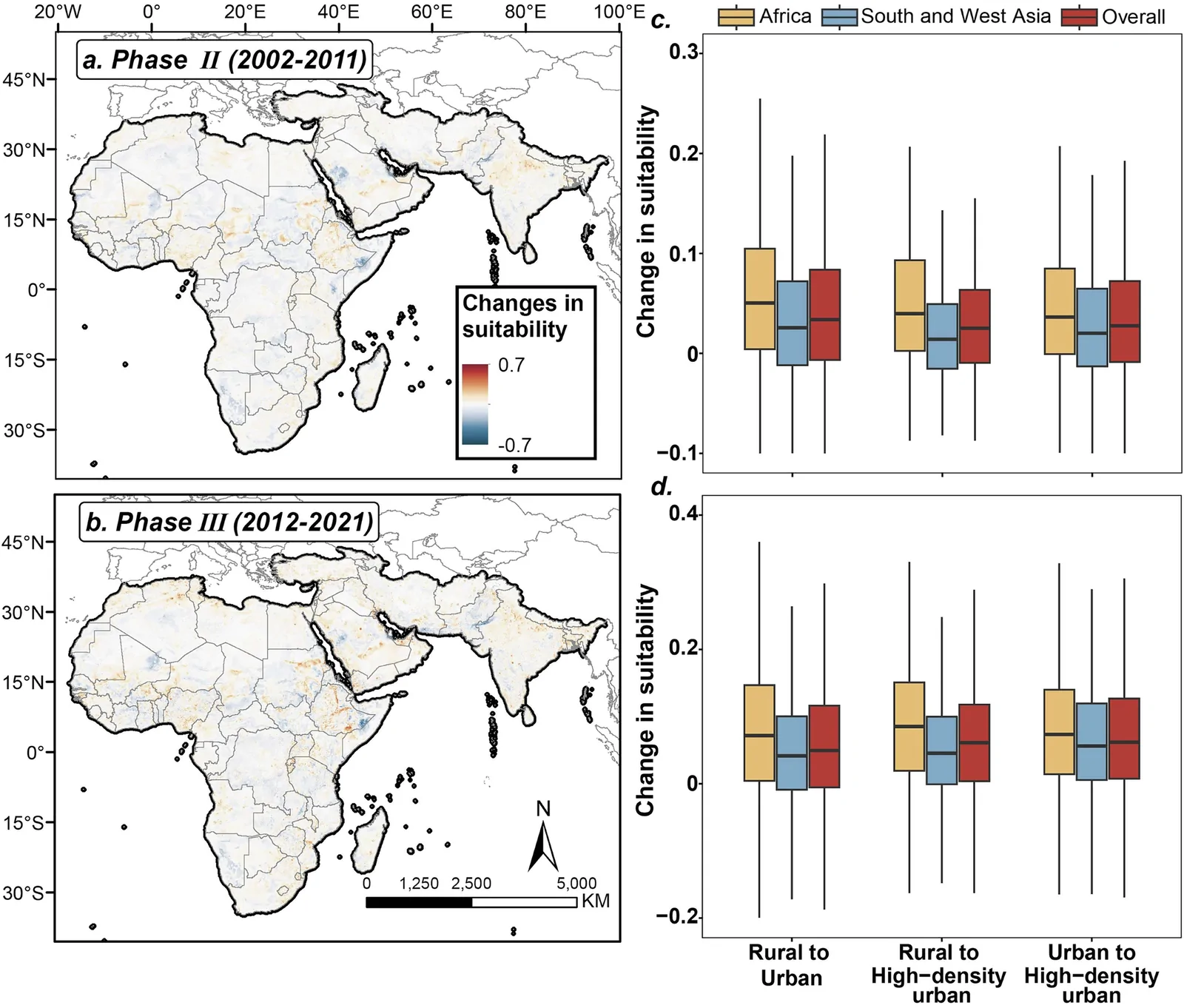
The changes in predicted habitat suitability of *An. stephensi* in Phase Ⅱ and Ⅲ. **a**, **b** the spatial distribution of changes in habitat suitability in Phase Ⅱ and Phase Ⅲ, respectively. **c**, **d** the boxplots of changes in habitat suitability in Africa, Asia and the whole study area in Phase Ⅱ (*N* = 13,971) and Phase Ⅲ (*N* = 33,473), respectively. The baseline was Phase Ⅰ. The differences in the habitat suitability between other two phases and baseline were the changes in habitat suitability in Phase Ⅱ and Phase Ⅲ, respectively.

Our reconstruction showed that the habitat suitability of *An. stephensi* was gradually increased in northern and central Africa, particularly during Phase Ⅲ (Fig. 3a, b). Meanwhile, habitat suitability also increased in India and the Arabian Peninsula, especially in Phase Ⅲ (Fig. 3a, b). Most land-use changes occurred during Phase Ⅱ and Phase Ⅲ (Fig. S4), and the number of grid cells that changed from rural to urban was the largest, with 84.396% and 86.780% of the total changing grids in Phase Ⅱ and Phase Ⅲ, respectively. Additionally, the number of rural-to-urban grids in Phase Ⅲ was approximately 2.464 times higher than that in Phase Ⅱ, suggesting that urban areas were expanding rapidly in Phase Ⅲ. Even though there were fewer high-density urban regions in Phase Ⅲ, many areas had undergone rapid urbanization in Africa (Fig. S4). This extensive urbanization might lead to a further increase in habitat suitability of *An. stephensi*. Figure 3c, d show the changes in habitat suitability in different types of urbanization transitions between rural, urban and high-density urban in Africa, South and West Asia and the whole study area. Among the three transformation types, the changes in habitat suitability in Phase Ⅱ were similar, with 0.039 (95% CI: 0.038–0.040) in rural-to-urban regions, 0.027 (95% CI: 0.018–0.036) in rural-to-high-density-urban and 0.032 (95% CI: 0.029–0.035) in urban-to-high-density-urban regions. Compared with Phase Ⅱ, the habitat suitability increased more in Phase Ⅲ, with the most substantial growth occurring in the transition from urban to high-density urban (0.070 [95% CI: 0.067–0.073]). This further analysis suggested that urbanization might be the main reason for the increased habitat suitability of *An. stephensi*.

The habitat suitability of *An. stephensi* in Africa has increased the most, compared with South and West Asia, and the whole study area (Fig. 3c, d). In Phase Ⅱ, the habitat suitability in Africa increased only slightly across different types of transmission in rural, urban and high-density urban, with the average changes of 0.056 (95% CI: 0.054–0.059) in rural-to-urban regions, 0.045 (95% CI: 0.029–0.060) in rural-to-high-density-urban regions and 0.041 (95% CI: 0.036–0.047) in urban-to-high-density-urban regions. In Phase Ⅲ, the habitat suitability increased significantly (Fig. 3d). The regions that transformed from rural to high-density urban had the largest increase, with the average changes of 0.089 (95% CI: 0.078–0.100). Meanwhile, many areas in Africa were transformed from rural to urban, with approximately 84.372% and 86.557% of all transformation types in Phase Ⅱ and Phase Ⅲ, respectively (Fig. S4). It indicated that urbanization may exacerbate the changes in habitat suitability of *An. stephensi* in Africa. Numerous rural areas in South and West Asia have also been converted to urban areas (Fig. S4), and the habitat suitability in these locations has increased (Fig. 3c, d). In Phase Ⅱ, the changes in habitat suitability in three types of transition between rural, urban and high-density urban were similar in South and West Asia. In Phase Ⅲ, the regions that transitioned from urban to high-density urban showed the largest increase in habitat suitability, with 0.065 (95% CI: 0.062–0.069).

We further analyzed the effect of single factors on the habitat suitability of *An. stephensi* by controlling other variables. Fig. S5a, b show the spatial distribution of habitat suitability when only urban land was considered. We found that urban land drove an increase in the habitat suitability in Central Africa, South Asia and northern West Asia, especially in Phase Ⅲ (Fig. S5a, b). Population drove an increase in the habitat suitability in Central and Southern Africa and South Asia, with increases in some area aligning with urban land (Fig. S5c, d). Climate and other land use types (except urban land) have also led to changes in habitat suitability (Fig. S6). Climatic factors exhibited spatial variability in its impact on the habitat suitability, resulting in increased habitat suitability in Central Africa and the Arabian Peninsula while decreasing habitat suitability in parts of South Asia (Fig. S6a, b). Other land-use types (except urban land) increased habitat suitability in most part of the study area (Fig. S6c, d).

**Fig. 4:**
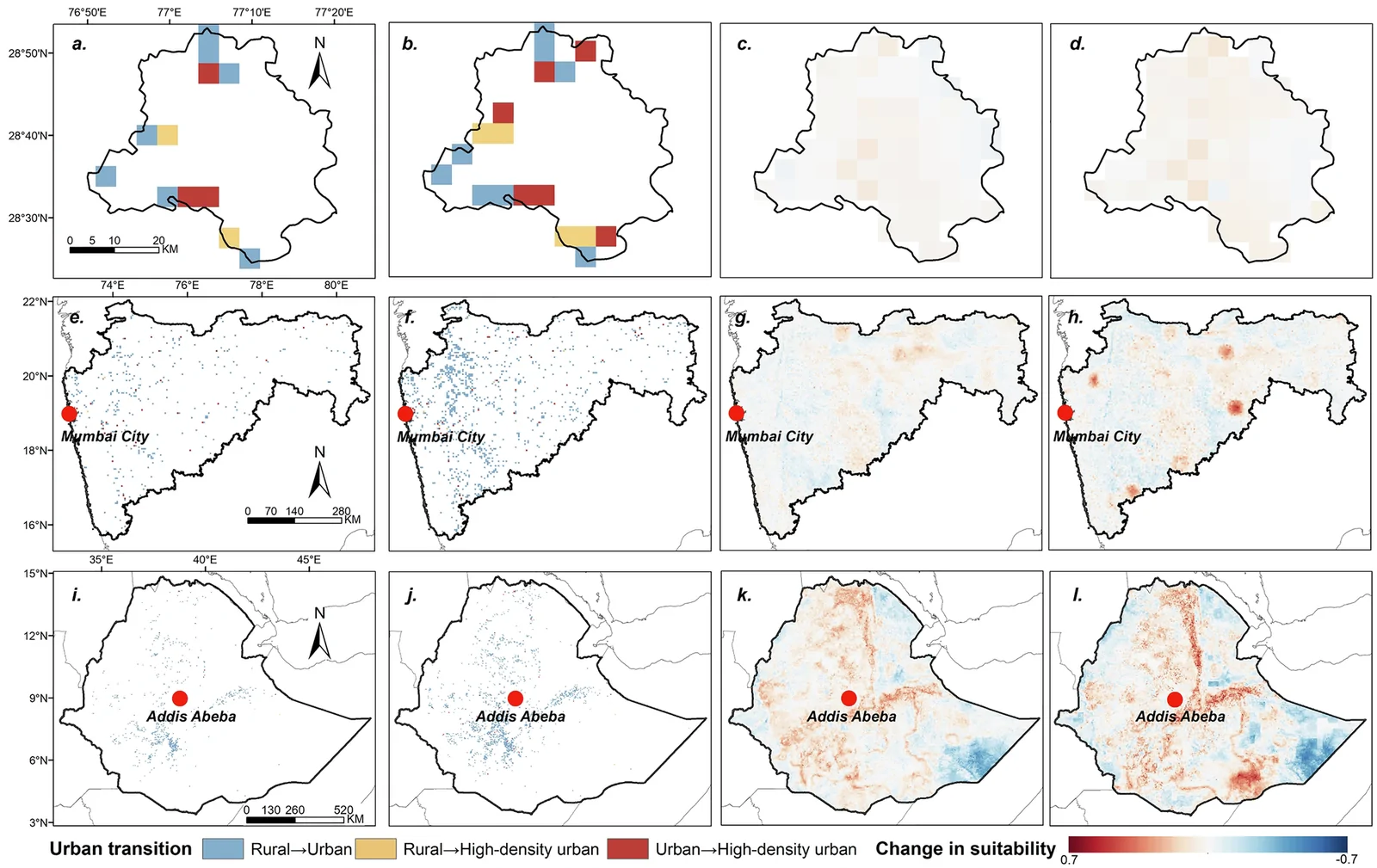
The changes in predicted habitat suitability of *An. stephensi* in the small scale. **a**, **b**, **e**, **f**, **i**, **j** the changes in rural, urban and high-density urban areas in Phase Ⅱ and Phase Ⅲ in New Delhi, India, Maharashtra State, India and Ethiopia, respectively. **c**, **d**, **g**, **h**, **k**, **l** the changes in habitat suitability of *An. stephensi* in Phase Ⅱ and Phase Ⅲ in New Delhi, India, Maharashtra State, India and Ethiopia, respectively. The baseline was Phase Ⅰ.

The results in a smaller scale further indicated that urbanization increased the habitat suitability of *An. stephensi* (Figs. S7, S8 and Fig. 4). We selected small-scale regions (New Delhi [India], Maharashtra State [India], and Ethiopia) to further demonstrate the impact of urbanization on habitat suitability of *An. stephensi*. Fig. S7 shows the distribution of rural, urban and high-density urban areas in the three regions or countries. We found that high-density urban areas were always surrounded by urban areas. We also found that most urban areas were highly suitable for *An. stephensi* (Fig. S8). Furthermore, we analyzed the changes in the habitat suitability during the process of urbanization (Fig. 4). For changes in urban areas, New Delhi had the smallest change, while urban areas in Maharashtra State and Ethiopia expanded obviously (Fig. 4a, b, e, f, i, j). The changes in habitat suitability were smallest in New Delhi, India (Fig. 4c, d). Many areas in Maharashtra State, India in Phase Ⅲ have gradually developed into urban or high-density urban areas (Fig. 4f). Distribution of habitat suitability changes was spotty in Maharashtra State, India, with areas of high increase mostly coinciding with areas of urban change, and larger changes occurred in Phase Ⅲ than Phase Ⅱ (Fig. 4g, h). These changes were even more apparent in Ethiopia (Fig. 4i–l). Ethiopia has undergone rapid urbanization, with a large number of urban areas emerging during Phase Ⅱ and Phase Ⅲ (Fig. 4j). The distribution of high habitat suitability aligned with newly emerging urban areas, and the increase was larger in Phase Ⅲ than Phase Ⅱ (Fig. 4k, l). The results in small scale illustrate that urbanization is a key factor contributing to increased habitat suitability of *An. stephensi*.

### Mechanisms of urbanization on habitat suitability

**Fig. 5:**
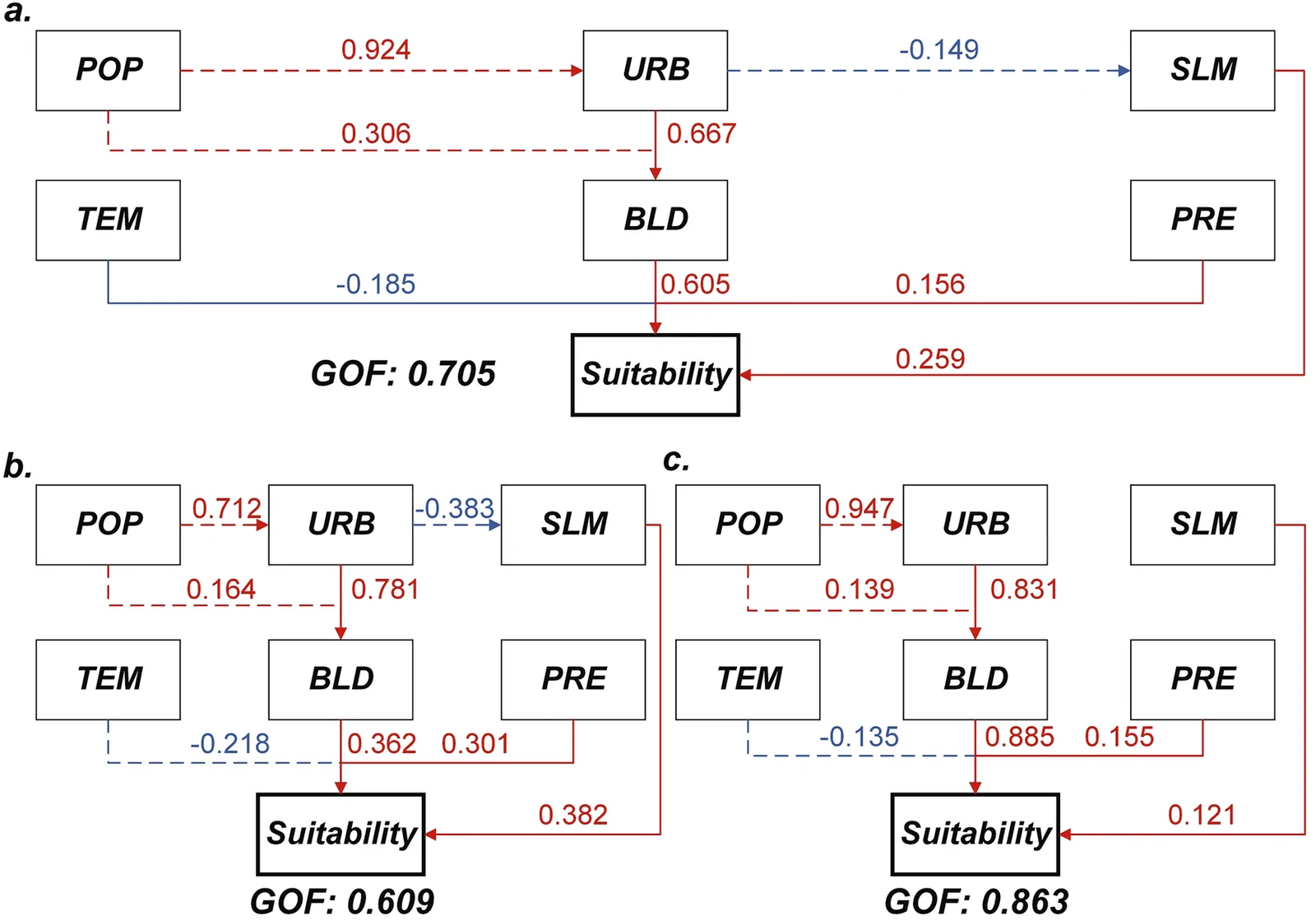
The mechanisms of factors on habitat suitability of *An. stephensi.* **a** the results of the PLS-PM model including all data in the study area (*N* = 246). **b**, **c** the results of the model only including data in Africa (*N* = 168), and South and West Asia (*N* = 78), respectively. The blue line is the negative relationship between factors and habitat suitability and the red line is the positive relationship between factors and habitat suitability. The solid line represents the direct influence of factors on habitat suitability and the dash line represents the indirect effect of factors on habitat suitability. Path significance is assessed using 1000 bootstrap resamples with two-sided tests and the path coefficient in the panel is significant (*p* < 0.05). Each box represents a latent variable, and the specific explicit variables included in each of the three models are shown in Table S3. GOF: goodness-of-fit. POP: population. URB: share of urban land. SLM: people living in slums in cities. BLD: construction-related indexes. TEM: temperature indexes. PRE: precipitation indexes. Suitability: the habitat suitability of *An. stephensi*.

Share of urban land (URB) and population (POP) indirectly affected habitat suitability mainly through construction-related indexes (BLD) (Fig. 5a). Specifically, URB affected the habitat suitability of *An. stephensi* mainly through construction-related indexes (BLD), with the path coefficient of approximately 0.404 (*p* < 0.05), which was the primary pathway affecting the habitat suitability. POP also indirectly affected habitat suitability through BLD, with the path coefficient of approximately 0.185 (*p* < 0.05). In addition, POP also affected the habitat suitability through URB and the path “POP-URB-BLD-Suitability” was approximately 0.373 (*p* < 0.05). Furthermore, the increase in people living in slums in cities (SLM) could increase the habitat suitability. However, an increase in URB resulted in a decline in SLM, thus reducing the habitat suitability through the path “URB-SLM-Suitability”, with the path coefficient of approximately −0.039 (*p* < 0.05). This path coefficient was offset by the path “URB-BLD-Suitability”, and URB could increase the habitat suitability of *An. stephensi*, with the total path coefficient of approximately 0.365 (*p* < 0.05).

The mechanism that urbanization affected the habitat suitability of *An. stephensi* in Africa had some differences from that in the overall study area (Fig. 5b). The primary path that urbanization affected the habitat suitability was “URB-BLD-Suitability”, with the path coefficient of approximately 0.283 (*p* < 0.05). URB could also affect SLM and further affect the habitat suitability, which was similar with the results in the overall study area. In addition, POP indirectly affected the habitat suitability through BLD, with the path coefficient of approximately 0.059 (*p* < 0.05). POP also affected URB and thereby influenced the habitat suitability, with the path coefficient of approximately 0.201 (*p* < 0.05).

Urbanization affected the habitat suitability in South and West Asia mainly through BLD (Fig. 5c). “URB-BLD-Suitability” was the primary path that urbanization affected the habitat suitability, with the path coefficient of 0.735 (*p* < 0.05). POP also affected the habitat suitability mainly through BLD, with the path coefficient of 0.123 (*p* < 0.05). However, URB could not increase SLM and further increase Suitability (*p* > 0.05), which was different from the results in the overall study area and Africa.

## Discussion

As a critical vector of malaria, the spatial distribution of *An. stephensi* has been affected by human activities and changed dramatically over the past 30 years. In the study, we analyzed the key elements affecting the habitat suitability by using mathematical models, and found that urbanization indicators, including share of urban land and population, were critical for *An. stephensi* habitat suitability. The spatial distribution of the habitat suitability showed that the increased habitat suitability was associated with urbanization, especially in Africa. Furthermore, we analyzed the mechanisms by which urbanization affected the habitat suitability. It revealed that urbanization indirectly affected the habitat suitability mainly through construction-related activities. The study quantitatively analyzed the mechanisms of urbanization on the habitat suitability of *An. stephensi*, which can provide key parameters for predicting malaria risks in the future and help to design target vector control strategies to eliminate *An. stephensi*.

Share of urban land and population within urbanization indicators are key factors influencing the habitat suitability of *An. stephensi* (Fig. 1). We selected two metrics to represent urbanization based on the previous study, including share of urban land and population^20^. Furthermore, another previous study also indicated that population played an important role in the potential distribution of *An. stephensi*^5^, which is similar to our results. Meanwhile, previous studies that predicted the potential distribution of *An. stephensi* have only considered climatic factors^18,21^. The study demonstrated that urbanization indicators, including share of urban land and population, could be included in the future studies to predict the potential distribution of *An. stephensi* more accurately. On the other hand, since BRT is a species distribution modeling (SDM) used to analyze species fully occupying the niche, and *An. stephensi* is an invasive species, we also constructed a BRT model only in Africa. Figure S9 shows that share of urban land and population also significantly affected the habitat suitability of *An. stephensi*, consistent with the overall finding, which demonstrated the robustness of the results of the BRT model in the study. However, some factors’ (i.e., share of grassland) contribution to the habitat suitability was different from the overall findings, suggesting that the ecological characteristics of *An. stephensi* might undergo changes during its invasion process. Future research is needed to explore the habitat characteristics of *An. stephensi* in Africa.

Our model suggested that urbanization accelerated the changes in the habitat suitability of *An. stephensi*. The study moved beyond treating urbanization as a simple categorical variable and instead quantified how habitat suitability changed across different stages of urban development, revealing non-linear transitions from rural to high-density urban environments. The habitat suitability of *An. stephensi* increased in Africa during Phase Ⅲ (Fig. 3), and its invasion into Africa since 2012^2^ also illustrated it. Urbanization progressed rapidly in Phase Ⅲ, and the number of becoming urban or high-density urban grids reached 2.464 times that of Phase Ⅱ (Fig. S4), and the urban-to-high-density-urban regions had the largest increase in the habitat suitability (Fig. 3). Despite the reliability of the urban classification, the delineation in the boundaries between urban and rural areas is hard. Because the population in these areas may be close to the urban threshold, and some environments may be quite similar to the habitats of *An. stephensi* in the urban areas. Future field research is needed to conduct in-depth analysis of *An. stephensi* larval habitats in these areas. However, the results indicate that *An. stephensi* is more likely to be found in urban environments and did not suggest that *An. stephensi* was only found in urban areas, which depends on the forms of *An. stephensi*. The type and intermediate forms of *An. stephensi* are also efficient vectors in rural environment^7^, such as freshwater ponds, stream beds, wells and seepage canals^22^. On the other hand, it is certain that multiple factors, including climate, urban land and population, have influenced the distribution of *An. stephensi*. For example, the habitat suitability in southern Ethiopia has increased, while there was a little urban area. This might be the result of climate effect, as the habitat suitability increased in this region under climate effects alone (Fig. S6). Our study suggested that urbanization and climate change in the future might have a substantial impact on the distribution of *An. stephensi*, potentially enabling its invasion into other continents or countries where climatic conditions were suitable and rural and urban larval habitat existed. Therefore, the adaptation to both urban and rural environments of *An. stephensi* needs to implement updated mosquito control methods to eliminate it.

Urbanization has created more larval sites for *An. stephensi*. Man-made conditions are suitable for *An. stephensi* larvae, such as overhead tank^22^, artificial water containers, construction pits^10^, condensation from air conditioning, open oil drums and water-filled tires after a rainfall^16^. The study further quantitatively revealed the mechanisms of urbanization on the habitat suitability of *An. stephensi* (Fig. 5). We used infrastructure investment and built-up areas to represent the construction-related activities and found urbanization affected the habitat suitability mainly through construction-related activities. Construction booms appear to create more larval sites for *An. stephensi*^23^. The expansion of urban areas and the growth in population resulted in the need for more buildings, and it then increased the habitat suitability of *An. stephensi*. Artificial water containers prevalent in construction sites, including concrete water tanks and car washing facilities, have become larval habitats for *An. stephensi*^16^. Although the study did not have construction sites data, infrastructure investment and built-up areas were closely associated with construction sites because all construction projects required construction sites during development. Therefore, it revealed that increased construction due to urban expansion and population growth added more artificial water -storage containers, which might create new larval sites for *An. stephensi*.

The proportion of people living in slums in cities would increase the habitat suitability of *An. stephensi*. The study also indicated that *An. stephensi* appeared in urban slums in Chennai, India^24^. However, we found that an increase in urban land would reduce the proportion of people living in slums in cities, thereby decreasing the habitat suitability through this pathway in the overall study area (Fig. 5a). From this perspective, urbanization may reduce the habitat suitability of *An. stephensi*. Due to the relatively minor path effect being offset by the paths through construction-related activities, overall urbanization still increased the habitat suitability. Compared with previous field surveys^16,22^, our study incorporated quantitative analysis and revealed the mechanisms by which urbanization affected the habitat suitability on a large scale, which bridges the gap between large-scale modeling and known ecological mechanisms. This enables the identification of broad patterns and high-risk regions that cannot be captured by local surveys alone and can provide scientific reference for better predicting the distribution of *An. stephensi* to design strategies to respond to malaria transmission in the future.

Africa is one of the regions with the fastest urbanization globally^25^, and is facing quick population growth in the 21st century, much of which will be absorbed by urban areas, with the total urban population projected to triple from current levels^26^. Malaria in Africa has been commonly considered a rural transmission disease^27^. However, we found that urbanization increased the habitat suitability in Africa (Fig. 3d and Fig. 4i–l), which might pose a malaria risks to African cities. The previous study also indicated that the African cities face challenges to malaria control, mainly due to unplanned urbanization and transmission of *An. stephensi*^28^. In fact, there have been multiple malaria outbreaks caused by the invasion of *An. stephensi* in Africa^29,30^. For example, spatial aggregation of *Plasmodium falciparum* infection around malaria patients in Ethiopia was associated with *An. stephensi* in the vicinity of households under a prospective case-control design experiment^29^. Furthermore, urbanization also affected the habitat suitability was mainly through construction-related activities (Fig. 5b). The study in Ethiopia also demonstrated that mass urban migration and construction booms across African cities were likely facilitating the expansion of *An. stephensi*, creating new larval habitats^31^. Although the differences in forms and behaviors of *An. stephensi* between Africa, and South and West Asia are not included in the study, it is certain that *An. stephensi*, which adapt to both rural and urban environments, may add a new threat to malaria transmission in Africa. It needs to apply targeted mosquito control technologies and strengthen surveillance to address malaria transmission in Africa.

*An. stephensi* was the malaria vector mainly in South Asia and the Arabian Peninsula before 2011^2^. Many studies in Asia confirmed that *An. stephensi* was transmitted malaria mainly in cities^32,33^, for example, the study indicated that *An. stephensi* was a major urban malaria vector in India^33^. Our study also indicated that the habitat suitability was high in urban and high-density urban areas in South and West Asia (Fig. 2). The changes in habitat suitability in South Asia and West Asia were relatively lower than that in Africa, and even decreased in some areas (Fig. 3). Single-factor analysis indicated that this decline in some areas might be climate-driven. For instance, the reduction in the habitat suitability in southeastern India coincided with a climate-driven decrease in habitat suitability (Fig. 3 and Fig. S6). Furthermore, urbanization also primarily affected the habitat suitability through construction-related activities. However, urbanization could increase the proportion of people living in slums in cites, which differed from the results in the overall study area and Africa (Fig. 5c). Urbanization in Asia was progressing faster than in Africa^34^, which may lead to regional development imbalances, resulting in an increase in slums. Therefore, urban planning should be prioritized during urbanization to reduce *An. stephensi* larval sites.

There are three limitations in the study. First, it is recognized that a perfect and unbiased sampling regime is challenging. While our methodological approach aims to mitigate this known data bias, some inherent biases associated with the original occurrence data may persist. Future study incorporating systematic field surveillance across diverse environments would be critical. Second, due to differences in data precision, we used resampling to standardize the data to a resolution of 2.5 min. This could potentially lead to a loss of information in the precise data. Meanwhile, due to the limited temporal updates of data, we adopted multiple approaches to unify the time scale, which may also have a slight impact on the results. Finally, the differences in *An. stephensi* behaviors between Africa, South Asia and West Asia were not included in the study. In-depth research is needed to analyze it in the future.

## Methods

**Fig. 6:**
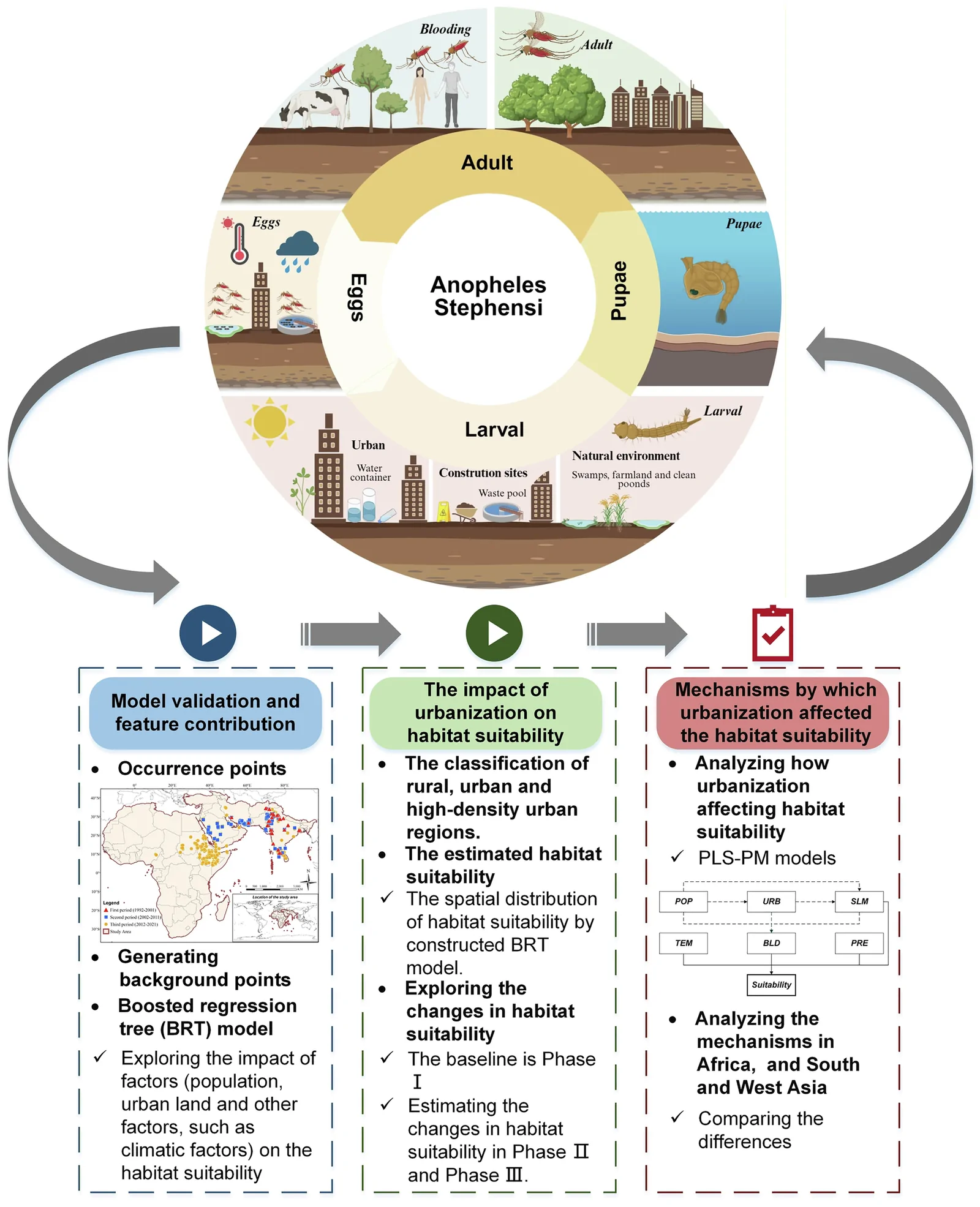
The framework in the study. The circle represents elements that may affect the four stages (eggs, larval, pupae and adult) of *An. stephensi, created in BioRender. Sun, Y. (2026)* https://BioRender.com/y91r407. POP: population. URB: share of urban land. SLM: people living in slums in cities. BLD: construction-related indexes. TEM: temperature indexes. PRE: precipitation indexes. Suitability: the habitat suitability of *An. stephensi*.

The study was conducted to analyze the impacts and mechanisms of urbanization on the habitat suitability of *An. stephensi* in West Asia, South Asia and Africa during 1992–2021. We divided the period into three phases based on the invasion of *An. stephensi* and the temporal structure of key spatial datasets: 1992–2001 (Phase Ⅰ), 2002–2011 (Phase Ⅱ) and 2012–2021 (Phase Ⅲ) (please see Supplementary Method 1 for details). We firstly generated background points using an environmental background-based technique and used kernel density estimation (KDE) to reflect the bias seen in the occurrence points. Then, we constructed BRT models to analyze the key factors affecting the distribution of *An. stephensi* and estimate the changes in habitat suitability under different levels of urbanization in the three phases. Finally, we conducted PLS-PM models to investigate the mechanisms driving the changes in habitat suitability of *An. stephensi*. The research framework is shown in Fig. 6.

### Occurrence and background points with bias correction

We obtained occurrence global records of *An. stephensi* from 1992 to 2021 through GBIF^35^, WHO^36^ (some data was from Sinka and colleagues^5^), and other literatures^15,37–41^. For data from GBIF^35^, WHO^36^ (some data was from Sinka and colleagues^5^), we only retain occurrence points with complete records containing date, latitude and longitude information. We examined these coordinates and excluded points in obviously incorrect locations, such as locating in the ocean or where the latitude and longitude coordinates did not align with national administrative regions. The numbers of data from GBIF^35^, WHO^36^ (some data was from Sinka and colleagues^5^) are 670 and 844, respectively. We also searched for literatures on *An. stephensi* using the keyword “*Anopheles stephensi*”, “*An. stephensi*”, “*An. stephensi suitability habitat*”, “*An. stephensi habitat*”, “*Anopheles stephensi suitability habitat*” and “*Anopheles stephensi habitat*” in Web of Science, PubMed and Google Scholar, and six relevant literatures^15,37–41^ were found, which recorded the complete locations and dates of *An. stephensi*. We then collected a total of 202 records from the literatures. Finally, we merged the data from these three sources, removed duplicate, and obtained a total of 632 occurrence points in the study region (54 in Phase Ⅰ, 132 in Phase Ⅱ and 446 in Phase Ⅲ). The distribution of occurrence points is shown in Fig. S10.

Due to the lack of true species absence data, we considered survey accessibility and environmental filtering to generate backgrounds points based on the previous studies^42,43^. To account for spatial sampling bias, for example, the sampling may be tended to target densely populated areas or places accessible to researchers, we assigned probability weights to each grid cell using KDE to establish a sampling similar with the occurrence points. The results of KDE are shown in Fig. S11. The KDE surface represents the relative probability that a location has been sampled. Background points were then sampled proportionally to this surface, ensuring that the background points reflect the same spatial sampling bias as the occurrence points. Then, to reduce environmental overlap between occurrence and background points, we applied an environmental background based technique^43^. A principal component analysis (PCA) was used to obtain a two-dimensional environmental background, and we conducted a restricted convex hull, which the environment outside the restricted convex hull was considered to the unfavorable environment for *An. stephensi*. Combined with the probability on the grid cells from KDE, background points were randomly generated outside this convex hull with the same number of the occurrence points in three phases. To improve the robustness of the results, we simulated 10 different sets of background points.

### Environmental and human activities data processing

The environmental factors associated with *An. stephensi* were found from the literatures, for example, the monthly densities of *An. stephensi* adults and larvae were associated with precipitation, maximum and mean temperature^44^. We then summarized the elements affecting the four stages of *An. stephensi* (Fig. 6), from which we obtained the environmental and human activities factors that may affect *An. stephensi*.

We obtained monthly total precipitation, monthly maximum temperature and monthly minimum temperature from the WorldClim in 1992–2021^45^. We used the “biovars” function in the R package “dismo”^46^ to estimate 19 bioclimatic variables for 1992–2021 using the monthly total precipitation, monthly maximum temperature and monthly minimum temperature. The spatial resolution is 2.5 min.

The land use dataset was collected from Land-Use Harmonization (LUH2)^47^ and the spatial resolution was 0.25^°^, which is the proportion of the land use pattern at the grid scale. The land use patterns of LUH2 are more finely categorized, containing 14 land use patterns, such as forested primary land and urban land, and we have reclassified the land use patterns into five types based on the previous study^48^, including barren, cropland, forest, grassland and urban land. The details about the reclassification of the land use are shown in Table S2. To eliminate interference, we finally included barren, cropland, grassland and urban land into the study, and removed forest areas because the share of forest areas in occurrence points was similar. Since year-to-year changes in land use data were not obvious, we used the data of the first year of each phase to represent the land use of the phase, i.e., the land use data in 1992 for the first phase, the land use data in 2002 for the second phase and the land use data in 2012 for the third phase.

Population data was obtained from LandScan^49^ and the spatial resolution is 30 arc-seconds. Since LandScan only provided data for 2000–2022, we assumed linear population growth to construct the linear algorithm to interpolate the data for 1992–1999 based on the previous study^42^. To facilitate calculations, we have resampled the data to a spatial resolution of 2.5 min. Elevation data was collected from WorldClim^45^ and the spatial resolution is 2.5 min. The build-up surface areas data was collected from European Commission^50^ with 1 km resolution, and updated every 5 years. We thus used the build-up surface areas data in 2000 for Phase Ⅰ, 2010 for Phase Ⅱ and 2020 for Phase Ⅲ. The infrastructure investment and people living in slums data were collected from World Bank Open Data on the national scale. The infrastructure investment data was the total private participation in infrastructure (PPI) investment in each country of the study area, and the people living in slums data was the proportion of people living in slums in cities. The details about all the data used in the study are shown in Table S1.

### Identifying rural, urban and high-density urban regions

We used the settlement typologies delineated and classified from European Commission^51^ to analyze the changes in the suitable habitat of *An. stephensi* during urbanization. The dataset defined the spatial entities at two hierarchical levels and the 1 km^2^ grids are divided into three categories of the first hierarchical level: rural, urban cluster and urban center grid cell (please see Supplementary Methods and Table S4 for details)^52^, and generated the settlement classification layers every 5 years since 1975^51^, which the resolution is 1 km. To standardize the resolution, we resampled the grids to achieve a 2.5-min resolution by using the nearest-neighbor method. Due to the several sub-levels of three categories in the data, we further consolidated it into three major categories: rural, urban and high-density urban for analysis. Furthermore, we selected the data in 2000 for Phase Ⅰ, the data in 2010 for Phase Ⅱ and the data in 2020 for Phase Ⅲ. The details about the classification methods and the approaches to verify the validation of this dataset are illustrated in the Supplementary Materials (Fig. S12). The grid-scale classification is shown in Fig. S13.

### Boosted regression trees

Boosted regression trees (BRT) is a statistical model for explaining and predicting nonlinear relationships between variables^53^. BRT models can handle different types of predictor variables and allow for missing data. BRT models can also fit complex nonlinear relationships and automatically calculate interactions between predictors without the need for a priori data transformation or outlier removal.

Before constructing the BRT model, we first examined multicollinearity among the 25 variables in 10 datasets. Initially, we assumed the data followed a normal distribution. The Shapiro-Wilk normality test revealed significance levels (*p* value) consistently below 0.001, indicating rejection of the null hypothesis. We then used Q-Q plots and found that the points were not uniformly distributed on either side of the reference line, further confirming the non-normal distribution (Fig. S14). Therefore, Spearman’s correlation was applied to assess multicollinearity among variables and the results are presented in Fig. S1. Variables with correlation coefficients exceeding 0.75 were excluded, leaving 14 variables for inclusion in the BRT model.

In the study, the “gbm” package in R version 4.4.1 and the BRT function^53^ were used to construct the effects of climatic and anthropogenic factors on the habitats of *An. stephensi* during 1992–2021. The data were temporally partitioned based on the three phases and then incorporated into the BRT model. We can obtain response curves of each variable to *An. stephensi* habitat in the BRT model, in which the horizontal coordinates represent the changes in the variables and the vertical coordinates represent the marginal effects of the variables on *An. stephensi* habitat suitability. In the BRT model, the nature of the error structure was set to “Gaussian”, the complexity of the tree was 10 and the learning rate was 0.001. To reduce the uncertainty, we repeated the process 10 times, randomly selecting 80% of the data for modeling and the remaining 20% for validation in each set of occurrence and background points. The study used area under curve (AUC), sensitivity and specificity to verify the accuracy of the BRT model.

To further analyze the spatial differences in the effects of the different factors on *An. stephensi* habitat, the study controlled the changes of individual factors by sensitivity tests to estimate the changes in the suitable habitat based on a previous study^54^. Specifically, we controlled for changes in only single factor while keeping all others constant to analyze the differences in the habitat suitability of *An. stephensi*. For example, we maintained constant population and land use parameters from Phase Ⅰ into the BRT models when projecting climate-driven changes in suitable habitats for *An. stephensi* during Phase Ⅱ and Phase Ⅲ by using the Phase Ⅰ as a baseline period. Meanwhile, we standardized habitat suitability values for each phase using the min-max transformation.

### Partial least-squares path (PLS-PM) model

The study used the PLS-PM model to analyze the mechanisms by which urbanization affected habitat suitability of *An. stephensi* on the national scale, and we also analyzed separately in the overall study area, Africa, West and South Asia. PLS-PM model can be applied to analyze complex relationships between modules that contains both latent and explicit variables^55^. Latent variables are theoretical concepts that cannot be directly measured, while explicit variables are concrete data indicators that can be directly observed or measured.

To analyze relationships at the national scale, we calculated the total habitat suitability index of *An. stephensi* (Suitability) in the study area. We also used the national total population (POP) and estimated the total urban area (URB) based on grid area in each country. Average temperature (TEM) and precipitation (PRE) indexes were included in the PLS-PM model as control variables. In addition, we calculated the total national value of built-up areas, and combined with infrastructure investment, which represents construction-related activities, formed the variable “construction-related indexes” (BLD).

The latent variables contained six factors, including population (POP), urban land (URB), construction-related indexes (BLD), people living in slums in cities (SLM), temperature indexes (TEM) and precipitation indexes (PRE). BIO1-BIO11 are the explicit variables of the latent temperature variable, and BIO12-BIO19 are the explicit variables of the latent precipitation variable. The absolute standardized loadings index was used to select the explicit variables that best reflect the latent variables (loadings > 0.7) and the final explicit variables contained within latent variables are shown in Table S3. The goodness-of-fit (GOF) can evaluate the merits of the model, and bootstrap resampling (for 1000 times) can explain the significance of each path of the model^55^. We considered all latent variables in the initial models but included the latent variables for which the path coefficient could pass through the significance test (*p* < 0.05). The PLS-PM was processed in R version 4.4.1 and the conceptual model is shown in Fig. S15.

## Supplementary information

Transparent Peer Review file

Supplementary information

## Data Availability

All data used in the study are from publicly available datasets. The occurrence data of *An. stephensi* is available from GBIF (https://doi.org/10.15468/dl.fh95wu), WHO (https://apps.who.int/malaria/maps/threats/#/download, some data are from Sinka et al. (https://doi.org/10.1073/pnas.2003976117)), and other literatures (https://doi.org/10.1186/s12936-023-04545-y, https://doi.org/10.1016/j.actatropica.2014.06.016, https://doi.org/10.3390/biology11030409, https://doi.org/10.1016/j.pt.2022.11.011, https://doi.org/10.1186/s12936-023-04656-6, and https://doi.org/10.1186/s12936-021-03801-3). The climate data and elevation data are from WorldClim (https://www.worldclim.org/data/monthlywth.html#google_vignette). The land use data is from LUH2 (https://luh.umd.edu/). The population data is from LandScan (https://landscan.ornl.gov). The settlements data is from European Commission (https://human-settlement.emergency.copernicus.eu/). The infrastructure investment and people living in slums data are from World Bank Open Data (https://data.worldbank.org/).

## Code availability

The R codes used to run the regression and generate tables and figures in the study are available at https://doi.org/10.6084/m9.figshare.32649039.
