# Supplementary material for: Urbanization development characteristics coincide with elevated Anopheles stephensi habitat suitability
Source: Commun Earth Environ. 2026 Jul 22;7(1):893. doi: 10.1038/s43247-026-03833-0 (PMC13759296; doi:10.1038/s43247-026-03833-0)
Supplement: Supplementary file 1 [file 43247_2026_3833_MOESM1_ESM.pdf]

nature portfolio

Peer Review File

Urbanization development characteristics coincide with elevated Anopheles stephensi habitat suitability

Corresponding Author: Professor Qiyong Liu

This file contains all reviewer reports in order by version, followed by all author rebuttals in order by version.

Attachments originally included by the reviewers as part of their assessment can be found at the end of this file.

Version 0:

Reviewer comments:

Reviewer #1

(Remarks to the Author)
Yelin Sun et al (review)

The manuscript reports a study looking to use BRT to establish the habitat preferences of An. stephensi.

While it is clear the authors have put in a lot of work, there are some significant issues with this work.

1. There does not appear to have been consideration of the limitations and problematic issues that have to be accounted for when used in data amalgamations like those from GBIF – specifically, data quality, data sampling, bias data. GBIF data is not very clean, nor has it undergone any quality checks. The data will be reporting where sampling of this species has occurred (i.e. the presence of an entomologist PLUS the mosquito) and so is hugely biased to areas that are accessible (close to research stations, for example). This is not a comprehensive sampling regime (which is what SDMs really require) but as unbiased data are rarely available, it is possible to address the bias in the modelling – that doesn't look like it has been done here.
2. It is not clear what novel ideas or findings are being reported – it shows the species may have a preference for urban areas (but in Africa, the sampling for this particular species has almost exclusively been in urban areas – it has now been shown to be present in rural areas in Ethiopia)
3. BRT is a SDM, and they have very specific assumptions, for example, whether the species is fully occupying its niche (i.e. not an invasive species) and therefore needs to be considered (and addressed) when using it for a species on the move.
4. The is no clear hypothesis or reason for doing the study – An. stephensi is the 'urban malaria vector' so showing it is associated with urban areas is not adding specifically to the current knowledge base
5. The conclusions seem to be drawn from the modelling with the assumption that the modelling is reality – rather than describing the data provided – nor considering what the outputs might be inferring – e.g. population density itself is wouldn't cause increased suitability for ay mosquitoes – but some of the anthropogenic land use changes that accompany increasing human populations may be altering the environment favourably for a mosquito that is adaptable to artificial larval sites.
6. There is a lack of necessary detail throughout – for example, what are the three phases? Why were they selected?
However, what may be interesting to explore is the authors' work to establish more effective urban/peri-urban, rural covariates. Distinguishing between these categories is notoriously tricky, and often not done very well. A surface that distinguishes well between these categories, includes social impacts and infrastructure, could potentially be extremely useful for future SDMs for multiple species, especially if there was some effort to ground truth estimates.
As it stands, I don't think this paper is ready for publication – I would suggest that it does provide a really interesting and quite comprehensive look at changes in urbanisation across several regions – but the assumption is that urbanisation = An. stephensi habitat. This is not entirely the case – there are lots of nuances in which larval sites this species will use. I would suggest that the paper would be more of use if references to the mosquito were removed! and the focus was just on how these environments have changed – with the discussion including potential implications for mosquito species that are able to inhabit this type of habitats (not just An. stephensi) as well as other impacts.

Detailed comments and edits in the attached file.

Reviewer #2

(Remarks to the Author)
I appreciate the opportunity to review this article and would like to commend the authors for their diligent work in developing a well-structured model analysis. The study effectively investigates the influence of population and urbanization on the habitat suitability of Anopheles Stephensi. The authors reported an increase in habitat suitability within areas undergoing rural-to-urban transition. The study's aims and methodological framework are well-conceived and thoughtfully executed. I offer the comment below that may further refine the manuscript and enhance its suitability for publication

It would be helpful for the authors to clarify whether the occurrence dataset of Anopheles stephensi, as well as the environmental variables at both occurrence and pseudo-absence locations, followed a normal distribution. Given that Pearson's correlation assumes normality and linear relationships, the choice of this method over Spearman's rank correlation, particularly if the data were non-normally distributed, requires further justification (Result section - page 3).

Reviewer #3

(Remarks to the Author)
This study evaluates the effects of urbanization on habitat suitability for a malaria vector in Africa and Asia. The study is interesting and well-presented. However, I have a few comments that could improve the manuscript.

Introduction

•All scientific names of genera and species ought to be italicized.
•In "Introduction" section, paragraph 1. The following sentence should be rephrased: "Prior to 2011, An. Stephensi was the primary malaria found mainly in South Asia and the Arabian Peninsula". Additionally, Sri Lanka is not an African country.
•In "Introduction" section, paragraph 2. The following sentence should be rephrased: " (…) has substantially contribute to eliminate malaria"

Results

•Section "The contribution of factors on the habitat suitability". Paragraph 1. These details (the first four sentences) have already been mentioned in the methodology section, so they should be removed. Here, the authors should focus on describing the main results; there is no need to repeat information or describe the methods. The multicollinearity analysis should be integrated into the BRT model computation.

Discussion

•The discussion could be improved by explaining the possible reasons behind the main scenarios observed in the study area, instead of simply repeating the description of the results.

Methods

•What algorithm did you use to interpolate the data?
•Section "Identifying rural, urban and high-density urban regions". It is unclear how the land use maps (e.g., barren, grassland, cropland) were used to classify the area into rural, urban and high-density regions. Moreover, the criteria used to classify rural areas should be clarified.
•Section "Booster regression trees": Here, the authors should describe the multicollinearity analysis performed before running the BRT model.

Version 1:

Reviewer comments:

Reviewer #1

(Remarks to the Author)
some comments in the uploaded file.

Reviewer #2

(Remarks to the Author)

Reviewer #3

(Remarks to the Author)
I have carefully read the revised manuscript.

The authors made significant improvements and addressed all my previous comments.
The manuscript is now fine and may be accepted.

Version 2:

Reviewer comments:

Reviewer #1

(Remarks to the Author)
Accept

Open Access This Peer Review File is licensed under a Creative Commons Attribution 4.0 International License, which permits use, sharing, adaptation, distribution and reproduction in any medium or format, as long as you give appropriate credit to the original author(s) and the source, provide a link to the Creative Commons license, and indicate if changes were made.
In cases where reviewers are anonymous, credit should be given to 'Anonymous Referee' and the source.
The images or other third party material in this Peer Review File are included in the article's Creative Commons license, unless indicated otherwise in a credit line to the material. If material is not included in the article's Creative Commons license and your intended use is not permitted by statutory regulation or exceeds the permitted use, you will need to obtain permission directly from the copyright holder.
To view a copy of this license, visit https://creativecommons.org/licenses/by/4.0/

Response Letter

Journal: Communications Earth & Environment
Manuscript Title: Urbanization accelerates changes in the habitat suitability of *Anopheles stephensi*
Manuscript ID: COMMSENV-25-4240-T

16 January 2026

Dear Dr. Tobias Landmann, Dr. Martina Grecequet, and Reviewers,

We sincerely thank you for handling our manuscript (COMMSENV-25-4240-T) and for the thoughtful feedback provided by the editors and the three anonymous reviewers. We greatly appreciate the time and effort dedicated to evaluating our work.

In the resubmission, we have carefully addressed all comments and suggestions. The revised manuscript and a version with tracked changes are attached for your review. Both the main text and supporting information have been formatted according to the Communications Journals' templates.

Below, we have provided point-by-point responses to the reviewers' comments, with our replies presented in blue font (the red font indicates the text in the revised manuscript). **The first three sections address the overall comments in the e-mail provided by each reviewer. The final section responds to the specific comments marked by Reviewer #1 in the attached file.**

We confirm that no part of this manuscript is under consideration for publication elsewhere.

Thank you again for your consideration. We look forward to your decision and are available for any further clarifications.

Yours sincerely,

Dr. Qiyong Liu and co-authors.

E-mail: liuqiyong@icdc.cn

Response to reviewers:

Reviewer #1

The manuscript reports a study looking to use BRT to establish the habitat preferences of An. stephensi. While it is clear the authors have put in a lot of work, there are some significant issues with this work:

We feel great thanks for your professional review work on our manuscript. These detailed comments and edits are really valuable and helpful for revising and improving our manuscript. We answered all the concerns in a comprehensive manner. The detailed corrections and responses are listed below.

【Question 1】

There does not appear to have been consideration of the limitations and problematic issues that have to be accounted for when used in data amalgamations like those from GBIF – specifically, data quality, data sampling, bias data. GBIF data is not very clean, nor has undergone any quality checks. The data will be reporting where sampling of this species has occurred (i.e. the presence of an entomologist PLUS the mosquito) and so is hugely biased to areas that are accessible (close to research stations, for example). This is not a comprehensive sampling regime (which is what SDMs really require) but as unbiased data are rarely available, it is possible to address the bias in the modelling – that doesn't look like it has been done here.

【Response 1】

Thanks for the kind and insightful comments and suggestions. We also agree with the reviewer's perspective that issues, such as data quality, sampling and bias can affect the results of the manuscript. Therefore, we have checked the data quality, and corrected data bias from sampling from the following three approaches to obtain more precise results.

First, the data of *An. stephensi* occurrence was collected from GBIF, WHO and literatures (Faulde et al., 2014; Abubakr et al., 2022; Hemming-Schroeder and Ahmed, 2023; Teka et al., 2023; Meshesha Balkew, 2021; Al-Eryani et al., 2023). We agree with the view that GBID data is not very clean, and have conducted quality checks on both GBIF and WHO data. Specifically, we only retain occurrence points with complete records containing date, latitude and longitude information. We examined these coordinates and excluded points in obviously incorrect locations, such as locating in the ocean or where the latitude and longitude coordinates did not align with national administrative regions. The amount of data from GBIF and WHO is 670 and 844, respectively. We also searched for literatures on *An. stephensi* using the

keyword "*Anopheles stephensi*", "*An. stephensi*", "*An. stephensi suitability habitat*", "*An. stephensi habitat*", "*Anopheles stephensi suitability habitat*" and "*Anopheles stephensi habitat*" in Web of Science, PubMed and Google Scholar, and six relevant literatures were founded, which recorded the complete locations and dates of *An. stephensi*. We then collecting a total of 202 records from the literatures (Faulde et al., 2014; Abubakr et al., 2022; Hemming-Schroeder and Ahmed, 2023; Teka et al., 2023; Meshesha Balkew, 2021; Al-Eryani et al., 2023). We merged these data, removed duplicate, and obtained a total of 632 occurrence points, which has environment data, in the study region (54 in Phase I, 132 in Phase II and 446 in Phase III). The distribution of occurrence points is shown in Fig. S9. We have added the detailed illustration in Line 429-443 and the text in the revision is as followed.

"We obtained occurrence global records of *An. stephensi* from 1992 to 2021 through GBIF[39], WHO[40] and literatures[20,41-45]. For data from GBIF and WHO, we only retain occurrence points with complete records containing date, latitude and longitude information. We examined these coordinates and excluded points in obviously incorrect locations, such as locating in the ocean or where the latitude and longitude coordinates did not align with national administrative regions. The amount of data from GBIF and WHO is 670 and 844, respectively. We also searched for literatures on *An. stephensi* using the keyword "*Anopheles stephensi*", "*An. stephensi*", "*An. stephensi suitability habitat*", "*An. stephensi habitat*", "*Anopheles stephensi suitability habitat*" and "*Anopheles stephensi habitat*" in Web of Science, PubMed and Google Scholar, and six relevant literatures[20,41-45] were found, which recorded the complete locations and dates of *An. stephensi*. We then collecting a total of 202 records from the literatures. Finally, we merged the data from these three sources, removed duplicate, and obtained a total of 632 occurrence points in the study region (54 in Phase I, 132 in Phase II and 446 in Phase III). The distribution of occurrence points is shown in Fig. S9."

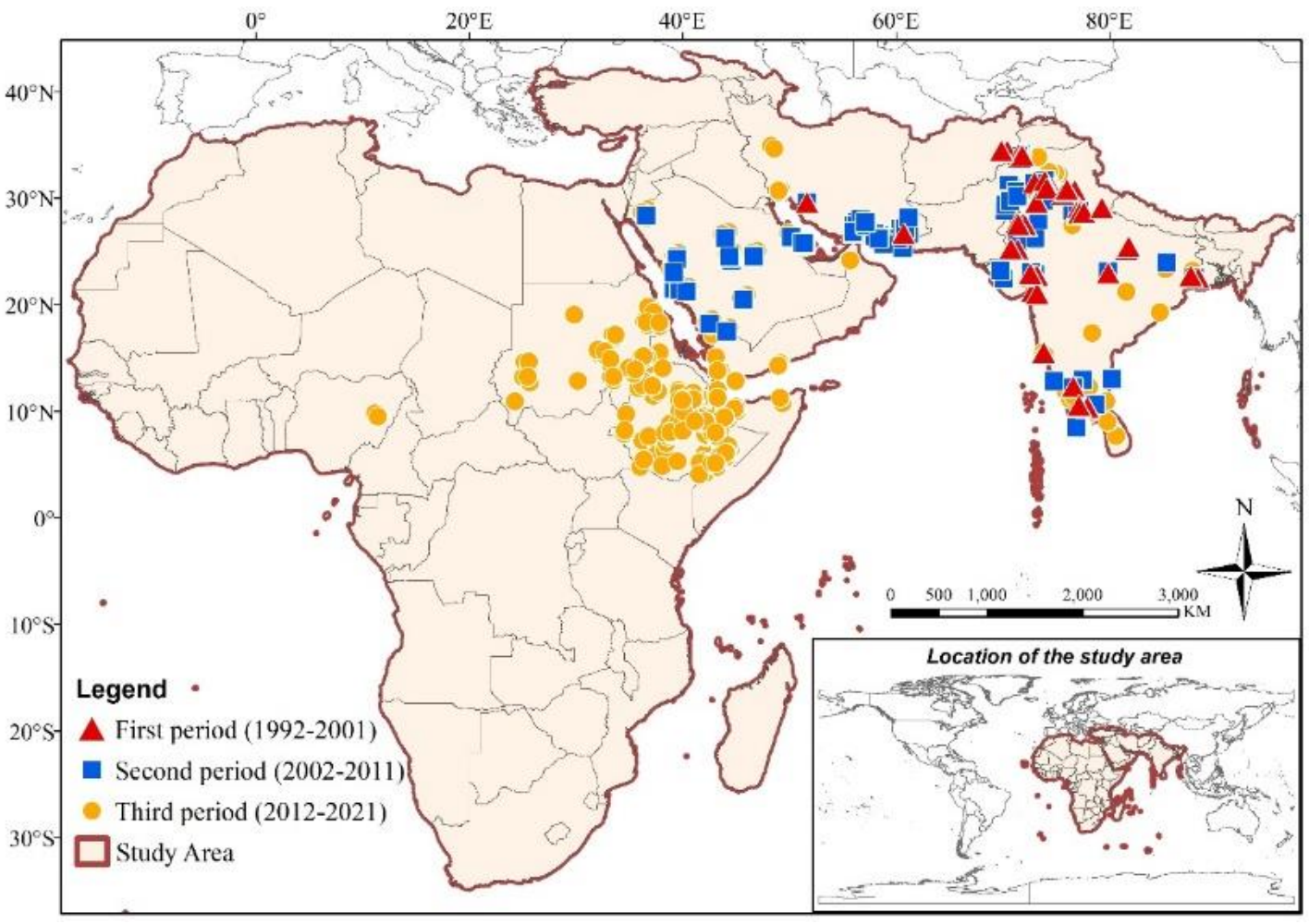

Supplementary Fig. S9. The recorded points of *An. stephensi* during 1992-2021. The numbers of occurrence points in three phases are 54, 132 and 446.

Second, we used kernel density estimation (KDE) to address sampling bias in occurrence points during generating background points. We assigned probability

weights to each grid cell using the kernel density estimation (KDE) to establish a sampling similar with the occurrence points. The results of KDE are shown in Fig. S10. Furthermore, we used the environmental background based technique from the literature (Chambault et al., 2021) to generate background points based on KDE. The technique assumed that absent points were distributed in areas where the environment was different from that of the occurrence points. A principal component analysis (PCA) was used to obtain a two-dimensional environmental background, and we conducted a restricted convex hull, which the environment outside the restricted convex hull was considered to the unfavorable environment for *An. stephensi*. Combined with the probability on the grid cells, pseudo-absence points were randomly generated outside this convex hull with the same number of points as the occurrence points in three phases. These pseudo-absence points were reprojected back into the geographic space unfavorable to the emergence of *An. stephensi*. To improve the robustness of the results, we simulated 10 different sets of background points. The results in the revision were the average outputs of the 10 sets of background points. We also elaborated on this method in detail in Line 444-459 and the text in the revision is as follows.

"Due to the lack of true species absence data, most studies conducted pseudo-absence algorithms to generate background points[46,47]. We generated background points by using an environmental background based technique[47], which assumed that absent points were distributed in areas where the environment was different from that of the occurrence points. A principal component analysis (PCA) was used to obtain a two-dimensional environmental background, and we conducted a restricted convex hull, which the environment outside the restricted convex hull was considered to the unfavorable environment for *An. stephensi*. To account for sampling bias, for example sampling may be tend to target densely populated areas, we assigned probability weights to each grid cell using KDE to establish a sampling similar with the occurrence points. The results of KDE are shown in Fig. S10. Combined with the probability on the grid cells, pseudo-absence points were randomly generated outside this convex hull with the same number of points as the occurrence points in three phases. These pseudo-absence points were reprojected back into the geographic space unfavorable to the emergence of *An. stephensi*. To improve the robustness of the results, we simulated 10 different sets of background points."

However, as the review point out, even if we adopted the method to correct sampling bias, it would be difficult to obtain unbiased data. Therefore, we have expanded the discussion of limitations to include an explanation of data bias in the manuscript. The text in Line 397-401 in the revision is as follows.

"First, it is recognized that a perfect and unbiased sampling regime is challenging. While our methodological approach aims to mitigate this known data bias, some inherent biases associated with the original occurrence data may persist. Future study incorporating systematic field surveillance across diverse environments would be critical."

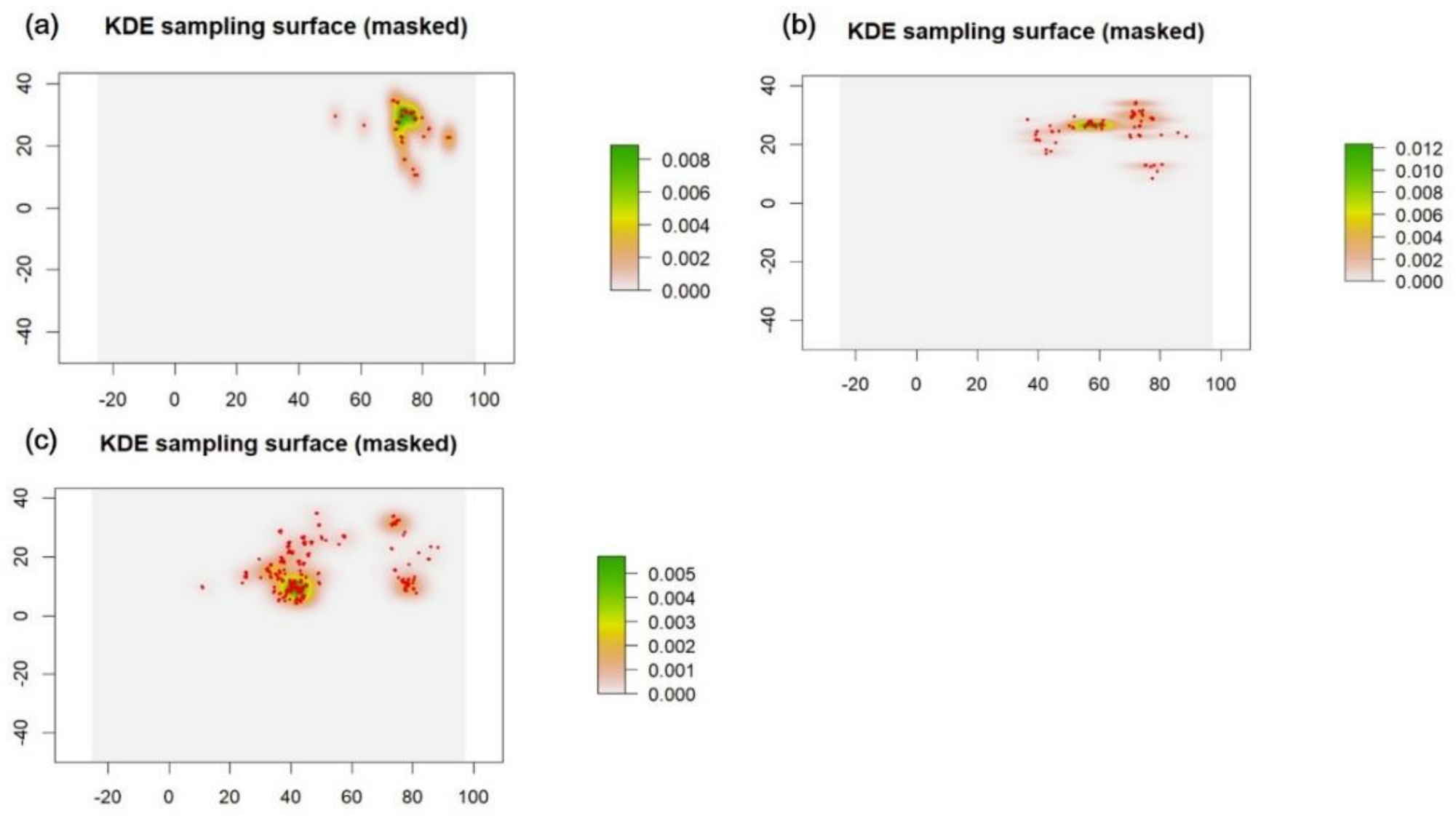

Supplementary Fig. S10. The results of kernel density estimation (KDE) in the three phases. The panel shows the probability value assigned to each grid based on the occurrence points. The red points are occurrences points in the three phases. a Phase I. b Phase II. c Phase III.

【Reference】

Abubakr M, Sami H, Mahdi I. The Phylodynamic and Spread of the Invasive Asian Malaria Vectors, Anopheles stephensi, in Sudan[J]. Biology. 2022, 11(3): 409.

Al-Eryani S M, Irish S R, Carter T E. Public health impact of the spread of Anopheles stephensi in the WHO Eastern Mediterranean Region countries in Horn of Africa and Yemen: need for integrated vector surveillance and control[J]. Malaria Journal. 2023, 22(1): 187.

Chambault, P. et al. A methodological framework to predict the individual and population‐level distributions from tracking data. Ecography (Copenhagen). 44, 766-777 (2021).

Faulde M K, Rueda L M, Khaireh B A. First record of the Asian malaria vector Anopheles stephensi and its possible role in the resurgence of malaria in Djibouti, Horn of Africa[J]. Acta tropica. 2014, 139: 39-43.

Hemming-Schroeder E, Ahmed A. Anopheles stephensi in Africa: vector control opportunities for cobreeding An. stephensi and Aedes arbovirus vectors[J]. Trends in parasitology. 2023, 39(2): 86-90.

Meshesha Balkew P M G Y. An update on the distribution, bionomics, and insecticide susceptibility of Anopheles stephensi in Ethiopia, 2018-2020[J]. Malaria Journal. 2021(20): 263.

Teka H, Golassa L, Medhin G. Trend analysis of malaria in urban settings in Ethiopia from 2014 to 2019[J]. Malaria Journal. 2023, 22(1): 235.

【Question 2】

It is not clear what novel ideas or findings are being reported – it shows the species may have a preference for urban areas (but in Africa, the sampling for this particular species has almost exclusively been in urban areas – it has now been shown to be present in rural areas in Ethiopia).

【Response 2】

We sincerely thank the reviewer for the helpful comment. We aim to analyze the mechanisms by which urbanization affects *An. stephensi* in the manuscript. We now clarify novelty in three aspects.

First, the results of the study identified key indicators of how urbanization affected the habitat suitability of *An. stephensi*. Meanwhile, previous studies that predicted the potential distribution of *An. stephensi* have only considered climatic factors (Liu et al., 2024; Acosta et al., 2025). Our study demonstrated that urbanization indicators, including share of urban land and population, could be included in the future studies to predict the potential distribution of *An. stephensi* and malaria risks more accurately. The text in Line 49-64 in 'Introduction' section and in Line 289-297 in 'Discussion' section are as follows, respectively.

"Environmental conditions (i.e. temperature and precipitation) were associated with the growth and breeding process of *An. stephensi*[10-13]. For example, the Iranian study indicated that the monthly density of adult and larvae *An. stephensi* was significantly correlated with precipitation, maximum and average temperature[14]. Moreover, *An. stephensi* can tolerate extreme hot and dry conditions, enabling them to adapt to the African envirnoment[15]. In addition, the studies focused on the climate-driven potential distribution of *An. stephensi*[16-18], suggesting that the current distribution of *An. stephensi* habitat suitability would be changed in the future, with previously unsuitable regions becoming suitable[17,19]. However, the larvae of *An. stephensi* was found in artificial environments, such as barrels, household water storage containers and cement/metal/plastic tanks[20,21], which may increase the malaria risk in African cities[22]. Therefore, predicting habitat suitability of *An. stephensi* should contain urbanization factors. In addition, although previous studies have indicated that *An. stephensi* can adapt to urban environment, the mechanisms by which urbanization factors affect its larvae sites remain unclear, which is critical for designing surveillance strategies and implementing targeted mosquito control measures in cities, especially in Africa."

"Share of urban land and population within urbanization indicators are key factors influencing the habitat suitability of *An. stephensi* (Fig. 1). We selected two metrics to represent urbanization based on the previous study, including share of urban land and population[23]. Furthermore, another previous study also indicated that population played an important role in the potential distribution of *An. stephensi*[24], which is similar to our results. Meanwhile, previous studies that predicted the potential distribution of *An. stephensi* have only considered climatic factors[17,19]. The study demonstrated that urbanization indicators, including share of urban land and population, could be included in the future studies to predict the potential distribution of *An. stephensi* more accurately."

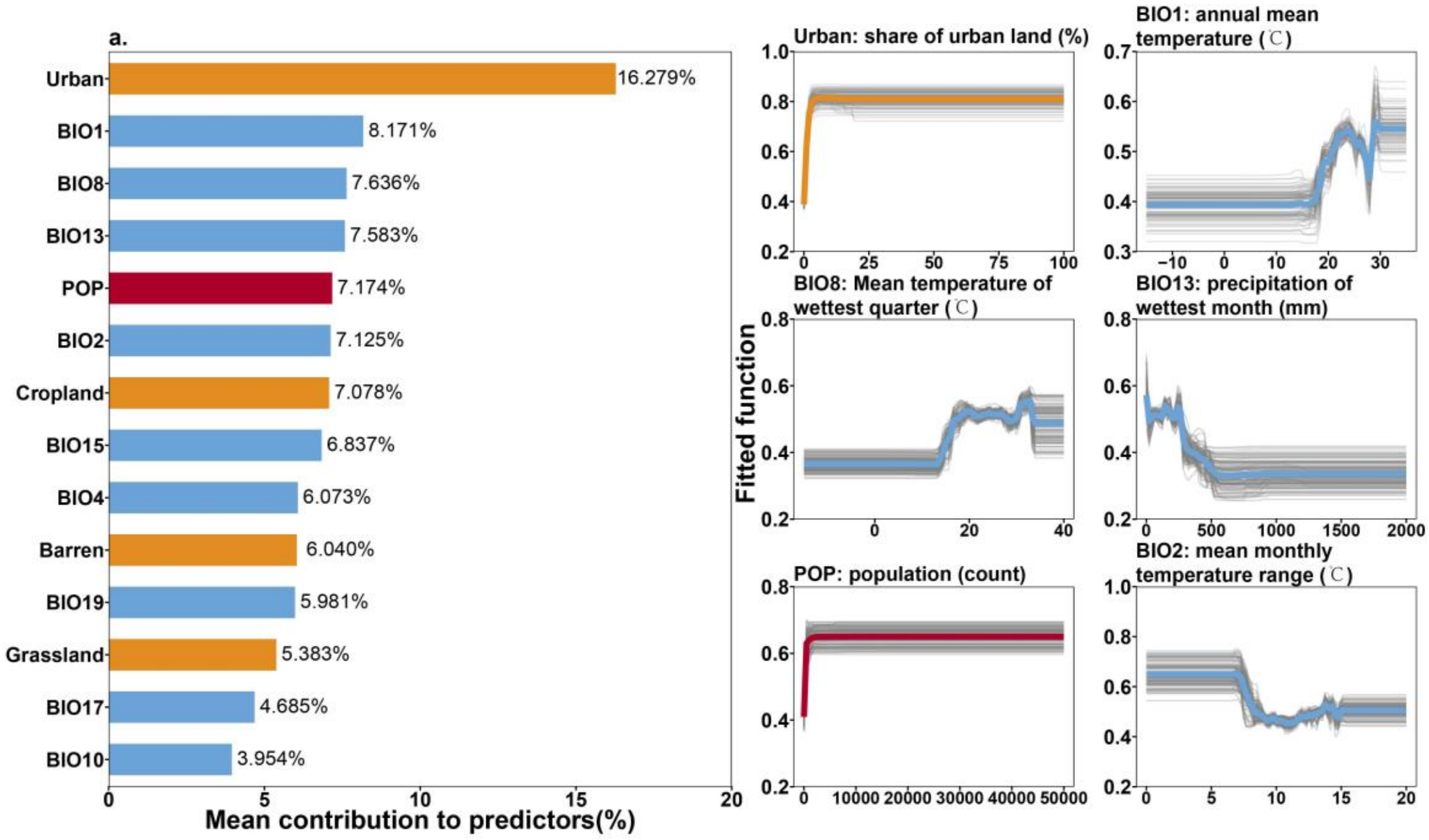

Fig. 1 The impact of factors on habitat suitability of *An. stephensi*. a the mean contribution of factors to the habitat suitability. The factors included in the BRT models were selected based on the Spearman's correlation (Fig. S1). **b** the panel of the response curves of habitat suitability to the top six factors. The response curves of habitat suitability to other factors are shown in Fig. S2. The solid lines are the average of 100 repetitions, while the grey lines are the results of individual runs. The details about the other factors are illustrated in Supplementary Table S2. BIO1: annual mean temperature. BIO2: mean monthly temperature range. BIO4: temperature seasonality. BIO8: mean temperature of wettest quarter. BIO10: mean temperature of coldest quarter. BIO13: precipitation of wettest month. BIO15: precipitation seasonality. BIO17: precipitation of driest quarter. BIO19: precipitation of coldest quarter. Barren: share of barren. Cropland: share of cropland. Grassland: share of grassland. Urban: share of urban land. POP: population.

Second, the previous studies understood the relationship between *An. stephensi* and urbanization primarily relied on field surveys conducted in specific areas (Hawaria et al., 2023; Thomas et al., 2016). These studies through investigation methods have found *An. stephensi* preference for artificial containers and urban environments, demonstrating significant pioneering value. However, such methods have inherent limitations in term of temporal and spatial scales: the conclusions are highly dependent on the selection of survey points, making extrapolation difficult; they primarily reflect static, current distributions, fail to quantify macro-level drivers, and struggle to predict future expansion risks at the larger scale. To address this knowledge gap, this study quantitatively analyzed the contribution of urbanization as a core driver and estimated its potential distribution by integrating land-use and climate factors. The study overcomes the limitations of traditional methods in spatial extrapolation and macro-level mechanism identification, providing scientific basis for regional forward-looking prevention and control strategies. The text in Line 355-359 in the revision is as follows.

"Compared with previous investigation studies[21,25], our study incorporated quantitative analysis and revealed the mechanisms by which urbanization affected the

habitat suitability on a large scale. It can provide scientific reference for better predicting the distribution of *An. stephensi* and design strategies to response to malaria transmission in the future."

Final, the study found that urbanization drove changes in the habitat suitability of *An. stephensi* mainly through construction activities. Meanwhile, we also found that the proportion of people living in slums in cities also increased the habitat suitability. The former created temporary waterlogged habitats such as densely packed construction sites during the building boom. The later resulted in the accumulation of household water storage containers and solid waste due to the lack of basic services. It provides policymakers with a scientific reference for designing strategies to eliminate larval sites of *An. stephensi*. The text in Line 360-396 in the revision is as follows.

"Africa is one of the regions with the fastest urbanization globally[29], and is facing unprecedented population growth in the 21st century, much of which will be absorbed by urban areas, with the total urban population projected to triple from current levels[30]. Malaria in Africa has been commonly considered a rural transmission disease[31]. However, we found that urbanization increased the habitat suitability in Africa (Fig. 3d and Fig. 4i-l), which might pose a malaria risks to African cities. The previous study also indicated that the African cities face challenges to malaria control, mainly due to unplanned urbanization and transmission of *An. stephensi*[32]. In fact, there have been multiple malaria outbreaks caused by the invasion of *An. stephensi* in Africa[33,34]. For example, spatial aggregation of *Plasmodium falciparum* infection around malaria patients in Ethiopia was associated with *An. stephensi* in the vicinity of households under a prospective case-control design experiment[33]. Furthermore, urbanization also affected the habitat suitability was mainly through construction activities (Fig. 5b). The study in Ethiopia also demonstrated that mass urban migration and unprecedented construction booms across African cities were likely facilitating the expansion of *An. stephensi*, creating new larval habitats[35]. Although the differences in behaviors of *An. stephensi* between Africa, and South and West Asia are not included in the study, it is certain that *An. stephensi*, which adapt to both rural and urban environments, may add a new threat to malaria transmission in Africa. It needs to apply targeted mosquito control technologies and strengthen surveillance to address malaria transmission in Africa.

An. stephensi was the malaria vector mainly in South Asia and the Arabian Peninsula before 2011[2]. Many studies in Asia confirmed that *An. stephensi* was transmitted malaria mainly in cities[36,37], for example, the study indicated that *An. stephensi* was a major urban malaria vector in India[37]. Our study also indicated that the habitat suitability was high in urban and high-density urban areas in South and West Asia (Fig. 2). The changes in habitat suitability in South Asia and West Asia were relatively lower than that in Africa, and even decreased in some areas (Fig. 3). Single-factor analysis indicated that this decline in some areas might be climate-driven. For instance, the reduction in the habitat suitability in southeastern India coincided with a climate-driven decrease in habitat suitability (Fig. 3 and Fig. S5). Furthermore, urbanization also primarily affected the habitat suitability through construction

activities. However, urbanization could increase the proportion of people living in slums in cites, which differed from the results in the overall study area and Africa (Fig. 5c). Urbanization in Asia was progressing faster than in Africa[38], which may lead to regional development imbalances, resulting in an increase in slums. Therefore, urban planning should be prioritized during urbanization to reduce *An. stephensi* breeding sites."

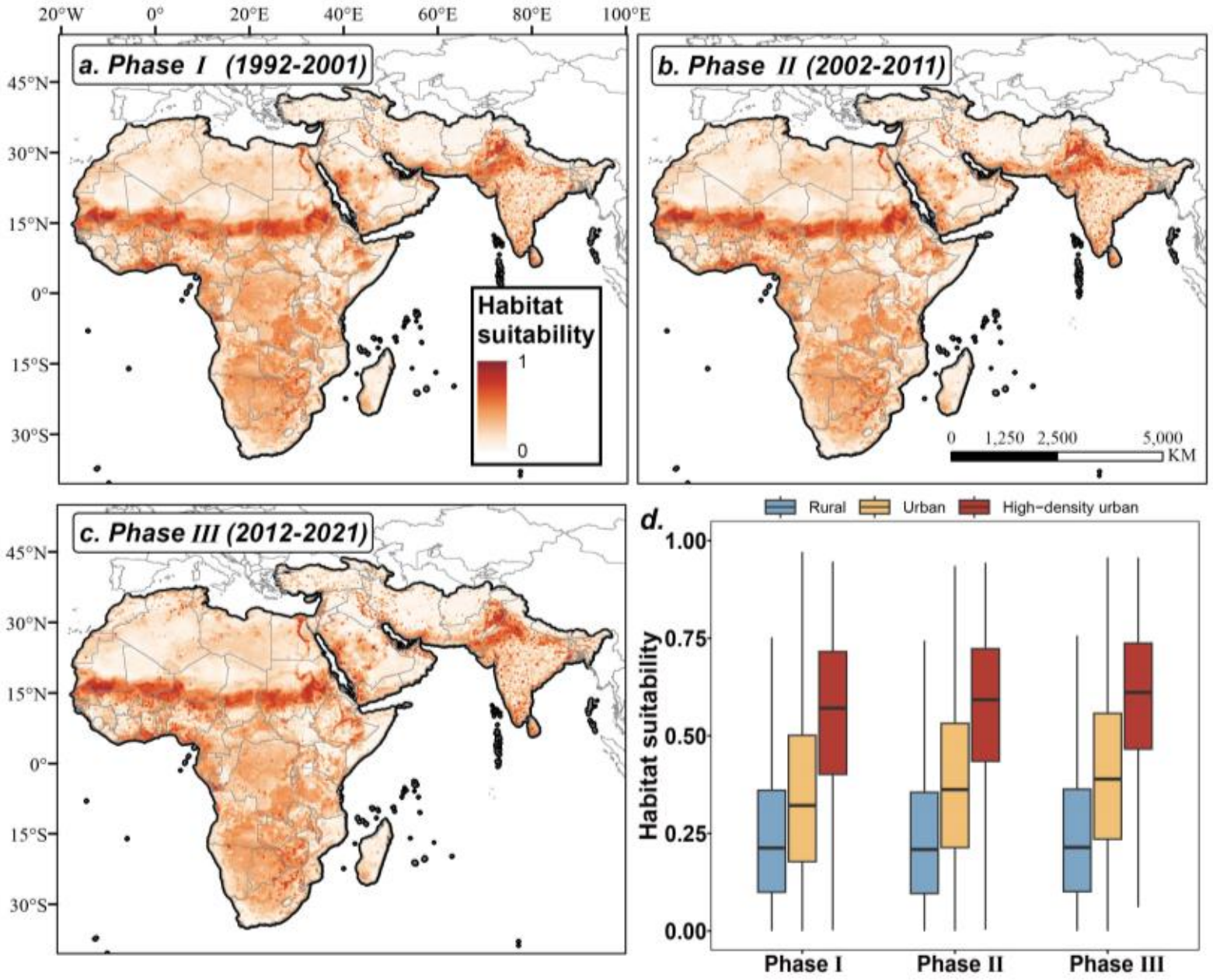

Fig. 2 The predicted habitat suitability of *An. stephensi* over three phases. a-c the mean estimated spatial distribution of the habitat suitability from the BRT models across 100 iterations in the three phases. **d** the boxplots represent habitat suitability of rural, urban and high-density urban regions according to the classification in the study. The predicted values are scaled from 0 to 1 through a min-max transformation.

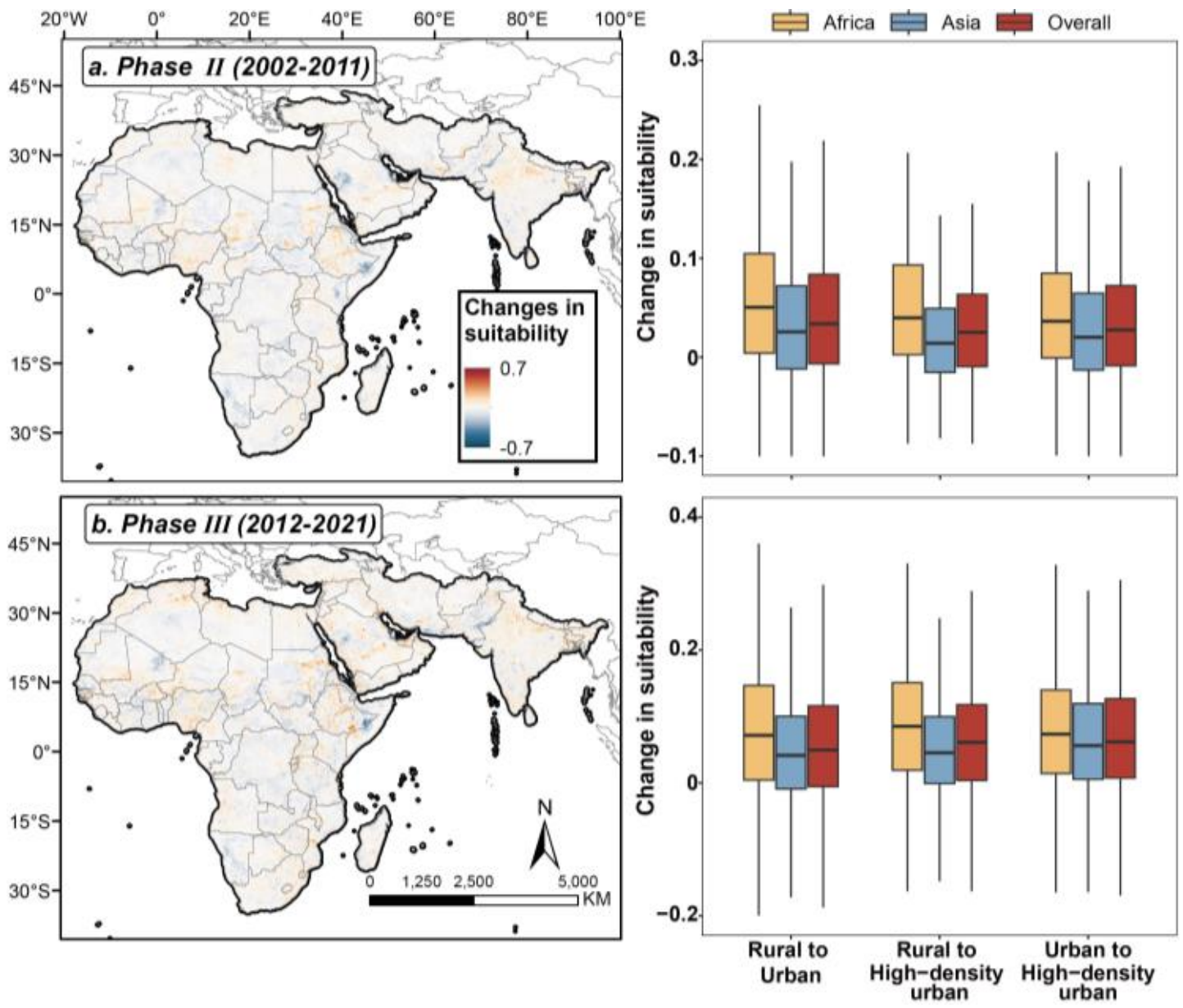

Fig. 3. The changes in predicted habitat suitability of *An. stephensi* in Phase II and III. a and b the spatial distribution of changes in habitat suitability in Phase II and Phase III, respectively. **c**

and d the boxplots of changes in habitat suitability in Africa, Asia and the whole study area in Phase II and Phase III, respectively. The baseline was Phase I. The differences in the habitat suitability between other two phases and baseline were the changes in habitat suitability in Phase II and Phase III, respectively. The habitat suitability in Fig.3 was the average of 100 iterations and normalized by a min-max transformation.

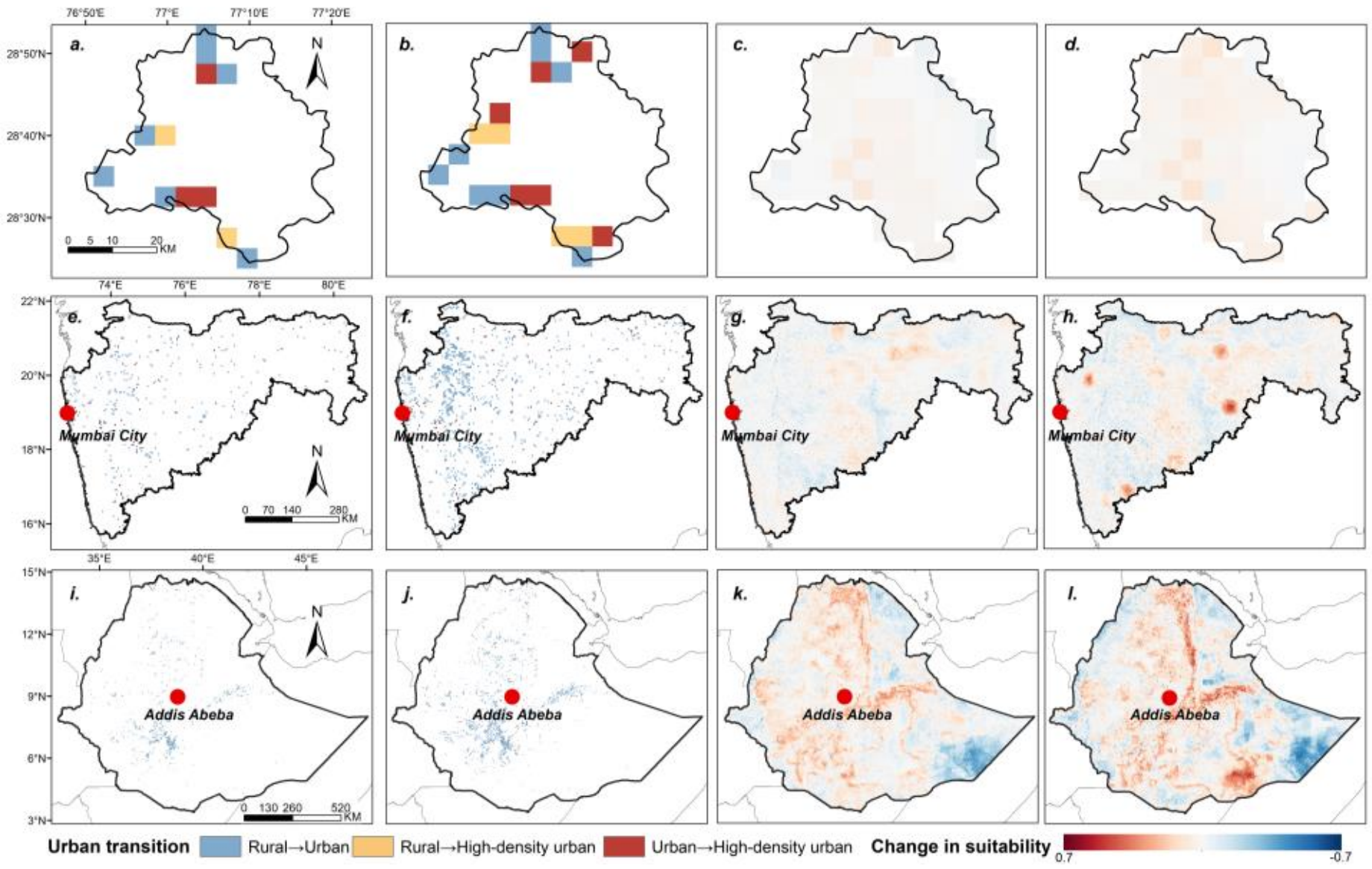

Fig. 4 The changes in predicted habitat suitability of *An. stephensi* in the small scale. a and b, e and f, i and j the changes in rural, urban and high-density urban areas in Phase II and Phase III in New Delhi, India, Maharashtra State, India and Ethiopia, respectively. **c and d, g and h, k and l** the changes in habitat suitability of *An. stephensi* in Phase II and Phase III in New Delhi, India, Maharashtra State, India and Ethiopia, respectively. The baseline was Phase I. The habitat suitability was the average of 100 iterations and normalized by a min-max transformation.

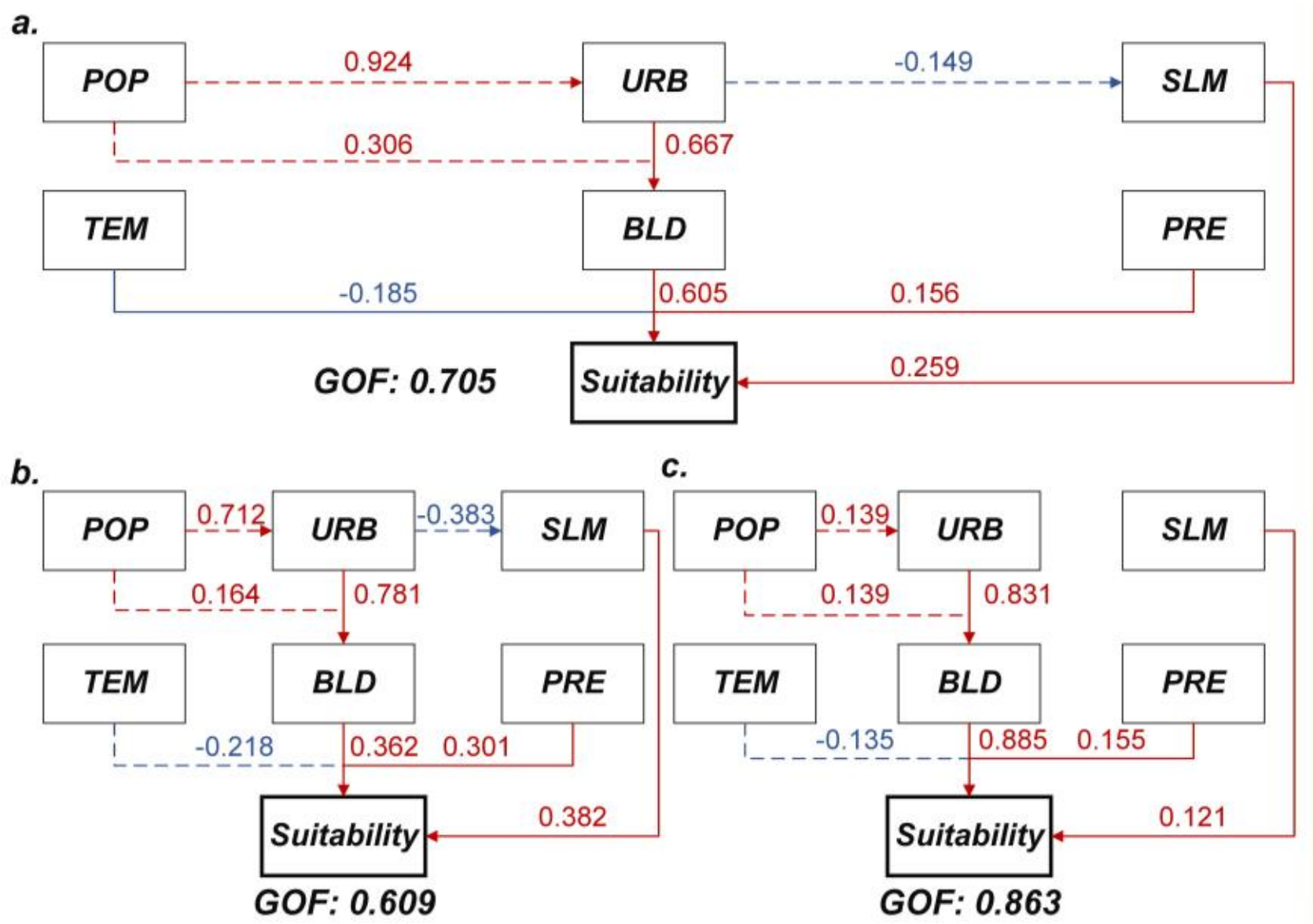

Fig. 5 The mechanisms of factors on habitat suitability of *An. stephensi*. a the results of the PLS-PM model including all data in the study area. **b and c** the results of the model only including data in Africa, and South and West Asia, respectively. The blue line is the negative relationship between factors and habitat suitability and the red line is the positive relationship

between factors and habitat suitability. The solid line represents the direct influence of factors on habitat suitability and the dash line represents the indirect effect of factors on habitat suitability. The path coefficient in the panel is significant ($p < 0.05$). Each box represents a latent variable, and the specific explicit variables included in each of the three models are shown in Table S4. GOF: goodness-of-fit. POP: population. URB: share of urban land. SLM: people living in slums in cities. BLD: construction indexes. TEM: temperature indexes. PRE: precipitation indexes. Suitability: the habitat suitability of *An. stephensi*.

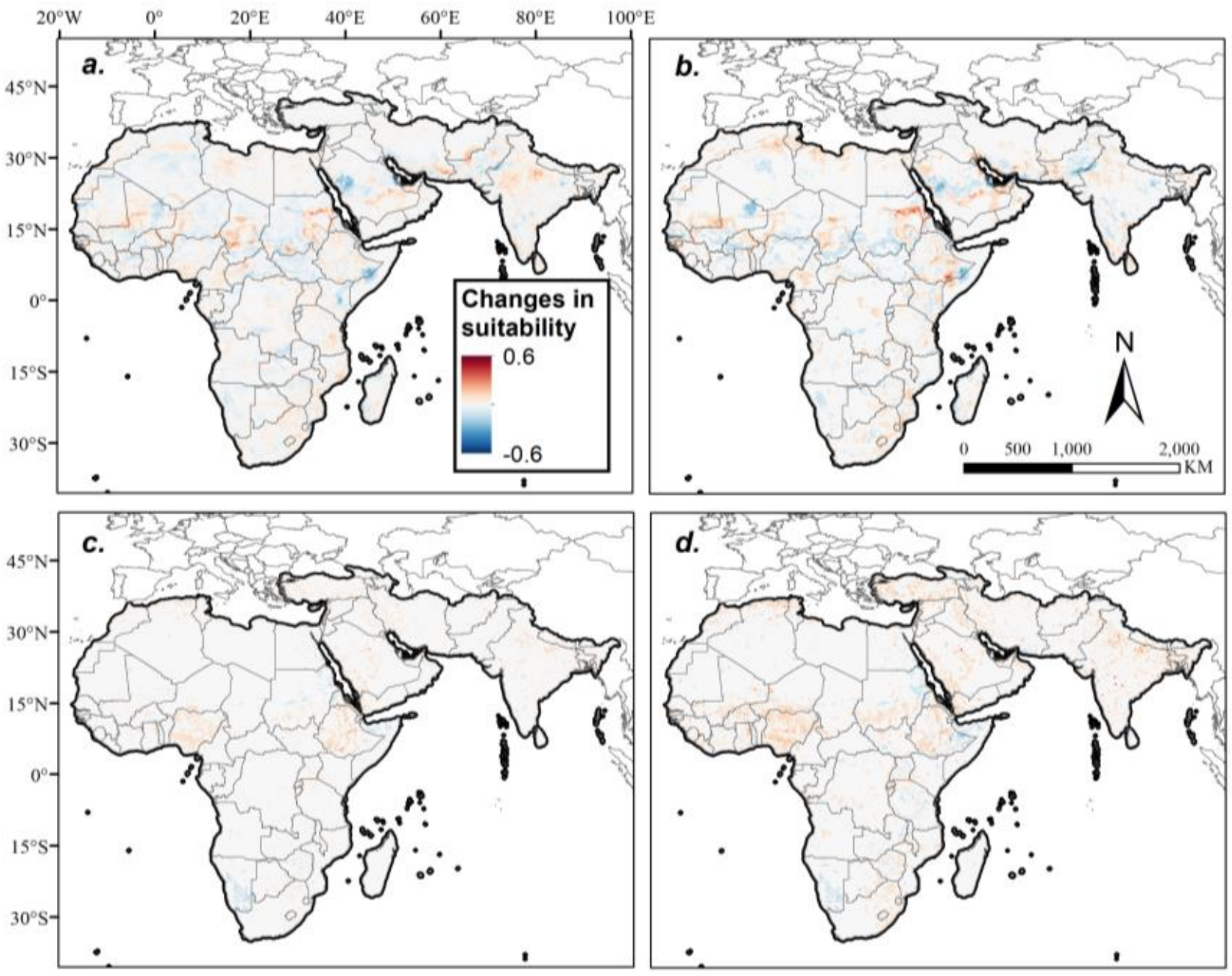

Supplementary Fig. S5. The habitat suitability of *An. stephensi* only affected by climatic factors and other land-use types. We used Phase I as baseline and kept the values of other factors in Phase II and III consistent with Phase I to estimate the habitat suitability only affected by climatic factors (a and b) or other land-use types (c and d). The changes in habitat suitability in Phase II and III were the differences in values between Phase II and III and baseline, respectively.

【References】

Acosta A L, Castro M C, Laporta G Z. Future global distribution and climatic suitability of Anopheles stephensi[J]. Sci Rep. 2025, 15(1): 22268.

Hawaria D, Kibret S, Zhong D. First report of *Anopheles stephensi* from southern Ethiopia[J]. Malaria journal. 2023, 22(1): 373.

Liu Q, Wang M, Du Y. Possible potential spread of *Anopheles stephensi*, the Asian malaria vector[J]. BMC Infectious Diseases. 2024, 24(1): 333.

Thomas S, Ravishankaran S, Justin J A. Overhead tank is the potential breeding habitat of *Anopheles stephensi* in an urban transmission setting of Chennai, India[J]. Malaria journal. 2016, 15(1): 274.

【Question 3】

BRT is a SDM, and they have very specific assumptions, for example, whether the species is fully occupying its niche (i.e. not an invasive species) and therefore needs

to be considered (and addressed) when using it for a species on the move.

【Response 3】

We sincerely thank the reviewer for the valuable comment. We agree that BRT is a SDM, and they are applied to analyze the species that fully occupies its niche, not an invasive species. Therefore, we applied two approaches to address this issue. First, the data used for the BRT model incorporates temporal dimensions. We did not simply used the average of 30 years data into the BRT model. It helps capture the dynamic changes in the habitat suitability of *An. stephensi* rather than predict the static distribution. We added the details about the method into the manuscript and the text in Line 531-532 is as follows.

"The data were temporally partitioned based on the three phases and then concluded into the BRT model."

Second, to validate the robustness of the results in the manuscript, we generated background points using African data alone and constructed the BRT model for analysis. We found that although the ranking of the mean contribution of factors to the habitat suitability has shifted, the importance of share of urban land and population still remains among the top six (Figure S8). It indicated that urbanization was also critical to the habitat suitability in Africa, which is consistent with the overall findings. On the other hand, the differences in importance may also indicate that the habitat characteristics of *An. stephensi* may undergo changes during the invasion process. The researches on *An. stephensi* in Africa is needed in the future. We also discussed it in the 'Discussion' section and the text in Line 298-306 is as follows.

"On the other hand, since BRT is a species distribution modelling (SDM) used to analyze species fully occupying the niche, and *An. stephensi* is an invasive species, we also constructed a BRT model only in Africa. Fig. S8 shows that share of urban land and population also significantly affected the habitat suitability of *An. stephensi*, consistent with the overall finding, which demonstrating the robustness of the results of the BRT model in the study. However, some factors' (i.e. share of grassland) contribution to the habitat suitability was different from the overall findings, suggesting that the ecological characteristics of *An. stephensi* might undergo changes during its invasion process. Future research is needed to explore the habitat characteristics of *An. stephensi* in Africa."

In summary, although SDM requires application to species occupying relatively stable ecological niches, results from both Africa and the overall analysis demonstrate that urbanization had an important impact on the habitat suitability of *An. stephensi*, consistent with our core findings. Meanwhile, the expanded analysis enriches our scientific understanding of the invasion process, indicating that Africa requires more targeted monitoring strategies extending beyond purely urban environments.

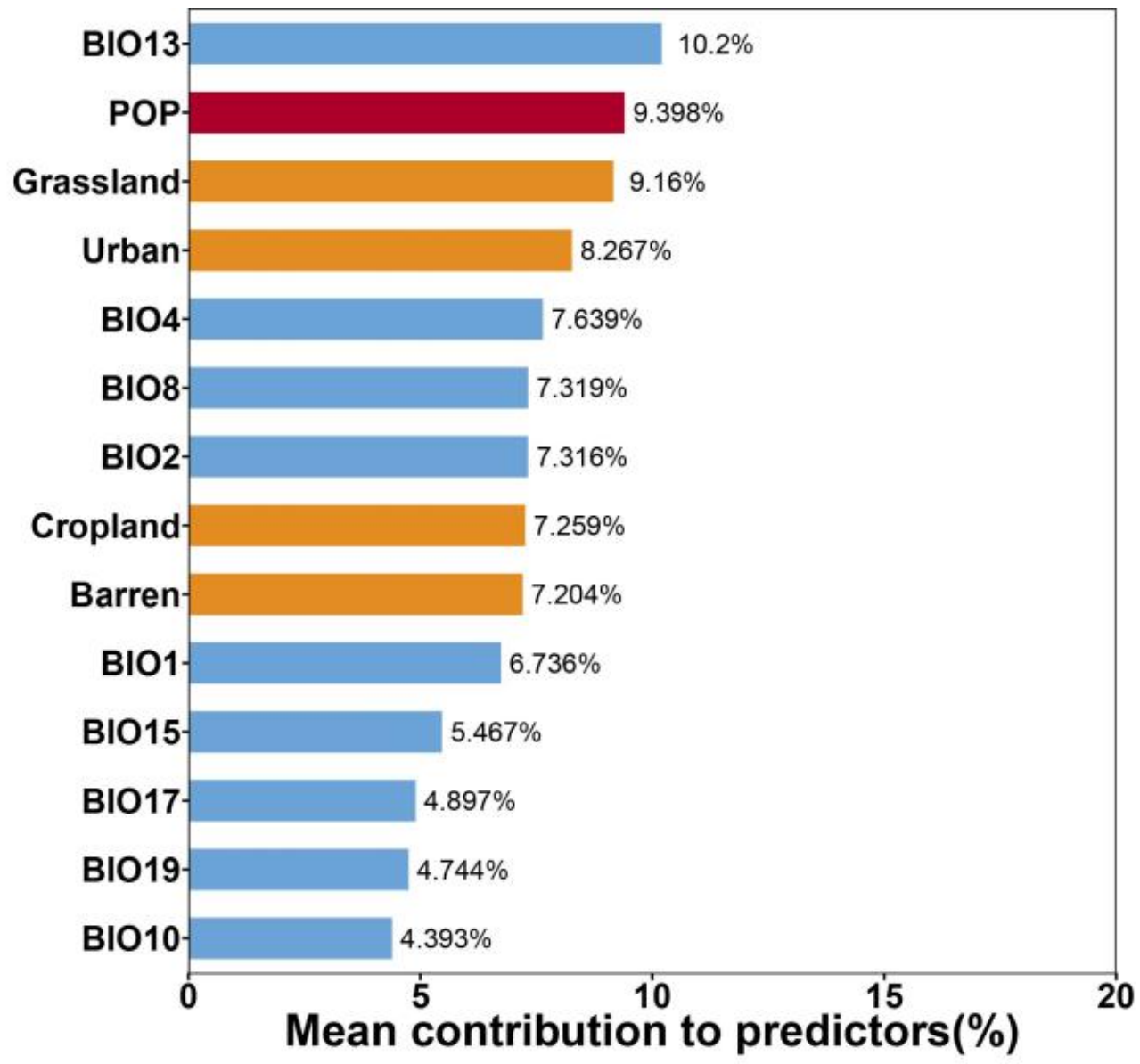

Supplementary Fig. S8. The impact of factors on the habitat suitability of *An. stephensi* in Africa. The factors in this BRT model are the same with the BRT model in overall study area. BIO1: annual mean temperature. BIO2: mean monthly temperature range. BIO4: temperature seasonality. BIO8: mean temperature of wettest quarter. BIO10: mean temperature of coldest quarter. BIO13: precipitation of wettest month. BIO15: precipitation seasonality. BIO17: precipitation of driest quarter. BIO19: precipitation of coldest quarter. Barren: share of barren. Cropland: share of cropland. Grassland: share of grassland. Urban: share of urban land. POP: population.

【Question 4】

This is no clear hypothesis or reason for doing the study – *An. stephensi* is the ‘urban malaria vector’ so showing it is associated with urban areas is not adding specifically to current knowledge base.

【Response 4】

We sincerely thank the reviewer for raising this fundamental question. It allows us to clarify that our study far beyond confirming the known urban affinity of *An. stephensi*. Our central hypothesis was that urbanization drove the changes in the habitat suitability. This hypothesis was tested through three key innovations that substantially advance the current knowledge base:

1. From Qualitative Association to Quantitative and Predictive Drivers. Previous distribution models have relied almost exclusively on climatic factors (Liu et al., 2024; Acosta et al., 2025). Our study is to **quantify that urbanization indicators (share of urban land and population) are the critical predictors**. This proves that future predictive models should incorporate these factors for accuracy. We have strengthened this point in the Introduction (Lines 49-64) and Discussion (Lines 289-397).

“Environmental conditions (i.e. temperature and precipitation) were associated with

the growth and breeding process of *An. stephensi*[10-13]. For example, the Iranian study indicated that the monthly density of adult and larvae *An. stephensi* was significantly correlated with precipitation, maximum and average temperature[14]. Moreover, *An. stephensi* can tolerate extreme hot and dry conditions, enabling them to adapt to the African envirnoment[15]. In addition, the studies focused on the climate-driven potential distribution of *An. stephensi*[16-18], suggesting that the current distribution of *An. stephensi* habitat suitability would be changed in the future, with previously unsuitable regions becoming suitable[17,19]. However, the larvae of *An. stephensi* was found in artificial environments, such as barrels, household water storage containers and cement/metal/plastic tanks[20,21], which may increase the malaria risk in African cities[22]. Therefore, predicting habitat suitability of *An. stephensi* should contain urbanization factors. In addition, although previous studies have indicated that *An. stephensi* can adapt to urban environment, the mechanisms by which urbanization factors affect its larvae sites remain unclear, which is critical for designing surveillance strategies and implementing targeted mosquito control measures in cities, especially in Africa."

"Share of urban land and population within urbanization indicators are key factors influencing the habitat suitability of *An. stephensi* (Fig. 1). We selected two metrics to represent urbanization based on the previous study, including share of urban land and population[23]. Furthermore, another previous study also indicated that population played an important role in the potential distribution of *An. stephensi*[24], which is similar to our results. Meanwhile, previous studies that predicted the potential distribution of *An. stephensi* have only considered climatic factors[17,19]. The study demonstrated that urbanization indicators, including share of urban land and population, could be included in the future studies to predict the potential distribution of *An. stephensi* more accurately."

2. **From Localized Surveys to Macroscopic Mechanisms and Risk Forecasting.** Prior understanding stemmed from invaluable but spatially limited field surveys (Hawaria et al., 2023; Thomas et al., 2016). Our large-scale analysis overcomes this extrapolation limit. **We estimated the spatial distribution of *An. stphensi* habitat suitability and identifies the urbanization accelerated the changes in the habitat suitability.** This spatial dynamic, unreachable via local surveys, is critical for proactive surveillance. We have added this contrast in the revised Discussion (Lines 355-359).

"Compared with previous investigation studies[21,25], our study incorporated quantitative analysis and revealed the mechanisms by which urbanization affected the habitat suitability on a large scale. It can provide scientific reference for better predicting the distribution of *An. stephensi* and design strategies to response to malaria transmission in the future."

3. Revealing the mechanisms by which urbanization affects the habitat suitability of *An. stephensi*. Most importantly, we moved past correlation to **reveal the causal pathways**. Our path model shows urbanization's impact is mediated primarily

by **construction activities** (creating sites like pits and containers). This mechanistic insight shifts policy from generic "urban clean-up" to **targeted interventions at construction sites and through improved basic services in informal settlements**. We have expanded this critical implication in the Discussion (Lines 330-347).

"Urbanization has created more breeding sites for *An. stephensi*. Urban environments are suitable for breeding of *An. stephensi*, which shows preferential breeding in man-made conditions, such as overhand tank[25], artificial water containers, construction pits[26], condensation from air conditioning, open oil drums and water-filled tires after a rainfall[21]. The study further quantitatively revealed the mechanisms of urbanization on the habitat suitability of *An. stephensi* (Fig. 5). We used infrastructure investment and build-up areas to represent the construction activities and found urbanization affected the habitat suitability was mainly through construction activities. Construction booms appear to create more breeding sites for *An. stephensi*[27]. The expansion of urban areas and the growth in population resulted in the need for more buildings, and it then increased the habitat suitability of *An. stephensi*. Artificial water containers prevalent in construction sites, including concrete water tanks and car washing facilities, have become breeding habitats for *An. stephensi*[21]. Although the study did not have construction sites data, infrastructure investment and build-up areas were closely associated with construction sites because all construction projects required construction sites during development. Therefore, it revealed that increased construction due to urban expansion and population growth added more acritical water storage containers, which might create new larval sites for *An. stephensi*."

【***References***】

Acosta A L, Castro M C, Laporta G Z. Future global distribution and climatic suitability of Anopheles stephensi[J]. Sci Rep. 2025, 15(1): 22268.

Hawaria D, Kibret S, Zhong D. First report of *Anopheles stephensi* from southern Ethiopia[J]. Malaria journal. 2023, 22(1): 373.

Liu Q, Wang M, Du Y. Possible potential spread of *Anopheles stephensi*, the Asian malaria vector[J]. BMC Infectious Diseases. 2024, 24(1): 333.

Thomas S, Ravishankaran S, Justin J A. Overhead tank is the potential breeding habitat of *Anopheles stephensi* in an urban transmission setting of Chennai, India[J]. Malaria journal. 2016, 15(1): 274.

【Question 5】

The conclusions seem to be drawn from the modelling with the assumption that the modelling is reality – rather than describing the data provided – nor considering what the outputs might be inferring – e.g. population density itself is wouldn't cause increased suitability for any mosquitoes – but some of the anthropogenic land use changes that accompany increasing human populations may be altering the environment favorably for a mosquito that is adaptable to artificial larval sites.

【Response 5】

Thank for the helpful comment. We have actually considered the inference of the model's output. The study firstly identified the association between urbanization indicators and the habitat suitability through the BRT model. In this section, we analyzed the reasons for the correlation between urbanization and the habitat suitability through investigation studies. The text in Line 289-306 in the 'Discussion' section is as follows.

"Share of urban land and population within urbanization indicators are key factors influencing the habitat suitability of *An. stephensi* (Fig. 1). We selected two metrics to represent urbanization based on the previous study, including share of urban land and population[23]. Furthermore, another previous study also indicated that population played an important role in the potential distribution of *An. stephensi*[24], which is similar to our results. Meanwhile, previous studies that predicted the potential distribution of *An. stephensi* have only considered climatic factors[17,19]. The study demonstrated that urbanization indicators, including share of urban land and population, could be included in the future studies to predict the potential distribution of *An. stephensi* more accurately. On the other hand, since BRT is a species distribution modelling (SDM) used to analyze species fully occupying the niche, and *An. stephensi* is an invasive species, we also constructed a BRT model only in Africa. Fig. S8 shows that share of urban land and population also significantly affected the habitat suitability of *An. stephensi*, consistent with the overall finding, which demonstrating the robustness of the results of the BRT model in the study. However, some factors' (i.e. share of grassland) contribution to the habitat suitability was different from the overall findings, suggesting that the ecological characteristics of *An. stephensi* might undergo changes during its invasion process. Future research is needed to explore the habitat characteristics of *An. stephensi* in Africa."

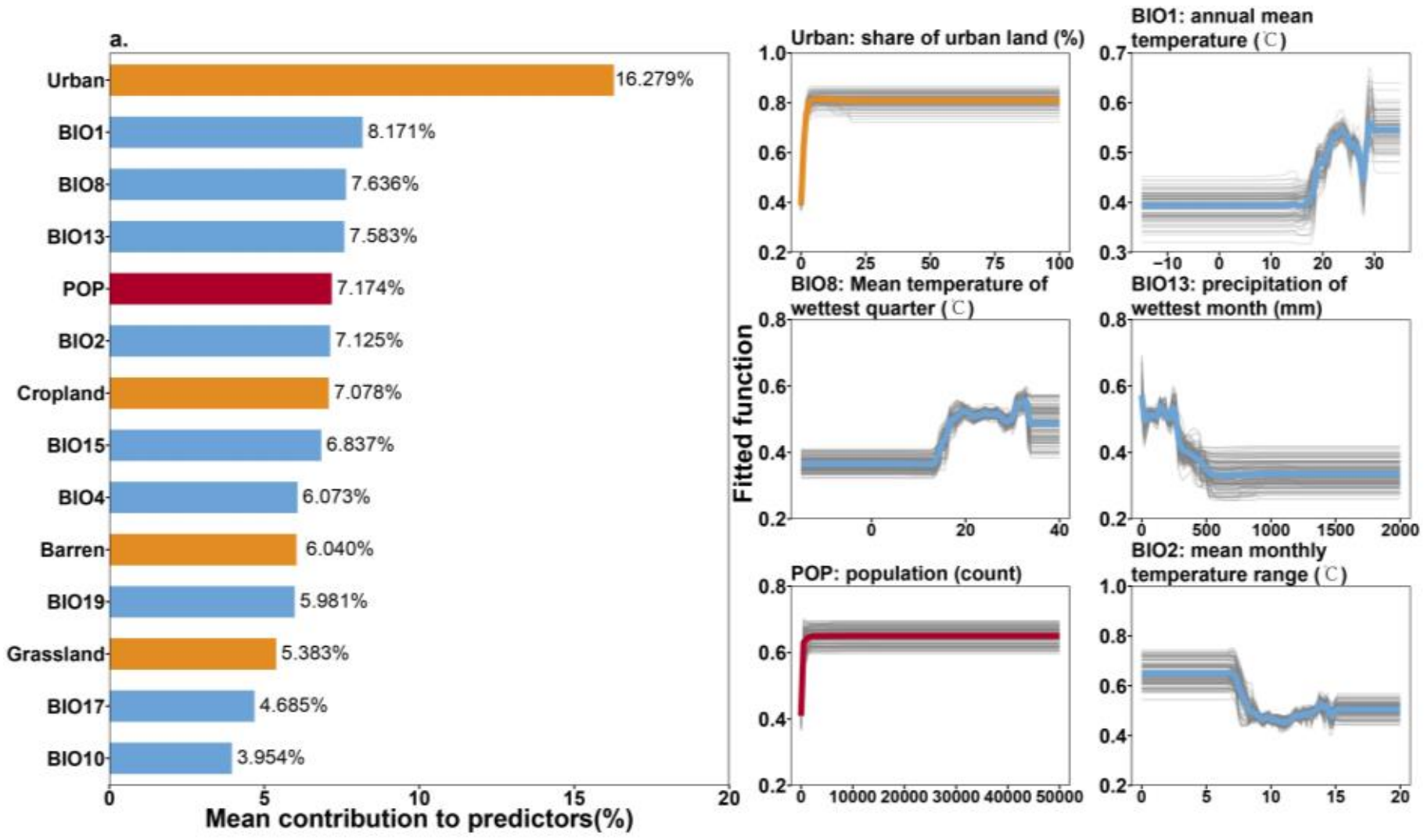

Fig. 1 The impact of factors on habitat suitability of *An. stephensi*. a the mean contribution of factors to the habitat suitability. The factors included in the BRT models were selected based on the Spearman's correlation (Fig. S1). **b** the panel of the response curves of habitat suitability to the

top six factors. The response curves of habitat suitability to other factors are shown in Fig. S2. The solid lines are the average of 100 repetitions, while the grey lines are the results of individual runs. The details about the other factors are illustrated in Supplementary Table S2. BIO1: annual mean temperature. BIO2: mean monthly temperature range. BIO4: temperature seasonality. BIO8: mean temperature of wettest quarter. BIO10: mean temperature of coldest quarter. BIO13: precipitation of wettest month. BIO15: precipitation seasonality. BIO17: precipitation of driest quarter. BIO19: precipitation of coldest quarter. Barren: share of barren. Cropland: share of cropland. Grassland: share of grassland. Urban: share of urban land. POP: population.

Second, we estimated the spatial distribution of the habitat suitability of *An. stephensi* and further explored the its changes during the process of urbanization. We also discussed it in Line 307-329 in the 'Discussion' section, and the text is as follows.

"Our model suggested that urbanization accelerated the changes in the habitat suitability of *An. stephensi*. The habitat suitability of *An. stephensi* increased in Africa during Phase III (Fig. 3), and its invasion into Africa since 2012[2] also illustrated it. Urbanization progressed rapidly in Phase III, and the number of becoming urban or high-density urban grids reached 2.464 times that of Phase II (Fig. S3), and the urban-to-high-density-urban regions had the largest increase in the habitat suitability (Fig. 3). Despite the reliability of the urban classification, the delineation in the boundaries between urban and rural areas is hard. Because the population in these areas may be close to the urban threshold, and some environments may be quite similar to the habitats of *An. stephensi* in the urban areas. The field researches in the future are required to conduct in-depth analysis of *An. stephensi* larval condition in these areas. However, the results did not indicate that *An. stephensi* larval sites was only found in urban areas. In fact, *An. stephensi* is also adaptive to the rural environment, such as freshwater ponds, stream beds, wells and seepage canals[25]. On the other hand, it is certain that multiple factors, including climate, urban land and population, have influenced the distribution of *An. stephensi*. For example, the habitat suitability in southern Ethiopia has increased, while there was a little urban area. This might be the result of climate effect, as the habitat suitability increased in this region under climate effects alone (Fig. S5). Our study suggested that urbanization and climate change in the future might have great impact on the distribution of *An. stephesni*, potentially enabling its invasion into other continent or countries where climatic conditions were suitable and rural and urban breeding habitat existed. Therefore, the adaptation to both urban and rural environments of *An. stephensi* needs to implement updated mosquito control methods to eliminate it."

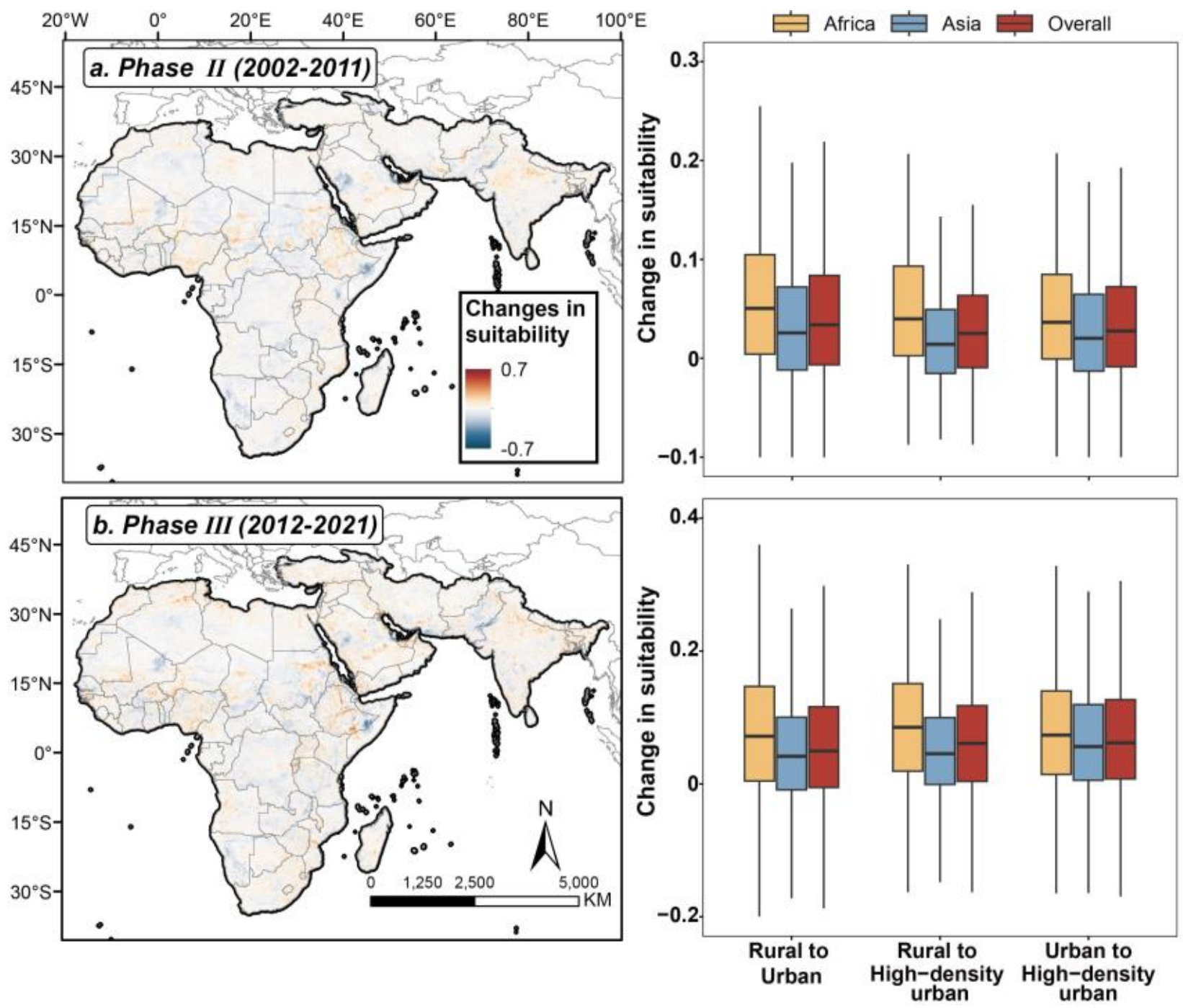

Fig. 3. The changes in predicted habitat suitability of *An. stephensi* in Phase II and III. a and b the spatial distribution of changes in habitat suitability in Phase II and Phase III, respectively. **c and d** the boxplots of changes in habitat suitability in Africa, Asia and the whole study area in Phase II and Phase III, respectively. The baseline was Phase I. The differences in the habitat suitability between other two phases and baseline were the changes in habitat suitability in Phase II and Phase III, respectively. The habitat suitability in Fig.3 was the average of 100 iterations and normalized by a min-max transformation.

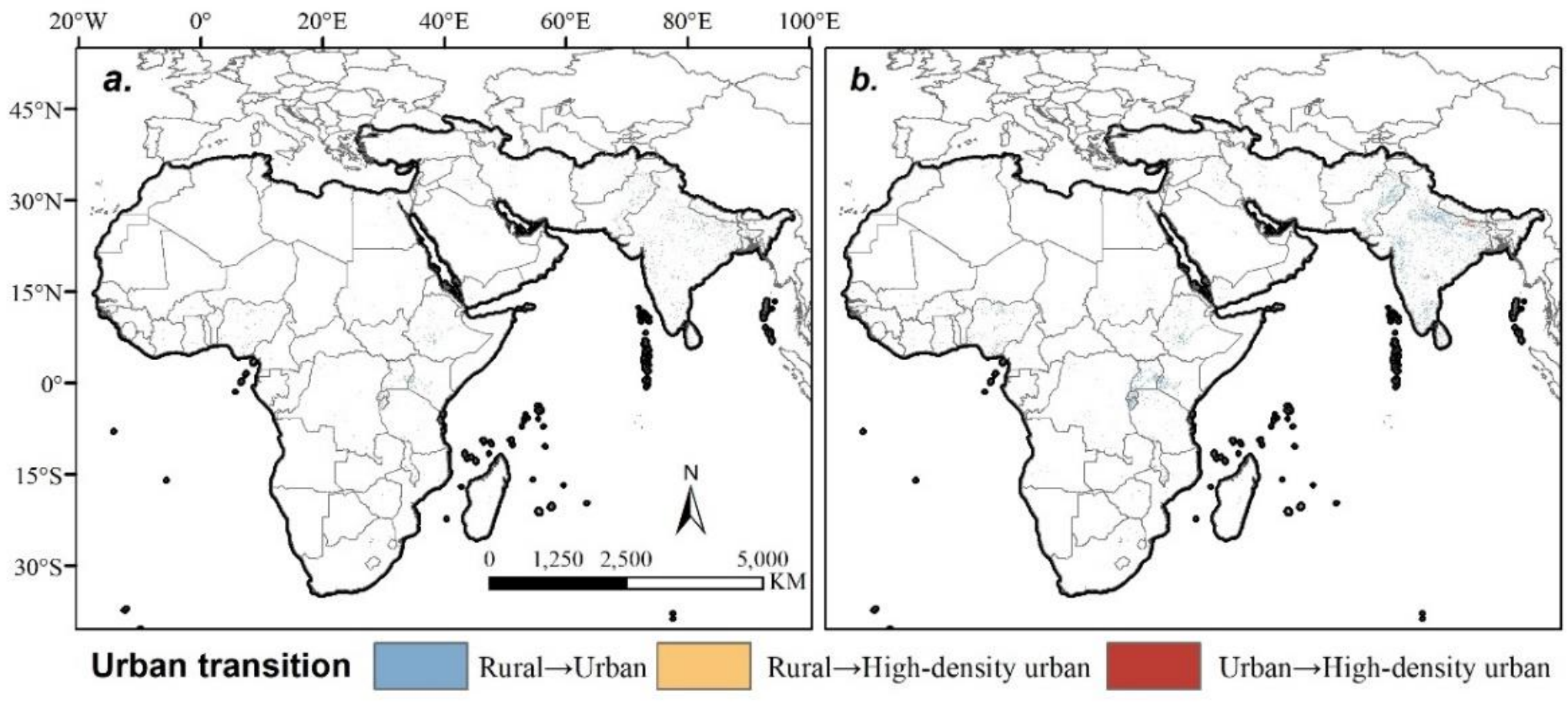

Supplementary Fig. S3. The changed between rural, urban and high-density urban in Phase II and Phase III. Using Phase I as the basic period, we calculated the transformation that rural was replaced by urban or high-density urban, and urban was replaced by high-density urban in Phase II (a) and Phase III (b).

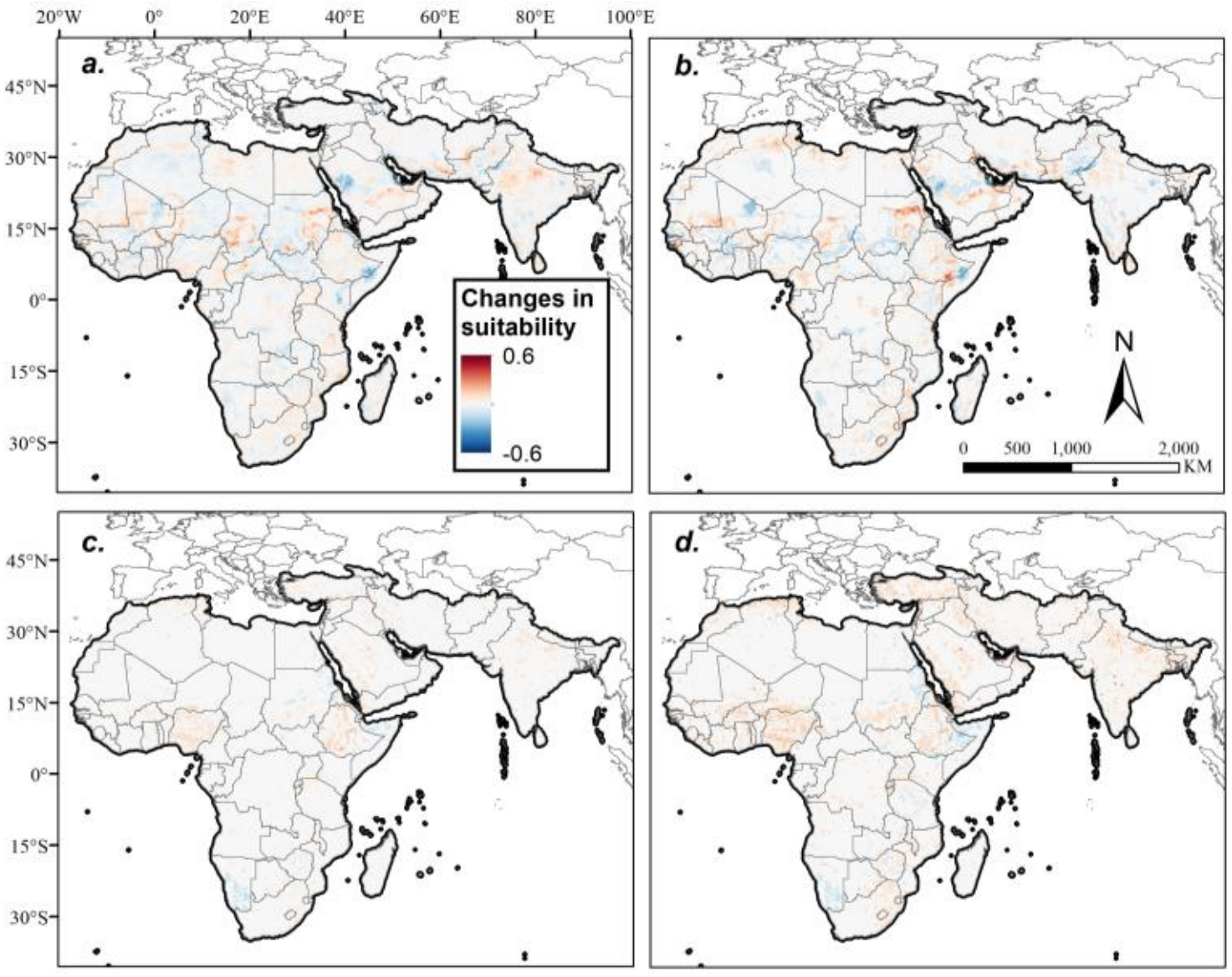

Supplementary Fig. S5. The habitat suitability of *An. stephensi* only affected by climatic factors and other land-use types. We used Phase I as baseline and kept the values of other factors in Phase II and III consistent with Phase I to estimate the habitat suitability only affected by climatic factors (a and b) or other land-use types (c and d). The changes in habitat suitability in Phase II and III were the differences in values between Phase II and III and baseline, respectively.

Final, we analyzed the mechanism by which urbanization affects *An. stephensi* through the PLS-PM model, specifically exploring how share of urban land and population indicators influence the habitat suitability, which explained quantitatively the model's output mentioned by the reviewer. We found that urbanization affecting the habitat suitability was mainly through construction activities. Furthermore, we also discussed it in the 'Discussion' section and the text in Line 330-359 is as follows.

"Urbanization has created more breeding sites for *An. stephensi*. Urban environments are suitable for breeding of *An. stephensi*, which shows preferential breeding in man-made conditions, such as overhand tank[25], artificial water containers, construction pits[26], condensation from air conditioning, open oil drums and water-filled tires after a rainfall[21]. The study further quantitatively revealed the mechanisms of urbanization on the habitat suitability of *An. stephensi* (Fig. 5). We used infrastructure investment and build-up areas to represent the construction activities and found urbanization affected the habitat suitability was mainly through construction activities. Construction booms appear to create more breeding sites for *An. stephensi*[27]. The expansion of urban areas and the growth in population resulted in the need for more buildings, and it then increased the habitat suitability of *An. stephensi*. Artificial water containers prevalent in construction sites, including concrete water tanks and car washing facilities, have become breeding habitats for *An. stephensi*[21]. Although the study did not have construction sites data, infrastructure investment and build-up areas were closely associated with construction sites because all construction projects required construction sites during development. Therefore, it revealed that increased construction due to urban expansion and population growth added more acritical

water storage containers, which might create new larval sites for *An. stephensi*.

The proportion of people living in slums in cities would increase the habitat suitability of *An. stephensi*. The study also indicated that *An. stephensi* appeared in urban slums in Chennai, India[28]. However, we found that an increase in urban land would reduce the proportion of people living in slums in cities, thereby decreasing the habitat suitability through this pathway in the overall study area (Fig. 5a). From this perspective, urbanization is beneficial for reducing the habitat suitability of *An. stephensi*. Due to the relatively minor path effect being offset by the paths through construction activities, overall urbanization still increased the habitat suitability. Compared with previous investigation studies[21,25], our study incorporated quantitative analysis and revealed the mechanisms by which urbanization affected the habitat suitability on a large scale. It can provide scientific reference for better predicting the distribution of *An. stephensi* and design strategies to response to malaria transmission in the future."

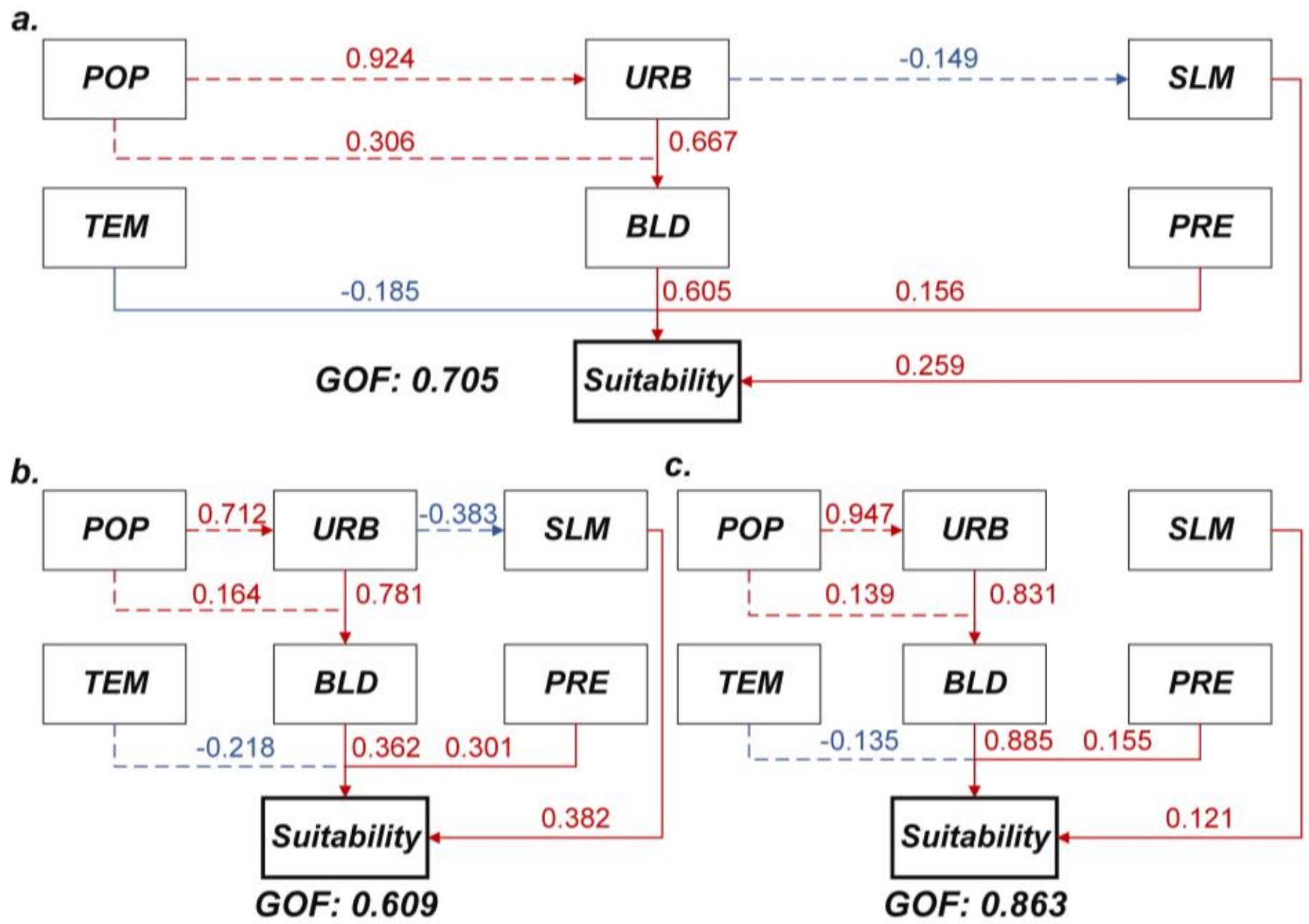

Fig. 5 The mechanisms of factors on habitat suitability of *An. stephensi*. a the results of the PLS-PM model including all data in the study area. **b and c** the results of the model only including data in Africa, and South and West Asia, respectively. The blue line is the negative relationship between factors and habitat suitability and the red line is the positive relationship between factors and habitat suitability. The solid line represents the direct influence of factors on habitat suitability and the dash line represents the indirect effect of factors on habitat suitability. The path coefficient in the panel is significant ($p < 0.05$). Each box represents a latent variable, and the specific explicit variables included in each of the three models are shown in Table S4. GOF: goodness-of-fit. POP: population. URB: share of urban land. SLM: people living in slums in cities. BLD: construction indexes. TEM: temperature indexes. PRE: precipitation indexes. Suitability: the habitat suitability of *An. stephensi*.

【Question 6】

There is a lack of necessary detail throughout – for example, what are the three phases? Why were they selected?

【Response 6】

We apologized for the lack of necessary details in the original manuscript. We added the details about the important methods used in the study. For the three phases, we considered two main reasons. First, since the previous study indicated that *An. stephensi* invaded Africa since 2012, we used 2012 as a temporal cutoff point. Second, considering data availability, our study period was selected as 1992-2021. To maintain consistent temporal intervals, we divided the period into ten-year segments. We then finally divided the study period into three phases: 1992-2001 (Phase I), 2002-2011 (Phase II) and 2012-2021 (Phase III) based on the previous study (Glidden et al., 2024). We have also revised it and add the details into the main text and supplementary materials. The text in Line 412-414 in the revision is as followed.

"We divided the period into three phases based on the invasion of *An. stephensi* and the temporal structure of key spatial datasets: 1992-2001 (Phase I), 2002-2011 (Phase II) and 2012-2021 (Phase III) (please see supplementary method 1 for details)."

The text in the 'Supplementary Method 1. Rationale for temporal stratification' is as follows.

"To examine how urbanization-related processes influence the habitat suitability of *An. stephens*i, we stratified the study period into three successive phases. This stratification reflects both the staged of invasion of *An. stephensi* and the temporal structure of key spatial datasets used in the study. Urbanization has been documented as a complicated process[1], for example, absolute population growth in the core city and thus the agglomeration as a whole and absolute population growth in the agglomeration, with growth being relatively concentrated on the suburban space[2]. These phases differ not only in the extent of urban land cover and population concentration, but also in the intensity of the built environment, which are relevant to mosquito breeding habitats. Therefore, we have divided the entire research phase into distinct stages. Due to the invasion of *An. stephensi* into Africa since 2012[3], we used 2012 as a dividing point in time series. On the other hand, the selected time windows align with the temporal resolution and continuity of datasets in the study. The previous study[4] used 10-year intervals as dividing points, so we followed this approach and divided the time periods of the study into three phases (Phase I [1992-2001], Phase II [2002-2011] and Phase III [2012-2021])."

For the classification of rural, urban and high-density urban, we have also added these details in the manuscript and supplementary materials, and the details about the revision on the classification of rural, urban and high-density urban are illustrated in 'Response 7'.

In summary, we have added the necessary details into the manuscript, which can increase the readability of the manuscript.

【References】

Glidden C K, Singleton A L, Chamberlin A. Climate and urbanization drive changes in the habitat suitability of Schistosoma mansoni competent snails in Brazil[J]. Nature Communications. 2024, 15(1): 4838.

【Question 7】

However, what may be interesting to explore is the authors' work to establish more effective urban/peri-urban, rural covariates. Distinguishing between these categories is notoriously tricky, and often not done very well. A surface that distinguishes well between these categories, includes social impacts and infrastructure, could potentially be extremely useful for future SDMs for multiple species, especially if there was some effort to ground truth estimates.

【Response 7】

We sincerely thank the reviewer for this essential comment. We agree that it is critical to distinguish well between rural and urban in a surface, including social impacts and infrastructure, which could potentially be extremely useful for future SDMs for multiple species, especially if there was more effort to ground truth estimates. Currently, many experts in remote sensing or landscape ecology are also attempting to use remote sensing, population, and other data to precisely delineate urban and rural areas. However, our research focus is on analyzing vector organisms and vector-borne diseases, and we are not proficient in identifying urban areas using specialized method. Meanwhile, the objective of the study is to elucidate the mechanisms by which urbanization affects the habitat suitability of *An. stephensi*, not to investigate a novel classification method for rural and urban areas. Therefore, we have chose to utilize more authoritative data for our study.

We used the settlement typologies delineated and classified from European Commission (Schiavina et al., 2025), which is "GHS-WUP DEGURBA settlement layers (GHS_WUP_DEGURBA_GLOBE_R2025A)". The dataset defined the spatial entities at two hierarchical levels and the 1km^2 grids are divided into three categories of the first hierarchical level: rural, urban cluster and urban center grid cell based on cell clusters population size, population and built-up area (European Commission, 2021). Specifically, an urban center is defined as a contiguous cluster of grid cells (based on 4-connectivity) where the permanent land has a population density of at least 1,500 inhabitants per km^2. This cluster should contain a total of 50,000 or more inhabitants. Its boundaries are smoothed using 3*3 conditional majority filtering, and any holes smaller than 15 km^2 within the cluster are filled. An urban cluster is formed by contiguous grid cells connected through 8-neighbor adjacency. It should have a density of at least 300 inhabitants per km^2 on permanent land and contain a total

population of 5,000 or more. The rural grid cells are all the other cells that do not belong to an urban cluster, most of which will have a density below 300 inhabitants per km^2. The second hierarchical levels contain eight categories: Urban center grid cell, dense urban cluster grid cell, semi-dense urban cluster grid cell, suburban or peri-urban grid cell, rural cluster grid cell, low density grid cell, very low density rural grid cell and water grid cell. We merged the secondary categories to derive three classifications (rural, urban and high-density urban) for analysis. Table S3 shows the introduction of categories in the first and second hierarchical levels.

The dataset generated the settlement classification layers every five years since 1975 (Schiavina et al., 2025), which the resolution is 1km. To standardize the resolution, we resampled the grids to achieve a 2.5-minute resolution by using the nearest-neighbor method. Due to the several sub-levels of three categories in the data, we further consolidated it into three major categories: rural, urban and high-density urban for analysis. Furthermore, we selected the data in 2000 for Phase I, the data in 2010 for Phase II and the data in 2020 for Phase III. The details about the classification methods and the approaches to verify the validation of this dataset are illustrated in the supplementary materials.

We also validated the settlement of the dataset. We calculated the share of urban areas across the three classifications. We found that the highest average share of urban areas was in high-density urban areas (5.188% [95%CI: 5.004%-5.373%] in Phase I, 5.832% [95%CI: 5.657-6.007%] in Phase III and 6.512% [95%CI: 6.345-6.680%] in Phase III). The second highest average share of urban land was in the urban areas (1.196% [95%CI: 1.172-1.220%] in Phase I, 1.305% [95%CI: 1.283-1.328%] in Phase II and 1.472% [95%CI: 1.451-1.493%] in Phase III). The rural areas had the lowest average share of urban areas (0.127% [95%CI: 0.126-0.128%] in Phase I, 0.161% [95%CI: 0.160-0.162%] in Phase II and 0.205% [95%CI: 0.204-0.206%] in Phase III). The share of urban areas in rural, urban and high-density urban areas in the three phases is shown in Fig. S11. It demonstrates that there is a relationship between urban area and this classification, and also validates the rationality of the classification dataset.

We have revised it and added the details into the manuscript and supplementary materials. The text in Line 500-513 in the revision is as follows.

"We used the settlement typologies delineated and classified from European Commission[55] to analyze the changes in the suitable habitat of *An. stephensi* during urbanization. The dataset defined the spatial entities at two hierarchical levels and the $1km^2$ grids are divided into three categories of the first hierarchical level: rural, urban cluster and urban center grid cell (please see supplementary methods and Table S3 for details)[56], and generated the settlement classification layers every five years since 1975[55], which the resolution is 1km. To standardize the resolution, we resampled the grids to achieve a 2.5-minute resolution by using the nearest-neighbor method. Due to the several sub-levels of three categories in the data, we further consolidated it into three major categories: rural, urban and high-density urban for analysis. Furthermore, we selected the data in 2000 for Phase I, the data in 2010 for Phase II and the data in

2020 for Phase III. The details about the classification methods and the approaches to verify the validation of this dataset are illustrated in the supplementary materials (Fig. S11). The grid-scale classification is shown in Fig. S12."

The text in supplementary materials is as followed.

"Supplementary Method 2. The classification method of urbanization in spatial raster datasets

The classification dataset of urbanization is the "GHS-WUP DEGURBA settlement layers (GHS_WUP_DEGURBA_GLOBE_R2025A)" from the previous study[5]. The dataset used cell clusters population size, population and built-up area to delineate settlements[6]. The dataset defined the spatial entities at two hierarchical levels and the 1km^2 grids are divided into three categories of the first hierarchical level: rural, urban cluster and urban center grid cell[6]. Specifically, an urban center is defined as a contiguous cluster of grid cells (based on 4-connectivity) where the permanent land has a population density of at least 1,500 inhabitants per km^2. This cluster should contain a total of 50,000 or more inhabitants. Its boundaries are smoothed using 3*3 conditional majority filtering, and any holes smaller than 15 km^2 within the cluster are filled. An urban cluster is formed by contiguous grid cells connected through 8-neighbor adjacency. It should have a density of at least 300 inhabitants per km2 on permanent land and contain a total population of 5,000 or more. The rural grid cells are all the other cells that do not belong to an urban cluster, most of which will have a density below 300 inhabitants per km^2. The second hierarchical levels contain eight categories: Urban center grid cell, dense urban cluster grid cell, semi-dense urban cluster grid cell, suburban or peri-urban grid cell, rural cluster grid cell, low density grid cell, very low density rural grid cell and water grid cell. We merged the secondary categories to derive three classifications (rural, urban and high-density urban) for analysis. Table S3 shows the introduction of categories in the first and second hierarchical levels.

We also validated the settlement of the dataset. We calculated the share of urban areas across the three classifications. We found that the highest average share of urban areas was in high-density urban areas (5.188% [95%CI: 5.004%-5.373%] in Phase I, 5.832% [95%CI: 5.657-6.007%] in Phase III and 6.512% [95%CI: 6.345-6.680%] in Phase III). The second highest average share of urban land was in the urban areas (1.196% [95%CI: 1.172-1.220%] in Phase I, 1.305% [95%CI: 1.283-1.328%] in Phase II and 1.472% [95%CI: 1.451-1.493%] in Phase III). The rural areas had the lowest average share of urban areas (0.127% [95%CI: 0.126-0.128%] in Phase I, 0.161% [95%CI: 0.160-0.162%] in Phase II and 0.205% [95%CI: 0.204-0.206%] in Phase III). The share of urban areas in rural, urban and high-density urban regions in the three phases is shown in Fig. S11. It demonstrates that there is a relationship between urban area and this classification, and also validates the rationality of the classification dataset."

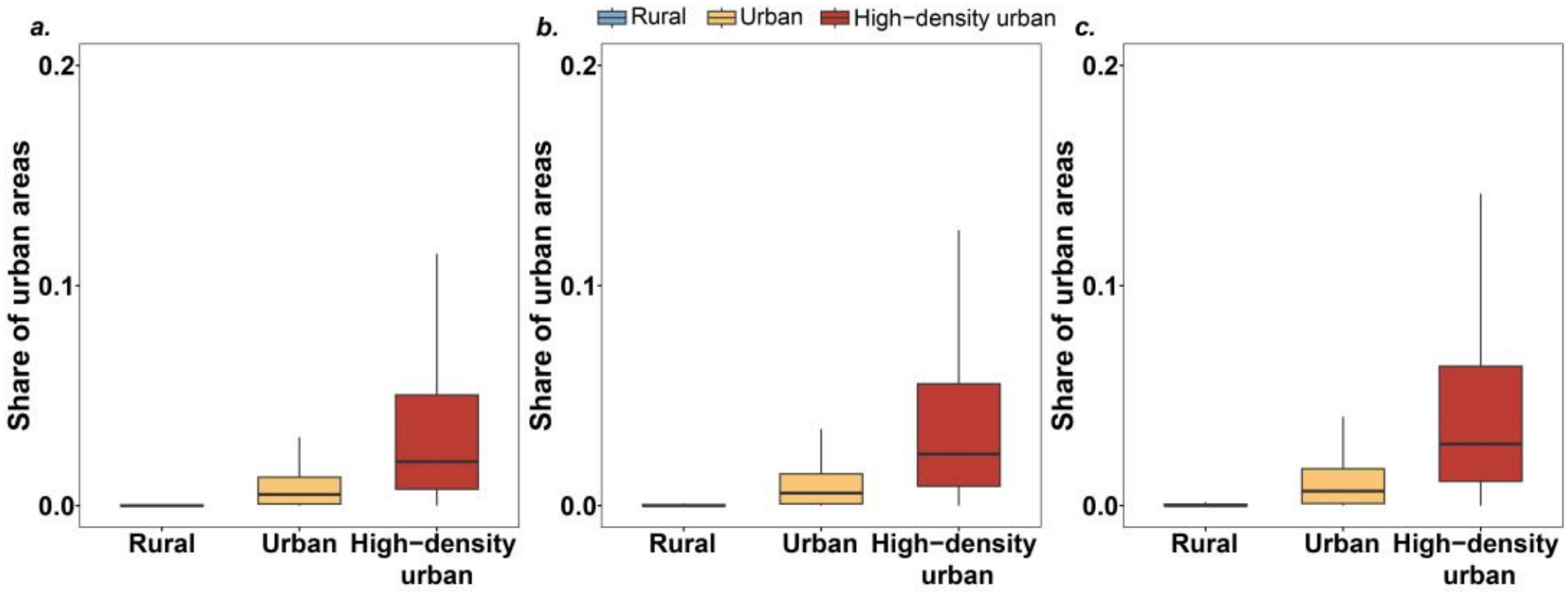

Supplementary Fig. S11. The share of urban areas in rural, urban and high-density urban in the three phases. The classification was obtained from the classification dataset [1]. a Phase I. b Phase II. c Phase III.

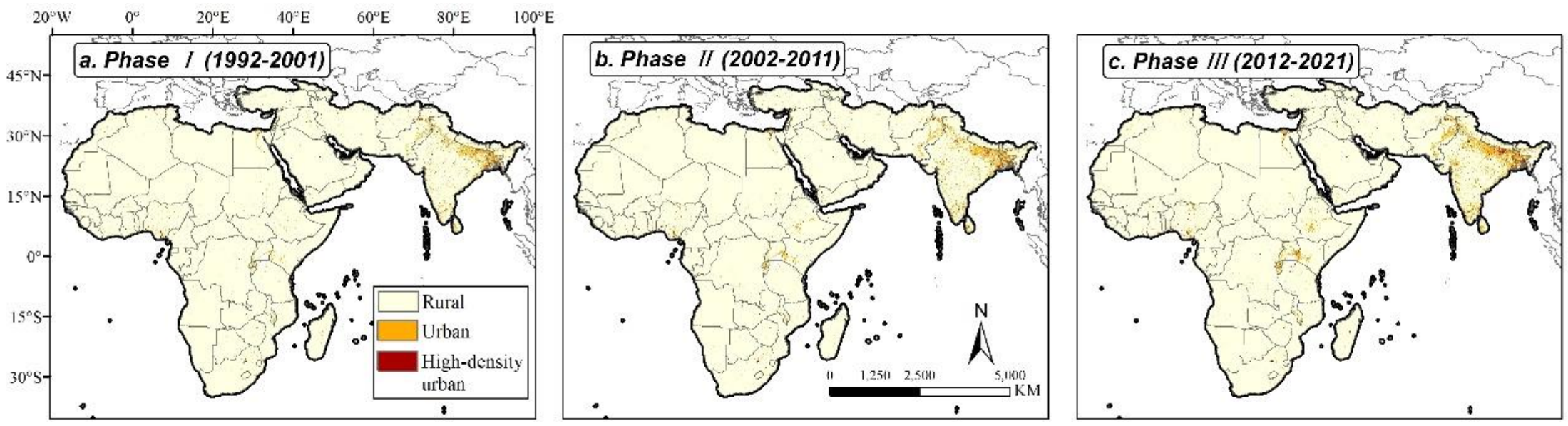

Supplementary Fig. S12. The spatial distribution of rural, urban and high-density urban in the three phases. The details about classification method are stated in 'Method' Part in the manuscript[1]**.** a Phase I. b Phase II. c Phase III.

Supplementary Table S3. The classification of rural, urban and high-density urban. The dataset is obtained from the previous study[6].

Class in original data	Sub-level class in original data (Code)	Definition	Class in the study
Urban center	Urban center grid cell (30)	An urban center consists of contiguous grid cells (4-connectivity cluster) with a density of at least 1,500 inhabitants per km^2 of permanent land, and has at least 50,000 inhabitants in the cluster with smoothed boundaries (3-by-3 conditional majority filtering12) and <15 km^2 holes filled.	High-density urban
Urban cluster	Dense urban cluster grid cell (23)	A Dense Urban Cluster consists of contiguous cells (4-connectivity cluster) with a density of at least 1,500 in habitants per km2 of permanent land and has at least 5,000 and less than 50,000 in habitants in the cluster.	Urban
	Semi-dense urban cluster grid cell (22)	A semi-dense urban cluster consists of contiguous (using	

		four-point contiguity) grid cells within a urban cluster (level 1) at least 2 km away13 from a dense urban cluster or an urban center with a density of at least 900 inhabitants per km2 and a collective population of at least 2,500 people	
	Suburban or peri-urban grid cell (21)	All the other cells that belong to an Urban Cluster but are not part of an Urban Centre, Dense Urban Cluster or a Semi-dense Urban Cluster	
Rural	Rural cluster grid cell (13)	A Rural Cluster consists of contiguous cells (8-connectivity cluster) with a density of at least 300 inhabit ants per km^2 of permanent land and has at least 500 and less than 5,000 inhabitants in the cluster.	Rural
	Low density rural grid cell (12)	Rural grid cells with a density of at least 50 inhabitants per km2 of permanent land and are not part of a Rural Cluster.	
	Very low density rural grid cell (11)	Cells with a density of less than 50 inhabitants per km2 of permanent land.	
Water grid cells	Water grid cell (10)	All the cells with more than 0.5 share covered by permanent surface water that are not populated nor built.	Other

【References】

European Commission, A. S. O. O. Applying the Degree of Urbanisation - A methodological manual to define cities, towns and rural areas for international comparisons. 2021 Edition Publications Office of the European Union, (2021).

Schiavina, M., Melchiorri, M., Pesaresi, M., Jacobs-Crisioni, C. & Dijkstra, L. GHS-WUP-DEGURBA R2025A – GHS-WUP DEGURBA settlement layers, application of the Degree of Urbanisation methodology (stage I) to GHS-WUP-POP R2025A, multitemporal (1975-2100). (2025).

【Question 8】

As it stands, I don't think this paper is ready for publication – I would suggest that it does provide a really interesting and quite comprehensive look at changes in urbanization across several regions – but the assumption is that urbanization = *An.*

stephensi habitat. This is not entirely the case – there are lots of nuances in which larval sites this species will use. I would suggest that the paper would be more of use if references to the mosquito were removed! and the focus was just on how these environments have changed – with the discussion including potential implications for mosquito species that are able to inhabit this type of habitats (not just *An. stephensi*) as well as other impacts.

【Response 8】

We sincerely thank the reviewer for this essential critique. The study aims to analyze the mechanisms by which urbanization affects the habitat suitability of *An. stephensi*. In fact, the study did not assume that urbanization = *An. stephensi* habitat. We also agree that the relationship between urbanization and *An. stephensi* is complex, which is one of our important aims to explore. We found that urbanization affecting the habitat suitability was mainly through construction activities. Although previous studies have also identified that *An. stephensi* is adaptable to the artificial environments through field surveys, the study further demonstrated this finding at a larger scale through mathematical modelling. Moreover, the urbanization indicators identified in the study provide scientific reference for predicting the potential distribution of *An. stephensi*'s habitat suitability and malaria risks in the future. It can also serve as a methodological reference for habitat suitability analysis of other species. On the other hand, we also agree that how these environments have change and how mosquito species that are able to inhabit this type of habitats under this scenario is an important and valuable problem to analyze. In fact, the study also demonstrated how urbanization changes affected the habitat of *An. stephensi*. Therefore, removing the mosquito may defeat the purpose of the study, and in the future, we will conduct the study about the relationships between these environments changed and the mosquito species.

We tried our best to improve the manuscript and highlight all changes in the tracked-changes version of the manuscript. We appreciate for Reviewer's warm work earnestly, and hope the correction will meet with approval. Once again, thank you very much for your comments and suggestions.

Reviewer #2

I appreciate the opportunity to review this article and would like to commend the authors for their diligent work in developing a well-structured model analysis. The study effectively investigates the influence of population and urbanization on the habitat suitability of Anopheles stephensi. The authors reported an increase in habitat suitability within areas undergoing rural-to-urban transition. The study's aims and methodological framework are well-conceived and thoughtfully executed. I offer the comment below that may further refine the manuscript and enhance its suitability for publication.

We thank Reviewer#2 for time spent in carefully reviewing our manuscript and the positive assessment of it. We answered all the concerns in comprehensive manner. Please see below and the revised version.

【Question 1】

It would be helpful for the authors to clarify whether the occurrence dataset of *Anopheles stephensi*, as well as the environmental variables at both occurrence and pseudo-absence locations, followed a normal distribution. Given that Pearson's correlation assumes normality and linear relationships, the choice of this method over Spearman's rank correlation, particularly if the data were non-normally distributed, requires further justification (Result section - page 3).

【Response 1】

Thank you for your valuable comment. We first assumed that the data followed a normal distribution. Using the Shapiro-Wilk normality test, we found that the significant level (p value) for all 25 variables across 10 datasets (combined with occurrence points and background points) is less than 0.001 ($p<0.001$). This indicated rejection of the null hypothesis, confirming that the data did not satisfy a normal distribution. Next, we conducted a visual assessment using a Q-Q plot. Figure S11 displays the Q-Q plot for 25 variables from one dataset. It shows that variables are not uniformly distributed on either side of the reference line, further confirming the non-normal distribution of the data. Therefore, Spearman's correlation was applied to assess multicollinearity among the variables. We have also added an explanation in the revision (Line 520-528) and the text is as followed.

"Before constructing the BRT model, we first examined multicollinearity among the 25 variables in 10 datasets. Initially, we assumed the data followed a normal distribution. The Shapiro-Wilk normality test revealed significance levels (p value) consistently below 0.001, indicating rejection of the null hypothesis. We then used Q-Q plots and found that the points were not uniformly distributed on either side of the reference line, further confirming the non-normal distribution (Fig. S13). Therefore,

Spearman's correlation was applied to assess multicollinearity among variables and the results are presented in Fig. S1. Variables with correlation coefficients exceeding 0.75 were excluded, ultimately containing 15 variables into the BRT model."

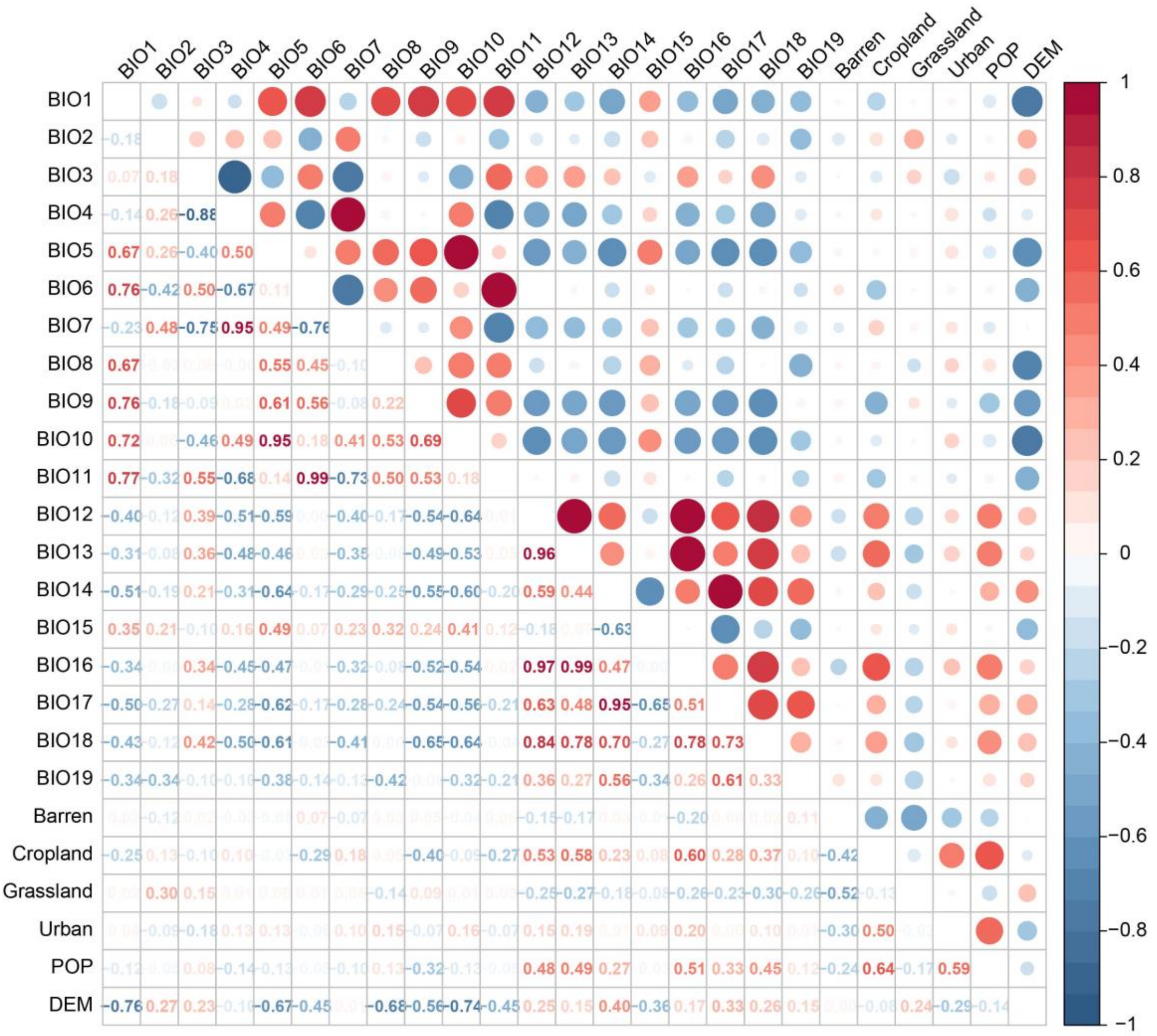

Supplementary Fig. S1. The Spearman's correlations between factors. We final selected 14 factors into BRT model, including BIO1, BIO2, BIO4, BIO8, BIO10, BIO13, BIO15, BIO17, BIO19, barren, cropland, grassland, urban and population. Meanwhile, we did not take the share of forest into account since the share of forest at all *An. stephensi* occurrence points were similar. BIO1: annual mean temperature. BIO2: mean monthly temperature range. BIO3: is thermality (BIO2/BIO7). BIO4: temperature seasonality. BIO5: max temperature of warmest month. BIO6: min temperature of coldest month. BIO7: Temperature annual range. BIO8: mean temperature of wettest quarter. BIO9: mean temperature of driest quarter. BIO10: mean temperature of warmest quarter. BIO11: mean temperature of coldest quarter. BIO12: annual precipitation. BIO13: precipitation of wettest month. BIO14: precipitation of driest month. BIO15: precipitation seasonality. BIO16: precipitation of wettest quarter. BIO17: precipitation of driest quarter. BIO18: precipitation of warmest quarter. BIO19: precipitation of coldest quarter. Barren: share of barren. Cropland: share of cropland. Grassland: share of grassland. Urban: share of urban land. POP: population. DEM: elevation.

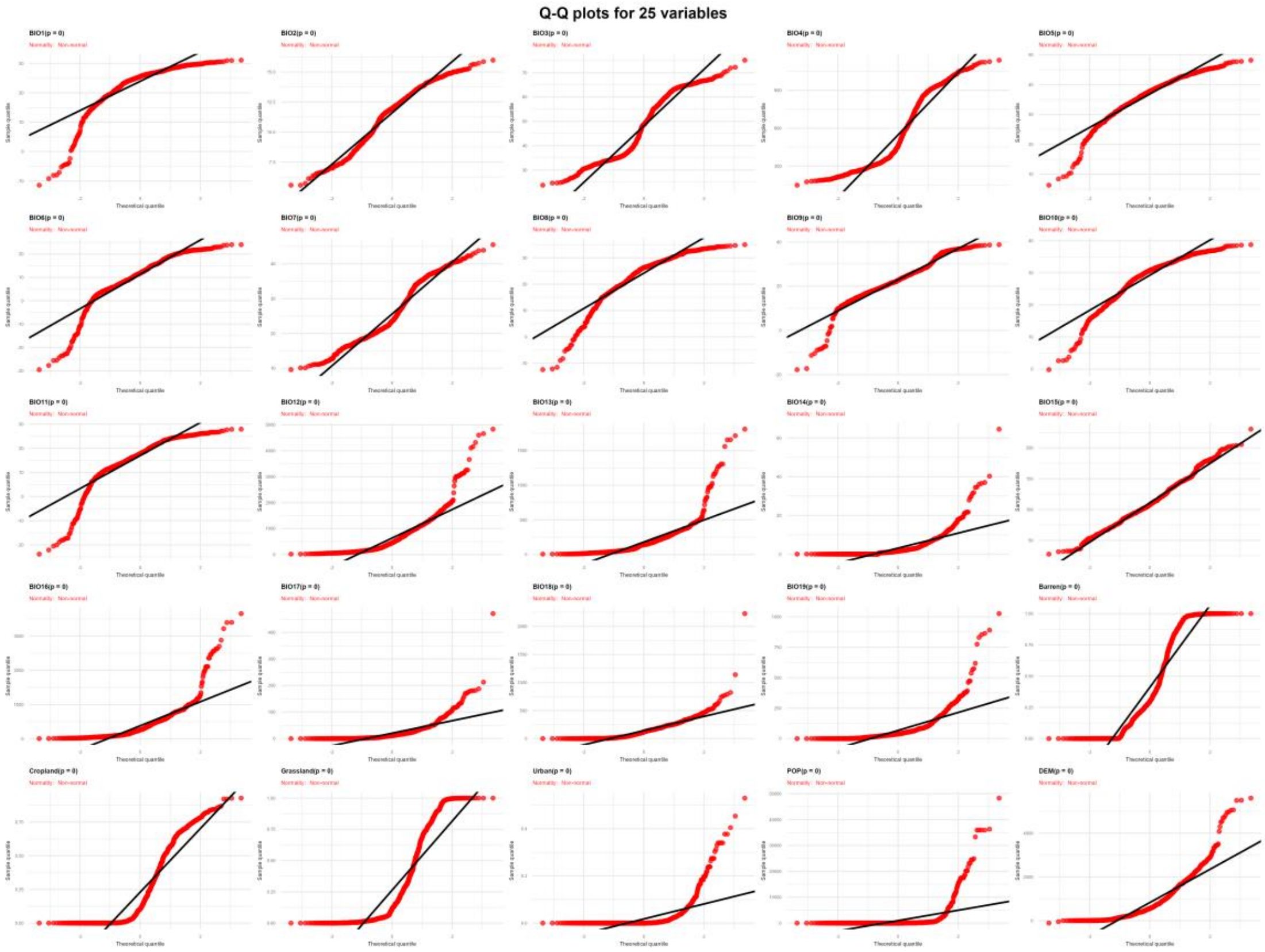

Supplementary Fig. S13. The Q-Q plots of 25 variables in one of the ten datasets. The red dots represent the ratio of theoretical quantiles to sample quantiles. The solid black line serves as a reference, depicting the theoretical normal distribution curve. The chart displays variable names, with the p value for the Shapiro-Wilk normality test. If p>0.05, it indicates accept the null hypothesis and the data is normal distribution, otherwise, it is not. BIO1: annual mean temperature. BIO2: mean monthly temperature range. BIO3: is thermality (BIO2/BIO7). BIO4: temperature seasonality. BIO5: max temperature of warmest month. BIO6: min temperature of coldest month. BIO7: Temperature annual range. BIO8: mean temperature of wettest quarter. BIO9: mean temperature of driest quarter. BIO10: mean temperature of warmest quarter. BIO11: mean temperature of coldest quarter. BIO12: annual precipitation. BIO13: precipitation of wettest month. BIO14: precipitation of driest month. BIO15: precipitation seasonality. BIO16: precipitation of wettest quarter. BIO17: precipitation of driest quarter. BIO18: precipitation of warmest quarter. BIO19: precipitation of coldest quarter. Barren: share of barren. Cropland: share of cropland. Grassland: share of grassland. Urban: share of urban land. POP: population. DEM: elevation.

Finally, thanks again for reviewing our paper and for providing your useful suggestions.

Reviewer #3

This study evaluates the effects of urbanization on habitat suitability for a malaria vector in Africa and Asia. The study is interesting and well-presented. However, I have a few comments that could improve the manuscript.

We are thankful to Reviewer #3 for the constructive comments, which help us improve the quality of the paper. Specific replies are given below.

【Question 1】

"Introduction" section – All scientific names of genera and species ought to be italicized.

【Response 1】

We were really sorry for our careless mistakes. Thank you for your reminder. We have italicized all scientific names of genera and species throughout the revision.

【Question 2】

In "Introduction" section, paragraph 1. The following sentence should be rephrased: "Prior to 2011, *An. Stephensi* was the primary malaria found mainly in South Asia and the Arabian Peninsula". Additionally, Sri Lanka is not an African country.

【Response 2】

We sincerely thank the reviewer for careful reading. We are sorry for our carelessness. As suggested by the reviewer, we have removed Sri Lanka, and the text in Line 34-35 in the revision is as followed.

"However, *An. stephensi* was invaded into Africa since 2012[2], and founded in Dijibouti (2012), Ethiopia (2016), the Republic of the Sudan (2019) and Ghana (2023)[3,4]."

【Question 3】

In "Introduction" section, paragraph 2. The following sentence should be rephrased: " (…) has substantially contribute to eliminate malaria".

【Response 3】

Thank you for your reminder. We were sorry for our careless mistakes. We have revised the sentence and the text in Line 44-46 in the revision is as follows.

“For instance, China’s experience suggests that removing the breeding sites of *Anopheles* mosquitoes, especially in the larval stage, substantially contributing to eliminate malaria[9].”

【Question 4】

Section “The contribution of factors on the habitat suitability”. Paragraph 1. These details (the first four sentences) have already been mentioned in the methodology section, so they should be removed. Here, the authors should focus on describing the main results; there is no need to repeat information or describe the methods. The multicollinearity analysis should be integrated into the BRT model computation.

【Response 4】

Thank you for your helpful comment. We have condensed the first paragraph in ‘The contribution of factors on the habitat suitability’ section. However, the Reviewer #1 suggests that due to journal formatting requirements, the results appear before the methods section. To facilitate reader comprehension, peripheral information should be provided upfront. Therefore, we have refined the original paragraph to concisely explain the peripheral information about how we conducted the model to make these results understandable. Furthermore, the multicollinearity analysis has been integrated into the BRT model computation in Line 520-528 (please see the response of Question 8). The text in Line 78-88 in the revision is as followed.

“We selected 25 climate, land use and population variables, which might be related with suitability habitat of *An. stephensi*, and the details about the variables were stated in Table S2. We contained 14 variables into booster regression tress (BRT) model through multicollinearity analysis (Fig. S1). Ten sets of background points with the same level of bias as the occurrence points were included, and each set was randomly sampled 10 times, with 80% of the data for training and 20% for testing. The process was repeated 100 times for BRT model, and the final result was the average of 100 outcomes. The models with 100 iterations performed accurately with a mean area-under-the-curve (AUC) of 0.900 (95%CI: 0.896-0.903), a mean sensitivity of 0.822 (95%CI: 0.809-0.835) and a mean specificity of 0.842 (95%CI: 0.830-0.854), showing that the model simulated accurately the habitat suitability for *An. stephensi*.”

Supplementary Table S2. The data resources in the study. The features were considered in the study. We analyzed the correlations between factors, and those with correlations greater than 0.75 were removed. The complete results of the Spearman’s correlations between factors are shown in Figure S1. The other urbanization indexes were not included in BRT model. We thus were not considered them into the Pearsons’s correlations.

Type	Factor	Definition	Spatial/temporal resolution	Source	Correlated with
Climate	BIO1	Annual mean	2.5	WorldCli	BIO6, BIO9, BIO11,

		temperature	minutes/ 10-year average	m	DEM
	BIO2	Mean monthly temperature range (Mean of monthly [max temperature – min temperature])			None
	BIO3	Is thermality (BIO2/BIO7)			BIO4, BIO7
	BIO4	Temperature seasonality (standard deviation ×100)			BIO3, BIO7
	BIO5	Max temperature of warmest month			BIO10
	BIO6	Min temperature of coldest month			BIO1, BIO7, BIO11
	BIO7	Temperature annual range (BIO5-BIO6)			BIO3, BIO4, BIO6
	BIO8	Mean temperature of wettest quarter			None
	BIO9	Mean temperature of driest quarter			BIO1
	BIO10	Mean temperature of warmest quarter			BIO5
	BIO11	Mean temperature of coldest quarter			BIO1, BIO6
	BIO12	Annual precipitation			BIO13, BIO16, BIO18
	BIO13	Precipitation of wettest month			BIO12, BIO16, BIO18
	BIO14	Precipitation of driest month			BIO17
	BIO15	Precipitation seasonality (coefficient of variation)			None
	BIO16	Precipitation of wettest quarter			BIO12, BIO13, BIO18
	BIO17	Precipitation of driest quarter			BIO14
	BIO18	Precipitation of warmest quarter			BIO12, BIO13, BIO16
	BIO19	Precipitation of coldest quarter			None
Land use	Barren	Share of barren	0.25°/10 -year average	LUH2	None
	Cropland	Share of cropland			None
	Forest	Share of forest			None
	Grassland	Share of grassland			None
	Urban	Share of urban land			None
Population	Population	Total population	1km/10-year average	LandScan	None
Elevation	Elevation	Elevation	2.5	WorldCli	BO1

			minutes/ 30-year average	m	
Settlement	Settlement	The classification in rural, urban and high-density urban	1km	European Commission	None
The build-up surface areas	The build-up surface areas	The build-up surface areas	1km	European Commission	None
Others	Infrastructure investment	Total private participation in infrastructure (PPI) investment	Country/ 10-year total	World Bank	None
	Population living in slums	Proportion of people living in slums in cities			

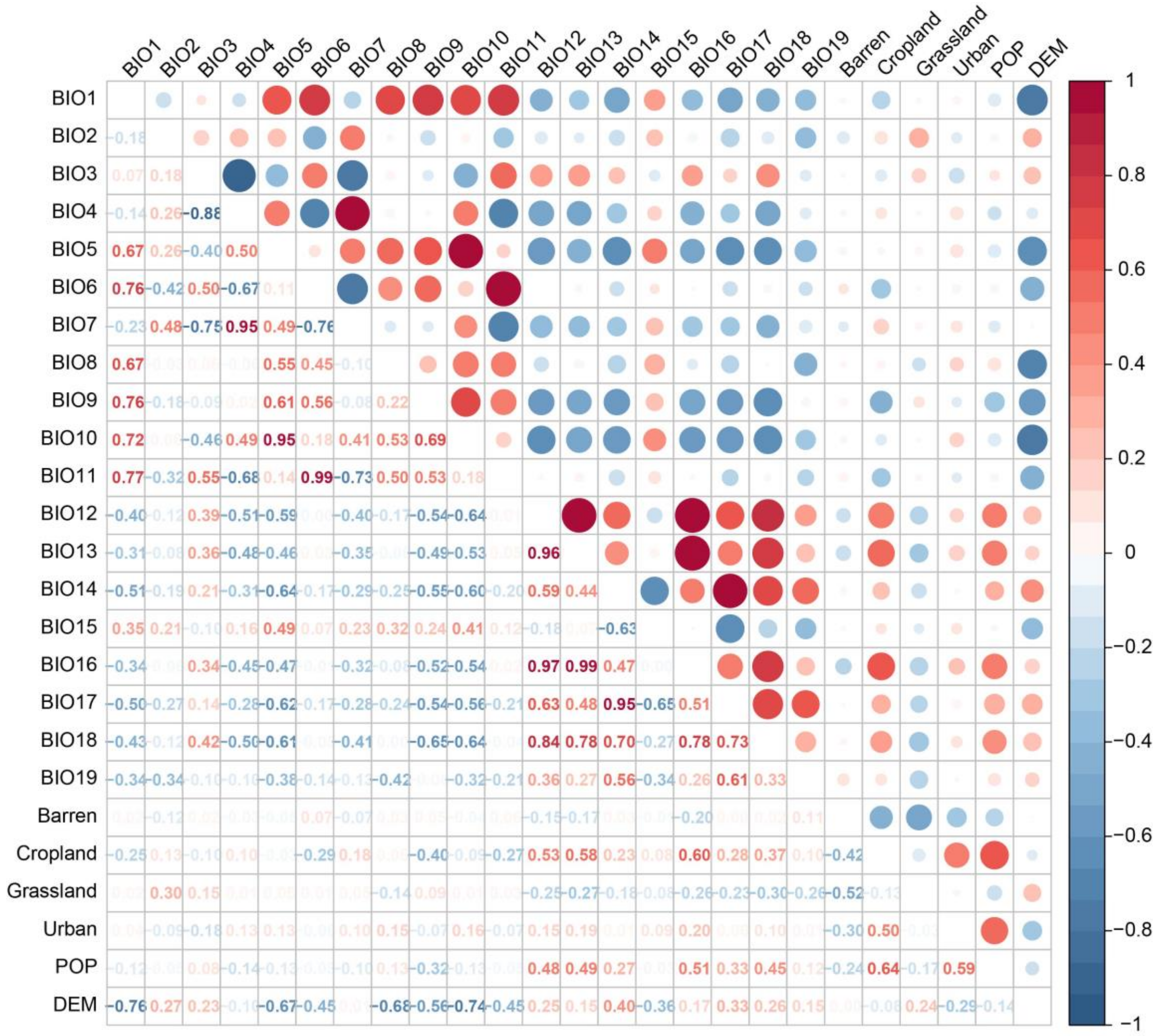

Supplementary Fig. S1. The Spearman's correlations between factors. We final selected 14 factors into BRT model, including BIO1, BIO2, BIO4, BIO8, BIO10, BIO13, BIO15, BIO17, BIO19, barren, cropland, grassland, urban and population. Meanwhile, we did not take the share of forest into account since the share of forest at all *An. stephensi* occurrence points were similar. BIO1: annual mean temperature. BIO2: mean monthly temperature range. BIO3: is thermality (BIO2/BIO7). BIO4: temperature seasonality. BIO5: max temperature of warmest month. BIO6: min temperature of coldest month. BIO7: Temperature annual range. BIO8: mean temperature of

wettest quarter. BIO9: mean temperature of driest quarter. BIO10: mean temperature of warmest quarter. BIO11: mean temperature of coldest quarter. BIO12: annual precipitation. BIO13: precipitation of wettest month. BIO14: precipitation of driest month. BIO15: precipitation seasonality. BIO16: precipitation of wettest quarter. BIO17: precipitation of driest quarter. BIO18: precipitation of warmest quarter. BIO19: precipitation of coldest quarter. Barren: share of barren. Cropland: share of cropland. Grassland: share of grassland. Urban: share of urban land. POP: population. DEM: elevation.

【Question 5】

The discussion could be improved by explaining the possible reasons behind the main scenarios observed in the study area, instead of simply repeating the description of the results.

【Response 5】

Thank the reviewer for the valuable comment. We have revised the ‘Discussion’ part and explained the possible reasons behind the main scenarios observed in the study area, instead of simply repeating the description of the results. We wrote the ‘Discussion’ section in the order of the results. First, we described the aims and content of the manuscript. The text in Line 277-288 is as follows.

“As a critical vector of malaria, the spatial distribution of *An. stephensi* have been affected by human activities and changed dramatically over the past 30 years. In the study, we analyzed the key elements affecting the habitat suitability by using mathematical models, and found that urbanization indexes, including share of urban land and population, were critical for *An. stephensi* habitat suitability. The spatial distribution of the habitat suitability showed that the increased habitat suitability was associated with urbanization, especially in Africa. Furthermore, we analyzed the mechanisms by which urbanization affected the habitat suitability. It revealed that urbanization indirectly affected the habitat suitability mainly through construction. The study quantitatively analyzed the mechanisms of urbanization on the habitat suitability of *An. stephensi*, which can provide key parameters for predicting malaria risks in the future and contribute to eliminate *An. stephensi*.”

We then discussed the possible reasons for the association between urbanization and the habitat suitability for *An. stephensi*. The text in Line 289-306 is as follows.

“Share of urban land and population within urbanization indicators are key factors influencing the habitat suitability of *An. stephensi* (Fig. 1). We selected two metrics to represent urbanization based on the previous study, including share of urban land and population[23]. Furthermore, another previous study also indicated that population played an important role in the potential distribution of *An. stephensi*[24], which is similar to our results. Meanwhile, previous studies that predicted the potential distribution of *An. stephensi* have only considered climatic factors[17,19]. The study demonstrated that urbanization indicators, including share of urban land and

population, could be included in the future studies to predict the potential distribution of *An. stephensi* more accurately. On the other hand, since BRT is a species distribution modelling (SDM) used to analyze species fully occupying the niche, and *An. stephensi* is an invasive species, we also constructed a BRT model only in Africa. Fig. S8 shows that share of urban land and population also significantly affected the habitat suitability of *An. stephensi*, consistent with the overall finding, which demonstrating the robustness of the results of the BRT model in the study. However, some factors' (i.e. share of grassland) contribution to the habitat suitability was different from the overall findings, suggesting that the ecological characteristics of *An. stephensi* might undergo changes during its invasion process. Future research is needed to explore the habitat characteristics of *An. stephensi* in Africa."

Third, we also discussed the distribution of habitat suitability for *An. stephensi*, and the text in Line 307-329 is as follows.

"Our model suggested that urbanization accelerated the changes in the habitat suitability of *An. stephensi*. The habitat suitability of *An. stephensi* increased in Africa during Phase III (Fig. 3), and its invasion into Africa since 2012[2] also illustrated it. Urbanization progressed rapidly in Phase III, and the number of becoming urban or high-density urban grids reached 2.464 times that of Phase II (Fig. S3), and the urban-to-high-density-urban regions had the largest increase in the habitat suitability (Fig. 3). Despite the reliability of the urban classification, the delineation in the boundaries between urban and rural areas is hard. Because the population in these areas may be close to the urban threshold, and some environments may be quite similar to the habitats of *An. stephensi* in the urban areas. The field researches in the future are required to conduct in-depth analysis of *An. stephensi* larval condition in these areas. However, the results did not indicate that *An. stephensi* larval sites was only found in urban areas. In fact, *An. stephensi* is also adaptive to the rural environment, such as freshwater ponds, stream beds, wells and seepage canals[25]. On the other hand, it is certain that multiple factors, including climate, urban land and population, have influenced the distribution of *An. stephensi*. For example, the habitat suitability in southern Ethiopia has increased, while there was a little urban area. This might be the result of climate effect, as the habitat suitability increased in this region under climate effects alone (Fig. S5). Our study suggested that urbanization and climate change in the future might have great impact on the distribution of *An. stephesni*, potentially enabling its invasion into other continent or countries where climatic conditions were suitable and rural and urban breeding habitat existed. Therefore, the adaptation to both urban and rural environments of *An. stephensi* needs to implement updated mosquito control methods to eliminate it."

Forth, we discussed the mechanisms by which urbanization affected the habitat suitability and indicated the implications for prevention and control of *An. stephensi* in the future. The text in Line 330-396 is as follows.

"Urbanization has created more breeding sites for *An. stephensi*. Urban environments are suitable for breeding of *An. stephensi*, which shows preferential breeding in man-

made conditions, such as overhand tank[25], artificial water containers, construction pits[26], condensation from air conditioning, open oil drums and water-filled tires after a rainfall[21]. The study further quantitatively revealed the mechanisms of urbanization on the habitat suitability of *An. stephensi* (Fig. 5). We used infrastructure investment and build-up areas to represent the construction activities and found urbanization affected the habitat suitability was mainly through construction activities. Construction booms appear to create more breeding sites for *An. stephensi*[27]. The expansion of urban areas and the growth in population resulted in the need for more buildings, and it then increased the habitat suitability of *An. stephensi*. Artificial water containers prevalent in construction sites, including concrete water tanks and car washing facilities, have become breeding habitats for *An. stephensi*[21]. Although the study did not have construction sites data, infrastructure investment and build-up areas were closely associated with construction sites because all construction projects required construction sites during development. Therefore, it revealed that increased construction due to urban expansion and population growth added more acritical water storage containers, which might create new larval sites for *An. stephensi*.

The proportion of people living in slums in cities would increase the habitat suitability of *An. stephensi*. The study also indicated that *An. stephensi* appeared in urban slums in Chennai, India[28]. However, we found that an increase in urban land would reduce the proportion of people living in slums in cities, thereby decreasing the habitat suitability through this pathway in the overall study area (Fig. 5a). From this perspective, urbanization is beneficial for reducing the habitat suitability of *An. stephensi*. Due to the relatively minor path effect being offset by the paths through construction activities, overall urbanization still increased the habitat suitability. Compared with previous investigation studies[21,25], our study incorporated quantitative analysis and revealed the mechanisms by which urbanization affected the habitat suitability on a large scale. It can provide scientific reference for better predicting the distribution of *An. stephensi* and design strategies to response to malaria transmission in the future.

Africa is one of the regions with the fastest urbanization globally[29], and is facing unprecedented population growth in the 21st century, much of which will be absorbed by urban areas, with the total urban population projected to triple from current levels[30]. Malaria in Africa has been commonly considered a rural transmission disease[31]. However, we found that urbanization increased the habitat suitability in Africa (Fig. 3d and Fig. 4i-l), which might pose a malaria risks to African cities. The previous study also indicated that the African cities face challenges to malaria control, mainly due to unplanned urbanization and transmission of *An. stephensi*[32]. In fact, there have been multiple malaria outbreaks caused by the invasion of *An. stephensi* in Africa[33,34]. For example, spatial aggregation of *Plasmodium falciparum* infection around malaria patients in Ethiopia was associated with *An. stephensi* in the vicinity of households under a prospective case-control design experiment[33]. Furthermore, urbanization also affected the habitat suitability was mainly through construction activities (Fig. 5b). The study in Ethiopia also demonstrated that mass urban migration and unprecedented

construction booms across African cities were likely facilitating the expansion of *An. stephensi*, creating new larval habitats[35]. Although the differences in behaviors of *An. stephensi* between Africa, and South and West Asia are not included in the study, it is certain that *An. stephensi,* which adapt to both rural and urban environments, may add a new threat to malaria transmission in Africa. It needs to apply targeted mosquito control technologies and strengthen surveillance to address malaria transmission in Africa.

An. stephensi was the malaria vector mainly in South Asia and the Arabian Peninsula before 2011[2]. Many studies in Asia confirmed that *An. stephensi* was transmitted malaria mainly in cities[36,37], for example, the study indicated that *An. stephensi* was a major urban malaria vector in India[37]. Our study also indicated that the habitat suitability was high in urban and high-density urban areas in South and West Asia (Fig. 2). The changes in habitat suitability in South Asia and West Asia were relatively lower than that in Africa, and even decreased in some areas (Fig. 3). Single-factor analysis indicated that this decline in some areas might be climate-driven. For instance, the reduction in the habitat suitability in southeastern India coincided with a climate-driven decrease in habitat suitability (Fig. 3 and Fig. S5). Furthermore, urbanization also primarily affected the habitat suitability through construction activities. However, urbanization could increase the proportion of people living in slums in cites, which differed from the results in the overall study area and Africa (Fig. 5c). Urbanization in Asia was progressing faster than in Africa[38], which may lead to regional development imbalances, resulting in an increase in slums. Therefore, urban planning should be prioritized during urbanization to reduce *An. stephensi* breeding sites."

The final part is the limitation in the study, and the text in Line 397-408 is as follows.

"There are three limitations in the study. First, it is recognized that a perfect and unbiased sampling regime is challenging. While our methodological approach aims to mitigate this known data bias, some inherent biases associated with the original occurrence data may persist. Future study incorporating systematic field surveillance across diverse environments would be critical. Second, due to differences in data precision, we used resampling to standardize the data to a resolution of 2.5 minutes. This could potentially lead to a loss of information in the precise data. Meanwhile, due to the limited temporal updates of data, we adopted multiple approaches to unify the time scale, which may also have a slight impact on the results. Finally, the study hypothesized that *An. stephensi* behaviors in Africa, South Asia and West Asia were identical, but it is possible that they actually exhibit certain differences. In-depth research is needed to analyze it in the future."

【Question 6】

What algorithm did you use to interpolate the data?

【Response 6】

We apologize for the lack details about the interpolating the data. The method we used to interpolating the data was based on the previous study (Glidden et al., 2024). We assumed linear population growth to construct the linear function based on data from 2000-2021 to interpolate the population data for 1992-1999. We revised this part and the text in Line 486-488 in the revision is as follows.

"Since LandScan only provided data for 2000-2022, we assumed linear population growth to construct the linear algorithm to interpolate the data for 1992-1999 based on the previous study[46]."

【References】

Glidden C K, Singleton A L, Chamberlin A. Climate and urbanization drive changes in the habitat suitability of Schistosoma mansoni competent snails in Brazil[J]. Nature Communications. 2024, 15(1): 4838.

【Question 7】

Section "Identifying rural, urban and high-density urban regions". It is unclear how the land use maps (e.g., barren, grassland, cropland) were used to classify the area into rural, urban and high-density regions. Moreover, the criteria used to classify rural areas should be clarified.

【Response 7】

Thank you for your valuable comment and we feel sorry for the lack of clarity in our previous explanation of the classification method. Since the objective of our study is not to propose a classification method for rural, urban and high-density urban areas, we utilized existing research data on classification of rural and urban for analysis in the revised manuscript. Since this classification will directly affect our findings, adopting authoritative data may enhance the credibility of the results. In this classification method, it defined the spatial entities at two hierarchical levels and the $1km^2$ grids are divided into three categories of the first hierarchical level: rural, urban cluster and urban center grid cell. For example, an urban center is defined as a contiguous cluster of grid cells (based on 4-connectivity) where the permanent land has a population density of at least 1,500 inhabitants per km^2. This cluster should contain a total of 50,000 or more inhabitants. Its boundaries are smoothed using 3*3 conditional majority filtering, and any holes smaller than 15 km^2 within the cluster are filled. The second hierarchical level has eight categories. The details about the classification method are shown in Table S3. We calculated the share of urban areas across the three classifications to validate the classification method.. We found that the highest average share of urban areas was in high-density urban areas (5.188% [95%CI: 5.004%-5.373%] in Phase I, 5.832% [95%CI: 5.657-6.007%] in Phase III

and 6.512% [95%CI: 6.345-6.680%] in Phase III). The second highest average share of urban land was in the urban areas (1.196% [95%CI: 1.172-1.220%] in Phase I, 1.305% [95%CI: 1.283-1.328%] in Phase II and 1.472% [95%CI: 1.451-1.493%] in Phase III). The rural areas had the lowest average share of urban areas (0.127% [95%CI: 0.126-0.128%] in Phase I, 0.161% [95%CI: 0.160-0.162%] in Phase II and 0.205% [95%CI: 0.204-0.206%] in Phase III). The share of urban areas in rural, urban and high-density urban areas in the three phases is shown in Fig. S11. It demonstrates that there is a relationship between urban area and this classification, and also validates the rationality of the classification dataset.

The text in Line 500-513 in the revision is as followed.

"We used the settlement typologies delineated and classified from European Commission[55] to analyze the changes in the suitable habitat of *An. stephensi* during urbanization. The dataset defined the spatial entities at two hierarchical levels and the 1km^2 grids are divided into three categories of the first hierarchical level: rural, urban cluster and urban center grid cell (please see supplementary methods and Table S3 for details)[56], and generated the settlement classification layers every five years since 1975[55], which the resolution is 1km. To standardize the resolution, we resampled the grids to achieve a 2.5-minute resolution by using the nearest-neighbor method. Due to the several sub-levels of three categories in the data, we further consolidated it into three major categories: rural, urban and high-density urban for analysis. Furthermore, we selected the data in 2000 for Phase I, the data in 2010 for Phase II and the data in 2020 for Phase III. The details about the classification methods and the approaches to verify the validation of this dataset are illustrated in the supplementary materials (Fig. S11). The grid-scale classification is shown in Fig. S12."

We also illustrated the details about this classification methods in the supplementary materials and the text is as follows.

"**Supplementary Method 2. The classification method of urbanization in spatial raster datasets**

The classification dataset of urbanization is the "GHS-WUP DEGURBA settlement layers (GHS_WUP_DEGURBA_GLOBE_R2025A)" from the previous study[5]. The dataset used cell clusters population size, population and built-up area to delineate settlements[6]. The dataset defined the spatial entities at two hierarchical levels and the 1km^2 grids are divided into three categories of the first hierarchical level: rural, urban cluster and urban center grid cell[6]. Specifically, an urban center is defined as a contiguous cluster of grid cells (based on 4-connectivity) where the permanent land has a population density of at least 1,500 inhabitants per km^2. This cluster should contain a total of 50,000 or more inhabitants. Its boundaries are smoothed using 3*3 conditional majority filtering, and any holes smaller than 15 km^2 within the cluster are filled. An urban cluster is formed by contiguous grid cells connected through 8-neighbor adjacency. It should have a density of at least 300 inhabitants per km2 on permanent land and contain a total population of 5,000 or more. The rural grid cells are all the other cells that do not belong to an urban cluster, most of which will have a

density below 300 inhabitants per km^2. The second hierarchical levels contain eight categories: Urban center grid cell, dense urban cluster grid cell, semi-dense urban cluster grid cell, suburban or peri-urban grid cell, rural cluster grid cell, low density grid cell, very low density rural grid cell and water grid cell. We merged the secondary categories to derive three classifications (rural, urban and high-density urban) for analysis. Table S3 shows the introduction of categories in the first and second hierarchical levels.

We also validated the settlement of the dataset. We calculated the share of urban areas across the three classifications. We found that the highest average share of urban areas was in high-density urban areas (5.188% [95%CI: 5.004%-5.373%] in Phase I, 5.832% [95%CI: 5.657-6.007%] in Phase III and 6.512% [95%CI: 6.345-6.680%] in Phase III). The second highest average share of urban land was in the urban areas (1.196% [95%CI: 1.172-1.220%] in Phase I, 1.305% [95%CI: 1.283-1.328%] in Phase II and 1.472% [95%CI: 1.451-1.493%] in Phase III). The rural areas had the lowest average share of urban areas (0.127% [95%CI: 0.126-0.128%] in Phase I, 0.161% [95%CI: 0.160-0.162%] in Phase II and 0.205% [95%CI: 0.204-0.206%] in Phase III). The share of urban areas in rural, urban and high-density urban regions in the three phases is shown in Fig. S11. It demonstrates that there is a relationship between urban area and this classification, and also validates the rationality of the classification dataset."

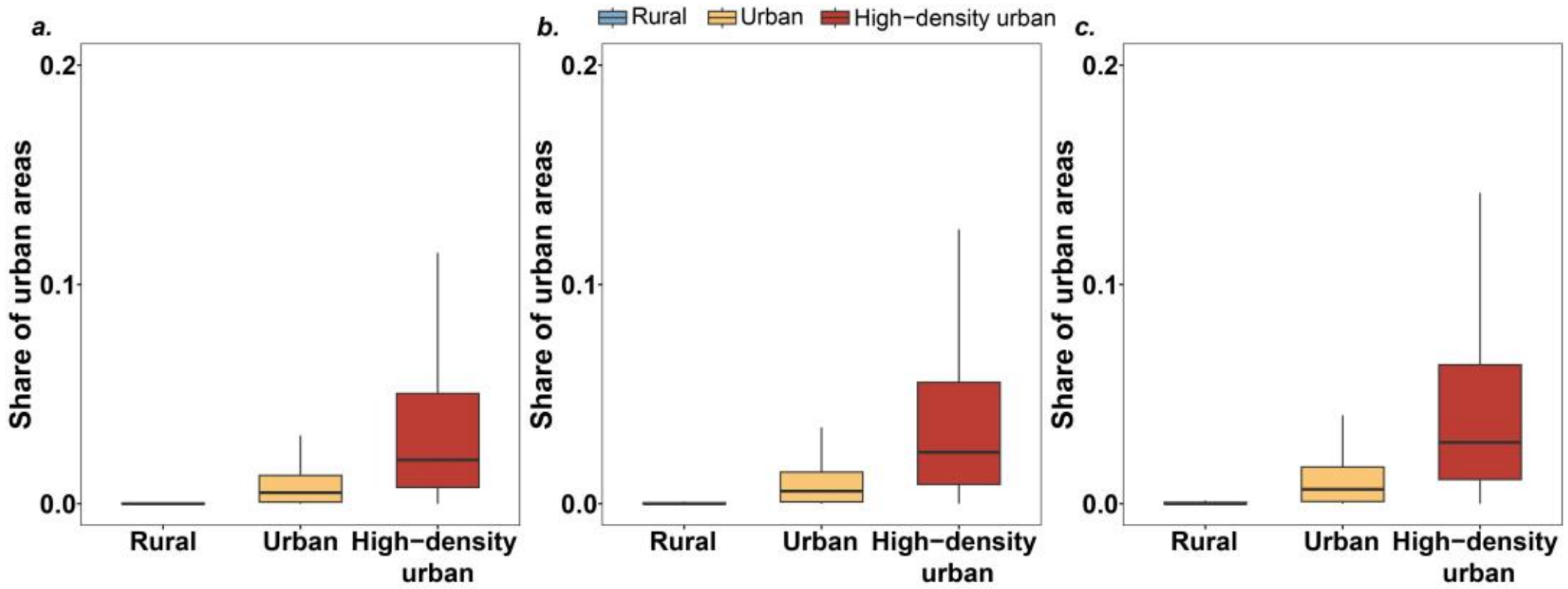

Supplementary Fig. S11. The share of urban areas in rural, urban and high-density urban in the three phases. The classification was obtained from the classification dataset [1]. a Phase I. b Phase II. c Phase III.

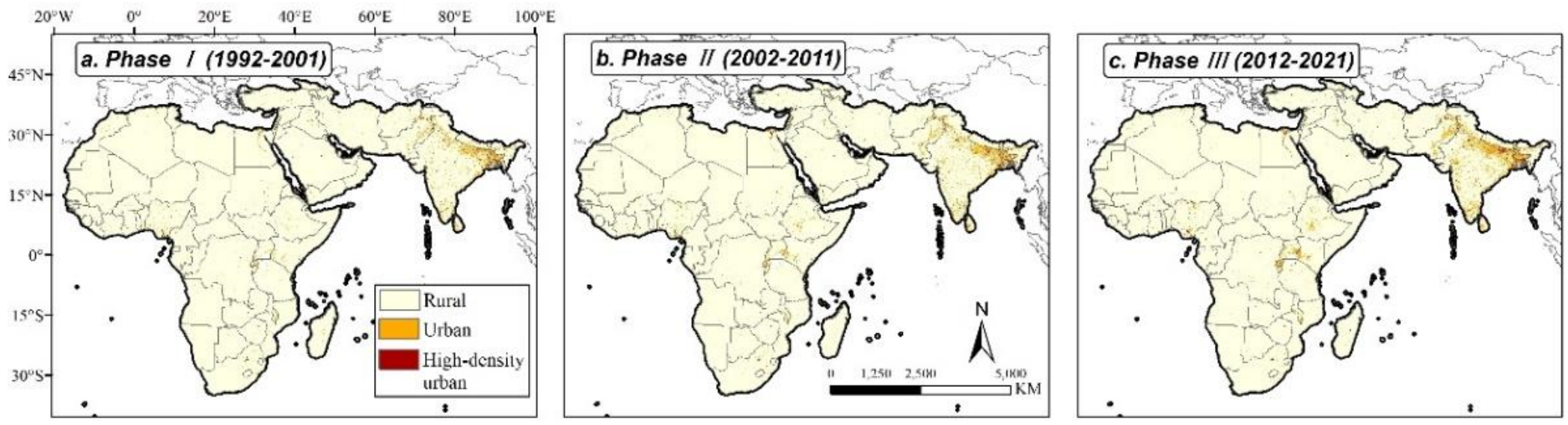

Supplementary Fig. S12. The spatial distribution of rural, urban and high-density urban in the three phases. The details about classification method are stated in 'Method' Part in the manuscript[1]**.** a Phase I. b Phase II. c Phase III.

Supplementary Table S3. The classification of rural, urban and high-density urban. The dataset is obtained from the previous study[6].

Class in original data	Sub-level class in original data (Code)	Definition	Class in the study
Urban center	Urban center grid cell (30)	An urban center consists of contiguous grid cells (4-connectivity cluster) with a density of at least 1,500 inhabitants per km^2 of permanent land, and has at least 50,000 inhabitants in the cluster with smoothed boundaries (3-by-3 conditional majority filtering12) and <15 km^2 holes filled.	High-density urban
Urban cluster	Dense urban cluster grid cell (23)	A Dense Urban Cluster consists of contiguous cells (4-connectivity cluster) with a density of at least 1,500 in habitants per km2 of permanent land and has at least 5,000 and less than 50,000 in habitants in the cluster.	Urban
	Semi-dense urban cluster grid cell (22)	A semi-dense urban cluster consists of contiguous (using four-point contiguity) grid cells within a urban cluster (level 1) at least 2 km away13 from a dense urban cluster or an urban center with a density of at least 900 inhabitants per km2 and a collective population of at least 2,500 people	
	Suburban or peri-urban grid cell (21)	All the other cells that belong to an Urban Cluster but are not part of an Urban Centre, Dense Urban Cluster or a Semi-dense Urban Cluster	
Rural	Rural cluster grid cell	A Rural Cluster consists of	Rural

	(13)	contiguous cells (8-connectivity cluster) with a density of at least 300 inhabit ants per km^2 of permanent land and has at least 500 and less than 5,000 inhabitants in the cluster.	
	Low density rural grid cell (12)	Rural grid cells with a density of at least 50 inhabitants per km2 of permanent land and are not part of a Rural Cluster.	
	Very low density rural grid cell (11)	Cells with a density of less than 50 inhabitants per km2 of permanent land.	
Water grid cells	Water grid cell (10)	All the cells with more than 0.5 share covered by permanent surface water that are not populated nor built.	Other

【Question 8】

Section "Booster regression trees": Here, the authors should describe the multicollinearity analysis performed before running the BRT model.

【Response 8】

We have revised this section in the new manuscript, and added the details about multicollinearity analysis performed in Line 520-528. The text in revision is as followed.

"Before constructing the BRT model, we first examined multicollinearity among the 25 variables in 10 datasets. Initially, we assumed the data followed a normal distribution. The Shapiro-Wilk normality test revealed significance levels (p value) consistently below 0.001, indicating rejection of the null hypothesis. We then used Q-Q plots and found that the points were not uniformly distributed on either side of the reference line, further confirming the non-normal distribution (Fig. S13). Therefore, Spearman's correlation was applied to assess multicollinearity among variables and the results are presented in Fig. S1. Variables with correlation coefficients exceeding 0.75 were excluded, ultimately containing 15 variables into the BRT model."

Finally, thanks again for reviewing our paper and for providing your useful suggestions.

The detailed comments in the attached file provided by Reviewer #1

We sincerely appreciate the reviewer's thoughtful evaluation and insightful feedback, which helped us identify inaccurate descriptions and unclear statements in the manuscript. We have carefully addressed all points requiring clarification and provided more detailed explanations of the key approaches. Due to the limited space in the response letter, we have not included the figures and tables mentioned above in this section. Thank you again for your time and expertise.

【Question 1 - Commented a1】

Taxonomic convention is that the species starts with a lower case letter (the genus starts with a capital letter).

【Response 2】

Thank you for your reminder. As suggested by the reviewer, we have corrected the "*Anopheles Stephensi*" or "*An. Stephensi*" into "*Anopheles stephensi*" or "*An. stephensi*" in the revision.

【Question 2 - Commented a2】

Line 15 "from rural to urban": There has always been some urban risk - a few species are found in urban and peri urban areas, and urban agriculture can also provide productive larval sites, so this is not quite as clear cut - but risk remains predominantly rural - so it is not shifting - perhaps reword to 'increasing risk in urban areas.'

【Response 2】

We agree with the reviewer's points that malaria risks remain predominantly rural. We thus revised this sentence, and the text in Line 16-17 is as followed.

"The invasion of *Anopheles stephensi* (*An. stephensi*) into Africa is increasing risks in urban areas, posing challenges to global malaria elimination."

【Question 3 - Commented a3】

Line 32 "ongoing climate change and trade globalization": We don't know what has caused the movement, but it is likely not to be climate change, but (as the paper points out) land use change. *Anopheles stephensi* buffers itself from extremes in temperature through its behaviour - so all the locations it is now being found in Africa have always been suitable - it just never got there before.

【Response 3】

We agree that the previous studies did not know what has caused the movement of *An. stephensi*, and the sentence "ongoing climate changing and trade globalization" in the original manuscript was misleading. Therefore, we have revised the sentence in Line 34-35 and the text in the revision is as followed.

"However, *An. stephensi* was invaded into Africa since 2012[2], and founded in Dijibouti (2012), Ethiopia (2016), the Republic of the Sudan (2019) and Ghana (2023)[3,4]."

【Question 4 - Commented a4】

Line 34 "spread": Again - as we don't know for sure how it arrived, we can't say 'spread' especially from Djibouti to Sri Lanka! I would suggest rewording to 'and in subsequent years found in more locations' (we also don't know how long it has been in any of these locations - it may simply be that people are now looking for it - it could have easily been sitting unnoticed in many of these locations for a while - African vector surveillance is not optimized for *An. stephensi*.

【Response 4】

We fully agree that it is unknown whether *An. stephensi* was spread from Djibouti to Sri Lanka. We are sorry for the deviation in writing and caused the reviewer's misunderstand on the meaning. In the original manuscript, we intended to express that *An. stephensi* was discovered in these locations after 2012, and did not suggest that *An. stephensi* was transmitted from Djibouti to other countries. We apologize for the erroneous use of the word "spread" and sincerely thank the reviewer for pointing out this issue. We have revised the sentence in the revision and the text in Line 34-35 is as followed.

"However, *An. stephensi* was invaded into Africa since 2012[2], and founded in Dijibouti (2012), Ethiopia (2016), the Republic of the Sudan (2019) and Ghana (2023)[3,4]."

【Question 5 - Commented a5】

Line 35-37 "were mainly caused by *An. stephensi* invasion": Again, although this is very likely- not yet 'proven' so perhaps 'have been strongly associated with the discovery of *An. stephensi* in these countries'

【Response 5】

We greatly appreciate the reviewer's comments. We also agree that the original sentence was not accurate. Therefore, we have revised this sentence and the text in

Line 35-37 in the revision is as followed (red bold font).

"**Malaria outbreaks were strongly associated with the discovery of *An. stephensi* in Ethiopia and Djibouti**[5], which has created new challenges for global malaria elimination[6]."

【Question 6 - Commented a6】

Line 36-37: "which slowed down the global process to eliminate malaria and consequently hedging the achievements of malaria elimination": *An. stephensi* invasion is not a global problem - it is a dominant malaria vector across Asia. It has not really slowed down malaria elimination - it has added another problem to the suite of problems.

【Response 6】

We agree that the invasion of *An. stephensi* is the dominant problem, rather than a global problem, and it has added another problem to the suite of problems. Therefore, we have revised this sentence and the text in Line 35-37 in the revision is as followed (red bold font).

"Malaria outbreaks were strongly associated with the discovery of *An. stephensi* in Ethiopia and Djibouti[5], **which has created new challenges for global malaria elimination**[6]**.**"

【Question 7 - Commented a7】

Line 37-38 "Therefore, it is necessary to analyze the main factors": I would suggest that the need to understand larval ecology of *An. stephensi* in Africa is more to understand if there are differences between African and Asian behaviours.

【Response 7】

We agree that it is necessary to understand larval ecology of *An. stephensi* in Africa if there are differences between African and Asian behaviors. Therefore, we added it into the 'Introduction' part and the text in Line 50-54 is as follows.

"For example, the Iranian study indicated that the monthly density of adult and larvae *An. stephensi* was significantly correlated with precipitation, maximum and average temperature[14]. Moreover, *An. stephensi* can tolerate extreme hot and dry conditions, enabling them to adapt to the African envirnoment[15]."

【Question 8 - Commented a8】

Line 49-50 "Evidence is accumulating that the breeding environment contributes to

the suitability of *An. Stephensi*": This is not new information and is true for all mosquitoes - suggest rewording to highlight the focus of this study and the novel element of what you are trying to find out.

【Response 8】

We appreciate this valuable comment by the reviewer. We have revised this paragraph. The overall logic of this paragraph is as follows: we first state that analyzing the habitat characteristics of *An. stephensi* is critical because vector control is one of the most important methods in malaria control. Subsequently, previous studies have focused on meteorological factors, such as temperature and precipitation. Meanwhile, some studies have predicted climate-driven habitat suitability of *An. stephensi*. However, given *An. stephensi* adaptability to urban environments, we propose incorporating urbanization indicators when predicting habitat suitability of *An. stephensi*. This addresses a gap in current research and represents one of the innovations of this study. Furthermore, the study quantitatively reveled the direct and indirect pathways through which urbanization affects *An. stephensi* habitat suitability – that is, the underlying mechanisms. Understanding these pathways is vital for developing effective countermeasures against *An. stephensi* within urbanization. The revised text in Line 49-64 is as followed.

"Environmental conditions (i.e. temperature and precipitation) were associated with the growth and breeding process of *An. stephensi*[10-13]. For example, the Iranian study indicated that the monthly density of adult and larvae *An. stephensi* was significantly correlated with precipitation, maximum and average temperature[14]. Moreover, *An. stephensi* can tolerate extreme hot and dry conditions, enabling them to adapt to the African envirnoment[15]. In addition, the studies focused on the climate-driven potential distribution of *An. stephensi*[16-18], suggesting that the current distribution of *An. stephensi* habitat suitability would be changed in the future, with previously unsuitable regions becoming suitable[17,19]. However, the larvae of *An. stephensi* was found in artificial environments, such as barrels, household water storage containers and cement/metal/plastic tanks[20,21], which may increase the malaria risk in African cities[22]. Therefore, predicting habitat suitability of *An. stephensi* should contain urbanization factors. In addition, although previous studies have indicated that *An. stephensi* can adapt to urban environment, the mechanisms by which urbanization factors affect its larvae sites remain unclear, which is critical for designing surveillance strategies and implementing targeted mosquito control measures in cities, especially in Africa."

【Question 9 - Commented a9】

Line 50 "Most": Not good to say 'Many' but only reference 2 - I would also suggest that pretty much every species on earth has optimal temperature and environmental conditions.

【Response 9】

We acknowledge the suggestion by the reviewer. We have revised this part and illustrated the specific climatic factors affecting *An. stephensi* and demonstrates their ability to adapt to hot and dry environment in Africa. The text in Line 49-54 in the revision is as followed.

"Environmental conditions (i.e. temperature and precipitation) were associated with the growth and breeding process of *An. stephensi*[10-13]. For example, the Iranian study indicated that the monthly density of adult and larvae *An. stephensi* was significantly correlated with precipitation, maximum and average temperature[14]. Moreover, *An. stephensi* can tolerate extreme hot and dry conditions, enabling them to adapt to the African envirnoment[15]."

【Question 10 – Commented a10】

Line 51-52 "For instance, the monthly densities of *An. Stephensi* adults and larvae were associated with precipitation, maximum and mean temperature": This is not providing any information, not many species wouldn't show a relationship to their own habitat niche-I feel like the point you are trying to make is not coming across clearly here -suggest rewording.

【Response 10】

Thanks for the reviewer's suggestion. Here we aim to highlight that some mathematic studies focused more on the relationship between climatic factors and *An. stephensi*, and to identify the climatic factors associated with *An. stephensi*. We also agree with the reviewer's point that this sentence is unclear and does not convey useful information. We thus revised this sentence, illustrates the details about the impact of climatic factors on *An. stephensi*, and demonstrates the specific characteristics of *An. stephensi* in Africa. The text in Line 50-54 in the revision.

"For example, the Iranian study indicated that the monthly density of adult and larvae *An. stephensi* was significantly correlated with precipitation, maximum and average temperature[14]. Moreover, *An. stephensi* can tolerate extreme hot and dry conditions, enabling them to adapt to the African envirnoment[15]."

【Question 11 – Commented a11】

Line 55-56 "It suggested that the area of *An. Stephensi* habitat suitability would increase in the future under climate change": In what way -need more specific details here? Many species impacted by climate change don't increase their range, but might shift it (some areas become suitable others are no longer suitable - this is current thinking for many malaria vectors). However, species that are closely associated/adapted to living within human altered environments have a greater chance

of increasing their range - but this is not necessarily through climate change.

【Response 11】

We agree that the distribution of some species was changed (some areas become suitable others are no longer suitable) due to climate change. Meanwhile, we also agree that species that closely adapted to living within human altered environments have a greater chance of increasing their range – but this is not necessarily through climate change, which is the aim of the study to quantitatively analyze how these human-made environments - urbanization affect the habitat suitability of *An. stephensi*. In other words, we suggest that previous studies of the *An. stephensi* habitat suitability only included climatic factors are insufficiently accurate, which ignored the impact of artificial conditions on *An. stephensi* habitat suitability. We have revised this sentence and the text (red bold font) in Line 54-61 is as followed.

"**In addition, the studies focused on the climate-driven potential distribution of *An. stephensi*[16-18], suggesting that the current distribution of *An. stephensi* habitat suitability would be changed in the future, with previously unsuitable regions becoming suitable[17,19].** However, the larvae of *An. stephensi* was found in artificial environments, such as barrels, household water storage containers and cement/metal/plastic tanks[20,21], which may increase the malaria risk in African cities[22]. Therefore, predicting habitat suitability of *An. stephensi* should contain urbanization factors."

【Question 12 – Commented a12】

Line 56-58 "In general, the previous study has demonstrated that *An. Stephensi* transmits malaria mainly in urban areas, rather than in rural areas": Again here -for a general comment like this -you need more evidence/references -and you need to make it clear this is for African vectors -as some forms of *An. Stephensi* transmit in rural areas outside Africa -also, how reliable is this statement if people have only been..

【Response 12】

We apologize for the general comment in the original manuscript. We also feel sorry that the 'Commented a15' has been collapsed and cannot be viewed completely due to character limitations. We have removed this sweeping generalization and added specific references related to the habitat of *An. stephensi* in the manuscript. The text in Line 49-64 is as follows.

"Environmental conditions (i.e. temperature and precipitation) were associated with the growth and breeding process of *An. stephensi*[10-13]. For example, the Iranian study indicated that the monthly density of adult and larvae *An. stephensi* was significantly correlated with precipitation, maximum and average temperature[14]. Moreover, *An. stephensi* can tolerate extreme hot and dry conditions, enabling them to adapt to the

African envirnoment[15]. In addition, the studies focused on the climate-driven potential distribution of *An. stephensi*[16-18], suggesting that the current distribution of *An. stephensi* habitat suitability would be changed in the future, with previously unsuitable regions becoming suitable[17,19]. However, the larvae of *An. stephensi* was found in artificial environments, such as barrels, household water storage containers and cement/metal/plastic tanks[20,21], which may increase the malaria risk in African cities[22]. Therefore, predicting habitat suitability of *An. stephensi* should contain urbanization factors. In addition, although previous studies have indicated that *An. stephensi* can adapt to urban environment, the mechanisms by which urbanization factors affect its larvae sites remain unclear, which is critical for designing surveillance strategies and implementing targeted mosquito control measures in cities, especially in Africa."

【Question 13 – Commented a13】

Line 58-61 "The surveillance research indicated that the larval of *An. Stephensi* was found in human-made environments, such as barrels, household water storage containers and cement/metal/plastic tanks[19], which increases the malaria risk in African cities and poses a threat to malaria elimination.": Again, needs more references[20] - also the ref here is for Yemen, not Africa -although can be used as a general indication of habits -assuming the Yemen population type is the same as is found in Africa.

【Response 13】

We have added the reference about the larval of *An. stephensi* in Africa, and the reference (Hawaria et al., 2023) was analyzed in Ethiopia. The text in Line 57-59 in the revision is as follows.

"However, the larvae of *An. stephensi* was found in artificial environments, such as barrels, household water storage containers and cement/metal/plastic tanks[20,21], which may increase the malaria risk in African cities[22]."

【Reference】

Hawaria D, Kibret S, Zhong D. First report of Anopheles stephensi from southern Ethiopia[J]. Malaria journal. 2023, 22(1): 373.

【Question 14 – Commented a14】

Line 61-64 "However, there is currently no quantitative research on the impact of urban environment on the habitat suitability of *An. Stephensi*, and the mechanisms of how urbanization affects the habitat suitability of *An. Stephensi* remain unknown.": I would suggest there is a fair bit of information on this from its established range in Asia.

【**Response 14**】

Thank the reviewer for the critical comment. Previous researches understanding stemmed from invaluable but spatially limited field surveys (Hawaria et al., 2023; Thomas et al., 2016). Our large-scale analysis overcomes this extrapolation limit. We estimated the spatial distribution of *An. stphensi* habitat suitability and identifies the urbanization accelerated the changes in the habitat suitability. This spatial dynamic, unreachable via field surveys, is critical for proactive surveillance.

【References】

Hawaria D, Kibret S, Zhong D. First report of Anopheles stephensi from southern Ethiopia[J]. Malaria journal. 2023, 22(1): 373.

Liu Q, Wang M, Du Y. Possible potential spread of Anopheles stephensi, the Asian malaria vector[J]. BMC Infectious Diseases. 2024, 24(1): 333.

Thomas S, Ravishankaran S, Justin J A. Overhead tank is the potential breeding habitat of Anopheles stephensi in an urban transmission setting of Chennai, India[J]. Malaria journal. 2016, 15(1): 274.

【**Question 15 – Commented a15**】

Line 73-76 "This study is one of the first to quantitatively determine and reveal the role and mechanisms of urbanization in the breeding of *An. Stephensi*. Ultimately, it helps policymakers to design scientific approaches to eliminate malaria in rapidly urbanizing areas, especially in Africa.": What is the hypothesis being tested? What are the data being used? What is the focus of this study that is novel? (we know this is a species that can thrive in urban areas, we know what larval sites it uses -so it needs to….

【**Response 15**】

We feel sorry that the 'Commented a15' has been collapsed and cannot be viewed completely due to character limitations. We have revised the research questions, content and innovations in the manuscript. The hypothesis is that urbanization accelerates the changes in the habitat suitability of *An. stephensi*. The data used in the study is occurrence data of *An. stephensi*, climate and land-use data. The study is one of the first to explore the compounding impacts of climate and land-use change on the distribution of infectious disease hosts. Ultimately, it helps to predict accurate potential distribution of *An. stephensi* and malaria transmission risks in the future by integrating both anthropogenic and climatic factors. These are the focus of the study that is novel. The text in Line 65-75 in the revision is as follows.

"In the study, we aim to explore and identify the underlying mechanisms by which urbanization on the habitat suitability for *An. stephensi*. We found that urbanization indicators, including share of urban land and population, were key factors affecting

the habitat suitability of *An. stephensi* based on occurrence, climate and land-use data. Further mathematical models indicated that urbanization primarily increased the habitat suitability of *An. stephensi* through construction activities. This work contributes to addressing the hypothesis of how urbanization affects the habitat suitability of *An. stephensi*, which is one of the first to explore the compounding impacts of climate and land-use change on the distribution of infectious disease vectors. Ultimately, it helps to predict accurate potential distribution of *An. stephensi* and malaria transmission risks in the future by integrating both anthropogenic and climatic factors."

【Question 16 – Commented a16】

"Results" Parts: As this journal format appears to put the results before the methods, there needs to be some peripheral information that makes these results make sense - what are these variables? What are these results referring to?

【Response 16】

We agree that it needs to be some peripheral information that makes these results make sense. However, due to the word limitations, we provided only a brief overview of the composition of each variable in this section. More detailed explanations are provided in the 'Method' section. The text in Line 78-88 in the revision is as followed.

"We selected 25 climate, land use and population variables, which might be related with suitability habitat of *An. stephensi*, and the details about the variables were stated in Table S2. We contained 14 variables into booster regression tress (BRT) model through multicollinearity analysis (Fig. S1). Ten sets of background points with the same level of bias as the occurrence points were included, and each set was randomly sampled 10 times, with 80% of the data for training and 20% for testing. The process was repeated 100 times for BRT model, and the final result was the average of 100 outcomes. The models with 100 iterations performed accurately with a mean area-under-the-curve (AUC) of 0.900 (95%CI: 0.896-0.903), a mean sensitivity of 0.822 (95%CI: 0.809-0.835) and a mean specificity of 0.842 (95%CI: 0.830-0.854), showing that the model simulated accurately the habitat suitability for *An. stephensi*."

【Question 17 – Commented a17】

Line 81 "25 variables": Spatial variables? How were these 25 selected - are they relevant to the biology of the mosquito?

【Response 17】

Yes, the 25 variables are spatial variables. We identified variables associated with the biological characteristics of *An. stephensi*. Specifically, for climatic factors, we reviewed the literatures and selected 19 bioclimatic variables, which commonly used

in niche modeling (Liu et al., 2024; Isamail et al., 2025). Population, as one of the key indicators of urbanization, is also a crucial aim of our analysis in the study. For land use, we focused on analyzing the relationship between urban land and the habitat suitability of *An. stephensi*. To better construct BRT model, we have also included other land use types (barren, cropland and grassland) since it can eliminate interference and define the baseline ecological niche. For example, barren areas (such as bare rock, desert, and saline-alkali land) lack standing water bodies – an absolute prerequisite for mosquito larvae – and are clearly unsuitable areas. Including them in the model helps the algorithm more clearly learn "what constitutes absolute unsuitability," thereby more precisely defining the species' potential distribution boundaries. In addition, it can mitigate sampling bias. The occurrence data likely completely avoids some land use types. If not introduced, the model will mistakenly assume "all sampled environments are potentially suitable," failing to distinguish between "unsampled" and "unsuitable." We incorporated elevation into the model. However, due to its correlation with bioclimatic variables (such as temperature), it was ultimately excluded from the final model. We have also revised the manuscript and added the details about selecting 25 variables in the revision. The text in Line 461-466 in the revision is as followed.

"The environmental factors associated with the breeding of *An. stephensi* were found from the literatures, for example, the monthly densities of *An. stephensi* adults and larvae were associated with precipitation, maximum and mean temperature[14]. We then summarized the elements affecting the four stages of *An. stephensi* breeding (Fig. 6), from which we obtained the environmental and human activities factors that may affect the breeding of *An. stephensi*."

【References】

*Liu, Q. et al. Possible potential spread of Anopheles stephensi, the Asian malaria vector. BMC Infect. Dis. **24**, 333 (2024).*

*Ismail, R. B. Y. et al. Predicting the environmental suitability for Anopheles stephensi under the current conditions in Ghana. Sci. Rep. **14**, 1116 (2024)*

【Question 18 – Commented a18】

Line 83-84 "Ten sets of background points": Independent sets? How were the background points generated -do they include the same level of bias as may be found in any none background points?

【Response 18】

We generated background points by using an environmental background based technique (Chambault et al., 2021). The method assumed that background points were distributed in areas where the environment was different from that of the occurrence points (Chambault et al., 2021). A principal component analysis (PCA) was used to obtain a two-dimensional environmental background, and we conducted a restricted

convex hull, which the environment outside the restricted convex hull was considered to the unfavorable environment for *An. stephensi*. The environmental values outside this convex hull are considered unsuitable for *An. stephensi* and we randomly generated background points outside this convex hull. Furthermore, we agree with your suggestion that sampling may be more inclined toward areas accessible to humans. Therefore, to address this sampling bias, we assigned probability values to each grid cells using a kernel density estimation (KDE) to construct the sampling approaches similar with the occurrence points. The probability value assigned to each grid according to occurrence points are shown in Fig. S10. Combined with the environmental background based technique, we generated separately points equivalent to the number of occurrence points in three phases by combining convex hulls with probability values from KDE. To improve the robustness of the results, we generated 10 sets of background points. We have also illustrated the details about the method and process in Line 444-459 in the revision, and the text is as follows.

"Due to the lack of true species absence data, most studies conducted pseudo-absence algorithms to generate background points[46,47]. We generated background points by using an environmental background based technique[47], which assumed that absent points were distributed in areas where the environment was different from that of the occurrence points. A principal component analysis (PCA) was used to obtain a two-dimensional environmental background, and we conducted a restricted convex hull, which the environment outside the restricted convex hull was considered to the unfavorable environment for *An. stephensi*. To account for sampling bias, for example sampling may be tend to target densely populated areas, we assigned probability weights to each grid cell using KDE to establish a sampling similar with the occurrence points. The results of KDE are shown in Fig. S10. Combined with the probability on the grid cells, pseudo-absence points were randomly generated outside this convex hull with the same number of points as the occurrence points in three phases. These pseudo-absence points were reprojected back into the geographic space unfavorable to the emergence of *An. stephensi*. To improve the robustness of the results, we simulated 10 different sets of background points."

【Reference】

Chambault, P. et al. A methodological framework to predict the individual and population - level distributions from tracking data. Ecography (Copenhagen). 44, 766-777 (2021).

【Question 19 – Commented a19】

Line 86-87 "assessed the accuracy of the model using AUC, sensitivity and specificity.": This does not tell you much except that the model fits the data give -this does not mean it is accurately reflecting reality -especially where the data are poor, biased, or not suitable for the purpose.

【Response 19】

We agree that the AUC, sensitivity and specificity of the model cannot tell us much if the data are poor biased, or not suitable for the purpose. Therefore, we have used various approaches to simulate the habitat suitability of *An. stephensi* and obtain accurate results. First, we performed quality control on the occurrence data. The occurrence data was collected by GBIF, WHO and literatures. For data from GBIF and WHO, we only retain occurrence points with complete records containing date, latitude and longitude information. We examined these coordinates and excluded points in obviously incorrect locations, such as locating in the ocean or where the latitude and longitude coordinates did not align with national administrative regions. The amount of data from GBIF and WHO is 670 and 844, respectively. We also searched for literatures on *An. stephensi* using the keyword "*Anopheles stephensi*", "*An. stephensi*", "*An. stephensi* suitability habitat", "*An. stephensi* habitat", "*Anopheles stephensi* suitability habitat" and "*Anopheles stephensi* habitat" in Web of Science, PubMed and Google Scholar, and six relevant literatures (Faulde et al., 2014; Abubakr et al., 2022; Hemming-Schroeder and Ahmed, 2023; Teka et al., 2023; Meshesha Balkew, 2021; Al-Eryani et al., 2023) were founded, which recorded the complete locations and dates of *An. stephensi*. We then collecting a total of 202 records from the literatures. Finally, we merged the data from these three sources, removed duplicate, and obtained a total of 632 occurrence points in the study region (54 in Phase I, 132 in Phase II and 446 in Phase III). The text in Line 429-443 in the revision is as follows.

"We obtained occurrence global records of *An. stephensi* from 1992 to 2021 through GBIF[39], WHO[40] and literatures[20,41-45]. For data from GBIF and WHO, we only retain occurrence points with complete records containing date, latitude and longitude information. We examined these coordinates and excluded points in obviously incorrect locations, such as locating in the ocean or where the latitude and longitude coordinates did not align with national administrative regions. The amount of data from GBIF and WHO is 670 and 844, respectively. We also searched for literatures on *An. stephensi* using the keyword "*Anopheles stephensi*", "*An. stephensi*", "*An. stephensi suitability habitat*", "*An. stephensi habitat*", "*Anopheles stephensi suitability habitat*" and "*Anopheles stephensi habitat*" in Web of Science, PubMed and Google Scholar, and six relevant literatures[20,41-45] were found, which recorded the complete locations and dates of *An. stephensi*. We then collecting a total of 202 records from the literatures. Finally, we merged the data from these three sources, removed duplicate, and obtained a total of 632 occurrence points in the study region (54 in Phase I, 132 in Phase II and 446 in Phase III). The distribution of occurrence points is shown in Fig. S9."

Second, we used kernel density estimation (KDE) to address sampling bias in occurrence points during generating background points. We assigned probability weights to each grid cell using the kernel density estimation (KDE) to establish a sampling similar with the occurrence points. The results of KDE are shown in Figure

S10. Furthermore, we used the environmental background based technique from the literature (Chambault et al., 2021) to generate background points based on KDE. The technique assumed that absent points were distributed in areas where the environment was different from that of the occurrence points. A principal component analysis (PCA) was used to obtain a two-dimensional environmental background, and we conducted a restricted convex hull, which the environment outside the restricted convex hull was considered to the unfavorable environment for *An. stephensi*. Combined with the probability on the grid cells, pseudo-absence points were randomly generated outside this convex hull with the same number of points as the occurrence points in three phases. These pseudo-absence points were reprojected back into the geographic space unfavorable to the emergence of *An. stephensi*. To improve the robustness of the results, we simulated 10 different sets of background points. The results in the revision were the average outputs of the 10 sets of background points. We also elaborated on this method in detail in Line 444-459 and the text in the revision is as follows.

"Due to the lack of true species absence data, most studies conducted pseudo-absence algorithms to generate background points[46,47]. We generated background points by using an environmental background based technique[47], which assumed that absent points were distributed in areas where the environment was different from that of the occurrence points. A principal component analysis (PCA) was used to obtain a two-dimensional environmental background, and we conducted a restricted convex hull, which the environment outside the restricted convex hull was considered to the unfavorable environment for *An. stephensi*. To account for sampling bias, for example sampling may be tend to target densely populated areas, we assigned probability weights to each grid cell using KDE to establish a sampling similar with the occurrence points. The results of KDE are shown in Fig. S10. Combined with the probability on the grid cells, pseudo-absence points were randomly generated outside this convex hull with the same number of points as the occurrence points in three phases. These pseudo-absence points were reprojected back into the geographic space unfavorable to the emergence of *An. stephensi*. To improve the robustness of the results, we simulated 10 different sets of background points."

However, even if we adopted the method to correct sampling bias, it would be difficult to obtain unbiased data. Therefore, we have expanded the discussion of limitations to include an explanation of data bias in the manuscript. The text in Line 397-401 in the revision is as followed.

"First, it is recognized that a perfect and unbiased sampling regime is challenging. While our methodological approach aims to mitigate this known data bias, some inherent biases associated with the original occurrence data may persist. Future study incorporating systematic field surveillance across diverse environments would be critical."

【Reference】

Abubakr M, Sami H, Mahdi I. The Phylodynamic and Spread of the Invasive Asian Malaria

Vectors, Anopheles stephensi, in Sudan[J]. Biology. 2022, 11(3): 409.
Al-Eryani S M, Irish S R, Carter T E. Public health impact of the spread of Anopheles stephensi in the WHO Eastern Mediterranean Region countries in Horn of Africa and Yemen: need for integrated vector surveillance and control[J]. Malaria Journal. 2023, 22(1): 187.
Chambault, P. et al. A methodological framework to predict the individual and population‐level distributions from tracking data. Ecography (Copenhagen). 44, 766-777 (2021).
Faulde M K, Rueda L M, Khaireh B A. First record of the Asian malaria vector Anopheles stephensi and its possible role in the resurgence of malaria in Djibouti, Horn of Africa[J]. Acta tropica. 2014, 139: 39-43.
Hemming-Schroeder E, Ahmed A. Anopheles stephensi in Africa: vector control opportunities for cobreeding An. stephensi and Aedes arbovirus vectors[J]. Trends in parasitology. 2023, 39(2): 86-90.
Meshesha Balkew P M G Y. An update on the distribution, bionomics, and insecticide susceptibility of Anopheles stephensi in Ethiopia, 2018-2020[J]. Malaria Journal. 2021(20): 263.
Teka H, Golassa L, Medhin G. Trend analysis of malaria in urban settings in Ethiopia from 2014 to 2019[J]. Malaria Journal. 2023, 22(1): 235.

【Question 20 – Commented a20】

Line 136-137 “habitat suitability of *An. Stephensi* gradually shifted toward Africa”: I’m not convinced -it shifted -it may have grown everywhere -assuming your covariates are all that limit the distribution of this species.

【Response 20】

We apologize for our misstatement. What we intended to state is that the habitat suitability of *An. stephensi* in northern and central Africa is gradually increasing, especially in Phase III. Therefore, we revised this sentence and the text in Line 150-151 in the revision is as follows.

“Our reconstruction showed that the habitat suitability of *An. stephensi* was gradually increased in northern and central Africa, particularly during Phase III (Fig. 3a and 3b).”

【Question 21 – Commented a21】

Line 141-142 “the number of changed grids that rural areas were replaced by urban areas was the largest”: So these maps seem to me to be plotting urbanisation -and then the assumption is that this is a direct link to *An. stephensi* - however there is more fine detail in the relationship. There also needs to be the presence of suitable larval sites - and there is also good evidence that despite a preference for urban areas (perhaps due to lack of competition) *An. stephensi* has also been reported from peri-urban and rural areas in natural larval sites in Ethiopia.

【Response 21】

We thank the reviewer for this helpful suggestion. These maps (Fig. 3) show the changes in the habitat suitability of *An. stephensi* during the urbanization process. The results indicate that urbanization is associated with *An. stephensi*, and we also agree with the reviewer that the relationship between urbanization and *An. Stephensi* is in fact more complex. Therefore, we further analyzed the mechanisms by which urbanization affected the habitat suitability of *An. stephensi* through PLS-PM model. We found that urbanization mainly affected the habitat suitability through construction activities. Construction booms appear to create more breeding sites for *An. stephensi* (Rani et al., 2024). The expansion of urban areas and the growth in population resulted in the need for more buildings, and it then increased the habitat suitability of *An. stephensi*. Artificial water containers prevalent in construction sites, including concrete water tanks and car washing facilities, have become breeding habitats for *An. stephensi* (Hawaria et al., 2023). The study in Ethiopia also suggested that mass urban migration and unprecedented construction booms across African cities were likely facilitating the expansion of *An. stephensi*, creating new larval habitats (Yared et al., 2023). There findings are consistent with the results of our study.

However, the study did not suggest that *An. stephensi* larval sites only existed in urban areas. Our findings only indicated that urbanization might create new larval sites for *An. stephensi*, not that *An. stephensi* larval sites were only in urban areas. We agree with the reviewer that the nature larval sites of *An. stephensi* could be found in peri-urban and rural areas. In fact, *An. stephensi* is also adaptive to the rural environment, such as freshwater ponds, stream beds, wells and seepage canals (Thomas et al.，2016). Therefore, we revised it in the “Discussion” section and the text in Line 307-329 is as follows.

“Our model suggested that urbanization accelerated the changes in the habitat suitability of *An. stephensi*. The habitat suitability of *An. stephensi* increased in Africa during Phase III (Fig. 3), and its invasion into Africa since 2012[2] also illustrated it. Urbanization progressed rapidly in Phase III, and the number of becoming urban or high-density urban grids reached 2.464 times that of Phase II (Fig. S3), and the urban-to-high-density-urban regions had the largest increase in the habitat suitability (Fig. 3). Despite the reliability of the urban classification, the delineation in the boundaries between urban and rural areas is hard. Because the population in these areas may be close to the urban threshold, and some environments may be quite similar to the habitats of *An. stephensi* in the urban areas. The field researches in the future are required to conduct in-depth analysis of *An. stephensi* larval condition in these areas. **However, the results did not indicate that *An. stephensi* larval sites was only found in urban areas. In fact, *An. stephensi* is also adaptive to the rural environment, such as freshwater ponds, stream beds, wells and seepage canals[25].** On the other hand, it is certain that multiple factors, including climate, urban land and population, have influenced the distribution of *An. stephensi*. For example, the habitat suitability in southern Ethiopia has increased, while there was a little urban area. This

might be the result of climate effect, as the habitat suitability increased in this region under climate effects alone (Fig. S5). Our study suggested that urbanization and climate change in the future might have great impact on the distribution of *An. stephesni*, potentially enabling its invasion into other continent or countries where climatic conditions were suitable and rural and urban breeding habitat existed. Therefore, the adaptation to both urban and rural environments of *An. stephensi* needs to implement updated mosquito control methods to eliminate it.”

【References】

Hawaria D, Kibret S, Zhong D. First report of Anopheles stephensi from southern Ethiopia[J]. Malaria journal. 2023, 22(1): 373.

Thomas S, Ravishankaran S, Justin J A. Overhead tank is the potential breeding habitat of Anopheles stephensi in an urban transmission setting of Chennai, India[J]. Malaria journal. 2016, 15(1): 274.

Rani A, Gupta A, Nagpal B N. Impact of urbanization on the abundance and distribution of Anophelines population in Ghaziabad district, Uttar Pradesh, India[J]. J Vector Borne Dis. 2024, 61(1): 29-42.

Yared S, Gebresilassie A, Aklilu E. Building the vector in: construction practices and the invasion and persistence of Anopheles stephensi in Jigjiga, Ethiopia[J]. Lancet Planet Health. 2023, 7(12): e999-e1005.

【Question 22 - Commented a22】

Line 177 “influenced only by population”: I am not sure of the value of this? High density population is going to be a proxy for urban areas?

【Response 22】

We used urban area and population as indicators of urbanization to analyze the relationship between urbanization and the habitat suitability of *An. stephensi*. We believe the innovation is as follows. First, although previous investigation studies can identify the habitats of *An. stephensi*, they have been constrained by spatial range and unable to cover large-scale regions. Therefore, it is crucial to derive the large-scale distribution of *An. stephensi*’s suitable habitat using mathematical analysis based on the survey data. This approach not only identifies area at risk for *An. stephensi* but also provides a basic for subsequent large-scale malaria risk assessments. Second, the study identifies urban area and population as key factors affecting the habitat suitability of *An. stephensi*. Compared with previous studies only focusing on climatic drivers (Acosta et al., 2025; Liu et al., 2024), the study uncovered the role of human activity factors. Therefore, it is necessary to contain these two parameters into future studies to accurately predict the potential distribution of *An. stephensi*. We discussed it and revised the “Discussion” section. The text in Line 292-300 is as follows.

“Share of urban land and population within urbanization indicators are key factors influencing the habitat suitability of *An. stephensi* (Fig. 1). We selected two metrics to

represent urbanization based on the previous study, including share of urban land and population[23]. Furthermore, another previous study also indicated that population played an important role in the potential distribution of *An. stephensi*[24], which is similar to our results. **Meanwhile, previous studies that predicted the potential distribution of *An. stephensi* have only considered climatic factors[17,19]. The study demonstrated that urbanization indicators, including share of urban land and population, could be included in the future studies to predict the potential distribution of *An. stephensi* more accurately.**"

【References】

Acosta A L, Castro M C, Laporta G Z. Future global distribution and climatic suitability of Anopheles stephensi[J]. Sci Rep. 2025, 15(1): 22268.

Liu Q, Wang M, Du Y. Possible potential spread of Anopheles stephensi, the Asian malaria vector[J]. BMC Infectious Diseases. 2024, 24(1): 333.

【Question 23 - Commented a23】

Line 186 "the impact of urbanization on the habitat suitability of *An. Stephensi*": As the assumption throughout is that urbanized areas = *An. stephensi* suitable habitat - I am not convinced this conclusion is really validated here -there is nuance in urban areas that impact the suitability.

【Response 23】

We thank the reviewer for the comments and apologize for the erroneous statement. However, we did not equate urbanized areas with *An. stephensi* suitable habitat. The manuscript aims to demonstrate that urbanization accelerates the changes in habitat suitability of *An. stephensi* using quantitative mathematical modelling. We agree that there is nuance in urban areas that impact the suitability. Therefore, we analyzed the mechanisms by which urbanization affects the habitat suitability using PLS-PM models, and revealed that urbanization affecting habitat suitability was mainly through construction activities. To express the aims more clearly, we have revised the manuscript as follows:

After describing the relationship between urbanization and the habitat suitability of *An. stephensi*, we detailed the pathways through which urbanization affected the habitat suitability in the 'Results' section. The text in Line 236-265 is as follows.

"**Mechanisms of urbanization on habitat suitability**

Share of urban land (URB) and population (POP) indirectly affected habitat suitability mainly through construction indexes (BLD) (Fig. 5a). Specifically, URB affected the habitat suitability of *An. stephensi* mainly through construction indexes (BLD), with the path coefficient of approximately 0.404 ($p<0.05$), which was the primary pathway affecting the habitat suitability. POP also indirectly affected habitat suitability through BLD, with the path coefficient of approximately 0.185 ($p<0.05$). In addition, POP also

affect the habitat suitability through URB and the path “POP-URB-BLD-Suitability” was approximately 0.373 ($p<0.05$). Furthermore, the increase in people living in slums in cities (SLM) could increase the habitat suitability. However, increasing in URB resulted in a decline in SLM, thus reducing the habitat suitability through the path “URB-SLM-Suitability”, with the path coefficient of approximately -0.039 ($p<0.05$). This path coefficient was offset by the path “URB-BLD-Suitability”, and URB could increase the habitat suitability of *An. stephensi*, with the total path coefficient of approximately 0.365 ($p<0.05$).

The mechanism that urbanization affected the habitat suitability of *An. stephensi* in Africa had some differences from that in the overall study area (Fig. 5b). The primary path that urbanization affected the habitat suitability was “URB-BLD-Suitability”, with the path coefficient of approximately 0.283 ($p<0.05$). URB could also affect people SLM and further affect the habitat suitability, which was similar with the results in the overall study area. In addition, POP indirectly affected the habitat suitability through BLD, with the path coefficient of approximately 0.059 ($p<0.05$). POP also affected URB and further had influence on the habitat suitability, with the path coefficient of approximately 0.201 ($p<0.05$).

Urbanization affected the habitat suitability in South and West Asia mainly through BLD (Fig. 5c). “URB-BLD-Suitability” was the primary path that urbanization affected the habitat suitability, with the path coefficient of 0.735 ($p<0.05$). POP also affected the habitat suitability mainly through BLD, with the path coefficient of 0.123 ($p<0.05$). However, URB could not increase SLM and further increase Suitability ($p>0.05$), which was different from the results in the overall study area and Africa.”

Then, we have discussed this mechanism in detail in the ‘Discussion’ section, and the text in Line 330-396 is as follows.

“Urbanization has created more breeding sites for *An. stephensi*. Urban environments are suitable for breeding of *An. stephensi*, which shows preferential breeding in man-made conditions, such as overhand tank[25], artificial water containers, construction pits[26], condensation from air conditioning, open oil drums and water-filled tires after a rainfall[21]. The study further quantitatively revealed the mechanisms of urbanization on the habitat suitability of *An. stephensi* (Fig. 5). We used infrastructure investment and build-up areas to represent the construction activities and found urbanization affected the habitat suitability was mainly through construction activities. Construction booms appear to create more breeding sites for *An. stephensi*[27]. The expansion of urban areas and the growth in population resulted in the need for more buildings, and it then increased the habitat suitability of *An. stephensi*. Artificial water containers prevalent in construction sites, including concrete water tanks and car washing facilities, have become breeding habitats for *An. stephensi*[21]. Although the study did not have construction sites data, infrastructure investment and build-up areas were closely associated with construction sites because all construction projects required construction sites during development. Therefore, it revealed that increased construction due to urban expansion and population growth added more acritical

water storage containers, which might create new larval sites for *An. stephensi*.

The proportion of people living in slums in cities would increase the habitat suitability of *An. stephensi*. The study also indicated that *An. stephensi* appeared in urban slums in Chennai, India[28]. However, we found that an increase in urban land would reduce the proportion of people living in slums in cities, thereby decreasing the habitat suitability through this pathway in the overall study area (Fig. 5a). From this perspective, urbanization is beneficial for reducing the habitat suitability of *An. stephensi*. Due to the relatively minor path effect being offset by the paths through construction activities, overall urbanization still increased the habitat suitability. Compared with previous investigation studies[21,25], our study incorporated quantitative analysis and revealed the mechanisms by which urbanization affected the habitat suitability on a large scale. It can provide scientific reference for better predicting the distribution of *An. stephensi* and design strategies to response to malaria transmission in the future.

Africa is one of the regions with the fastest urbanization globally[29], and is facing unprecedented population growth in the 21st century, much of which will be absorbed by urban areas, with the total urban population projected to triple from current levels[30]. Malaria in Africa has been commonly considered a rural transmission disease[31]. However, we found that urbanization increased the habitat suitability in Africa (Fig. 3d and Fig. 4i-l), which might pose a malaria risks to African cities. The previous study also indicated that the African cities face challenges to malaria control, mainly due to unplanned urbanization and transmission of *An. stephensi*[32]. In fact, there have been multiple malaria outbreaks caused by the invasion of *An. stephensi* in Africa[33,34]. For example, spatial aggregation of *Plasmodium falciparum* infection around malaria patients in Ethiopia was associated with *An. stephensi* in the vicinity of households under a prospective case-control design experiment[33]. Furthermore, urbanization also affected the habitat suitability was mainly through construction activities (Fig. 5b). The study in Ethiopia also demonstrated that mass urban migration and unprecedented construction booms across African cities were likely facilitating the expansion of *An. stephensi*, creating new larval habitats[35]. Although the differences in behaviors of *An. stephensi* between Africa, and South and West Asia are not included in the study, it is certain that *An. stephensi*, which adapt to both rural and urban environments, may add a new threat to malaria transmission in Africa. It needs to apply targeted mosquito control technologies and strengthen surveillance to address malaria transmission in Africa.

An. stephensi was the malaria vector mainly in South Asia and the Arabian Peninsula before 2011[2]. Many studies in Asia confirmed that *An. stephensi* was transmitted malaria mainly in cities[36,37], for example, the study indicated that *An. stephensi* was a major urban malaria vector in India[37]. Our study also indicated that the habitat suitability was high in urban and high-density urban areas in South and West Asia (Fig. 2). The changes in habitat suitability in South Asia and West Asia were relatively lower than that in Africa, and even decreased in some areas (Fig. 3). Single-factor analysis indicated that this decline in some areas might be climate-driven. For

instance, the reduction in the habitat suitability in southeastern India coincided with a climate-driven decrease in habitat suitability (Fig. 3 and Fig. S5). Furthermore, urbanization also primarily affected the habitat suitability through construction activities. However, urbanization could increase the proportion of people living in slums in cites, which differed from the results in the overall study area and Africa (Fig. 5c). Urbanization in Asia was progressing faster than in Africa[38], which may lead to regional development imbalances, resulting in an increase in slums. Therefore, urban planning should be prioritized during urbanization to reduce *An. stephensi* breeding sites."

【Question 24 - Commented a24】

Line 253-254 "and have changed dramatically over the past 30 years.": In what way? I would suggest they haven't change very much at all -they are still concrete or brick water tanks, artificial water containers -locations that buffer the mosquito from temperature extremes -but they may have increased due to anthropogenic land use change.

【Response 24】

We apologize for our misstatement in the original manuscript. Several researchers have identified the presence of *An. stephensi* in Africa since 2012. Therefore, we aimed to demonstrate that the spatial distribution of *An. stephensi* has undergone changes over past 30 years in the study. We agree that the breeding sites of *An. stephensi* have not changed very much at all in the past 30 years. Therefore, we corrected the expression and the text in Line 277-278 in the revision is as follows.

"As a critical vector of malaria, the spatial distribution of *An. stephensi* have been affected by human activities and changed dramatically over the past 30 years."

【Question 25 - Commented a25】

Line 255 "occurrence points": Where were the data -what is the spatial range? How evenly spread were these data -what was the sampling regime? What impact is sampling bias?

【Response 25】

The occurrence data were collected from GBIF, WHO and literatures. The details about the occurrence data and the sampling bias are as followed.

First, the data of *An. stephensi* occurrence was collected from GBIF, WHO and literatures. For data from GBIF and WHO, and we only retain occurrence points with complete records containing date, latitude and longitude information. We examined these coordinates and excluded points in obviously incorrect locations, such as

locating in the ocean or where the latitude and longitude coordinates did not align with national administrative regions. The amount of data from GBIF and WHO is 670 and 844, respectively. We also searched for literatures on *An. stephensi* using the keyword "*Anopheles stephensi*", "*An. stephensi*", "*An. stephensi suitability habitat*", "*An. stephensi habitat*", "*Anopheles stephensi suitability habitat*" and "*Anopheles stephensi habitat*" in Web of Science, PubMed and Google Scholar, and six relevant literatures were founded, which recorded the complete locations and dates of *An. stephensi*. We then collecting a total of 202 records from the literatures (Faulde et al., 2014; Abubakr et al., 2022; Hemming-Schroeder and Ahmed, 2023; Teka et al., 2023; Meshesha Balkew, 2021; Al-Eryani et al., 2023). We merged these data, removed duplicate, and obtained a total of 632 occurrence points, which has environment data, in the study region (54 in Phase I, 132 in Phase II and 446 in Phase III). The distribution of occurrence points is shown in Fig. S9. We have added the detailed illustration in Line 429-443 and the text in the revision is as follows.

"We obtained occurrence global records of *An. stephensi* from 1992 to 2021 through GBIF[39], WHO[40] and literatures[20,41-45]. For data from GBIF and WHO, we only retain occurrence points with complete records containing date, latitude and longitude information. We examined these coordinates and excluded points in obviously incorrect locations, such as locating in the ocean or where the latitude and longitude coordinates did not align with national administrative regions. The amount of data from GBIF and WHO is 670 and 844, respectively. We also searched for literatures on *An. stephensi* using the keyword "*Anopheles stephensi*", "*An. stephensi*", "*An. stephensi suitability habitat*", "*An. stephensi habitat*", "*Anopheles stephensi suitability habitat*" and "*Anopheles stephensi habitat*" in Web of Science, PubMed and Google Scholar, and six relevant literatures[20,41-45] were found, which recorded the complete locations and dates of *An. stephensi*. We then collecting a total of 202 records from the literatures. Finally, we merged the data from these three sources, removed duplicate, and obtained a total of 632 occurrence points in the study region (54 in Phase I, 132 in Phase II and 446 in Phase III). The distribution of occurrence points is shown in Fig. S9."

Second, we used kernel density estimation (KDE) to address sampling bias in occurrence points during generating background points. We assigned probability weights to each grid cell using the kernel density estimation (KDE) to establish a sampling similar with the occurrence points. The results of KDE are shown in Figure S10. Furthermore, we used the environmental background based technique from the literature (Chambault et al., 2021) to generate background points based on KDE. The technique assumed that absent points were distributed in areas where the environment was different from that of the occurrence points. A principal component analysis (PCA) was used to obtain a two-dimensional environmental background, and we conducted a restricted convex hull, which the environment outside the restricted convex hull was considered to the unfavorable environment for *An. stephensi*. Combined with the probability on the grid cells, pseudo-absence points were randomly generated outside this convex hull with the same number of points as the

occurrence points in three phases. These pseudo-absence points were reprojected back into the geographic space unfavorable to the emergence of *An. stephensi*. To improve the robustness of the results, we simulated 10 different sets of background points. The results in the revision were the average outputs of the 10 sets of background points. We also elaborated on this method in detail in Line 444-459 and the text in the revision is as follows.

"Due to the lack of true species absence data, most studies conducted pseudo-absence algorithms to generate background points[46,47]. We generated background points by using an environmental background based technique[47], which assumed that absent points were distributed in areas where the environment was different from that of the occurrence points. A principal component analysis (PCA) was used to obtain a two-dimensional environmental background, and we conducted a restricted convex hull, which the environment outside the restricted convex hull was considered to the unfavorable environment for *An. stephensi*. To account for sampling bias, for example sampling may be tend to target densely populated areas, we assigned probability weights to each grid cell using KDE to establish a sampling similar with the occurrence points. The results of KDE are shown in Fig. S10. Combined with the probability on the grid cells, pseudo-absence points were randomly generated outside this convex hull with the same number of points as the occurrence points in three phases. These pseudo-absence points were reprojected back into the geographic space unfavorable to the emergence of *An. stephensi*. To improve the robustness of the results, we simulated 10 different sets of background points."

However, as the review point out, even if we adopted the method to correct sampling bias, it would be difficult to obtain unbiased data. Therefore, we have expanded the discussion of limitations to include an explanation of data bias in the manuscript. The text in Line 397-401 in the revision is as follows.

"First, it is recognized that a perfect and unbiased sampling regime is challenging. While our methodological approach aims to mitigate this known data bias, some inherent biases associated with the original occurrence data may persist. Future study incorporating systematic field surveillance across diverse environments would be critical."

In summary, the occurrence data were obtained from GBIF, WHO and literatures, covering the study area: Africa, South Asia and West Asia. We believed that sampling bias existed, such as sampling places might be accessible to people or densely populated. Therefore, we used KDR to solve the sampling bias in occurrence data. Meanwhile, we also demonstrated the limitations in occurrence data in the 'Discussion' part.

【Reference】

Abubakr M, Sami H, Mahdi I. The Phylodynamic and Spread of the Invasive Asian Malaria Vectors, Anopheles stephensi, in Sudan[J]. Biology. 2022, 11(3): 409.

Al-Eryani S M, Irish S R, Carter T E. Public health impact of the spread of Anopheles stephensi in

the WHO Eastern Mediterranean Region countries in Horn of Africa and Yemen: need for integrated vector surveillance and control[J]. Malaria Journal. 2023, 22(1): 187.
Chambault, P. et al. A methodological framework to predict the individual and population‐level distributions from tracking data. Ecography (Copenhagen). 44, 766-777 (2021).
Faulde M K, Rueda L M, Khaireh B A. First record of the Asian malaria vector Anopheles stephensi and its possible role in the resurgence of malaria in Djibouti, Horn of Africa[J]. Acta tropica. 2014, 139: 39-43.
Hemming-Schroeder E, Ahmed A. Anopheles stephensi in Africa: vector control opportunities for cobreeding An. stephensi and Aedes arbovirus vectors[J]. Trends in parasitology. 2023, 39(2): 86-90.
Meshesha Balkew P M G Y. An update on the distribution, bionomics, and insecticide susceptibility of Anopheles stephensi in Ethiopia, 2018-2020[J]. Malaria Journal. 2021(20): 263.
Teka H, Golassa L, Medhin G. Trend analysis of malaria in urban settings in Ethiopia from 2014 to 2019[J]. Malaria Journal. 2023, 22(1): 235.

【Question 26 - Commented a26】

Line 255-256: "*An. Stephensi*, and found that urbanization indexes, including population and share of urban land": As it is likely that the data are biased to urban areas -the modelling is reflecting this -this of course may be accurate -but without addressing the data bias, it is not possible to make too many conclusions.

【Response 26】

We agree with Reviewer #1 that the data bias would lead to inaccurate results. We account for the data bias through kernel density estimation (KDE). Specifically, we assigned probability values to each grid cells using KDE to construct the sampling approaches similar with the occurrence points, and the results of KDE are shown in Fig. S10. We generated background points by using an environmental background based technique (Chambault et al., 2021). The method assumed that background points were distributed in areas where the environment was different from that of the occurrence points (Chambault et al., 2021). A principal component analysis (PCA) was used to obtain a two-dimensional environmental background, and we conducted a restricted convex hull, which the environment outside the restricted convex hull was considered to the unfavorable environment for *An. stephensi*. The environmental values outside this convex hull are considered unsuitable for *An. stephensi* and we randomly generated background points outside this convex hull. Finally, we generated separately background points equivalent to the number of occurrence points in three phases by combining convex hulls with probability weights from KDE. To improve the robustness of the results, we generated 10 sets of background points. The results in the revision were the average of the results of 10 datasets (occurrence and background points) included in the model. We have also illustrated the details about the method and process in Line 444-459 in the revision, and the text is as follows.

"Due to the lack of true species absence data, most studies conducted pseudo-absence

algorithms to generate background points[46,47]. We generated background points by using an environmental background based technique[47], which assumed that absent points were distributed in areas where the environment was different from that of the occurrence points. A principal component analysis (PCA) was used to obtain a two-dimensional environmental background, and we conducted a restricted convex hull, which the environment outside the restricted convex hull was considered to the unfavorable environment for *An. stephensi*. To account for sampling bias, for example sampling may be tend to target densely populated areas, we assigned probability weights to each grid cell using KDE to establish a sampling similar with the occurrence points. The results of KDE are shown in Fig. S10. Combined with the probability on the grid cells, pseudo-absence points were randomly generated outside this convex hull with the same number of points as the occurrence points in three phases. These pseudo-absence points were reprojected back into the geographic space unfavorable to the emergence of *An. stephensi*. To improve the robustness of the results, we simulated 10 different sets of background points."

【Reference】

Chambault, P. et al. A methodological framework to predict the individual and population‐level distributions from tracking data. Ecography (Copenhagen). 44, 766-777 (2021).

【Question 27 – Commented a27】

Line 265 "Population is the most critical factor influencing habitat suitability": Not necessarily population itself -but the consequences of larger populations -increased water storage, increased standing water during construction etc.

【Response 27】

We agree that population itself did not increase the habitat suitability of *An. stephensi*, but the consequences of larger populations, such as increasing water storage, standing water during construction, can increase the habitat suitability of *An. stephensi*. In fact, we found that population could affect the habitat suitability in BRT model. Furthermore, we analyzed the mechanisms by which population affected the habitat suitability of *An. stephensi*, and found that population growth drove increased construction demand, which was the larval environments of *An. stephensi*, thereby increasing the habitat suitability. We have revised it in the 'Discussion' part, and the details are as follows.

Firstly, we preliminarily discussed the relationship between population and the habitat suitability of *An. stephensi* based on the results of the BRT model in the second paragraph of 'Discussion' part. The text in Line 289-297 in the revision is as followed and the red font sentences are about population.

"**Share of urban land and population within urbanization indicators are key factors influencing the habitat suitability of *An. stephensi* (Fig. 1). We selected**

two metrics to represent urbanization based on the previous study, including share of urban land and population[23]. Furthermore, another previous study also indicated that population played an important role in the potential distribution of *An. stephensi*[24], which is similar to our results. Meanwhile, previous studies that predicted the potential distribution of *An. stephensi* have only considered climatic factors[17,19]. The study demonstrated that urbanization indicators, including share of urban land and population, could be included in the future studies to predict the potential distribution of *An. stephensi* more accurately."

Then, in the third paragraph, we discussed the mechanisms by which population drove the habitat suitability of *An. stephensi*. The text in Line 330-347 is as follows and the red font is about population.

"**Urbanization has created more breeding sites for *An. stephensi*. Urban environments are suitable for breeding of *An. stephensi*, which shows preferential breeding in man-made conditions, such as overhand tank[25], artificial water containers, construction pits[26], condensation from air conditioning, open oil drums and water-filled tires after a rainfall[21]. The study further quantitatively revealed the mechanisms of urbanization on the habitat suitability of *An. stephensi* (Fig. 5). We used infrastructure investment and build-up areas to represent the construction activities and found urbanization affected the habitat suitability was mainly through construction activities. Construction booms appear to create more breeding sites for *An. stephensi*[27]. The expansion of urban areas and the growth in population resulted in the need for more buildings, and it then increased the habitat suitability of *An. stephensi*. Artificial water containers prevalent in construction sites, including concrete water tanks and car washing facilities, have become breeding habitats for *An. stephensi*[21].** Although the study did not have construction sites data, infrastructure investment and build-up areas were closely associated with construction sites because all construction projects required construction sites during development. Therefore, it revealed that increased construction due to urban expansion and population growth added more acritical water storage containers, which might create new larval sites for *An. stephensi*."

【Question 28 - Commented a28】

Line 282-285 "Construction booms appear to create more breeding sites for *An. Stephensi*. We used infrastructure investment as a proxy for construction sites and found that population indirectly affected habitat suitability through infrastructure investment (Figure 5a)": This is an interesting and potentially useful additional for making estimates of urbanization and indicating the edges where land use is being significantly changed.

【Response 28】

We sincerely appreciate the reviewer's insightful feedback. We also further explored

mechanisms by which urbanization affected the habitat suitability of *An. stephensi*. Compared with the original manuscript, we added build-up areas combined with infrastructure investment to represent construction activities. *An. stephensi* is suitable for urban environments, such as overhand tank (Thomas et al., 2016), artificial water containers, construction pits (Guofa Zhou, 2024), condensation from air conditioning, open oil drums and water-filled tires after a rainfall (Hawaria et al., 2023). We also found that the expansion of urban areas and the growth in population resulted in the need for more buildings, and it then increased the habitat suitability of *An. stephensi*. Artificial water containers prevalent in construction sites, including concrete water tanks and car washing facilities, have become breeding habitats for *An. stephensi* (Hawaria et al., 2023). We also agree that if more refined architectural indicators become available, we can estimate the degree of urbanization and delineate the boundary areas where significant changes in land use occur in the future. We further optimized these sentences and the text in Line 334-342 is as follows.

"The study further quantitatively revealed the mechanisms of urbanization on the habitat suitability of *An. stephensi* (Fig. 5). We used infrastructure investment and build-up areas to represent the construction activities and found urbanization affected the habitat suitability was mainly through construction activities. Construction booms appear to create more breeding sites for *An. stephensi*[27]. The expansion of urban areas and the growth in population resulted in the need for more buildings, and it then increased the habitat suitability of *An. stephensi*. Artificial water containers prevalent in construction sites, including concrete water tanks and car washing facilities, have become breeding habitats for *An. stephensi*[21]."

【*References*】

Guofa Zhou D Z D Y. Anopheles stephensi ecology and control in Africa[J]. Trends in Parasitology. 2024, 40(2): 102-105.

Hawaria D, Kibret S, Zhong D. First report of Anopheles stephensi from southern Ethiopia[J]. Malaria journal. 2023, 22(1): 373.

Thomas S, Ravishankaran S, Justin J A. Overhead tank is the potential breeding habitat of Anopheles stephensi in an urban transmission setting of Chennai, India[J]. Malaria journal. 2016, 15(1): 274.

【Question 29 - Commented a29】

Line 296 "these prove": 'Prove' is a very string word to use here -the model is describing the data given -not proving reality.

【Response 29】

We apologize for the incorrect wording. We have revised this paragraph, and removed the word "prove". We mainly illustrated the innovations and practical significance of the study in the revision and the text in Line 355-359 is as follows.

"Compared with previous investigation studies[21,25], our study incorporated quantitative analysis and revealed the mechanisms by which urbanization affected the habitat suitability on a large scale. It can provide scientific reference for better predicting the distribution of *An. stephensi* and design strategies to response to malaria transmission in the future."

【Question 30 - Commented a30】

Line 330 "*An. Stephensi* was the main vector in South Asia and the Arabian Peninsula before 2011.": Not sure if it just how this is phrased -but *An. stephensi* one of many dominant vectors in South Asia -and arguably, *An. culicifaciesis* the 'major' vector.

【Response 30】

We apologize for the incorrect expression. *An. stephensi* was mainly located in South Asia and the Arabian Peninsula before 2011. Therefore, we revised this sentence and the text in Line 381-382 is as followed.

"*An. stephensi* was the malaria vector mainly in South Asia and the Arabian Peninsula before 2011[2]."

【Question 31 - Commented a31】

Line 342-344 "For example, a study in India showed that the insecticides have been used to eliminate *An. Stephensi*[5], which could be the reason that the habitat suitability was reduced in some areas.": This is misleading.

【Response 31】

We apologize for the misleading sentence. In the revision, we removed this sentence and revised this paragraph. The text in Line 381-396 in the revision is as followed.

"*An. stephensi* was the malaria vector mainly in South Asia and the Arabian Peninsula before 2011[2]. Many studies in Asia confirmed that *An. stephensi* was transmitted malaria mainly in cities[36,37], for example, the study indicated that *An. stephensi* was a major urban malaria vector in India[37]. Our study also indicated that the habitat suitability was high in urban and high-density urban areas in South and West Asia (Fig. 2). The changes in habitat suitability in South Asia and West Asia were relatively lower than that in Africa, and even decreased in some areas (Fig. 3). Single-factor analysis indicated that this decline in some areas might be climate-driven. For instance, the reduction in the habitat suitability in southeastern India coincided with a climate-driven decrease in habitat suitability (Fig. 3 and Fig. S5). Furthermore, urbanization also primarily affected the habitat suitability through construction activities. However, urbanization could increase the proportion of people living in slums in cites, which differed from the results in the overall study area and Africa (Fig.

5c). Urbanization in Asia was progressing faster than in Africa[38], which may lead to regional development imbalances, resulting in an increase in slums. Therefore, urban planning should be prioritized during urbanization to reduce *An. stephensi* breeding sites."

【Question 32 - Commented a32】

Line 368-369 "there may still be errors caused by human observations in occurrence points.": This needs to be addressed.

【Response 32】

Thanks for the valuable comment. We conducted quality checks on occurrence data and generated background points with the same sampling bias as the occurrence points to account for the errors caused by human observations in occurrence points.

First, we examined the data from GBIF, WHO and literature sources. For data from GBIF and WHO, we only retain occurrence points with complete records, including date, latitude and longitude. We also examined these coordinates and excluded points in obviously incorrect locations, such as locating in the ocean or where the latitude and longitude coordinates did not align with national administrative regions. Meanwhile, we also searched for literatures by using the keywords "*Anopheles stephensi*", "*An. stephensi*", "*An. stephensi suitability habitat*", "*An. stephensi habitat*", "*Anopheles stephensi suitability habita*t" and "*Anopheles stephensi habitat*" in Web of Science, PubMed and Google Scholar, and collected a total of 202 records. We merged the data from these sources, removed duplicate, and obtained a total of 632 occurrence points in the study region. The distribution of occurrence points is shown in Fig. S9. The text in Line 429-443 in the revision is as follows.

"We obtained occurrence global records of *An. stephensi* from 1992 to 2021 through GBIF[39], WHO[40] and literatures[20,41-45]. For data from GBIF and WHO, we only retain occurrence points with complete records containing date, latitude and longitude information. We examined these coordinates and excluded points in obviously incorrect locations, such as locating in the ocean or where the latitude and longitude coordinates did not align with national administrative regions. The amount of data from GBIF and WHO is 670 and 844, respectively. We also searched for literatures on *An. stephensi* using the keyword "*Anopheles stephensi*", "*An. stephensi*", "*An. stephensi suitability habitat*", "*An. stephensi habitat*", "*Anopheles stephensi suitability habitat*" and "*Anopheles stephensi habitat*" in Web of Science, PubMed and Google Scholar, and six relevant literatures[20,41-45] were found, which recorded the complete locations and dates of *An. stephensi*. We then collecting a total of 202 records from the literatures. Finally, we merged the data from these three sources, removed duplicate, and obtained a total of 632 occurrence points in the study region (54 in Phase I, 132 in Phase II and 446 in Phase III). The distribution of occurrence points is shown in Fig. S9."

Second, we used kernel density function (KDE) to account for the data bias reflected in occurrence data in generating background points. We assigned probability weights to each grid cell using the KDE to establish a sampling similar with the occurrence points, and the probability values assigned to each grid according to occurrence points are shown in Figure S10. We generated background points by using an environmental background based technique (Chambault et al., 2021). The method assumed that background points were distributed in areas where the environment was different from that of the occurrence points (Chambault et al., 2021). A principal component analysis (PCA) was used to obtain a two-dimensional environmental background, and we conducted a restricted convex hull excluding, which the environment outside the restricted convex hull was considered to the unfavorable environment for *An. stephensi*. The environmental values outside this convex hull are considered unsuitable for *An. stephensi* and we randomly generated background points outside this convex hull. Combined with the probability weights from KDE, we generated separately points equivalent to the number of occurrence points in three phases by combining convex hulls. To improve the robustness of the results, we generated 10 sets of background points. We have also illustrated the details about the method and process in Line 444-459 in the revision, and the text is as follows.

"Due to the lack of true species absence data, most studies conducted pseudo-absence algorithms to generate background points[46,47]. We generated background points by using an environmental background based technique[47], which assumed that absent points were distributed in areas where the environment was different from that of the occurrence points. A principal component analysis (PCA) was used to obtain a two-dimensional environmental background, and we conducted a restricted convex hull, which the environment outside the restricted convex hull was considered to the unfavorable environment for *An. stephensi*. To account for sampling bias, for example sampling may be tend to target densely populated areas, we assigned probability weights to each grid cell using KDE to establish a sampling similar with the occurrence points. The results of KDE are shown in Fig. S10. Combined with the probability on the grid cells, pseudo-absence points were randomly generated outside this convex hull with the same number of points as the occurrence points in three phases. These pseudo-absence points were reprojected back into the geographic space unfavorable to the emergence of *An. stephensi*. To improve the robustness of the results, we simulated 10 different sets of background points."

However, although we applied two approaches to minimize sampling bias, no completely unbiased sampling regime exists, minor deviations may still occur. Therefore, we have added this into limitations in our discussion and the text in Line 397-401 in the revision is as followed.

"First, it is recognized that a perfect and unbiased sampling regime is challenging. While our methodological approach aims to mitigate this known data bias, some inherent biases associated with the original occurrence data may persist. Future study incorporating systematic field surveillance across diverse environments would be critical."

【Reference】

Chambault, P. et al. A methodological framework to predict the individual and population - level distributions from tracking data. Ecography (Copenhagen). 44, 766-777 (2021).

【Question 33 - Commented a33】

Line 370-371 "to standardize the data to a resolution of 2.5 minutes. This could potentially lead to a loss of information in the precise data.": Also many of the covariates didn't cover the time period you were modelling.

【Response 33】

We agree that some covariates did not cover the time period you were modelling due to the data limitation. Although we strove to ensure that data remained on the same time scale as much as possible, this issue was indeed unavoidable. Therefore, we added it into the 'limitation' part in the 'Discussion' section and the text in Line 401-405 is as followed.

"Second, due to differences in data precision, we used resampling to standardize the data to a resolution of 2.5 minutes. This could potentially lead to a loss of information in the precise data. Meanwhile, due to the limited temporal updates of data, we adopted multiple approaches to unify the time scale, which may also have a slight impact on the results."

【Question 34 - Commented a34】

Line 378 "West Asia, South Asia": Isn't this already well known/studied in Asia - plenty of south Asian publications?

【Response 34】

We agree that the plenty of publications about *An. stephensi* in south Asia. However, there are three reasons why we included West Asia and South Asia into our study. First, although numerous studies on *An. stephensi* have been conducted in south Asia, and the studies found the its breeding sites in urban environments, quantitative research on analyzing the impact of urban environments on the habitat suitability of *An. stephensi* is needed since we can identify the key variables affecting *An. stephensi* in the study. Identifying key covariates is crucial for both forecasting *An. stephensi* potential distribution and predicting malaria risks related with *An. stephensi* in the future. Therefore, quantifying the relationship between urban area and population with *An. stephensi* suitable habitats is essential for future risk prediction. On the other hand, our study further analyzed the mechanisms and revealed specific pathways through which urbanization affects *An. stephensi* habitat suitability. It is critical for urban planning and targeting the elimination of *An. stephensi* breeding sites during

urbanization. Meanwhile, the studies on *An. stephensi* in Africa remains limited, comparing the results of *An. stephensi* in West and South Asia, and Africa is one of the aims in the manuscript. Although the species between Asian and African populations of *An. stephensi* may be different, this comparison can partially reveal that how different process of urbanization affects the habitat suitability of *An. stephensi* in West and South Asia and Africa.

【Question 35 - Commented a35】

Line 378-379 “We firstly generated background points using an environmental background-based technique.”: This needs far more detail!! What was done to make sure the background data reflected the bias seen in the occurrence data?

【Response 35】

We appreciate Reviewer #1’s expertise and concur that our initial approach to data analysis could be improved. In the revision, we used kernel density function (KDE) to account for the data bias of background data, which reflected in the occurrence data, and have also illustrated the details about the methods of generating background points. Specifically, we assigned probability values to each grid cells using KDE to construct the sampling approaches similar with the occurrence points. We generated background points by using an environmental background based technique (Chambault et al., 2021). The method assumed that background points were distributed in areas where the environment was different from that of the occurrence points (Chambault et al., 2021). A principal component analysis (PCA) was used to obtain a two-dimensional environmental background, and we conducted a restricted convex hull, which the environment outside the restricted convex hull was considered to the unfavorable environment for *An. stephensi*. The environmental values outside this convex hull are considered unsuitable for *An. stephensi* and we randomly generated background points outside this convex hull. Finally, we generated separately background points equivalent to the number of occurrence points in three phases by combining convex hulls with probability weights from KDE. To improve the robustness of the results, we generated 10 sets of background points. The results in the revision were the average of the results of 10 datasets (occurrence and background points) included in the model. We have also illustrated the details about the method in the revision.

Before writing the specific methods, we briefly outlined our methodology steps in the study. We have also revised it in this section. The text in Line 415-417 in the revision is as follows.

“We firstly generated background points using an environmental background-based technique and used kernel density estimation (KDE) to reflected the bias seen in the occurrence points.”

The text in Line 444-459 in ‘Occurrence and background points with bias correction’

part is as follows.

"Due to the lack of true species absence data, most studies conducted pseudo-absence algorithms to generate background points[46,47]. We generated background points by using an environmental background based technique[47], which assumed that absent points were distributed in areas where the environment was different from that of the occurrence points. A principal component analysis (PCA) was used to obtain a two-dimensional environmental background, and we conducted a restricted convex hull, which the environment outside the restricted convex hull was considered to the unfavorable environment for *An. stephensi*. To account for sampling bias, for example sampling may be tend to target densely populated areas, we assigned probability weights to each grid cell using KDE to establish a sampling similar with the occurrence points. The results of KDE are shown in Fig. S10. Combined with the probability on the grid cells, pseudo-absence points were randomly generated outside this convex hull with the same number of points as the occurrence points in three phases. These pseudo-absence points were reprojected back into the geographic space unfavorable to the emergence of *An. stephensi*. To improve the robustness of the results, we simulated 10 different sets of background points."

【Reference】

Chambault, P. et al. A methodological framework to predict the individual and population - level distributions from tracking data. Ecography (Copenhagen). 44, 766-777 (2021).

【Question 36 – Commented a36】

Line 380 "to analyze the key factors": This model won't tell you much more than you tell it if you don't put in appropriate covariates.

【Response 36】

We agree that this model would not tell us much more than we tell it if we do not put in appropriate covariates. Therefore, we reviewed a large volume of literatures on *An. stephensi* to identify factors that may affect its breeding. We have included four factors: meteorological factors, population, land use and elevation. For meteorological factors, as we illustrated in the 'Introduction' part, it may affect the breeding of *An. stephensi*, a point that is also included in most literatures. In the manuscript, we used 19 bioclimatic variables, which are often used in species distribution modeling and related ecological modeling techniques, from WorldClim to characterize the meteorological factors. For population, we believed it may have an indirect impact on the habitat suitability of *An. stephensi*, which was one of the aims of the manuscript, and this it was included into the model. For land use, we selected barren, cropland, forest, grassland and urban into the original manuscript. However, the share of forest areas in occurrence points was similar, we thus removed the share of forest areas. Even though our primary aim is to analyze the relationship between urban area and habitat suitability of *An. stephensi*, we included other land use types into the model to

simulate the habitat suitability for *An. stephensi* as accurately as possible. For elevation, since it was associated with bioclimatic variables, such as temperature (Figure S1), we excluded it. In addition to including as many variables (related with *An. stephensi*) as possible, we have also ensured the accuracy of the variable data to make sure the accuracy of model in the manuscript. We demonstrated the selection of covariates and the associated procedures in the 'Method' section. The text in Line 461-498 in the revision is as follows.

"The environmental factors associated with the breeding of *An. stephensi* were found from the literatures, for example, the monthly densities of *An. stephensi* adults and larvae were associated with precipitation, maximum and mean temperature[14]. We then summarized the elements affecting the four stages of *An. stephensi* breeding (Fig. 6), from which we obtained the environmental and human activities factors that may affect the breeding of *An. stephensi*.

We obtained monthly total precipitation, monthly maximum temperature and monthly minimum temperature from the WorldClim in 1992-2021[48]. We used the 'biovars' function in the R package 'dismo'[49] to estimated 19 bioclimatic variables for 1992-2021 using the monthly total precipitation, monthly maximum temperature and monthly minimum temperature. The spatial resolution is 2.5 minutes.

The land use dataset was collected from Land-Use Harmonization (LUH2)[50] and the spatial resolution was 0.25°, which is the unit quartile of the land use pattern at the grid scale. The land use patterns of LUH2 are more finely categorized, containing 14 land use patterns, such as forested primary land and urban land, and we have reclassified the land use patterns into five types based on the previous study[51], including barren, cropland, forest, grassland and urban land. The details about the reclassification of the land use are shown in Table S1. To eliminate interference, we finally included barren, cropland grassland and urban land into the study, and removed forest areas because the share of forest areas in occurrence points was similar. Since year-to-year changes in land use data were not obvious, we used the data of the first year of each phase to represent the land use of the phase, i.e., the land use data in 1992 for the first phase, the land use data in 2002 for the second phase and the land use data in 2012 for the third phase.

Population data was obtained from LandScan[52] and the spatial resolution is 30 arc-seconds. Since LandScan only provided data for 2000-2022, we assumed linear population growth to construct the linear algorithm to interpolate the data for 1992-1999 based on the previous study[46]. To facilitate calculations, we have resampled the data to a spatial resolution of 2.5 minutes.

Elevation data was collected from WorldClim[48] and the spatial resolution is 2.5 minutes. The build-up surface areas data was collected from European Commission[53] with 1km resolution, and updated every five years. We thus used the build-up surface areas data in 2000 for Phase I, 2010 for Phase II and 2020 for Phase III. The infrastructure investment and people living in slums were collected from World Bank[54] on the national scale. The infrastructure investment data was the total private

participation in infrastructure (PPI) investment in each country of the study area, and the people living in slums data was the proportion of people living in slums in cities. The details about all the data used in the study are shown in Table S2."

【Question 37 – Commented a37】

Line 382-383 "(Phase I, 1992-2001), second (Phase II, 2002-2011) and third (Phase III, 2012-2021) phase": What are these phases?

【Response 37】

Thank you very much for the comment and apologize for the lack of detailed method on this point in the manuscript. Regarding the division of phases, we considered two main reasons. First, since the previous study indicated that *An. stephensi* invaded Africa since 2012 (Ochomo et al., 2023), we used 2012 as a temporal cutoff point. Second, considering data availability, our study period was selected as 1992-2021. To maintain consistent temporal intervals, we divided the period into ten-year segments based on the previous study (Glidden et al., 2024). We then finally divided the study period into three phases: 1992-2001 (Phase I), 2002-2011 (Phase II) and 2012-2021 (Phase III). We have also revised it and added the details into the main text and the supplementary materials. The text in Line 410-414 in the revision is as follows.

"The study was conducted to analyze the impacts and mechanisms of urbanization on the habitat suitability of *An. stephensi* in West Asia, South Asia and Africa during 1992-2021. **We divided the period into three phases based on the invasion of *An. stephensi* and the temporal structure of key spatial datasets: 1992-2001 (Phase I), 2002-2011 (Phase II) and 2012-2021 (Phase III) (please see supplementary method 1 for details).**"

The text in supplementary materials is as follows.

"**Supplementary Method 1. Rationale for temporal stratification**

To examine how urbanization-related processes influence the habitat suitability of *An. stephensi*, we stratified the study period into three successive phases. This stratification reflects both the staged of invasion of *An. stephensi* and the temporal structure of key spatial datasets used in the study. Urbanization has been documented as a complicated process[1], for example, absolute population growth in the core city and thus the agglomeration as a whole and absolute population growth in the agglomeration, with growth being relatively concentrated on the suburban space[2]. These phases differ not only in the extent of urban land cover and population concentration, but also in the intensity of the built environment, which are relevant to mosquito breeding habitats. Therefore, we have divided the entire research phase into distinct stages. Due to the invasion of *An. stephensi* into Africa since 2012[3], we used 2012 as a dividing point in time series. On the other hand, the selected time windows align with the temporal resolution and continuity of datasets in the study. The

previous study[4] used 10-year intervals as dividing points, so we followed this approach and divided the time periods of the study into three phases (Phase I [1992-2001], Phase II [2002-2011] and Phase III [2012-2021])."

【*References*】

Glidden C K, Singleton A L, Chamberlin A. Climate and urbanization drive changes in the habitat suitability of Schistosoma mansoni competent snails in Brazil[J]. Nature Communications. 2024, 15(1): 4838.

Ochomo E O, Milanoi S, Abong'O B. Detection of Anopheles stephensi Mosquitoes by Molecular Surveillance, Kenya[J]. Emerg Infect Dis. 2023, 29(12): 2498-2508.

【Question 38 - Commented a38】

Line 390 "literatures": What was done with the literature? What were the search terms? How many sources were looked at?

【Response 38】

We searched for literatures on *An. stephensi* using the keyword "*Anopheles stephensi*", "*An. stephensi*", "*An. stephensi suitability habitat*", "*An. stephensi habitat*", "*Anopheles stephensi suitability habitat*" and "*Anopheles stephensi habitat*" in Web of Science, PubMed and Google Scholar. Since we required complete location and date for *An. Stephensi* occurrence, only six literatures (Faulde et al., 2014; Abubakr et al., 2022; Hemming-Schroeder and Ahmed, 2023; Teka et al., 2023; Meshesha Balkew, 2021; Al-Eryani et al., 2023) were effective. We then collect 202 records from the literatures. After merging data from GBIF and WHO, we obtained a total of 632 occurrence points by selecting data within the study area and removing duplicates. The distribution of *An. stephensi* record points is shown in Fig. S9. We have elaborated in the revision on the detailed literatures searching for occurrence points of *An. stephensi*, and the text in Line 435-443 in the revision is as follows. We have also added the access ability to retrieve the DOI of the literatures within the 'Data availability' section.

"We also searched for literatures on *An. stephensi* using the keyword "*Anopheles stephensi*", "*An. stephensi*", "*An. stephensi suitability habitat*", "*An. stephensi habitat*", "*Anopheles stephensi suitability habitat*" and "*Anopheles stephensi habitat*" in Web of Science, PubMed and Google Scholar, and six relevant literatures[20,41-45] were found, which recorded the complete locations and dates of *An. stephensi*. We then collecting a total of 202 records from the literatures. Finally, we merged the data from these three sources, removed duplicate, and obtained a total of 632 occurrence points in the study region (54 in Phase I, 132 in Phase II and 446 in Phase III). The distribution of occurrence points is shown in Fig. S9."

【*References*】

Abubakr M, Sami H, Mahdi I. The Phylodynamic and Spread of the Invasive Asian Malaria

Vectors, Anopheles stephensi, in Sudan[J]. Biology. 2022, 11(3): 409.
Al-Eryani S M, Irish S R, Carter T E. Public health impact of the spread of Anopheles stephensi in the WHO Eastern Mediterranean Region countries in Horn of Africa and Yemen: need for integrated vector surveillance and control[J]. Malaria Journal. 2023, 22(1): 187.
Faulde M K, Rueda L M, Khaireh B A. First record of the Asian malaria vector Anopheles stephensi and its possible role in the resurgence of malaria in Djibouti, Horn of Africa[J]. Acta tropica. 2014, 139: 39-43.
Hemming-Schroeder E, Ahmed A. Anopheles stephensi in Africa: vector control opportunities for cobreeding An. stephensi and Aedes arbovirus vectors[J]. Trends in parasitology. 2023, 39(2): 86-90.
Meshesha Balkew P M G Y. An update on the distribution, bionomics, and insecticide susceptibility of Anopheles stephensi in Ethiopia, 2018-2020[J]. Malaria Journal. 2021(20): 263.
Teka H, Golassa L, Medhin G. Trend analysis of malaria in urban settings in Ethiopia from 2014 to 2019[J]. Malaria Journal. 2023, 22(1): 235.

【Question 39 – Commented a39】

Line 389-392 "We obtained occurrence records of *An. Stephensi* from 1992 to 2021 through GBIF, WHO and literatures. Only on-site records with complete temporal metadata were retained, and 668 occurrence points were contained after removing duplicate records in the study (Figure S8).": What was done to quality check the georeferencing, GBIF lists *An. stephensi* as present in the USA as the georeferencing links to the museum where the specimen is held and not where it was captured. GBIF is not the best resource for vector data.

【Response 39】

We conducted quality checks on the coordinates sourced from GBIF. First, we retained only points with complete records of latitude and longitude coordinates and date. Then, we verified each point and removed those with obviously incorrect locations – such as points falling within oceans or coordinates inconsistent with national administrative boundaries. We also have added illustration about quality checks in GBIF data into the revision and the text in Line 430-435 is as followed.

"For data from GBIF and WHO, we only retain occurrence points with complete records containing date, latitude and longitude information. We examined these coordinates and excluded points in obviously incorrect locations, such as locating in the ocean or where the latitude and longitude coordinates did not align with national administrative regions. The amount of data from GBIF and WHO is 670 and 844, respectively."

【Question 40 – Commented a40】

Line 395-397 "which assumed that absent points were distributed in areas where the environment was different from that of the occurrence points.": This is potentially

problematic. Does it account for accessibility for sampling, sampling bias etc. How different is 'different'? Would this generate background in desert/poles?

【Response 40】

We think this is an excellent suggestion and agree with the data bias due to accessibility for sampling should be addressed. We have added a kernel density estimation (KDE) to the revision. Specifically, to account for sampling bias, for example sampling may be tend to target densely populated areas, we assigned probability weights to each grid cell using the kernel density estimation (KDE) to establish a sampling similar with the occurrence points. The results of KDE are shown in Fig. S10. We generated background points by using an environmental background based technique based on the reference (Chambault et al., 2021), which assumed that absent points were distributed in areas where the environment was different from that of the occurrence points. A principal component analysis (PCA) was used to obtain a two-dimensional environmental background, and we conducted a restricted convex hull, which the environment outside the restricted convex hull was considered to the unfavorable environment for *An. stephensi*. Combined with the probability on the grid cells from KDE, pseudo-absence points were randomly generated outside this convex hull with the same number of points as the occurrence points in three phases. These pseudo-absence points were reprojected back into the geographic space unfavorable to the emergence of *An. stephensi*. To improve the robustness of the results, we simulated 10 different sets of background points. We also illustrated the details about the method in the revision and the revised text in Line 444-459 is as follows.

"Due to the lack of true species absence data, most studies conducted pseudo-absence algorithms to generate background points[46,47]. We generated background points by using an environmental background based technique[47], which assumed that absent points were distributed in areas where the environment was different from that of the occurrence points. A principal component analysis (PCA) was used to obtain a two-dimensional environmental background, and we conducted a restricted convex hull, which the environment outside the restricted convex hull was considered to the unfavorable environment for *An. stephensi*. To account for sampling bias, for example sampling may be tend to target densely populated areas, we assigned probability weights to each grid cell using KDE to establish a sampling similar with the occurrence points. The results of KDE are shown in Fig. S10. Combined with the probability on the grid cells, pseudo-absence points were randomly generated outside this convex hull with the same number of points as the occurrence points in three phases. These pseudo-absence points were reprojected back into the geographic space unfavorable to the emergence of *An. stephensi*. To improve the robustness of the results, we simulated 10 different sets of background points."

Regarding the last two question you mentioned, this method ensures that the environment of background points differs significantly from that of occurrence points of *An. stephensi*, thereby satisfying the assumption that background sites are locations

where *An. stephensi* is absent. Meanwhile, we performed bias correction to ensure background points exhibit the same sampling bias as occurrence points, thereby minimizing the impact of accessibility constraints. Therefore, background points wound not be generated in inaccessible regions, such as deserts or poles.

【Reference】

Chambault, P. et al. A methodological framework to predict the individual and population-level distributions from tracking data. Ecography (Copenhagen). ***44,*** *766-777 (2021).*

【Question 41 – Commented a41】

Line 403 “unfavorable to the emergence”: How do you know it is unfavorable? How was this assessed?

【Response 41】

Based on the environment background based technique from the literature (Chambault et al., 2021), we first extracted environmental values, such as climate, population and land use from the occurrence points. We then used principal component analysis (PCA) for dimensionality reduction, generating a convex hull that represents the environment suitable for *An. stephensi*. Points outside this convex hull are unsuitable for *An. stephensi*. Meanwhile, we have also added correction for sampling bias into the method. We also provide a more detailed illustration of this method in the revision and the text in Line 444-459 is as followed.

“Due to the lack of true species absence data, most studies conducted pseudo-absence algorithms to generate background points[46,47]. We generated background points by using an environmental background based technique[47], which assumed that absent points were distributed in areas where the environment was different from that of the occurrence points. A principal component analysis (PCA) was used to obtain a two-dimensional environmental background, and we conducted a restricted convex hull, which the environment outside the restricted convex hull was considered to the unfavorable environment for *An. stephensi*. To account for sampling bias, for example sampling may be tend to target densely populated areas, we assigned probability weights to each grid cell using KDE to establish a sampling similar with the occurrence points. The results of KDE are shown in Fig. S10. Combined with the probability on the grid cells, pseudo-absence points were randomly generated outside this convex hull with the same number of points as the occurrence points in three phases. These pseudo-absence points were reprojected back into the geographic space unfavorable to the emergence of *An. stephensi*. To improve the robustness of the results, we simulated 10 different sets of background points.”

【Reference】

Chambault, P. et al. A methodological framework to predict the individual and population - level distributions from tracking data. Ecography (Copenhagen). 44, 766-777 (2021).

【Question 42 – Commented a42】

Line 411-412 “Since the WorldClim only provides data for 19 bioclimatic variables from 1970 to 2000”: There are probably other (and considerably better) datasets that cover the date range of your study? What else was considered?

【Response 42】

Thank you for your comment. Indeed, there are other long-term meteorological datasets, such as ERA5 reanalysis data from European Centre for Medium-Range Weather Forecasts (ECMWF). Initially, we considered this dataset because it spans a long period, stating from 1940 to present, and includes hourly temperature, precipitation, relative humidity and other indicators. However, the WorldClim datasets provides 19 bioclimatic variables, widely used in species distribution modeling and related ecological modeling techniques. Moreover, the algorithm that estimated 19 bioclimatic variables through monthly maximum temperature, minimum temperature and precipitation applied is publicly documented by WorldClim. Meanwhile, while species distribution certainly varies across different times of the day – meaning hourly temperature and precipitation affect species – hourly time stamps are not recorded in the occurrence data. Therefore, monthly meteorological indicators are more meaningful for our research. We have also revised the main text to present this data more clearly and the text in Line 467-471 in the revision is as followed.

“We obtained monthly total precipitation, monthly maximum temperature and monthly minimum temperature from the WorldClim in 1992-2021[48]. We used the ‘biovars’ function in the R package ‘dismo’[49] to estimated 19 bioclimatic variables for 1992-2021 using the monthly total precipitation, monthly maximum temperature and monthly minimum temperature. The spatial resolution is 2.5 minutes.”

【Question 43 – Commented a43】

Line 421 “barren, forest, grassland, urban land and cropland”: Why include covariates that are not relevant to the mosquito species?

【Response 43】

These are the major types of land use, and incorporating them into the study is considered essential for constructing a robust model. First, the reason that included all land use types into the study is to eliminate interference and define the baseline ecological niche. For example, barren regions (such as bare rock, desert, and saline-alkali land) lack standing water bodies – an absolute prerequisite for mosquito larvae – and are clearly unsuitable areas. Including them in the model helps the algorithm more clearly learn “what constitutes absolute unsuitability,” thereby more precisely defining the species’ potential distribution boundaries. The second reason is to

mitigate sampling bias. The occurrence data likely completely avoids some land use types. If not introduced, the model will mistakenly assume “all sampled environments are potentially suitable,” failing to distinguish between “unsampled” and “unsuitable.” For example, barren areas, as explicitly unsuitable environments, provide crucial negative sample information. Therefore, we included covariates that are not relevant to the mosquito species. In fact, due to the similarity of forest area in occurrence data, we did not include forest area into the BRT model, which stated in the text in the original manuscript. We have also revised it and the text in Line 472-480 is as follows.

“The land use dataset was collected from Land-Use Harmonization (LUH2)[50] and the spatial resolution was 0.25°, which is the unit quartile of the land use pattern at the grid scale. The land use patterns of LUH2 are more finely categorized, containing 14 land use patterns, such as forested primary land and urban land, and we have reclassified the land use patterns into five types based on the previous study[51], **including barren, cropland, forest, grassland and urban land.** The details about the reclassification of the land use are shown in Table S1. **To eliminate interference, we finally included barren, cropland grassland and urban land into the study, and removed forest areas because the share of forest areas in occurrence points was similar.**”

【Question 44 – Commented a44】

Line 428-429 “Since LandScan only provided data for 2000-2022, we interpolated to complete the data for 1992-1999.”: Again, there are datasets that do cover the time period

【Response 44】

Thank you for the comment. We searched a lot of authority data, such as WorldPop and GPW. However, all of this data is since. The data from WorldPop is from 2000 to 2020, which is not updated in 2021. The data from GPW is also since 2000 and updated every five years. Therefore, we finally used the population data from LandScan.

【Question 45 – Commented a45】

Line 432 “Other urbanization indexes”: These are not spatial though? How were they integrated into the data?

【Response 45】

We apologize for the lack of key information in the study. To further analyze the mechanisms by which urbanization affects the habitat suitability of *An. stephensi*, we selected infrastructure investment and people living in slums. However, the two data was available at the grid level, so we obtained both from the World Bank. In the

mechanism analysis, we conducted our analysis at the national level. Specifically, we calculated the total habitat suitability index of *An. stphensi* (Suitability) in the study area. We also used the national total population (POP) and estimated the total urban area (URB) based on grid area in each country. Average temperature (TEM) and precipitation (PRE) indexes were included in the PLS-PM model as control variables. In addition, we calculated the total national value of built-up areas, and combined with infrastructure investment, formed the variable "Construction" (BLD). We have added it into the 'Partial least-squares path (PLS-PM) model' part in 'Method' part. The text in Line 559-565 in the revision is as followed.

"To analyze relationships at the national scale, we calculated the total habitat suitability index of *An. stphensi* (Suitability) in the study area. We also used the national total population (POP) and estimated the total urban area (URB) based on grid area in each country. Average temperature (TEM) and precipitation (PRE) indexes were included in the PLS-PM model as control variables. In addition, we calculated the total national value of built-up areas, and combined with infrastructure investment, which represents construction activities, formed the variable "Construction indexes" (BLD). "

【Question 46 – Commented a46】

Line 439 "rural, urban and high-density urban regions": This might be a bit tricky - determining the "boundaries" between these categories can be quite challenging in some cases. For instance, urban agriculture and unplanned urban areas may be difficult to distinguish - will your approach be able to adequately cover these aspects?

【Response 46】

We agree that determining the "boundaries" between these categories is a bit tricky. In the revision, we adopted the existing classification of rural, urban and high-density urban from the literature (Schiavina et al., 2025). The 1 km^2 grid cells are divided in the two hierarchical levels and the categories in the first hierarchical level are rural, urban cluster and urban center. In addition to classifying based on population density within grid cells, this method aggregates the total population of surrounding grid cells (such as 4-connectivity) for analysis, thereby reducing misclassification of urban agriculture and unplanned urban areas. The details about each category are shown in Table S3. However, we also recognized that any classification method may contain errors, and issue of imprecise identification may arise in these particular regions (such as urban agriculture and unplanned urban areas). We have discussed this in the 'Discussion' section.

The text about the details of the method in Line 500-513 in the revision is as followed.

"We used the settlement typologies delineated and classified from European Commission[55] to analyze the changes in the suitable habitat of *An. stephensi* during urbanization. The dataset defined the spatial entities at two hierarchical levels and the

1km^2 grids are divided into three categories of the first hierarchical level: rural, urban cluster and urban center grid cell (please see supplementary methods and Table S3 for details)[56], and generated the settlement classification layers every five years since 1975[55], which the resolution is 1km. To standardize the resolution, we resampled the grids to achieve a 2.5-minute resolution by using the nearest-neighbor method. Due to the several sub-levels of three categories in the data, we further consolidated it into three major categories: rural, urban and high-density urban for analysis. Furthermore, we selected the data in 2000 for Phase I, the data in 2010 for Phase II and the data in 2020 for Phase III. The details about the classification methods and the approaches to verify the validation of this dataset are illustrated in the supplementary materials (Fig. S11). The grid-scale classification is shown in Fig. S12."

The text in 'Discussion part in Line 313-317 in the revision is as followed.

"Despite the reliability of the urban classification, the delineation in the boundaries between urban and rural areas is hard. Because the population in these areas may be close to the urban threshold, and some environments may be quite similar to the habitats of *An. stephensi* in the urban areas. The field researches in the future are required to conduct in-depth analysis of *An. stephensi* larval condition in these areas."

The text in supplementary materials is as follows.

"The classification dataset of urbanization is the "GHS-WUP DEGURBA settlement layers (GHS_WUP_DEGURBA_GLOBE_R2025A)" from the previous study[5]. The dataset used cell clusters population size, population and built-up area to delineate settlements[6]. The dataset defined the spatial entities at two hierarchical levels and the 1km^2 grids are divided into three categories of the first hierarchical level: rural, urban cluster and urban center grid cell[6]. Specifically, an urban center is defined as a contiguous cluster of grid cells (based on 4-connectivity) where the permanent land has a population density of at least 1,500 inhabitants per km^2. This cluster should contain a total of 50,000 or more inhabitants. Its boundaries are smoothed using 3*3 conditional majority filtering, and any holes smaller than 15 km^2 within the cluster are filled. An urban cluster is formed by contiguous grid cells connected through 8-neighbor adjacency. It should have a density of at least 300 inhabitants per km2 on permanent land and contain a total population of 5,000 or more. The rural grid cells are all the other cells that do not belong to an urban cluster, most of which will have a density below 300 inhabitants per km^2. The second hierarchical levels contain eight categories: Urban center grid cell, dense urban cluster grid cell, semi-dense urban cluster grid cell, suburban or peri-urban grid cell, rural cluster grid cell, low density grid cell, very low density rural grid cell and water grid cell. We merged the secondary categories to derive three classifications (rural, urban and high-density urban) for analysis. Table S3 shows the introduction of categories in the first and second hierarchical levels.

We also validated the settlement of the dataset. We calculated the share of urban areas across the three classifications. We found that the highest average share of urban areas was in high-density urban areas (5.188% [95%CI: 5.004%-5.373%] in Phase I, 5.832%

[95%CI: 5.657-6.007%] in Phase III and 6.512% [95%CI: 6.345-6.680%] in Phase III). The second highest average share of urban land was in the urban areas (1.196% [95%CI: 1.172-1.220%] in Phase I, 1.305% [95%CI: 1.283-1.328%] in Phase II and 1.472% [95%CI: 1.451-1.493%] in Phase III). The rural areas had the lowest average share of urban areas (0.127% [95%CI: 0.126-0.128%] in Phase I, 0.161% [95%CI: 0.160-0.162%] in Phase II and 0.205% [95%CI: 0.204-0.206%] in Phase III). The share of urban areas in rural, urban and high-density urban regions in the three phases is shown in Fig. S11. It demonstrates that there is a relationship between urban area and this classification, and also validates the rationality of the classification dataset.”

【References】

Schiavina M, Melchiorri M, Pesaresi M. 2025. GHS-WUP-DEGURBA R2025A – GHS-WUP DEGURBA settlement layers, application of the Degree of Urbanisation methodology (stage I) to GHS-WUP-POP R2025A, multitemporal (1975-2100)European Commission, Joint Research Centre (JRC).

【Question 47 – Commented a47】

Line 446-447 “results of definition in rural, urban and high-density urban regions”: Was anything done to ground truth these? As this is intrinsic to the study, it would be good to see how effective these classifications were - and if they are effective, they would be very useful for other modelers trying to do similar work

【Response 47】

We agree that the classification of rural, urban and high-density urban is intrinsic to the study. Since our study did not aim to categorizing rural, urban and high-density urban areas, we adopted existing research dataset that classify these three categories. We analyzed the relationship between share of urban land and the classification data to verify the validation of the classification in the previous study (Schiavina et al., 2025). We found that the highest average share of urban areas was in high-density urban areas (5.188% [95%CI: 5.004%-5.373%] in Phase I, 5.832% [95%CI: 5.657-6.007%] in Phase III and 6.512% [95%CI: 6.345-6.680%] in Phase III). The second highest average share of urban land was in the urban areas (1.196% [95%CI: 1.172-1.220%] in Phase I, 1.305% [95%CI: 1.283-1.328%] in Phase II and 1.472% [95%CI: 1.451-1.493%] in Phase III). The rural areas had the lowest average share of urban areas (0.127% [95%CI: 0.126-0.128%] in Phase I, 0.161% [95%CI: 0.160-0.162%] in Phase II and 0.205% [95%CI: 0.204-0.206%] in Phase III). The share of urban areas in rural, urban and high-density urban areas in the three phases is shown in Fig. S11. It demonstrates that there is a relationship between urban area and this classification. Therefore, we considered this dataset to be reliable in terms of classification in rural, urban and high-density urban areas. The text in Line 500-513 in the revision is as follows.

“We used the settlement typologies delineated and classified from European

Commission[55] to analyze the changes in the suitable habitat of *An. stephensi* during urbanization. The dataset defined the spatial entities at two hierarchical levels and the 1km^2 grids are divided into three categories of the first hierarchical level: rural, urban cluster and urban center grid cell (please see supplementary methods and Table S3 for details)[56], and generated the settlement classification layers every five years since 1975[55], which the resolution is 1km. To standardize the resolution, we resampled the grids to achieve a 2.5-minute resolution by using the nearest-neighbor method. Due to the several sub-levels of three categories in the data, we further consolidated it into three major categories: rural, urban and high-density urban for analysis. Furthermore, we selected the data in 2000 for Phase I, the data in 2010 for Phase II and the data in 2020 for Phase III. The details about the classification methods and the approaches to verify the validation of this dataset are illustrated in the supplementary materials (Fig. S11). The grid-scale classification is shown in Fig. S12."

The text in supplementary materials is as followed.

"The classification dataset of urbanization is the "GHS-WUP DEGURBA settlement layers (GHS_WUP_DEGURBA_GLOBE_R2025A)" from the previous study[5]. The dataset used cell clusters population size, population and built-up area to delineate settlements[6]. The dataset defined the spatial entities at two hierarchical levels and the 1km^2 grids are divided into three categories of the first hierarchical level: rural, urban cluster and urban center grid cell[6]. Specifically, an urban center is defined as a contiguous cluster of grid cells (based on 4-connectivity) where the permanent land has a population density of at least 1,500 inhabitants per km^2. This cluster should contain a total of 50,000 or more inhabitants. Its boundaries are smoothed using 3*3 conditional majority filtering, and any holes smaller than 15 km^2 within the cluster are filled. An urban cluster is formed by contiguous grid cells connected through 8-neighbor adjacency. It should have a density of at least 300 inhabitants per km2 on permanent land and contain a total population of 5,000 or more. The rural grid cells are all the other cells that do not belong to an urban cluster, most of which will have a density below 300 inhabitants per km^2. The second hierarchical levels contain eight categories: Urban center grid cell, dense urban cluster grid cell, semi-dense urban cluster grid cell, suburban or peri-urban grid cell, rural cluster grid cell, low density grid cell, very low density rural grid cell and water grid cell. We merged the secondary categories to derive three classifications (rural, urban and high-density urban) for analysis. Table S3 shows the introduction of categories in the first and second hierarchical levels.

We also validated the settlement of the dataset. We calculated the share of urban areas across the three classifications. We found that the highest average share of urban areas was in high-density urban areas (5.188% [95%CI: 5.004%-5.373%] in Phase I, 5.832% [95%CI: 5.657-6.007%] in Phase III and 6.512% [95%CI: 6.345-6.680%] in Phase III). The second highest average share of urban land was in the urban areas (1.196% [95%CI: 1.172-1.220%] in Phase I, 1.305% [95%CI: 1.283-1.328%] in Phase II and 1.472% [95%CI: 1.451-1.493%] in Phase III). The rural areas had the lowest average share of urban areas (0.127% [95%CI: 0.126-0.128%] in Phase I, 0.161% [95%CI:

0.160-0.162%] in Phase II and 0.205% [95%CI: 0.204-0.206%] in Phase III). The share of urban areas in rural, urban and high-density urban regions in the three phases is shown in Fig. S11. It demonstrates that there is a relationship between urban area and this classification, and also validates the rationality of the classification dataset."

【References】

Schiavina M, Melchiorri M, Pesaresi M. 2025. GHS-WUP-DEGURBA R2025A – GHS-WUP DEGURBA settlement layers, application of the Degree of Urbanisation methodology (stage I) to GHS-WUP-POP R2025A, multitemporal (1975-2100)European Commission, Joint Research Centre (JRC).

【Question 48 – Commented a48】

Line 455-456 "habitats of *An. Stephensi* during 1992-2021.": Did the occurrence data combine adult and larval data? were they temporally partitioned?

【Response 48】

Yes, the occurrence data concluded adult and larval date and the occurrence point was divided temporally. Specifically, we consolidated the timing of occurrence points into three phases. For instance, if a point was recorded in 2016, it was grouped into Phase II (2012-2021). Furthermore, environmental data was combined with the occurrence points into the BRT model, which were temporally partitioned. We have also revised the manuscript and the text in Line 531-532 is as followed.

"The data were temporally partitioned based on the three phases and then concluded into the BRT model."

【Question 49 – Commented a49】

Line 464 "verify the accuracy of the BRT models": This does not verify the accuracy of the model - it assesses how well the model uses the data given - if the data is poor, the model is poor, even if the AUC indicates good fit.

【Response 49】

We agree that if the data is poor, the model is poor, even if the AUC indicates good fit. Therefore, we applied multiple approaches to control data quality, ensuring to the greatest extent possible that the model accurately reflected the actual habitat suitability of *An. stephensi*. First of all, we performed quality control on the occurrence data. The occurrence data was collected by GBIF, WHO and literatures. For data from GBIF and WHO, we only retain occurrence points with complete records containing date, latitude and longitude information. We examined these coordinates and excluded points in obviously incorrect locations, such as locating in the ocean or where the latitude and longitude coordinates did not align with national

administrative regions. The amount of data from GBIF and WHO is 670 and 844, respectively. We also searched for literatures on *An. stephensi* using the keyword "*Anopheles stephensi*", "*An. stephensi*", "*An. stephensi* suitability habitat", "*An. stephensi* habitat", "*Anopheles stephensi* suitability habitat" and "*Anopheles stephensi* habitat" in Web of Science, PubMed and Google Scholar, and six relevant literatures (Faulde et al., 2014; Abubakr et al., 2022; Hemming-Schroeder and Ahmed, 2023; Teka et al., 2023; Meshesha Balkew, 2021; Al-Eryani et al., 2023) were founded, which recorded the complete locations and dates of *An. stephensi*. We then collecting a total of 202 records from the literatures. Finally, we merged the data from these three sources, removed duplicate, and obtained a total of 632 occurrence points in the study region (54 in Phase I, 132 in Phase II and 446 in Phase III). The text in Line 429-443 in the revision is as followed.

"We obtained occurrence global records of *An. stephensi* from 1992 to 2021 through GBIF[39], WHO[40] and literatures[20,41-45]. For data from GBIF and WHO, we only retain occurrence points with complete records containing date, latitude and longitude information. We examined these coordinates and excluded points in obviously incorrect locations, such as locating in the ocean or where the latitude and longitude coordinates did not align with national administrative regions. The amount of data from GBIF and WHO is 670 and 844, respectively. We also searched for literatures on *An. stephensi* using the keyword "*Anopheles stephensi*", "*An. stephensi*", "*An. stephensi suitability habitat*", "*An. stephensi habitat*", "*Anopheles stephensi suitability habitat*" and "*Anopheles stephensi habitat*" in Web of Science, PubMed and Google Scholar, and six relevant literatures[20,41-45] were found, which recorded the complete locations and dates of *An. stephensi*. We then collecting a total of 202 records from the literatures. Finally, we merged the data from these three sources, removed duplicate, and obtained a total of 632 occurrence points in the study region (54 in Phase I, 132 in Phase II and 446 in Phase III). The distribution of occurrence points is shown in Fig. S9."

Second, we used kernel density estimation (KDE) to address sampling bias in occurrence points during generating background points. We assigned probability weights to each grid cell using the kernel density estimation (KDE) to establish a sampling similar with the occurrence points. The results of KDE are shown in Figure S10. Furthermore, we used the environmental background based technique from the literature (Chambault et al., 2021) to generate background points based on KDE. The technique assumed that absent points were distributed in areas where the environment was different from that of the occurrence points. A principal component analysis (PCA) was used to obtain a two-dimensional environmental background, and we conducted a restricted convex hull, which the environment outside the restricted convex hull was considered to the unfavorable environment for *An. stephensi*. Combined with the probability on the grid cells, pseudo-absence points were randomly generated outside this convex hull with the same number of points as the occurrence points in three phases. These pseudo-absence points were reprojected back into the geographic space unfavorable to the emergence of *An. stephensi*. To improve

the robustness of the results, we simulated 10 different sets of background points. The results in the revision were the average outputs of the 10 sets of background points. We also elaborated on this method in detail in Line 444-459 and the text in the revision is as follows.

"Due to the lack of true species absence data, most studies conducted pseudo-absence algorithms to generate background points[46,47]. We generated background points by using an environmental background based technique[47], which assumed that absent points were distributed in areas where the environment was different from that of the occurrence points. A principal component analysis (PCA) was used to obtain a two-dimensional environmental background, and we conducted a restricted convex hull, which the environment outside the restricted convex hull was considered to the unfavorable environment for *An. stephensi*. To account for sampling bias, for example sampling may be tend to target densely populated areas, we assigned probability weights to each grid cell using KDE to establish a sampling similar with the occurrence points. The results of KDE are shown in Fig. S10. Combined with the probability on the grid cells, pseudo-absence points were randomly generated outside this convex hull with the same number of points as the occurrence points in three phases. These pseudo-absence points were reprojected back into the geographic space unfavorable to the emergence of *An. stephensi*. To improve the robustness of the results, we simulated 10 different sets of background points."

However, even if we adopted the method to correct sampling bias, it would be difficult to obtain unbiased data. Therefore, we have expanded the discussion of limitations to include an explanation of data bias in the manuscript. The text in Line 397-401 in the revision is as followed.

"First, it is recognized that a perfect and unbiased sampling regime is challenging. While our methodological approach aims to mitigate this known data bias, some inherent biases associated with the original occurrence data may persist. Future study incorporating systematic field surveillance across diverse environments would be critical."

【Reference】

Abubakr M, Sami H, Mahdi I. The Phylodynamic and Spread of the Invasive Asian Malaria Vectors, Anopheles stephensi, in Sudan[J]. Biology. 2022, 11(3): 409.

Al-Eryani S M, Irish S R, Carter T E. Public health impact of the spread of Anopheles stephensi in the WHO Eastern Mediterranean Region countries in Horn of Africa and Yemen: need for integrated vector surveillance and control[J]. Malaria Journal. 2023, 22(1): 187.

Chambault, P. et al. A methodological framework to predict the individual and population - level distributions from tracking data. Ecography (Copenhagen). 44, 766-777 (2021).

Faulde M K, Rueda L M, Khaireh B A. First record of the Asian malaria vector Anopheles stephensi and its possible role in the resurgence of malaria in Djibouti, Horn of Africa[J]. Acta tropica. 2014, 139: 39-43.

Hemming-Schroeder E, Ahmed A. Anopheles stephensi in Africa: vector control opportunities for cobreeding An. stephensi and Aedes arbovirus vectors[J]. Trends in parasitology. 2023, 39(2): 86-

90.
Meshesha Balkew P M G Y. An update on the distribution, bionomics, and insecticide susceptibility of Anopheles stephensi in Ethiopia, 2018-2020[J]. Malaria Journal. 2021(20): 263.
Teka H, Golassa L, Medhin G. Trend analysis of malaria in urban settings in Ethiopia from 2014 to 2019[J]. Malaria Journal. 2023, 22(1): 235.

【Question 50 – Commented a50】

Line 465-468 "To further analyze the spatial differences in the effects of the influencing factors on *An. Stephensi* habitat, the study controlled the changes of individual factors by sensitivity tests to estimate the changes in the suitable habitat of *An. Stephensi* based on a previous study [118]": I'm not sure I understand what this was doing - please clarify.

【Response 50】

We aim to analyze the distribution of *An. stephensi* under the influence of individual factors. For example, we analyzed how the spatial distribution of habitat suitability of *An. stephensi* changed when only urban land shifts occurred while other factors (i.e. climatic factors) remain constant. It enables to understand which areas' habitat suitability is influenced by specific factors. The method is similar to reconstructing the spatial distribution of habitat suitability. Specifically, we controlled for changes in only single factor while keeping all others constant to analyze the differences in the habitat suitability of *An. stephensi*. For example, we maintained constant population, climate and other land use type parameters from Phase I into the BRT models when projecting urban-driven changes in suitable habitats for *An. stephensi* during the Phase II and Phase III by using the Phase I as a baseline period. Meanwhile, we standardized habitat suitability values for each phase using the min-max transformation. We have revised it in Line 541-550 and the text in the revision is as followed.

"To further analyze the spatial differences in the effects of the different factors on *An. stephensi* habitat, the study controlled the changes of individual factors by sensitivity tests to estimate the changes in the suitable habitat based on a previous study[59]. Specifically, we controlled for changes in only single factor while keeping all others constant to analyze the differences in the habitat suitability of *An. stephensi*. For example, we maintained constant population and land use parameters from Phase I into the BRT models when projecting climate-driven changes in suitable habitats for *An. stephensi* during Phase II and Phase III by using the Phase I as a baseline period. Meanwhile, we standardized habitat suitability values for each phase using the min-max transformation."

【Question 51 – Commented a51】

Line 468 "the first phase": There is no information about these phases except a classification in the first paragraph of the methods - what was the reason for these

phases - what was the reasoning for classifying them as you have done?

【Response 51】

We apologize for the lack of detailed method on this point in the manuscript. Regarding the division of phases, we considered two main reasons. First, since the previous study indicated that *An. stephensi* invaded Africa since 2012 (Ochomo et al., 2023), we used 2012 as a temporal cutoff point. Second, considering data availability, our study period was selected as 1992-2021. To maintain consistent temporal intervals, we divided the period into ten-year segments based on the previous study (Glidden et al., 2024). We then finally divided the study period into three phases: 1992-2001 (Phase I), 2002-2011 (Phase II) and 2012-2021 (Phase III). We have also revised it and added the details into the main manuscript and supplementary materials. The text in Line 410-414 in the revision is as follows.

"The study was conducted to analyze the impacts and mechanisms of urbanization on the habitat suitability of *An. stephensi* in West Asia, South Asia and Africa during 1992-2021. **We divided the period into three phases based on the invasion of *An. stephensi* and the temporal structure of key spatial datasets: 1992-2001 (Phase I), 2002-2011 (Phase II) and 2012-2021 (Phase III) (please see supplementary method 1 for details).**"

The text in supplementary materials is as follows.

"**Supplementary Method 1. Rationale for temporal stratification**

To examine how urbanization-related processes influence the habitat suitability of *An. stephensi*, we stratified the study period into three successive phases. This stratification reflects both the staged of invasion of *An. stephensi* and the temporal structure of key spatial datasets used in the study. Urbanization has been documented as a complicated process[1], for example, absolute population growth in the core city and thus the agglomeration as a whole and absolute population growth in the agglomeration, with growth being relatively concentrated on the suburban space[2]. These phases differ not only in the extent of urban land cover and population concentration, but also in the intensity of the built environment, which are relevant to mosquito breeding habitats. Therefore, we have divided the entire research phase into distinct stages. Due to the invasion of *An. stephensi* into Africa since 2012[3], we used 2012 as a dividing point in time series. On the other hand, the selected time windows align with the temporal resolution and continuity of datasets in the study. The previous study[4] used 10-year intervals as dividing points, so we followed this approach and divided the time periods of the study into three phases (Phase I [1992-2001], Phase II [2002-2011] and Phase III [2012-2021])."

【References】

Glidden C K, Singleton A L, Chamberlin A. Climate and urbanization drive changes in the habitat suitability of Schistosoma mansoni competent snails in Brazil[J]. Nature Communications. 2024,

15(1): 4838.
Ochomo E O, Milanoi S, Abong'O B. Detection of Anopheles stephensi Mosquitoes by Molecular Surveillance, Kenya[J]. Emerg Infect Dis. 2023, 29(12): 2498-2508.

【Question 52 – Commented a52】

Line 470 "climate-driven changes in suitable habitats": Real or predicted?

【Response 52】

This is the predicted climate-driven changes in suitable habitats of *An. stephensi*.

【Question 53 – Commented a53】

Line 476-477 "the national scale": Across Africa? Or the whole of the *An. stephensi* range?

【Response 53】

We analyzed the mechanisms by which urbanization affected the habitat suitability of *An. stephensi* on the national scale in overall study area, Africa, West and South Asia. Specifically, we constructed three PLS-PM models in the overall study area, Africa, West and South Asia, respectively, and we also compared the differences between results in the overall study area, Africa, West and South Asia. The revised text (red bold font) in Line 552-558 is as follows.

"The study used the PLS-PM model to analyze the mechanisms by which urbanization affected habitat suitability of *An. stephensi* on the national scale, **and we also analyzed separately in the overall study area, Africa, West and South Asia.** PLS-PM model can be applied to analyze complex relationships between modules that contains both latent and explicit variables[60]. Latent variables area theoretical concepts that cannot be directly measured, while explicit variables are concrete data indicators that can be directly observed or measured."

【Question 54 – Commented a54】

Line 478 "The latent variables contained night factors": Sorry - getting confused here – what was the latent variable here? The list given includes measurable variables - are these what were used to calculate the latent variable??

【Response 54】

We sincerely thank for the valuable comment and apologize the lack details about the method. PLS-PM model contains latent and explicit variables. Latent variables area

theoretical concepts that cannot be directly measured, while explicit variables are concrete data indicators that can be directly observed or measured. In the study, the latent variables were population (POP), urban land (URB), construction (BLD), people living in slums in cities (SLM), temperature indexes (TEM) and precipitation indexes (PRE). BIO1-BIO11 are the explicit variables of the latent temperature variable, and BIO12-BIO19 are the explicit variables of the latent precipitation variable. The total population is the explicit variable of POP. The urban area is the explicit variable of URB. The share of people living in slums in cities is the explicit variable of SLM. The built-up area and infrastructure investment are the explicit variables of BLD. In the model's computations, we applied the absolute standardized loadings index (loadings>0.7) to select explicit variables in the latent variables. the final explicit variables contained within latent variables are shown in Table S4. We have also added it into the manuscript and the text in Line 552-578 in the revision is as follows.

"The study used the PLS-PM model to analyze the mechanisms by which urbanization affected habitat suitability of *An. stephensi* on the national scale, and we also analyzed separately in the overall study area, Africa, West and South Asia. PLS-PM model can be applied to analyze complex relationships between modules that contains both latent and explicit variables[60]. **Latent variables area theoretical concepts that cannot be directly measured, while explicit variables are concrete data indicators that can be directly observed or measured.**

To analyze relationships at the national scale, we calculated the total habitat suitability index of *An. stphensi* (Suitability) in the study area. We also used the national total population (POP) and estimated the total urban area (URB) based on grid area in each country. Average temperature (TEM) and precipitation (PRE) indexes were included in the PLS-PM model as control variables. In addition, we calculated the total national value of built-up areas, and combined with infrastructure investment, which represents construction activities, formed the variable "Construction indexes" (BLD).

The latent variables contained six factors, including population (POP), urban land (URB), construction indexes (BLD), people living in slums in cities (SLM), temperature indexes (TEM) and precipitation indexes (PRE). BIO1-BIO11 are the explicit variables of the latent temperature variable, and BIO12-BIO19 are the explicit variables of the latent precipitation variable. The absolute standardized loadings index was used to select the explicit variables that best reflect the latent variables (loadings>0.7) and the final explicit variables contained within latent variables are shown in Table S4. The goodness-of-fit (GOF) can evaluate the merits of the model, and band resampling (for 1000 times) can explain the significance of each path of the model[60]. We considered all latent variables in the initial models but included the latent variables for which the path coefficient could pass through the significance test ($p<0.05$). The PLS-PM was processed in R version 4.4.1 and the conceptual model is shown in Fig. S14."

Supplementary Table S4. The latent variables contained within explicit variables in all the study area, Africa and South and West Asia.

**The latent variables**	**The study area**	**Africa**	**Asia**
Population (POP)	Population	Population	Population
Urban (URB)	The area of urban land	The area of urban land	The area of urban land
Construction (BLD)	The build-up areas<br>Infrastructure investment	The build-up areas<br>Infrastructure investment	The build-up areas<br>Infrastructure investment
People living in slums (SLM)	Share of People living in slums in cities	Share of People living in slums in cities	Share of People living in slums in cities
Temperature index (TEM)	BIO4<br>BIO10	BIO4<br>BIO5<br>BIO8<br>BIO10	BIO8
Precipitation index (PRE)	BIO15	BIO12<br>BIO13<br>BIO14<br>BIO15<br>BIO17	BIO13<br>BIO14<br>BIO16<br>BIO18

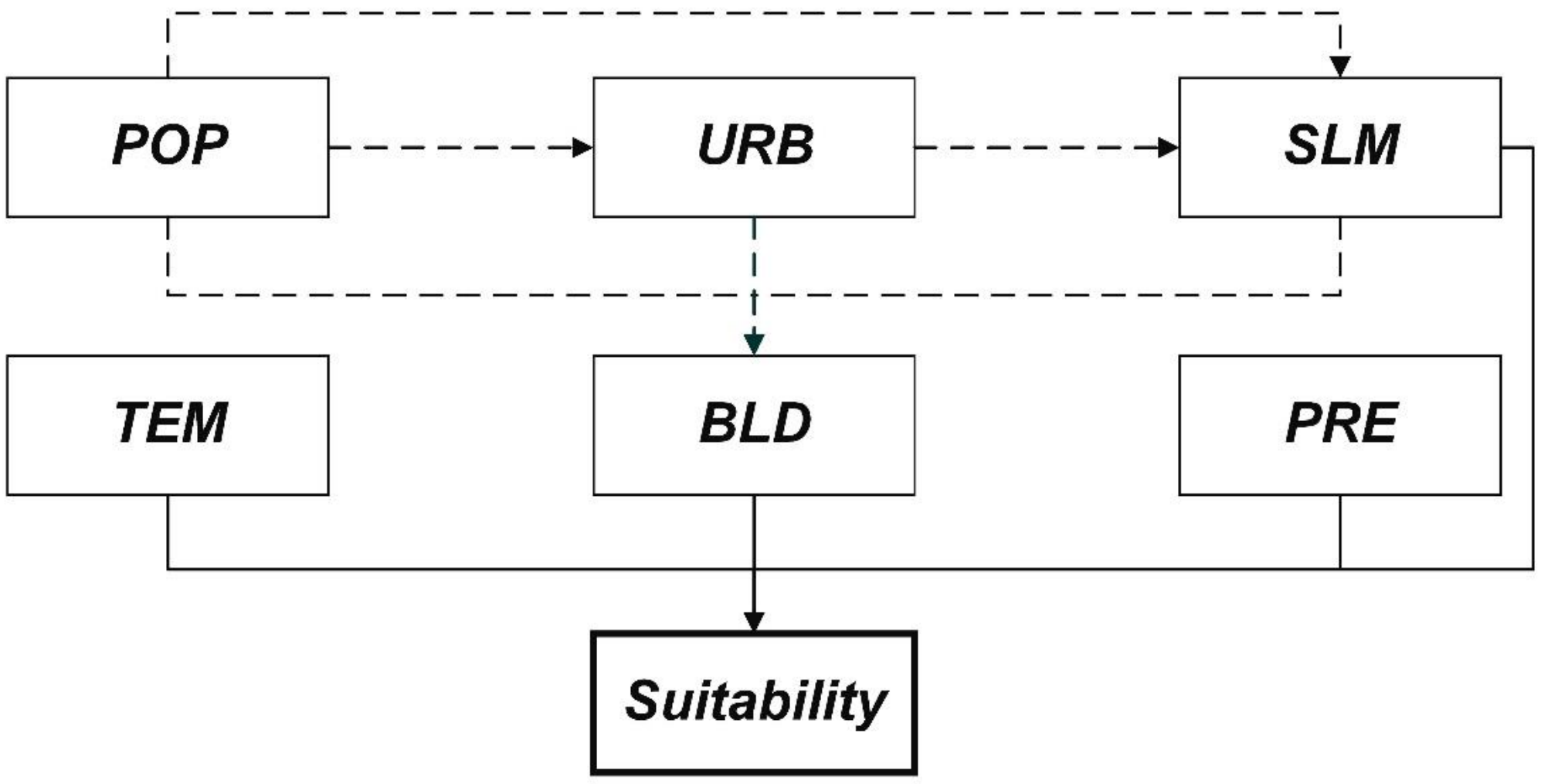

Supplementary Fig. S14. The conceptual model of PLS-PM model in the study. POP: population. URB: share of urban land. SLM: people living in slums in cities. BLD: Construction indexes. TEM: temperature indexes. PRE: precipitation indexes. Suitability: habitat suitability of *An. stephensi*.

【Question 55 – Commented a55】

Line 490-494 "We obtained data from GBIF (https://www.gbif.org/), WHO (https://apps.who.int/malaria/maps/threats/#/download), WorldClim (https://www.worldclim.org/data/monthlywth.html#google_vignette), LUH2 (https://luh.umd.edu/), LandScan (https://landscan.ornl.gov) and World Bank (https://data.worldbank.org/).": What was done to check the quality of the data?

(georeferencing, species ID) what is the DOI for the actual dataset rather than a generic GBIF link, what was the date range? How were duplicates dealt with?

【Response 55】

We checked the quality of data through the approach below. We obtained occurrence global records of *An. stephensi* from 1992 to 2021 through GBIF, WHO and literatures. For data from GBIF and WHO, we only retain occurrence points with complete records containing date, latitude and longitude information. We examined these coordinates and excluded points in obviously incorrect locations, such as locating in the ocean or where the latitude and longitude coordinates did not align with national administrative regions. The amount of data from GBIF and WHO is 670 and 844, respectively. We also searched for literatures on *An. stephensi* using the keyword "*Anopheles stephensi*", "*An. stephensi*", "*An. stephensi suitability habitat*", "*An. stephensi habitat*", "*Anopheles stephensi suitability habitat*" and "*Anopheles stephensi habitat*" in Web of Science, PubMed and Google Scholar, and six relevant literatures were found, which recorded the complete locations and dates of *An. stephensi*. We then collecting a total of 202 records from the literatures. Finally, we merged the data from these three sources, removed duplicate, and obtained a total of 632 occurrence points in the study region (54 in Phase I, 132 in Phase II and 446 in Phase III). The text about the quality checking of occurrence data is in Line 429-443 in the revision (below).

"We obtained occurrence global records of *An. stephensi* from 1992 to 2021 through GBIF[39], WHO[40] and literatures[20,41-45]. For data from GBIF and WHO, we only retain occurrence points with complete records containing date, latitude and longitude information. We examined these coordinates and excluded points in obviously incorrect locations, such as locating in the ocean or where the latitude and longitude coordinates did not align with national administrative regions. The amount of data from GBIF and WHO is 670 and 844, respectively. We also searched for literatures on *An. stephensi* using the keyword "*Anopheles stephensi*", "*An. stephensi*", "*An. stephensi suitability habitat*", "*An. stephensi habitat*", "*Anopheles stephensi suitability habitat*" and "*Anopheles stephensi habitat*" in Web of Science, PubMed and Google Scholar, and six relevant literatures[20,41-45] were found, which recorded the complete locations and dates of *An. stephensi*. We then collecting a total of 202 records from the literatures. Finally, we merged the data from these three sources, removed duplicate, and obtained a total of 632 occurrence points in the study region (54 in Phase I, 132 in Phase II and 446 in Phase III). The distribution of occurrence points is shown in Fig. S9."

We have added the DOI into the 'data availability' section and the text in Line 580-587 in the revision is as followed.

"We obtained data from GBIF (https://doi.org/10.15468/dl.fh95wu), WHO (https://apps.who.int/malaria/maps/threats/#/download), literatures (https://doi/org/10.1186/s12936-023-04545-y, https://doi.org/10.1016/j.actatropica.2014.06.016, https://doi.org/0.3

390/biology11030409, https://doi.org/10.1016/j.pt.2022.11.011, https://doi.org/10.1186/s12936-023-04656-6, and https://doi.org/10.1186/s12936-021-03801-3), WorldClim (https://www.worldclim.org/data/monthlywth.html#google_vignette), LUH2 (https://luh.umd.edu/), LandScan (https://landscan.ornl.gov) and World Bank (https://data.worldbank.org/)."

【Question 56 – Commented a56】

All species names in the references also need to be in italics.

【Response 56】

We thank the reviewer for the reminder. We have changed all species names in the references to italics.

Finally, thanks again for reviewing our paper and for providing your useful suggestions.
