# Supplementary material for: Urbanization development characteristics coincide with elevated Anopheles stephensi habitat suitability
Source: Commun Earth Environ. 2026 Jul 22;7(1):893. doi: 10.1038/s43247-026-03833-0 (PMC13759296; doi:10.1038/s43247-026-03833-0)
Supplement: Supplementary file 2 [file 43247_2026_3833_MOESM2_ESM.pdf]

# Urbanization development characteristics coincide with elevated Anopheles stephensi habitat suitability

Yelin Sun[1], Weihang Liu[2], Qinmei Han[3], Haoqiang Ji[1], Meng Shang[1], Xiaoxu Wang[1], Zhenxu Wang[4], Wenqin Liang[5], Qiyong Liu[1,*]

[1]National Key Laboratory of Intelligent Tracking and Forecasting for Infectious Diseases, National Institute for Communicable Disease Control and Prevention, Chinese Center for Disease Control and Prevention, Beijing, Beijing China.

[2]School of government, Nanjing University, Nanjing, Jiangsu China.

[3]National climate center, China Meteorological Administration, Beijing, China.

[4]Shandong Center for Disease Control and Prevention; Shandong Provincial Academy of Preventive Medicine, Jinan, Shandong China.

[5]Vector Surveillance Division, Laboratory Center, Guizhou Center for Disease Control and Prevention, Guiyang, Guizhou China.

***Corresponding author: liuqiyong@icdc.cn**

# Supplementary materials

**Supplementary Method 1. Rationale for temporal stratification**

To examine how urbanization-related processes influence the habitat suitability of *An. stephens*i, we stratified the study period into three successive phases. This stratification reflects both the stages of invasion of *An. stephensi* and the temporal structure of key spatial datasets used in the study. Urbanization has been documented as a complex process[1], for example, absolute population growth in the core city and thus the agglomeration as a whole and absolute population growth in the agglomeration, with growth being relatively concentrated on the suburban space[2]. These phases differ not only in the extent of urban land cover and population concentration, but also in the intensity of the built environment, which are relevant to mosquito breeding habitats. Therefore, we have divided the entire research phase into distinct stages. Due to the invasion of *An. stephensi* into Africa since 2012[3], we used 2012 as a dividing point in time series. On the other hand, the selected time windows align with the temporal resolution and continuity of datasets in the study. The previous study[4] used 10-year intervals as dividing points, so we followed this approach and divided the study period into three phases (Phase I [1992-2001], Phase II [2002-2011] and Phase III [2012-2021]).

**Supplementary Method 2. The classification method of urbanization in spatial raster datasets**

The classification dataset of urbanization is the "GHS-WUP DEGURBA settlement layers (GHS_WUP_DEGURBA_GLOBE_R2025A)" from the previous study[5]. The dataset used cell cluster population size, population and built-up area to delineate settlements[6]. The dataset defined the spatial entities at two hierarchical levels and the 1km$^2$ grids are divided into three categories of the first hierarchical level: rural, urban cluster and urban center grid cell[6]. Specifically, an urban center is defined as a contiguous cluster of grid cells (based on 4-connectivity) where the permanent land area has a population density of at least 1,500 inhabitants per km$^2$. This cluster should contain a total of 50,000 or more inhabitants. Its boundaries are smoothed using 3*3 conditional majority filtering, and any holes smaller than 15 km$^2$ within the cluster are filled. An urban cluster is formed by contiguous grid cells connected through 8-neighbor adjacency. It should have a density of at least 300 inhabitants per km$^2$ on permanent land and contain a total population of 5,000 or more. The rural grid cells are all the other cells that do not belong to an urban cluster, most of which will have a density below 300 inhabitants per km$^2$. The second hierarchical levels contain eight categories: Urban center grid cell, dense urban cluster grid cell, semi-dense urban cluster grid cell, suburban or peri-urban grid cell, rural cluster grid cell, low density grid cell, very low density rural grid cell and water grid cell. We merged the secondary categories to derive three classifications (rural, urban and high-density urban) for analysis. Table S3 shows the introduction of categories in the first and second hierarchical levels.

We also validated the settlement classification of the dataset. We calculated the share of urban areas across the three classifications. We found that the highest average share of urban areas was in high-density urban areas (5.188% [95%CI: 5.004%-5.373%] in Phase I, 5.832% [95%CI: 5.657-6.007%] in Phase II and 6.512% [95%CI: 6.345-6.680%] in Phase III). The second highest average share of urban land was in the urban areas (1.196% [95%CI: 1.172-1.220%] in Phase I, 1.305% [95%CI: 1.283-1.328%] in Phase II and 1.472% [95%CI: 1.451-1.493%] in Phase III). The rural areas had the lowest average share of urban areas (0.127% [95%CI: 0.126-0.128%] in Phase I, 0.161% [95%CI: 0.160-0.162%] in Phase II and 0.205% [95%CI: 0.204-0.206%] in Phase III). The share of urban areas in rural, urban and high-density urban regions in the three phases is shown in Fig. S12. It demonstrates that there is a relationship between urban area and this classification, and also validates the rationality of the classification dataset.

**Supplementary Table S1. The data resources in the study.** The features were considered in the study. We analyzed the correlations between factors, and those with correlations greater than 0.75 were removed. The complete results of the Spearman's correlations between factors are shown in Figure S1. The other indexes (the build-up surface areas, infrastructure investment and population living in slums) were not included in BRT model. These variables were therefore not included into the Spearman's correlations.

| Type | Factor | Definition | Spatial/temporal resolution | Source | Correlated with |
|---|---|---|---|---|---|
| Climate | BIO1 | Annual mean temperature | 2.5 minutes/ 10-year average | WorldClim | BIO6, BIO9, BIO11, DEM |
| | BIO2 | Mean monthly temperature range (Mean of monthly [max temperature – min temperature]) | | | None |
| | BIO3 | Is thermality (BIO2/BIO7) | | | BIO4, BIO7 |
| | BIO4 | Temperature seasonality (standard deviation ×100) | | | BIO3, BIO7 |
| | BIO5 | Max temperature of warmest month | | | BIO10 |
| | BIO6 | Min temperature of coldest month | | | BIO1, BIO7, BIO11 |
| | BIO7 | Temperature annual range (BIO5-BIO6) | | | BIO3, BIO4, BIO6 |
| | BIO8 | Mean temperature of wettest quarter | | | None |
| | BIO9 | Mean temperature of driest quarter | | | BIO1 |
| | BIO10 | Mean temperature of warmest quarter | | | BIO5 |
| | BIO11 | Mean temperature of coldest quarter | | | BIO1, BIO6 |
| | BIO12 | Annual precipitation | | | BIO13, BIO16, BIO18 |
| | BIO13 | Precipitation of wettest month | | | BIO12, BIO16, BIO18 |
| | BIO14 | Precipitation of driest month | | | BIO17 |
| | BIO15 | Precipitation seasonality (coefficient of variation) | | | None |
| | BIO16 | Precipitation of wettest quarter | | | BIO12, BIO13, BIO18 |
| | BIO17 | Precipitation of | | | BIO14 |

| | | | | | |
|---|---|---|---|---|---|
| | | driest quarter | | | |
| | BIO18 | Precipitation of warmest quarter | | | BIO12, BIO13, BIO16 |
| | BIO19 | Precipitation of coldest quarter | | | None |
| Land use | Barren | Share of barren | 0.25° /10-year average | LUH2 | None |
| | Cropland | Share of cropland | | | None |
| | Forest | Share of forest | | | None |
| | Grassland | Share of grassland | | | None |
| | Urban | Share of urban land | | | None |
| Population | Population | Total population | 1km/10-year average | LandScan | None |
| Elevation | Elevation | Elevation | 2.5 minutes/ 30-year average | WorldClim | BIO1 |
| Settlement | Settlement | The classification in rural, urban and high-density urban | 1km | European Commission | None |
| The build-up surface areas | The build-up surface areas | The build-up surface areas | 1km | European Commission | None |
| Others | Infrastructure investment | Total private participation in infrastructure (PPI) investment | Country/ 10-year total | World Bank | None |
| | Population living in slums | Proportion of people living in slums in cities | | | |

**Supplementary Table S2. The reclassification of the type of land use from LUH2.** Our reclassification of land use types was based on the previous study[7].

| Code in LUH2 | Land Use in LUH2 | Reclassification |
|---|---|---|
| primf<br>secdf | forested primary land<br>potentially forested secondary land | Forest |
| primn<br>secdn | non-forested primary land<br>potentially non-forested secondary land | Barren |
| pastr<br>range | managed pasture<br>rangeland | Grassland |
| urban | urban land | Urban |
| c3ann<br>c3per<br>c4ann<br>c4per<br>c3nfx | c3 annual crops<br>c3 perennial crops<br>c4 annual crops<br>c4 perennial crops<br>c3 nitrogen-fixing crops | Cropland |

**Supplementary Table S3. The latent variables contained within explicit variables in all the study area, Africa and South and West Asia.**

| The latent variables | The study area | Africa | Asia |
|---|---|---|---|
| Population (POP) | Population | Population | Population |
| Urban (URB) | The area of urban land | The area of urban land | The area of urban land |
| Construction (BLD) | The build-up areas<br>Infrastructure investment | The build-up areas<br>Infrastructure investment | The build-up areas<br>Infrastructure investment |
| People living in slums (SLM) | Share of People living in slums in cities | Share of People living in slums in cities | Share of People living in slums in cities |
| Temperature index (TEM) | BIO4<br>BIO10 | BIO4<br>BIO5<br>BIO8<br>BIO10 | BIO8 |
| Precipitation index (PRE) | BIO15 | BIO12<br>BIO13<br>BIO14<br>BIO15<br>BIO17 | BIO13<br>BIO14<br>BIO16<br>BIO18 |

**Supplementary Table S4. The classification of rural, urban and high-density urban.** The dataset is obtained from the previous study[6].

| Class in original data | Sub-level class in original data (Code) | Definition | Class in the study |
|---|---|---|---|
| Urban center | Urban center grid cell (30) | An urban center consists of contiguous grid cells (4-connectivity cluster) with a density of at least 1,500 inhabitants per $km^2$ of permanent land, and has at least 50,000 inhabitants in the cluster with smoothed boundaries (3-by-3 conditional majority filtering12) and $<15$ $km^2$ holes filled. | High-density urban |
| Urban cluster | Dense urban cluster grid cell (23) | A Dense Urban Cluster consists of contiguous cells (4-connectivity cluster) with a density of at least 1,500 in habitants per km2 of permanent land and has at least 5,000 and less than 50,000 in habitants in the cluster. | Urban |
| | Semi-dense urban cluster grid cell (22) | A semi-dense urban cluster consists of contiguous (using four-point contiguity) grid cells within a urban cluster (level 1) at least 2 km away13 from a dense urban cluster or an urban center with a density of at least 900 inhabitants per km2 and a collective population of at least 2,500 people | |
| | Suburban or peri-urban grid cell (21) | All the other cells that belong to an Urban Cluster but are not part of an Urban Centre, Dense Urban Cluster or a Semi-dense Urban Cluster | |

| | | | |
|---|---|---|---|
| Rural | Rural cluster grid cell (13) | A Rural Cluster consists of contiguous cells (8-connectivity cluster) with a density of at least 300 inhabitants per $km^2$ of permanent land and has at least 500 and less than 5,000 inhabitants in the cluster. | Rural |
| | Low density rural grid cell (12) | Rural grid cells with a density of at least 50 inhabitants per km2 of permanent land and are not part of a Rural Cluster. | |
| | Very low density rural grid cell (11) | Cells with a density of less than 50 inhabitants per km2 of permanent land. | |
| Water grid cells | Water grid cell (10) | All the cells with more than 0.5 share covered by permanent surface water that are not populated nor built. | Other |

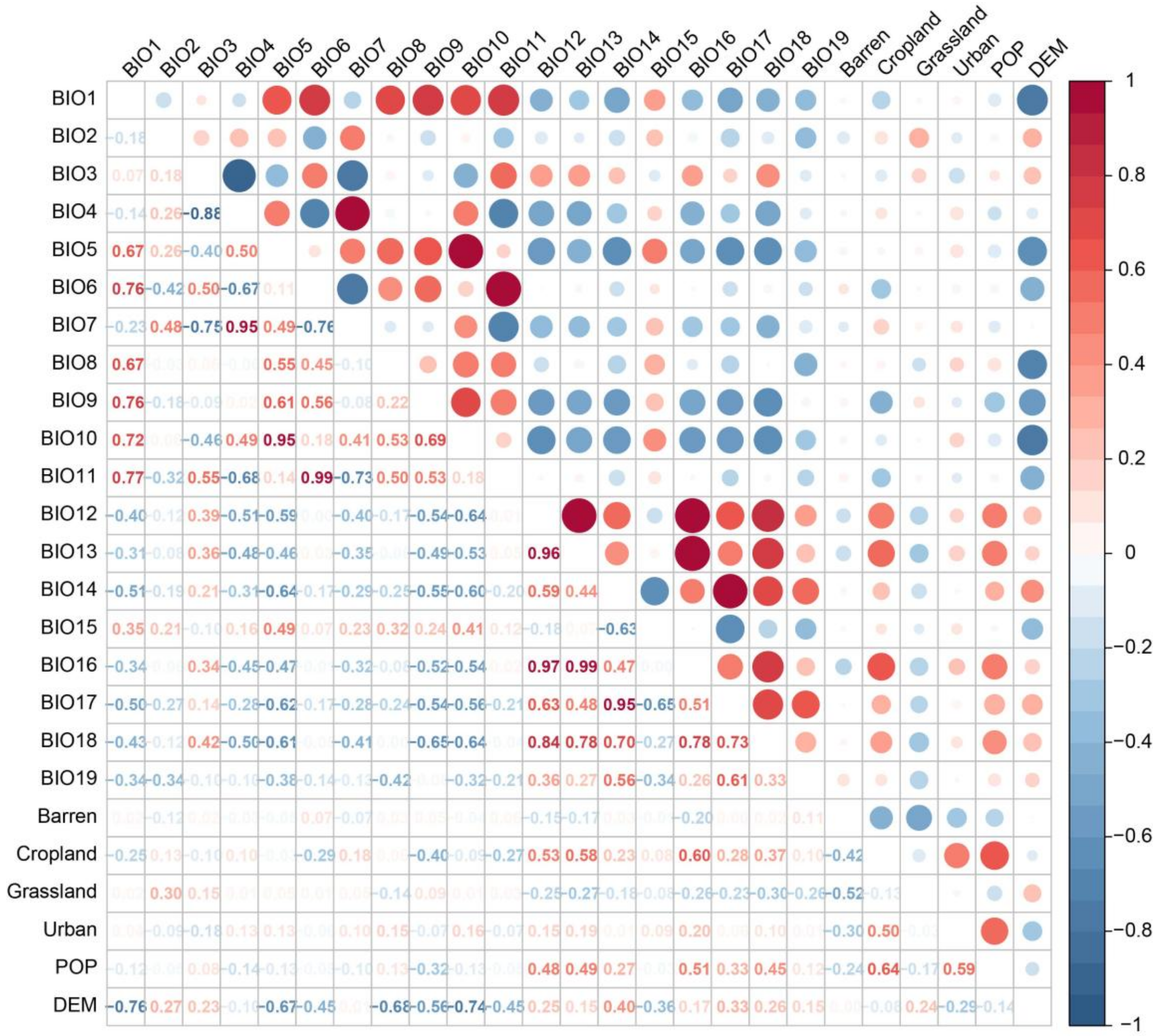

**Supplementary Fig. S1. The Spearman's correlations between factors.** We finally selected 14 factors into BRT model, including BIO1, BIO2, BIO4, BIO8, BIO10, BIO13, BIO15, BIO17, BIO19, barren, cropland, grassland, urban and population. Meanwhile, we did not take the share of forest into account since the share of forest at all *An. stephensi* occurrence points were similar. BIO1: annual mean temperature. BIO2: mean monthly temperature range. BIO3: is thermality (BIO2/BIO7). BIO4: temperature seasonality. BIO5: max temperature of warmest month. BIO6: min temperature of coldest month. BIO7: Temperature annual range. BIO8: mean temperature of wettest quarter. BIO9: mean temperature of driest quarter. BIO10: mean temperature of warmest quarter. BIO11: mean temperature of coldest quarter. BIO12: annual precipitation. BIO13: precipitation of wettest month. BIO14: precipitation of driest month. BIO15: precipitation seasonality. BIO16: precipitation of wettest quarter. BIO17: precipitation of driest quarter. BIO18: precipitation of warmest quarter. BIO19: precipitation of coldest quarter. Barren: share of barren. Cropland: share of cropland. Grassland: share of grassland. Urban: share of urban land. POP: population. DEM: elevation.

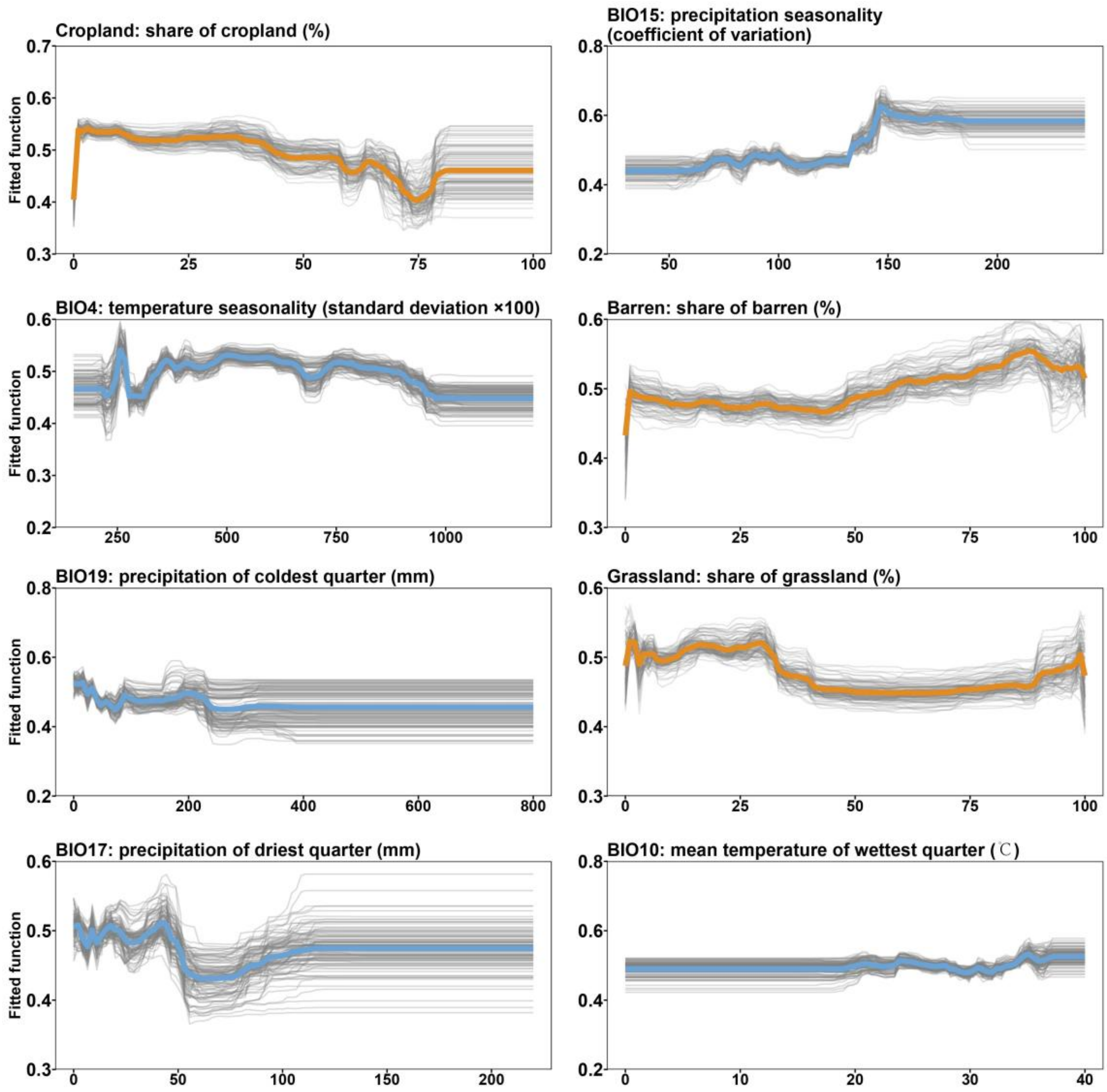


**Supplementary Fig. S2. The impact of other factors on the habitat suitability of *An. stephensi*.** Fig. S2 is the panel of the response curves of the habitat suitability to the variables except the top six variables in the manuscript. The solid black lines are the average of 100 repetitions, while the grey lines are the results of individual runs. The details about the other factors are illustrated in Supplementary Table S2.

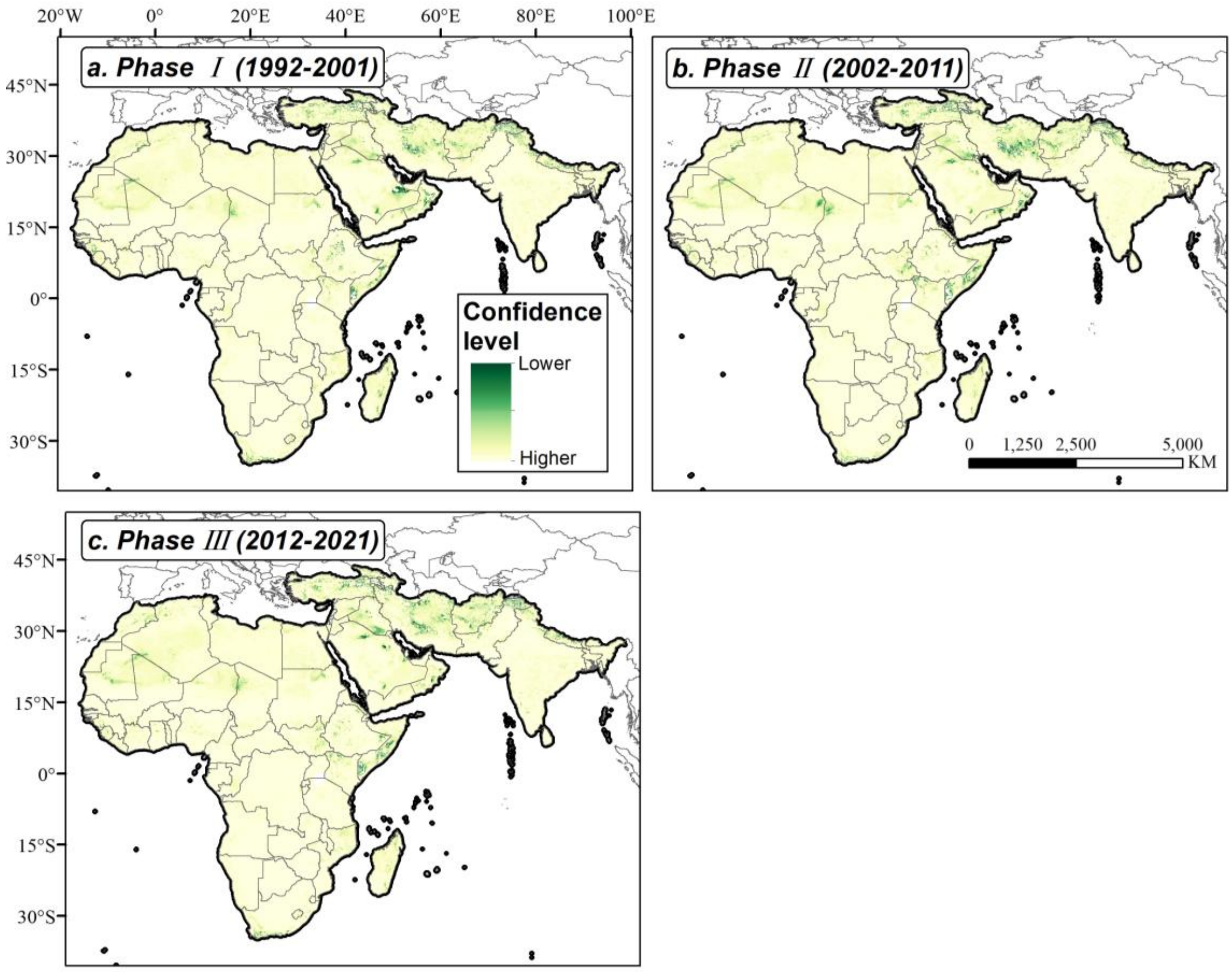


**Supplementary Fig. S3. The coefficient of variation map in the three phases. a-c** the coefficient of variation estimated per pixel across 100 simulations of the BRT model. The value is the ratio of standard deviation to mean, indicating the reliability of the predicted results of the BRT model. The higher value reflects a greater variability around the mean, indicating lower confidence in the mean value. The light green (lower value) represents higher confidence and the dark green (higher value) represents lower confidence.

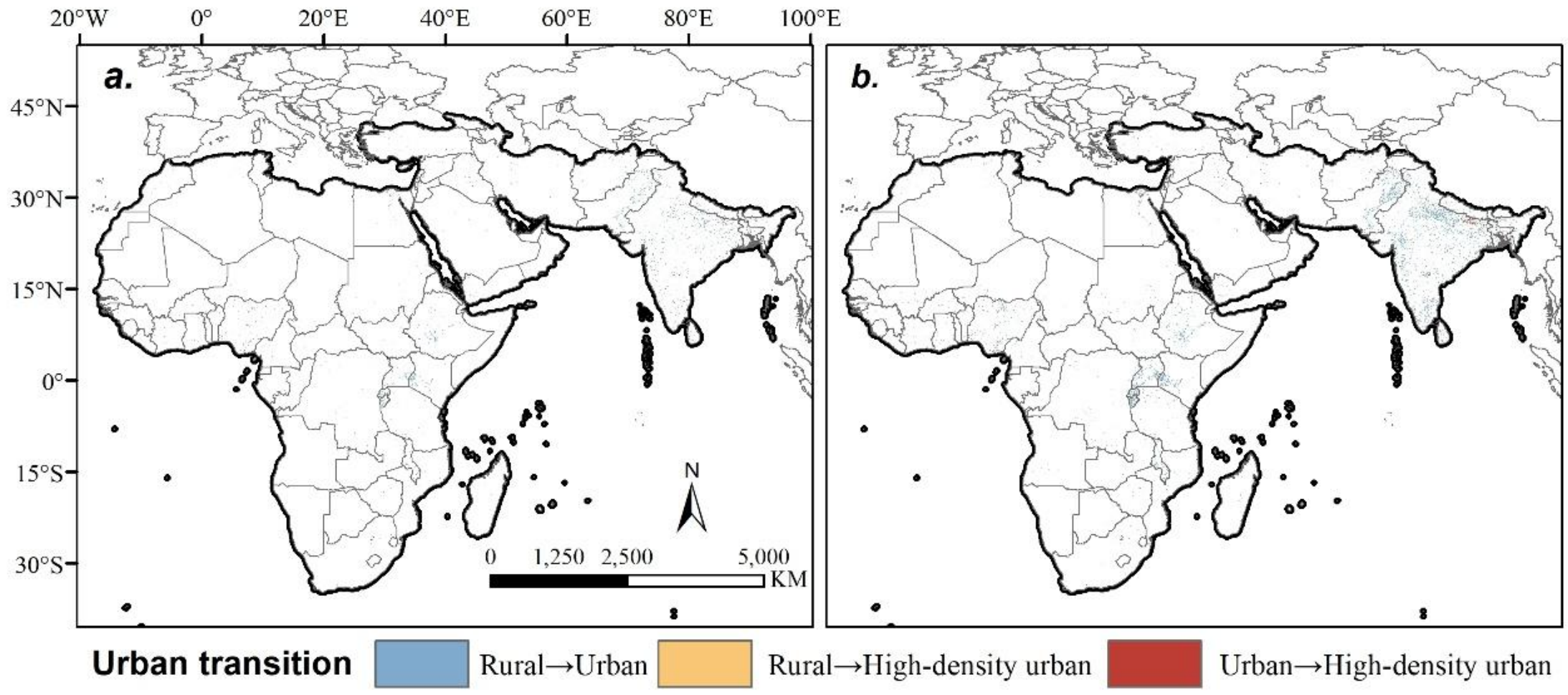


**Supplementary Fig. S4. The changes between rural, urban and high-density urban in Phase II and Phase III.** Using Phase I as the baseline, we calculated the transitions from rural to urban or high-density urban, and urban was replaced by high-density urban in Phase II (a) and Phase III (b).

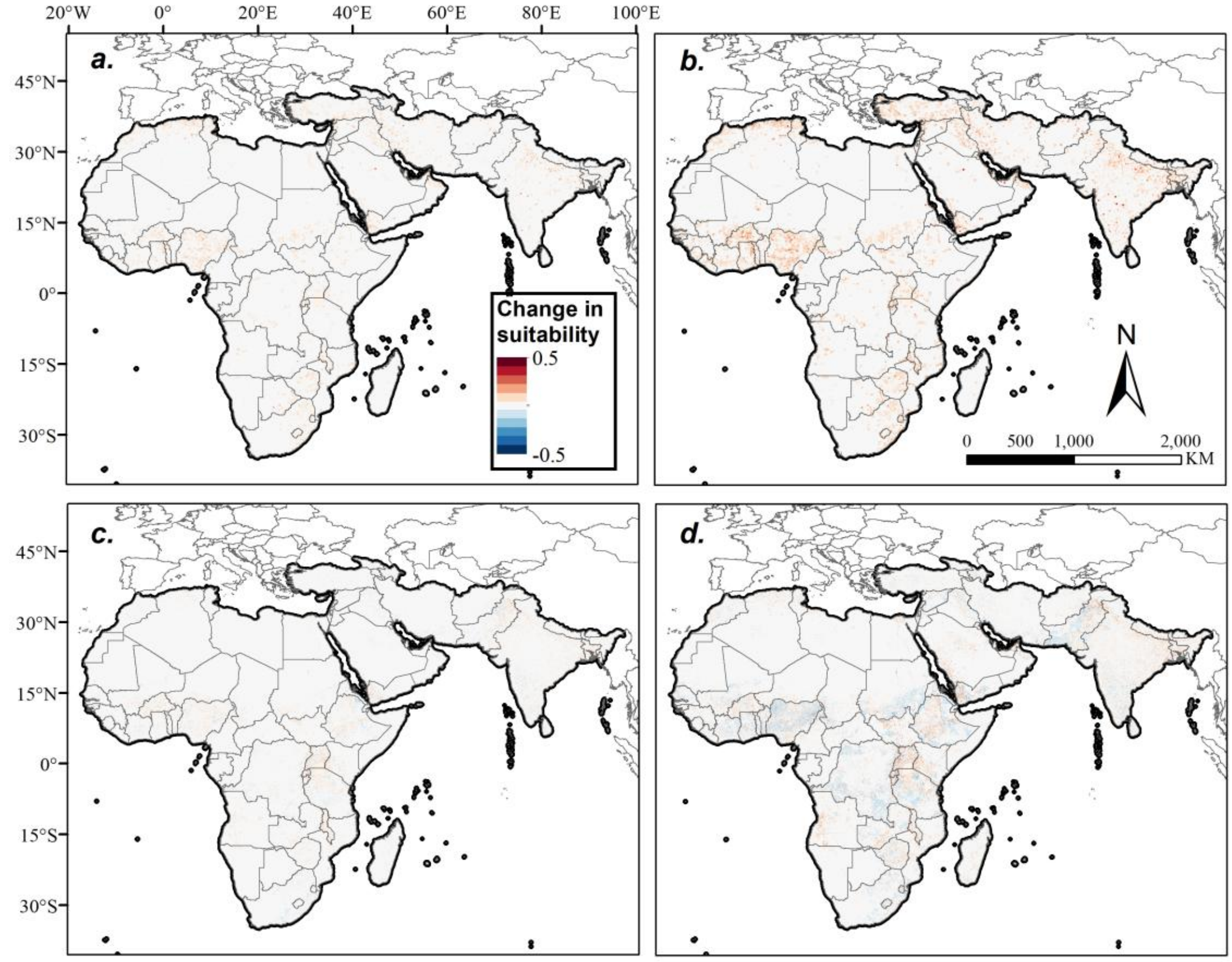


**Supplementary Fig. S5. The habitat suitability of *An. stephensi* only affected by share of urban land and population.** The baseline is Phase I and kept the values of other factors in Phase II and III consistent with Phase I to estimate the habitat suitability driven only by urban land (a and b) or population (c and d). The changes in habitat suitability in Phase II and III were the differences in values between Phase II and III and the baseline, respectively.

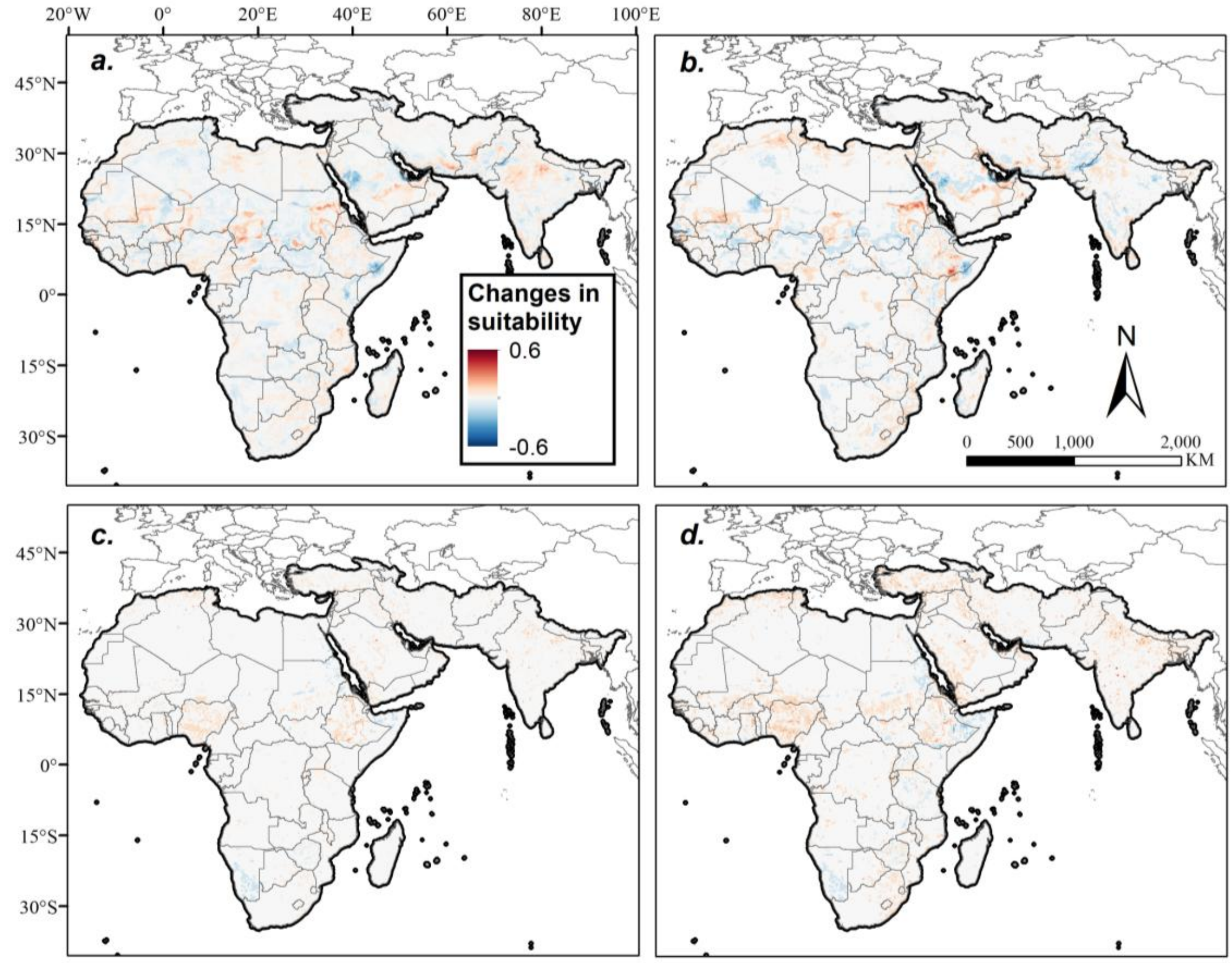


**Supplementary Fig. S6. The habitat suitability of *An. stephensi* only affected by climatic factors and other land-use types.** We used Phase I as baseline and kept the values of other factors in Phase II and III consistent with Phase I to estimate the habitat suitability only affected by climatic factors (a and b) or other land-use types (c and d). The changes in habitat suitability in Phase II and III were the differences in values between Phase II and III and baseline, respectively.

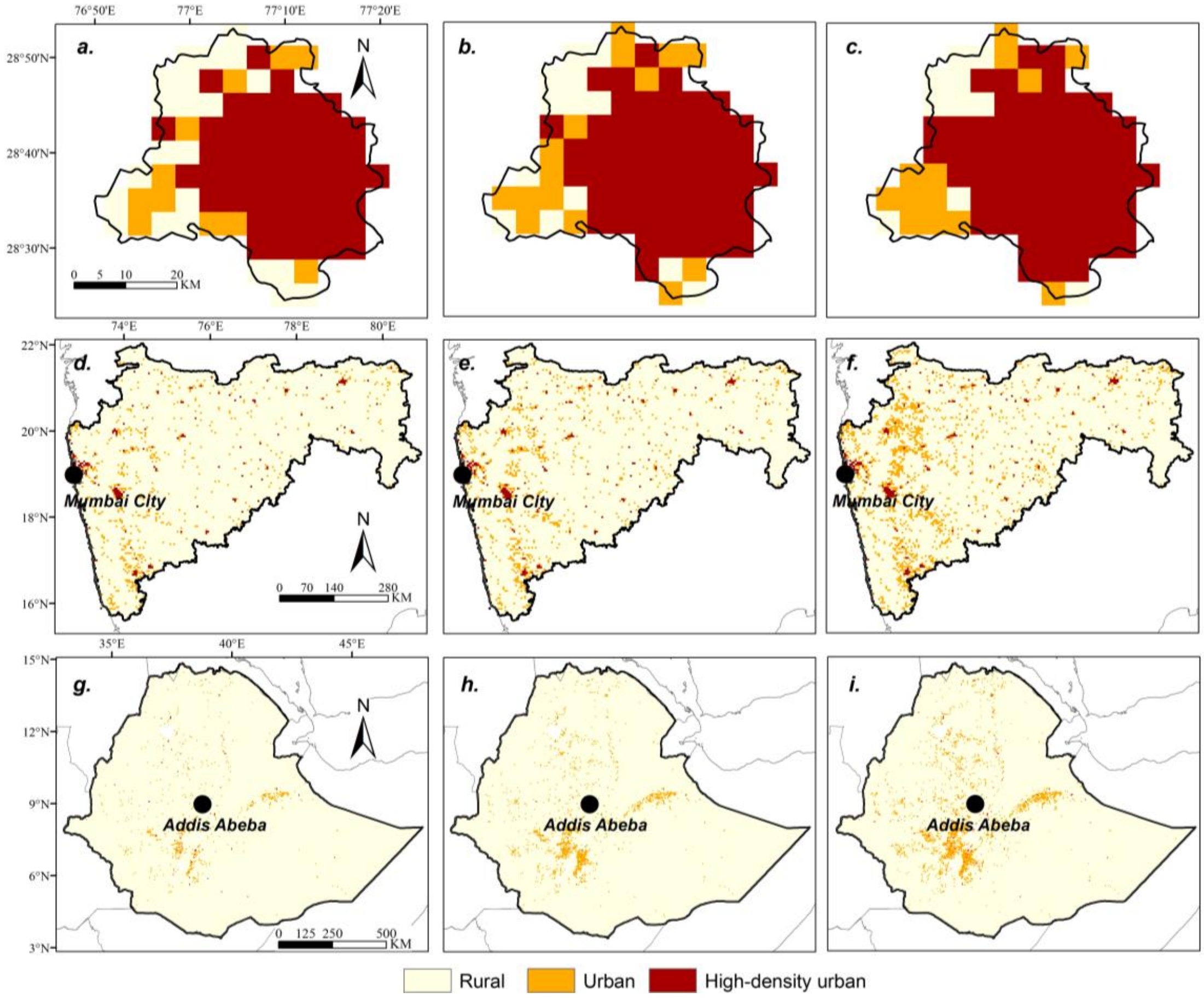


**Supplementary Fig. S7. The spatial distribution of rural, urban and high-density urban in New Delhi, India, Maharashtra State, India and Ethiopia in the three phases. a-c** New Delhi, India. **d-f** Maharashtra State, India. **g-i** Ethiopia.

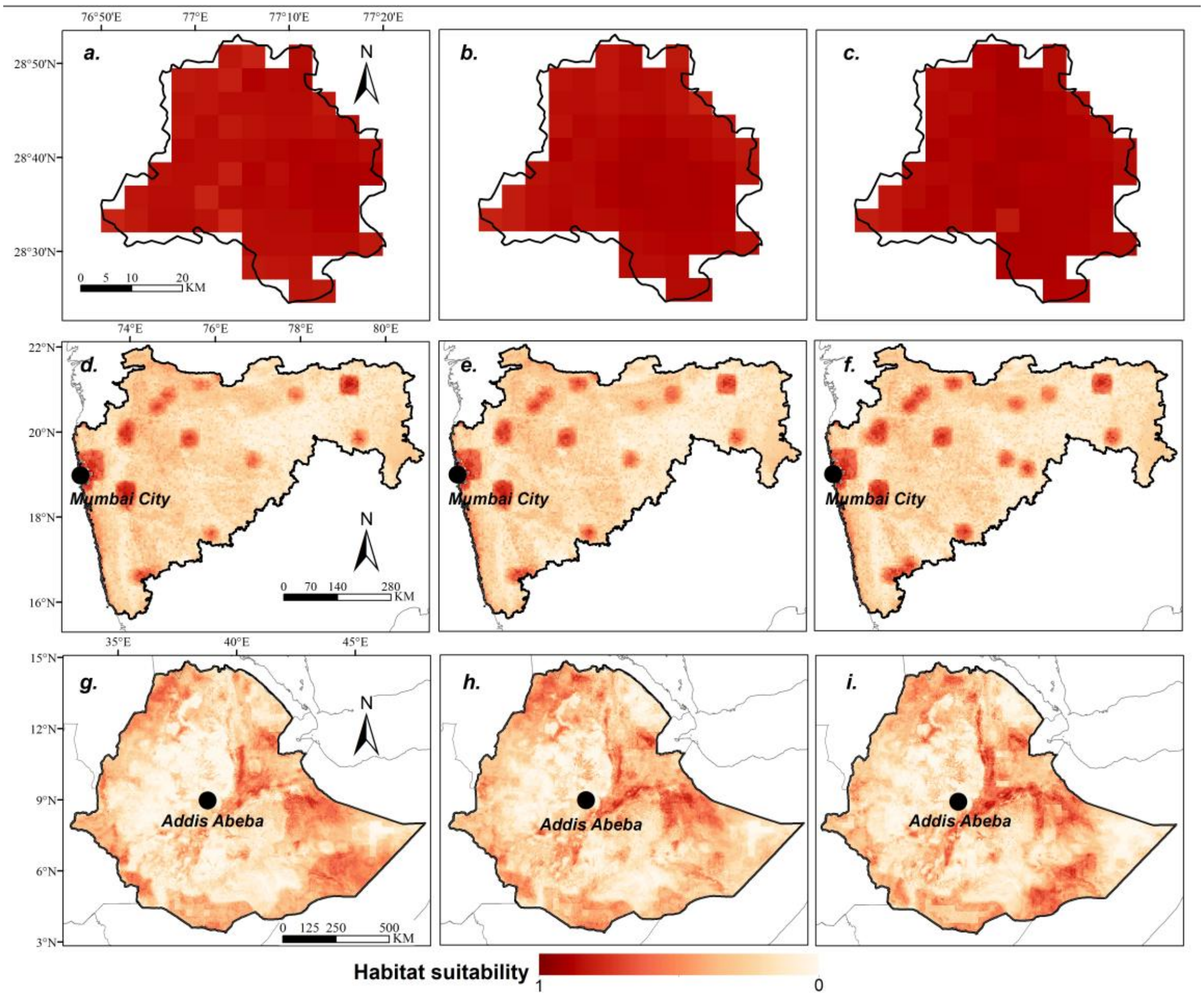


**Supplementary Fig. S8. The habitat suitability of *An. stephensi* in New Delhi, India, Maharashtra State, India and Ethiopia in the three phases. a-c** New Delhi, India. **d-f** Maharashtra State, India. **g-i** Ethiopia.

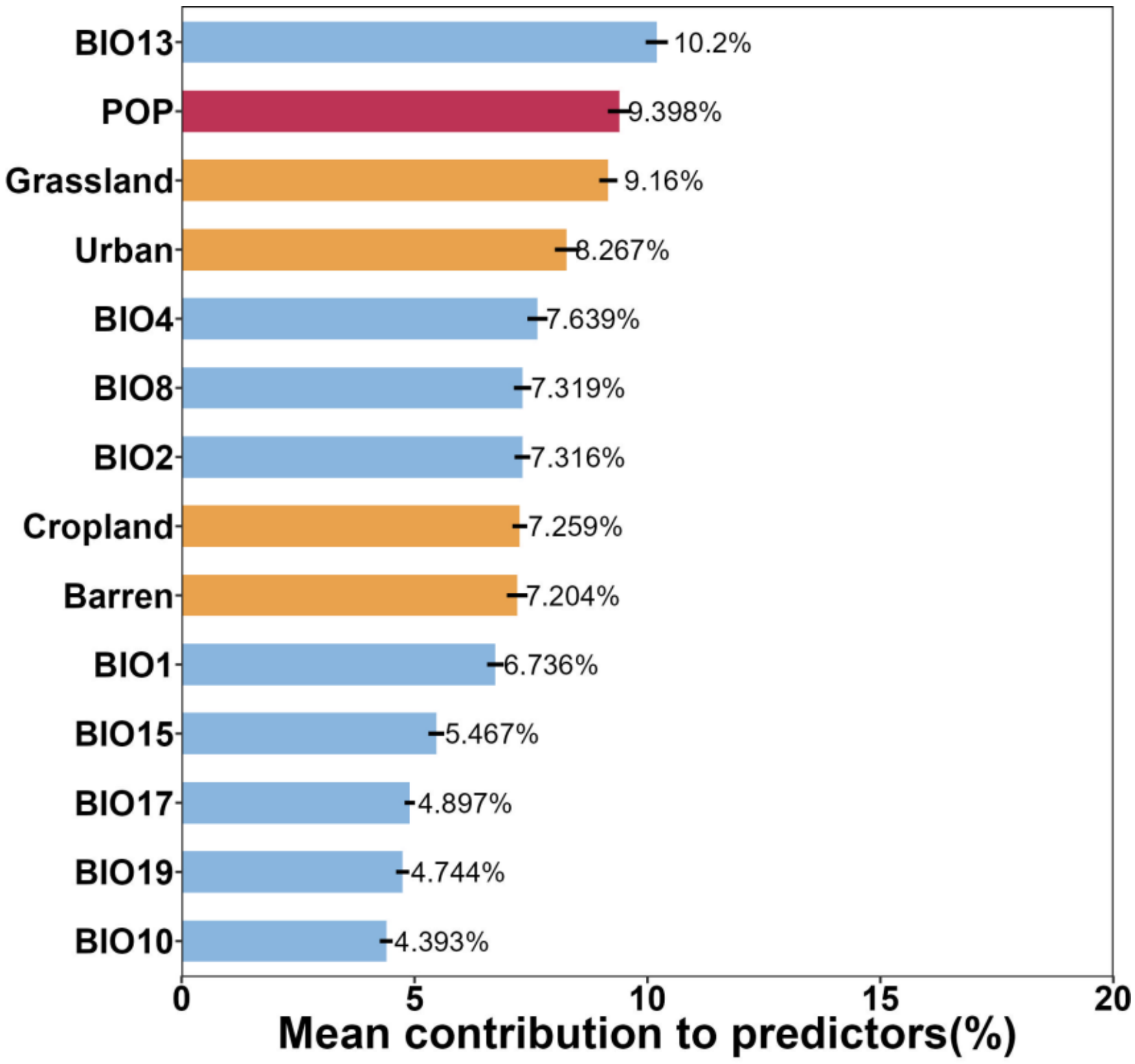


**Supplementary Fig. S9. The impact of factors on the habitat suitability of *An. stephensi* in Africa.** The factors in this BRT model are the same with the BRT model in overall study area. Bars represent the 95% confidence interval across 100 iterations. BIO1: annual mean temperature. BIO2: mean monthly temperature range. BIO4: temperature seasonality. BIO8: mean temperature of wettest quarter. BIO10: mean temperature of coldest quarter. BIO13: precipitation of wettest month. BIO15: precipitation seasonality. BIO17: precipitation of driest quarter. BIO19: precipitation of coldest quarter. Barren: share of barren. Cropland: share of cropland. Grassland: share of grassland. Urban: share of urban land. POP: population.

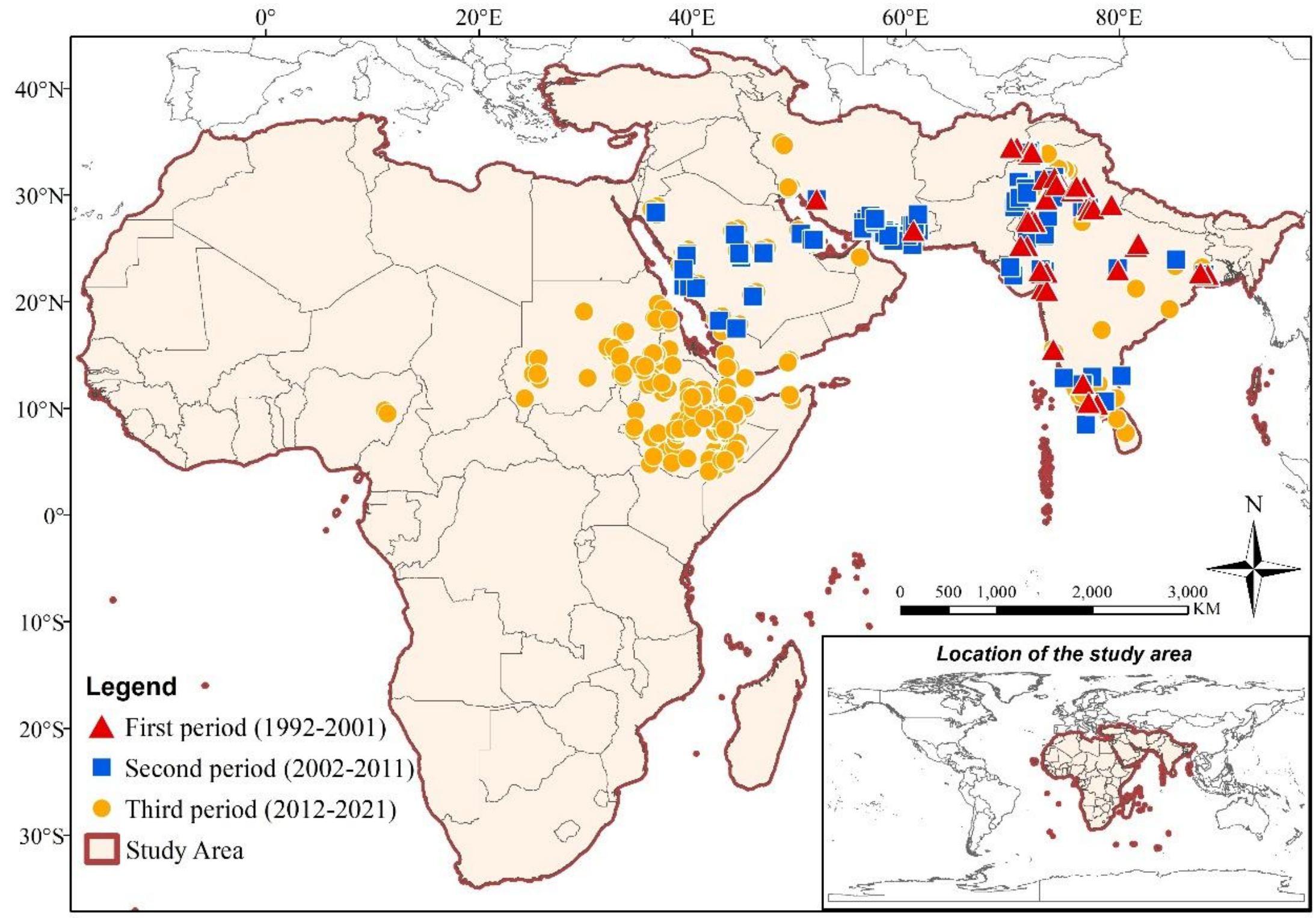


**Supplementary Fig. S10. The recorded points of *An. stephensi* during 1992-2021.** The numbers of occurrence points in three phases are 54, 132 and 446.

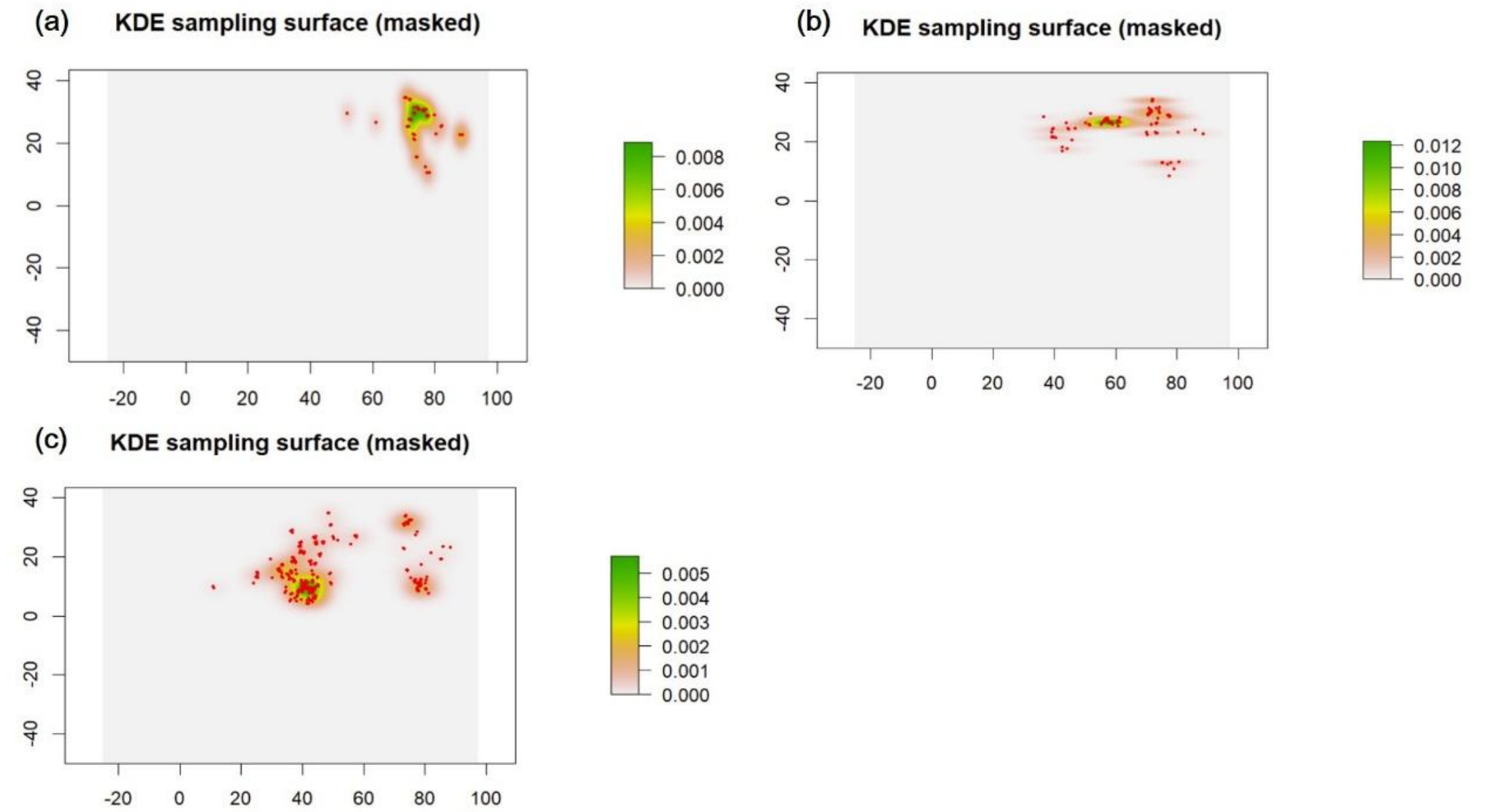


**Supplementary Fig. S11. The results of kernel density estimation (KDE) in the three phases.** The panel shows the probability value assigned to each grid based on the occurrence points. The red points are occurrence points in the three phases. **a** Phase I. **b** Phase II. **c** Phase III.

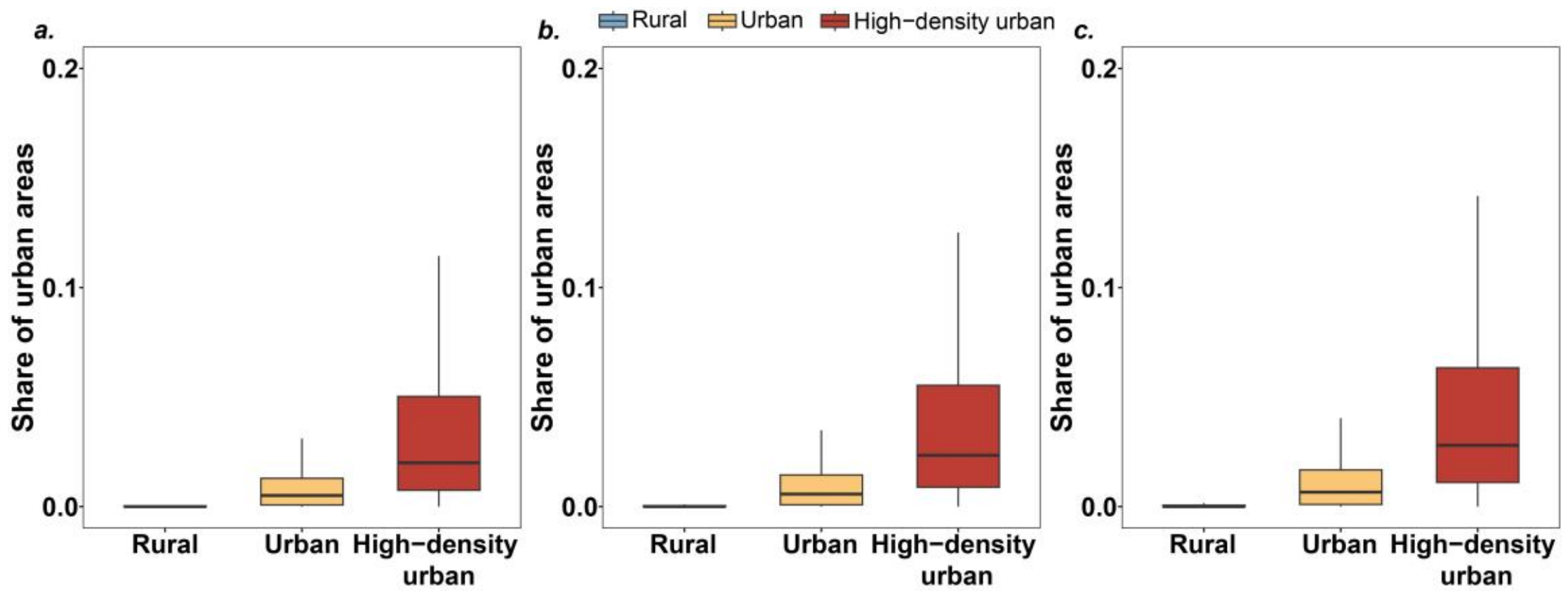


**Supplementary Fig. S12. The share of urban areas in rural, urban and high-density urban in the three phases.** The classification was obtained from the classification dataset[5]. **a** Phase I. **b** Phase II. **c** Phase III.

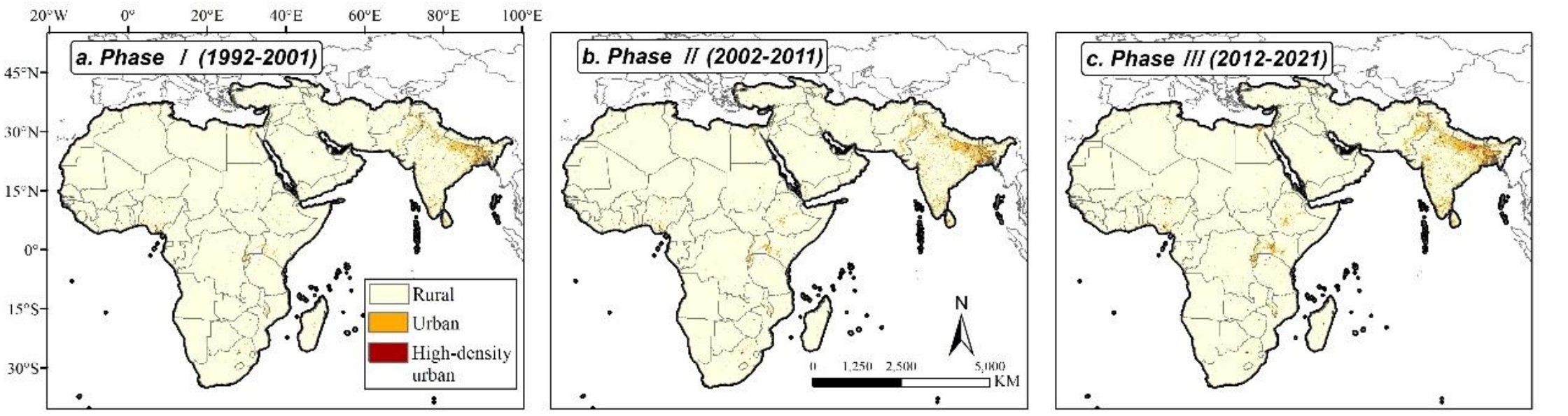


**Supplementary Fig. S13. The spatial distribution of rural, urban and high-density urban in the three phases.** The details about classification method are described in Methods section in the manuscript[5]. **a** Phase I. **b** Phase II. **c** Phase III.

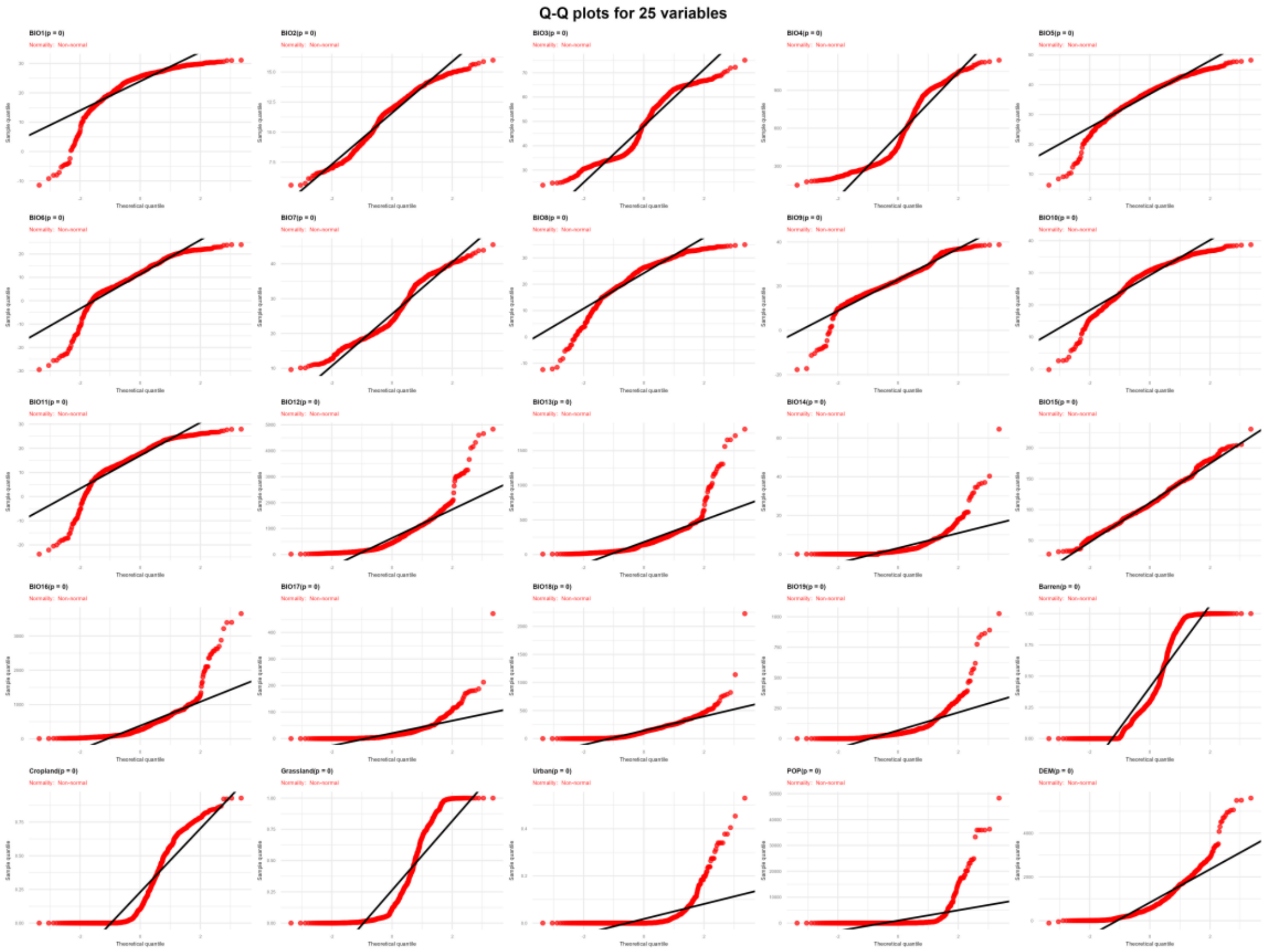


**Supplementary Fig. S14**. **The Q-Q plots of 25 variables in one of the ten datasets.** The red dots represent the ratio of theoretical quantiles to sample quantiles. The solid black line serves as a reference, depicting the theoretical normal distribution curve. The chart displays variable names, with the p value for the Shapiro-Wilk normality test. If $p>0.05$, it indicates that the null hypothesis cannot be rejected and the data is normal distribution, otherwise, it is not. BIO1: annual mean temperature. BIO2: mean monthly temperature range. BIO3: is thermality (BIO2/BIO7). BIO4: temperature seasonality. BIO5: max temperature of warmest month. BIO6: min temperature of coldest month. BIO7: Temperature annual range. BIO8: mean temperature of wettest quarter. BIO9: mean temperature of driest quarter. BIO10: mean temperature of warmest quarter. BIO11: mean temperature of coldest quarter. BIO12: annual precipitation. BIO13: precipitation of wettest month. BIO14: precipitation of driest month. BIO15: precipitation seasonality. BIO16: precipitation of wettest quarter. BIO17: precipitation of driest quarter. BIO18: precipitation of warmest quarter. BIO19: precipitation of coldest quarter. Barren: share of barren. Cropland: share of cropland. Grassland: share of grassland. Urban: share of urban land. POP: population. DEM: elevation.

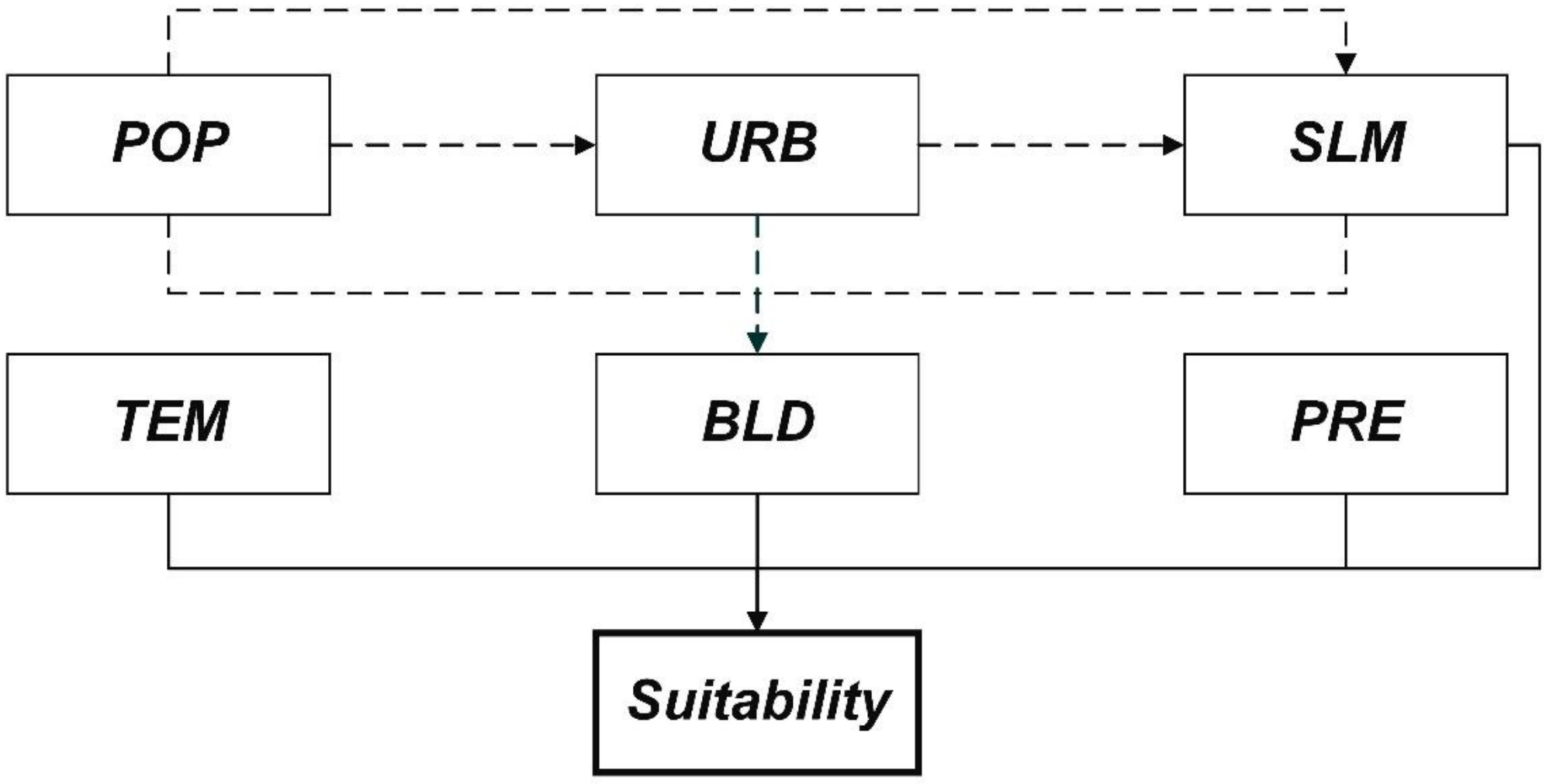


**Supplementary Fig. S15. The conceptual model of PLS-PM model in the study.** POP: population. URB: share of urban land. SLM: people living in slums in cities. BLD: Construction indexes. TEM: temperature indexes. PRE: precipitation indexes. Suitability: habitat suitability of *An. stephensi*.

## References


1. Liu, X., Wang, Y., Li, Y. & Wu, J. Quantifying the Spatio-Temporal Process of Township Urbanization: A Large-Scale Data-Driven Approach. *ISPRS Int. J. Geo-Inf.* **8**, 389 (2019).10.3390/ijgi8090389
2. Scheuer, S., Haase, D. & Volk, M. On the Nexus of the Spatial Dynamics of Global Urbanization and the Age of the City. *PLoS One*. **11**, e0160471 (2016).10.1371/journal.pone.0160471
3. Ochomo, E. O. et al. Detection of *Anopheles stephensi* Mosquitoes by Molecular Surveillance, Kenya. *Emerg. Infect. Dis.* **29**, 2498-2508 (2023).10.3201/eid2912.230637
4. Glidden, C. K. et al. Climate and urbanization drive changes in the habitat suitability of Schistosoma mansoni competent snails in Brazil. *Nat. Commun.* **15**, 4838 (2024).10.1038/s41467-024-48335-9
5. Schiavina, M., Melchiorri, M., Pesaresi, M., Jacobs-Crisioni, C. & Dijkstra, L. GHS-WUP-DEGURBA R2025A – GHS-WUP DEGURBA settlement layers, application of the Degree of Urbanisation methodology (stage I) to GHS-WUP-POP R2025A, multitemporal (1975-2100). (2025).1c049178-ab00-4bbc-b638-3e3c19daaacb
6. European Commission, A. S. O. O. Applying the Degree of Urbanisation - A methodological manual to define cities, towns and rural areas for international comparisons. *2021 Edition Publications Office of the European Union,* (2021).doi: 10.2785/706535
7. Liao, W. et al. Projections of land use changes under the plant functional type classification in different SSP-RCP scenarios in China. *Science Bulletin (Beijing)*. **65**, 1935-1947 (2020).10.1016/j.scib.2020.07.014
